# Revealing Causal Protein Biomarkers and Potential Therapeutic Targets for Histologic‐Specific Lung Cancer

**DOI:** 10.1111/jcmm.70866

**Published:** 2025-12-03

**Authors:** Wen Sun, Jingyang Liu, Jiayan Li, Ning Li, Xiaoyu Zhang, Changwei Li, Li Zhang, Yan He, Lijuan Wu, Xiao Wang, Jianguang Ji, Deqiang Zheng

**Affiliations:** ^1^ Department of Epidemiology and Health Statistics, School of Public Health Capital Medical University Beijing China; ^2^ Beijing Key Laboratory of environment and aging Capital Medical University Beijing China; ^3^ Beijing Fangshan District Center for Disease Prevention and Control Beijing China; ^4^ Department of Medical Data Science Center, Beijing Tsinghua Changgung Hospital, School of Clinical Medicine, Tsinghua Medicine Tsinghua University Beijing China; ^5^ Department of Epidemiology Tulane University School of Public Health and Tropical Medicine New Orleans USA; ^6^ School of Population Medicine and Public Health Chinese Academy of Medical Sciences and Peking Union Medical College Beijing China; ^7^ Center for Primary Health Care Research, Department of Clinical Sciences Malmö Lund University Sweden; ^8^ University Clinic Primary Care Skåne Region Skåne Sweden; ^9^ Faculty of Health Science University of Macau Taipa Macao SAR China

**Keywords:** drug target, lung cancer, Mendelian randomization, protein, protein quantitative trait loci

## Abstract

Considering the distinct etiological pathways and molecular characteristics of different lung cancer subtypes, it is crucial to develop subtype‐specific prevention strategies and therapeutic targets. This study aimed to identify protein biomarkers and potential therapeutic targets for specific subtypes of lung cancer by integrating population‐based observational studies and Mendelian randomisation (MR) analyses. The cohort study was conducted in the UK Biobank, including about 47,000 participants whose blood samples were measured for 2,923 unique proteins and who were followed for the development of lung cancer. Two‐sample MR was performed leveraging publicly available data from genome‐wide association studies (GWAS) and protein quantitative trait loci (pQTL). Proteins were prioritised based on consistent associations across logistic regression, MR, transcriptomic validation and sensitivity analyses. Tier 1 proteins passed all evaluations, including GP1BA (squamous cell carcinoma) and ACADSB (small cell carcinoma). Tier 2 proteins, supported by transcriptomic evidence but not sensitivity analyses, included AGRN, ITGB2, SEPTIN3 (adenocarcinoma) and DPP10 (squamous cell carcinoma). Tier 3 proteins, supported by logistic regression and MR only, included CD5L, GNPDA, ACAN, C7, DMP1, HEPH, CEACAM6, COX6B1, CPXM2 and IL12RB2. Druggability evaluation suggests that existing drugs targeting ITGB2, GP1BA, ACADSB and COX6B1 could potentially be repurposed for the treatment of specific lung cancer subtypes.

## 1 Introduction

Lung cancer was the leading cause of cancer incidence and mortality in 2022, with nearly 2.5 million new cases and over 1.8 million deaths worldwide [1]. The 5‐year survival rate for lung cancer is below 20% in most countries, primarily because the majority of cases are diagnosed with a poor pulmonary condition [1]. Early screening among high‐risk individuals has demonstrated a 16%–24% reduction in lung cancer mortality [2]. Despite significant advancements in targeted therapies and immunotherapy, the overall progression‐free survival rate for lung cancer patients remains suboptimal [3]. Therefore, earlier detection and more effective treatment strategies are urgent to improve survival and quality of life.

Lung cancer is a highly complex and heterogeneous disease, with distinct genomic profiles across its subtypes [4]. Non‐small‐cell carcinoma, which comprises the majority of cases, is categorised into two major types, adenocarcinoma and squamous cell carcinoma. These subtypes differ in their prognosis, underlying genomic alterations and therapeutic responses [5]. Adenocarcinoma has become the most common subtype globally in 2020, accounting for 50% of all lung cancer diagnoses [6, 7].

Small cell lung cancer accounts for 15% of all lung cancers and represents the most aggressive and lethal subtype [8]. Small cell lung cancer is a neuroendocrine carcinoma characterised by rapid proliferation, early and widespread metastasis and pronounced resistance to treatment, leading to its extremely poor prognosis [9]. Small cell lung cancer is distinct from non‐small cell lung cancer in clinical presentation, pathological features, biological behaviour and therapeutic approaches [10].

Genetic variations such as *TP53* have been reported to be present in different lung cancer subtypes, although the prevalence might vary. Besides these findings, subtype‐specific mutations have been reported, such as *MET* in adenocarcinoma, *FGFR1* and *FGFR3* in squamous cell carcinoma and *MYC* in small cell lung carcinoma [4]. Conducting subtype‐specific analyses is therefore essential to determine whether a protein plays a broad role in lung carcinogenesis or is specifically involved in the pathogenesis of a particular subtype.

Plasma proteins play a pivotal role in cancer biology, emerging as promising targets for early screening and therapeutic interventions [11]. Recent advancements in proteomic assays allow for the measurement of thousands of plasma proteins [12], leading to the discovery of novel biomarkers in various diseases through prospective studies [13]. Moreover, Mendelian randomisation (MR) analysis, which employs protein quantitative trait loci (pQTL) as instrumental variables, is increasingly utilised to explore causal relationships between proteins and tumours [14]. A previous two‐sample MR analysis identified five proteins, including TFPI, ICAM5, SFTPB, COL6A3 and EPHB1, associated with overall lung cancer risk [15]. However, this study did not evaluate the association with specific lung cancer subtypes, such as adenocarcinoma, squamous cell carcinoma and small cell carcinoma. Given the distinct etiological pathways and molecular characteristics of different lung cancer subtypes [3], it is crucial to assess whether these associations hold true across specific subtypes. Furthermore, the study lacked individual proteomic data, necessitating further validation to confirm these findings.

To bridge this gap, we integrated analyses of observational and genetic data for 2911 proteins with the risk of incident lung adenocarcinoma, squamous cell carcinoma and small cell carcinoma. The study aimed to (1) identify proteins associated with the risk of three site‐specific lung cancers through a longitudinal cohort study; (2) perform two‐sample MR analyses for each protein to assess their causal relevance for lung cancer subtypes; (3) explore the underlying mechanisms linking identified proteins with lung cancer subtypes using enrichment analyses; (4) assess the druggability of protein targets; (5) verify identified targets by analysing bulk RNA sequencing data; (6) evaluate the robustness of identified proteins through sensitivity analyses. This study could inform subtype‐specific prevention strategies and therapeutic targets for lung cancer.

## 2 Methods

### 2.1 Study Population

**FIGURE 1:**
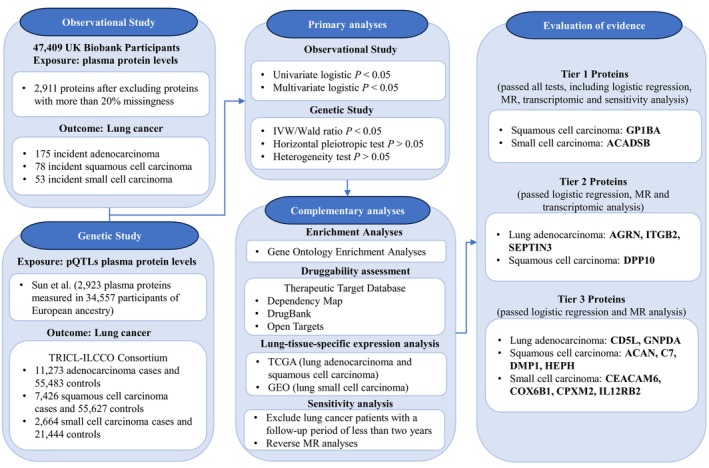
Summary of study design, methods of analysis and critical process. GEO, Gene Expression Omnibus; TCGA, The Cancer Genome Atlas; TRICL‐ILCCO, Transdisciplinary Research of Cancer in Lung of the International Lung Cancer Consortium.

This observational study was based on the UK Biobank which is a large‐scale prospective population‐based cohort study that recruited over 500,000 participants aged 40–69 years from England, Scotland, and Wales between 2006 and 2010 [16]. A schematic overview of the study design is depicted in Figure 1.

### 2.2 Protein Measurements

Proteomic profiling was performed on blood plasma samples from 54,219 participants by the UK Biobank Pharma Proteomics Project (UKB‐PPP) [12]. They utilised the antibody‐based Olink Explore 3072 Proximity Extension Assay, measuring 2941 protein analytes and capturing 2923 unique proteins. 53,029 individuals were kept after excluding those whose proteins presented with outliers, as well as likely sample swaps. The protein abundances were log_2_‐transformed and standardised to facilitate comparisons across proteins. Proteins were excluded if quality control resulted in more than 20% missingness, resulting in a set of 2911 proteins. For missing measurements, a mean‐imputed approach was employed [12].

### 2.3 Study Outcome

The outcomes of the observational study were lung adenocarcinoma, squamous cell carcinoma and small cell carcinoma, which were defined according to the 10th revision of the International Classification of Diseases (ICD‐10) codes (C33–C34) and the third edition of ICD for Oncology (ICD‐O‐3) morphology codes where appropriate. Patients with adenocarcinoma, squamous cell carcinoma and small cell carcinoma were identified from the National Cancer Register. Participants were followed from cohort entry to the occurrence of cancer, death, loss of follow‐up, or the last censoring date (31 December 2020 for England, 30 November 2021 for Scotland and 31 December 2016 for Wales). A detailed list, coding and case numbers for each site‐specific lung cancer are shown in Table S1.

### 2.4 Assessment of Covariates

Based on the previous studies [17], a group of possible confounders was adjusted in this study, including age, sex, ethnicity, townsend deprivation index (TDI) quintile, body mass index (BMI), highest educational attainment, alcohol intake frequency, smoking status, physical activity and family history of cancer. We used multiple imputations to estimate missing covariate data. The details of covariates are included in Table S2.

### 2.5 Statistical Analysis of Observational Study

The study cohort comprised 47,409 UK Biobank participants with available proteomic data after excluding 5620 individuals with preexisting cancers. To ensure the specificity of analyses for each lung cancer subtype, participants diagnosed with other lung cancer subtypes were excluded from the respective subtype analyses. Participant characteristics are presented in Table S3. For the observational analyses, logistic regression models were used to estimate both unadjusted and adjusted odds ratios (ORs) with 95% confidence intervals (CIs) for the association of each protein with the risk of each lung cancer subtype.

### 2.6 Summary‐Level Proteomic Data Source and Genetic Instruments Selection

Summary‐level genetic data for proteins were derived from the UK Biobank involving 34,557 participants of European ancestry [12]. Genetic variants associated with each protein were selected at the genome‐wide significance level (*p* < 5 × 10^−8^) and filtered for linkage disequilibrium with a cutoff of *r* ^2^ < 0.001 based on the European 1000 Genomes panel. Single nucleotide polymorphisms (SNPs) were excluded if they had the *F*‐statistic < 10 or were located in the major histocompatibility complex region (chromosome 6: 28,477,797–33,448,354) [18]. After applying these criteria, a total of 17,990 instruments and 2593 unique proteins were included.

### 2.7 Data Sources for Outcomes

Transdisciplinary Research of Cancer in Lung of the International Lung Cancer Consortium (TRICL‐ILCCO) data was accessed for GWAS lung adenocarcinoma (11,273 cases and 55,483 controls), squamous cell carcinoma (7426 cases and 55,627 controls) and small cell carcinoma (2664 cases and 21,444 controls) [19].

### 2.8 Two‐Sample Mendelian Randomisation Analysis

Statistical analysis of genetic study was conducted by two‐sample MR analyses (Figure S1). The inverse‐variance weighted (IVW) model or Wald ratio model (1 SNP) was conducted as the primary statistical method to determine the causality between genetically predicted protein levels and lung cancer subtypes. To ensure the robustness of the results, heterogeneity and pleiotropy analyses were assessed by using Cochran's *Q* test and MR–Egger intercept test. If the *p* for Cochran's *Q* test or MR–Egger intercept is less than 0.05, radial MR was conducted to detect and remove outliers by setting a threshold of 0.05 and using modified second‐order weights. Statistical analyses were performed using the TwoSampleMR (version 0.5.7) and RadialMR (version 1.1) packages in R (version 4.2.3).

### 2.9 Define Significant Causal Protein Targets

Significant causal protein targets were defined as proteins with high‐support evidence, meeting the criteria of (1) *p* for unadjusted analyses < 0.05, (2) *p* for adjusted analyses < 0.05, (3) *p* for two‐sample MR analyses < 0.05, and (4) consistent directionality between observational and genetic studies.

### 2.10 Enrichment Analyses

Gene ontology (GO) enrichment analyses [20] were conducted to investigate the biological functions and pathways of identified protein targets through cohort study and MR analysis for each lung cancer subtype using clusterProfiler package (v4.6.2). A *p* < 0.05 was defined as significant enrichment. To better understand the biological relevance of the enriched pathways to specific lung cancer subtypes, we searched PubMed for literature related to the top three pathways identified for each subtype.

### 2.11 Druggable Proteins Identification

To assess the druggability of identified proteins for each lung cancer subtype, we searched identified proteins in DrugBank (https://go.drugbank.com/), Dependency Map (https://depmap.org/portal/) and Open Targets (https://platform.opentargets.org/) databases to identify the potential drugs that target these proteins. Additionally, we conducted thorough searches of PubMed to review the research on approved drugs targeting identified proteins associated with lung cancer.

### 2.12 Verify the Identified Targets Using Bulk RNA Sequencing Data

To examine whether the identified casual protein‐coding genes through cohort study and MR analysis associated with each lung cancer subtype were differently expressed in tumour tissue, we conducted differential expression analyses using The Cancer Genome Atlas (TCGA) cohorts (https://portal.gdc.cancer.gov/) and Gene Expression Omnibus (GEO) dataset (https://www.ncbi.nlm.nih.gov/geo/). We analysed the bulk RNA sequencing data from the TCGA database, which includes 516 lung adenocarcinoma tissue samples and 59 normal lung tissue samples, as well as 501 squamous cell carcinoma tissue samples and 51 normal lung tissue samples, using the DESeq2 package (v1.38.3) to assess gene expression differences. TCGA database was converted to log_2_(counts+1). For small cell carcinoma, we employed the GSE30219 dataset from the GEO database, comprising 21 small cell carcinoma tissue samples and 14 normal tissue samples [21]. The limma package (v3.54.2) was employed to detect differentially expressed genes in tumour samples compared to normal controls. A false discovery rate (FDR) threshold of 0.05 was applied for multiple testing correction. Genes with a statistically significant differential expression were identified based on the threshold of *p*_FDR_ < 0.05 and |Log_2_ Fold Change (Log_2_FC)| ≥ 1.0. For genes with moderate expression changes (|Log_2_FC| > 0.5), we further applied Wilcoxon tests to verify the results [22]. A Wilcoxon test was used for the hypothesis tests, with the *p* < 0.05 considered statistically significant.

### 2.13 Sensitivity Analysis

To evaluate the robustness of the proteins identified through the cohort study and MR analyses for each lung cancer subtype, we conducted a sensitivity analysis by excluding individuals who have been diagnosed with lung cancer within 2 years after the baseline. This approach was designed to reduce the risk of reverse causation and improve the stability of the associations.

In addition, we performed reverse MR analyses to investigate whether genetic liability to specific lung cancer subtypes could causally influence circulating protein levels, rather than protein levels affecting cancer risk.

Based on the strength of evidence from comprehensive data analyses, we classified the identified proteins into three tiers. Tier 1 included proteins that passed all analyses, including logistic regression, MR, transcriptomic analysis and sensitivity analysis. Tier 2 included proteins that failed the sensitivity analysis but passed the others. Tier 3 included proteins that failed both the transcriptomic and sensitivity analyses (Figure 1).

## 3 Results

### 3.1 Observational Association of Proteins With Lung Cancer Subtypes

During a median follow‐up period of 11.5 years, 175 cases of adenocarcinoma, 78 cases of squamous cell carcinoma and 53 cases of small cell carcinoma were identified (Table S1). The participant characteristics are detailed in Table S3.

Among the 2911 plasma proteins analysed, significant associations (*p* < 0.05) were observed between 674 proteins and adenocarcinoma, 892 proteins and squamous cell carcinoma and 536 proteins and small cell carcinoma in the unadjusted model (Figure S2 and Table S4). In the fully adjusted models, 172 proteins remained significantly associated (*p* < 0.05) with adenocarcinoma, 328 proteins with squamous cell carcinoma and 189 proteins with small cell carcinoma (Figure S3 and Table S5).

### 3.2 Genetic Associations of Proteins With Lung Cancer Subtypes

**FIGURE 2:**
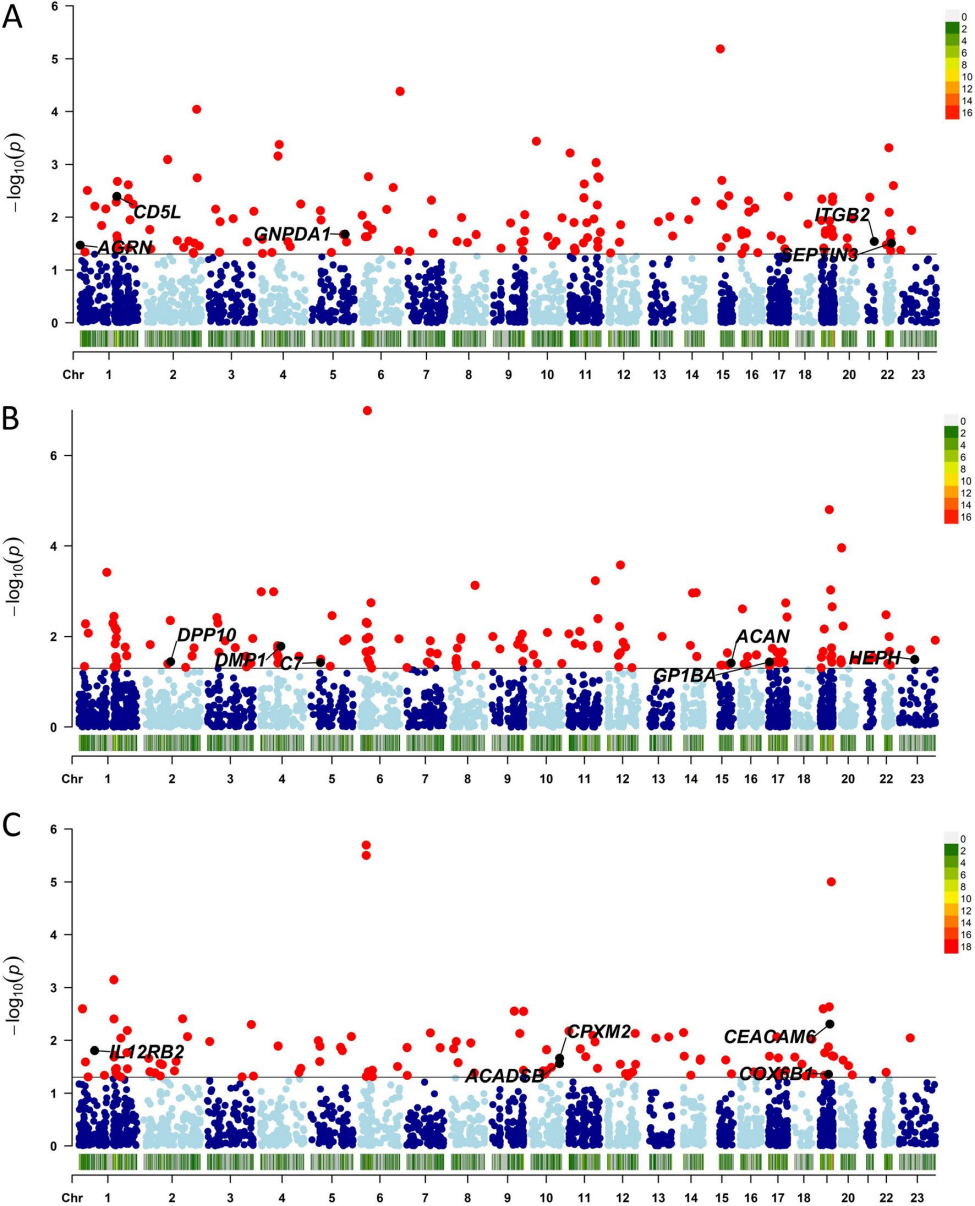
Manhattan plots of results from two‐sample Mendelian randomisation analyses. (A–C) represents the associations between proteins and adenocarcinoma, squamous cell carcinoma, small cell carcinoma, respectively. The black solid line indicates the *p*‐value threshold for significance (*p* < 0.05). Each dot represents a protein.

We performed two‐sample MR analyses to explore the potential causality between proteins and each lung cancer subtype. Our findings revealed that 156 proteins showed significant associations (*p* < 0.05) with adenocarcinoma, 158 proteins with squamous cell carcinoma and 123 proteins with small cell carcinoma (Figure 2 and Tables S6–S8).

**FIGURE 3:**
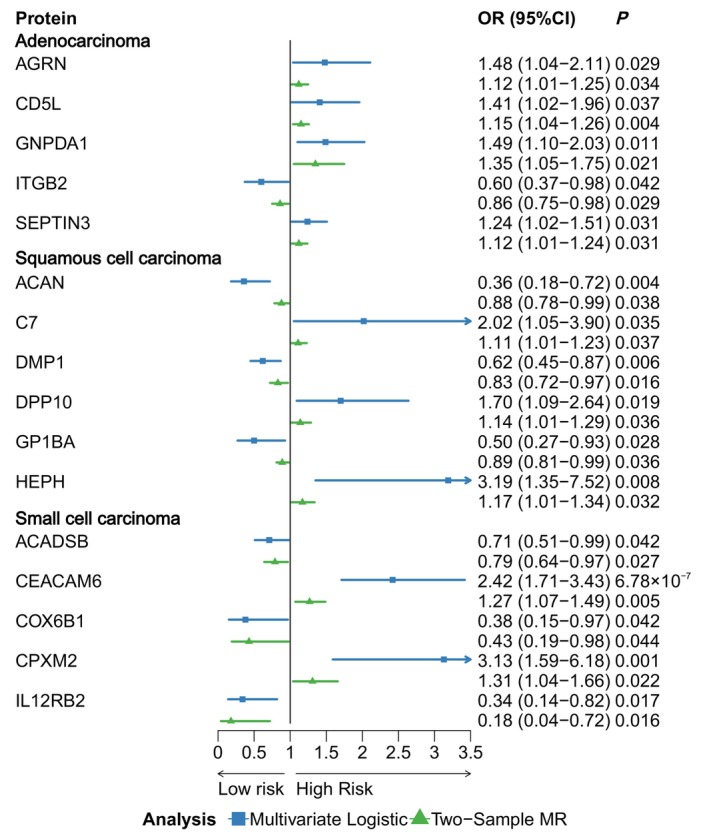
Forest plot of the multivariate logistic regression, and two‐sample Mendelian randomisation results between significant proteins and adenocarcinoma, squamous cell carcinoma and small cell carcinoma, respectively.

For the significant proteins identified in the observational analyses, Agrin (AGRN), CD5 antigen‐like (CD5L), glucosamine‐6‐phosphate isomerase 1 (GNPDA1), integrin beta‐2 (ITGB2) and neuronal‐specific septin‐3 (SEPTIN3) remained associated with lung adenocarcinoma in the two‐sample MR analyses. Aggrecan core protein (ACAN), complement component C7 (C7), Dentin matrix acidic phosphoprotein 1 (DMP1), inactive dipeptidyl peptidase 10 (DPP10), tumour necrosis factor receptor superfamily member 27 (EDA2R), platelet glycoprotein Ib alpha chain (GP1BA) and Hephaestin (HEPH) continued to be associated with squamous cell carcinoma, while short/branched chain specific acyl‐CoA dehydrogenase, mitochondrial (ACADSB), carcinoembryonic antigen‐related cell adhesion molecule 6 (CEACAM6), cytochrome *c* oxidase subunit 6B1 (COX6B1), inactive carboxypeptidase‐like protein X2 (CPXM2) and interleukin‐12 receptor subunit beta‐2 (IL12RB2) remained associated with small cell carcinoma in two‐sample MR analyses. These associations were consistent with the directionalities observed in the observational study (Figure 3).

The association between EDA2R and squamous cell carcinoma showed significant heterogeneity (*p* of Cochran's *Q* test = 0.026). However, after excluding three outliers using radial MR, the heterogeneity disappeared, and the association between EDA2R and squamous cell carcinoma was no longer observed (Table S7). For the other 16 significant proteins, no heterogeneity or pleiotropy was observed, as indicated by Cochran's *Q* test and the MR‐Egger intercept test (Table S9).

### 3.3 GO Pathway Analysis

For the five proteins identified through cohort study and MR analysis for lung adenocarcinoma, GO pathways analysis revealed that ITGB2 and AGRN were significantly enriched in the receptor clustering pathway (*p* = 8.88 × 10^−5^). Additionally, CD5L and ITGB2 were involved in the regulation of the immune effector process pathway (*p* = 0.004) and the cargo receptor activity pathway (*p* = 1.94 × 10^−4^) (Table S10). Additional pathways associated with proteins related to squamous cell carcinoma and small cell carcinoma are detailed in Tables S11 and S12. To assess the biological relevance of the enriched pathways to specific lung cancer subtypes, we reviewed published literature on the top three pathways identified for each subtype. A summary of relevant studies retrieved from PubMed is provided in Table S13.

### 3.4 Druggability Evaluation on the Identified Proteins

In druggability evaluation, we found that six of the 16 protein targets, GNPDA1, ITGB2, ACAN, GP1BA, ACADSB and COX6B1, have already been targeted for drug development (Table 1). Drugs targeting ITGB2 have been approved for regulating abnormal lipid levels (Simvastatin) and treating dry eye disease (Lifitegrast). Ibuprofen and Dexibuprofen, the drugs corresponding to GP1BA, are utilised to treat pain, fever and inflammation. Valproic acid, targeting ACADSB, has been developed to control complex partial seizures and absence seizures. For COX6B1, cholic acid is used to treat bile acid synthesis disorders. Drugs targeting GNPDA1 and ACAN are currently in the investigational and experimental stages. However, no drugs have been reported for targeting the remaining proteins, including AGRN, CD5L, SEPTIN3, C7, DMP1, DPP10, HEPH, CEACAM6, CPXM2 and IL12RB2. Previous studies have demonstrated their strong tumour‐inhibitory effect on lung cancer (Table S14).

**TABLE 1:** Druggability of proteins potentially causally associated with lung adenocarcinoma, squamous cell carcinoma and small cell carcinoma.

Protein	Drug or component name	Drug groups	Function
Lung adenocarcinoma			
GNPDA1	2‐Deoxy‐2‐amino glucitol‐6‐phosphate^a^	Experimental	—
	N‐acetyl‐d‐glucosamine‐6‐phosphate^a^	Experimental	—
	Beta‐d‐glucose^a^	Experimental	Fluid and nutrient replacement
ITGB2	Simvastatin^b^	Approved	Manage abnormal lipid levels
	BMS‐587101 (Lifitegrast)^b^, ^c^	Approved	Treat dry eye disease
Squamous cell carcinoma			
ACAN	Ilomastat^a^, ^b^	Experimental	Matrix metalloproteinase inhibitor
GP1BA	Egaptivon pegol^a^	Investigational	Treat thrombotic thrombocytopenic purpura and acute coronary syndromes
	Liposomal prostaglandin E1^a^	Investigational	Potent vasodilator and platelet inhibitor
	Ibuprofen^a^	Approved	Treat mild–moderate pain, fever and inflammation
	Dexibuprofen^a^	Approved	Treat pain and inflammation
	Anfibatide^c^	Investigational	Treat myocardial infarction and acquired thrombotic thrombocytopenic purpura
Small cell carcinoma			
ACADSB	Isoleucine^a^, ^b^	Investigational, nutraceutical	Component of total parenteral nutrition
	Valproic acid^a^, ^b^	Approved, investigational	Control complex partial seizures and both simple and complex absence seizures
COX6B1	Cholic acid^a^, ^b^	Approved	Treat bile acid synthesis disorders
	N‐formylmethionine^a^	Experimental	Effective in the initiation of protein synthesis

^a^ Drugbank.

^b^ Dependency Map.

^c^ Open Targets.

### 3.5 Verification of Identified Targets in the Bulk RNA Sequencing Data

**FIGURE 4:**
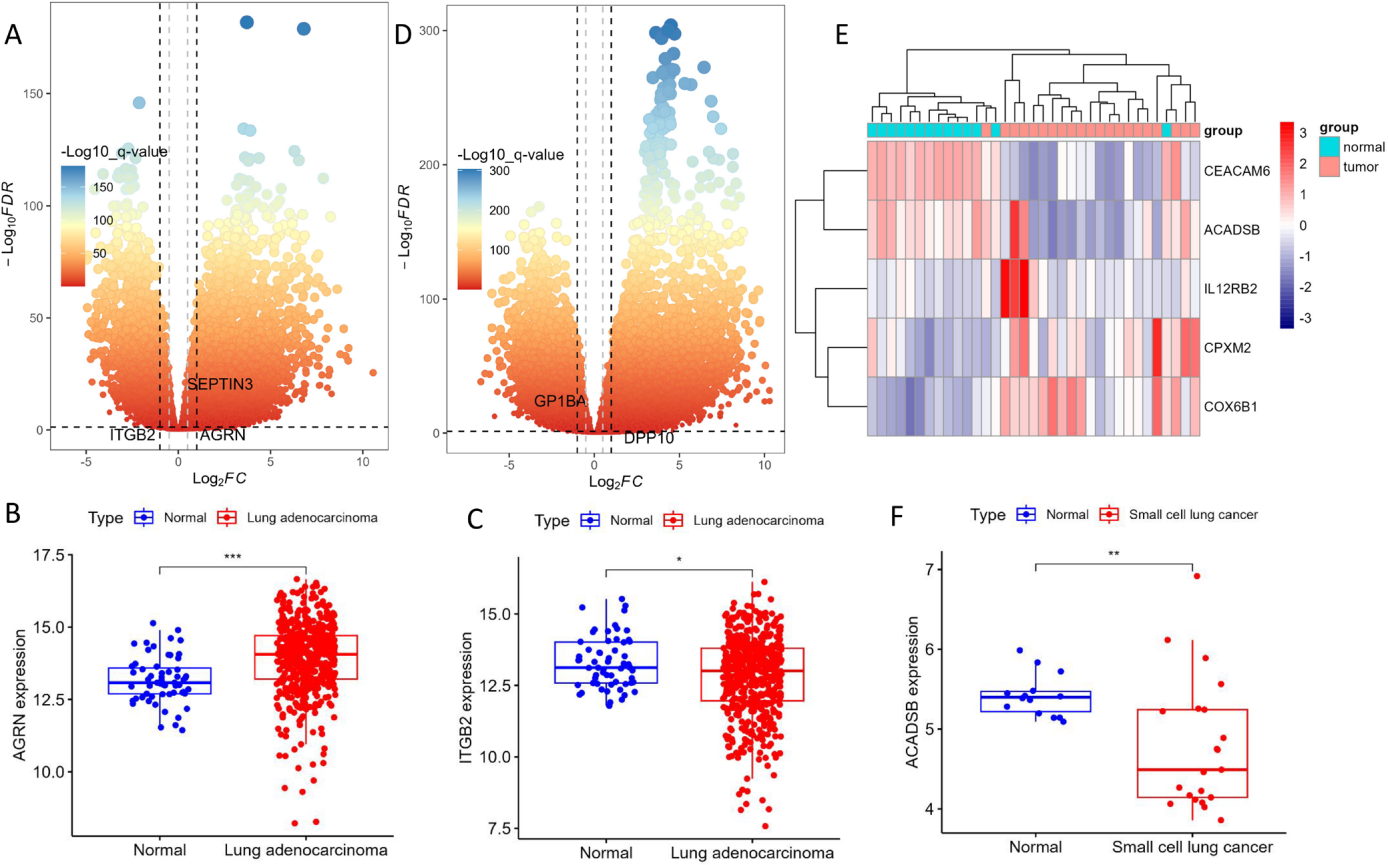
The results of verification of identified targets in the bulk RNA sequencing data. (A) Volcano plots of the differentially expressed genes between lung adenocarcinoma and normal lung tissues in TCGA datasets. (B) Comparison of *AGRN* expression between lung adenocarcinoma and normal tissues in TCGA datasets (Wilcoxon test). ***p < 0.001. (C) Volcano plots of the differentially expressed genes between lung squamous cell carcinoma and normal lung tissues in TCGA datasets. (D) Comparison of *ITGB2* expression between lung squamous cell carcinoma and normal tissues in TCGA datasets (Wilcoxon test). *p < 0.05. (E) Heatmap of the *ACADSB*, *CEACAM6*, *COX6B1*, *CPXM2* and *IL12RB2* expressed between lung small cell carcinoma and normal tissues in GEO datasets. (F) Comparison of *ACADSB* expression between lung small cell carcinoma and normal tissues in GEO datasets (Wilcoxon test). **p < 0.01.

We further verified the 16 candidate targets significantly associated with specific lung cancer subtypes in the cohort study and MR analysis using bulk RNA sequencing data. Analysis of the TCGA database revealed that *ITGB2* (Log_2_FC = −0.62, *p*_FDR_ = 3.53 × 10^−5^), *SEPTIN3* (Log_2_FC = 1.54, *p*_FDR_ = 2.03 × 10^−13^) and *AGRN* (Log_2_FC = 0.56, *p*_FDR_ = 5.41 × 10^−6^) exhibited different expression between lung adenocarcinoma and normal tissues, consistent with results of observational and genetic studies (Figure 4A and Table S15). As shown in Figure 4B,C, the relative expression levels of *AGRN* and *ITGB2* in lung adenocarcinoma samples were significantly different from those in normal tissues (*p* for Wilcoxon tests < 0.05). Additionally, we found that *DPP10* (Log_2_FC = 1.08, *p*_FDR_ = 0.007) and *GP1BA* (Log_2_FC = −1.21, *p*_fdr_ = 1.24 × 10^−11^) exhibited significantly different expression between squamous cell carcinoma and normal tissues (Figure 4D and Table S16). After normalising the GSE30219 dataset, the *ACADSB* gene exhibited lower expression in small cell lung tumour tissues compared to normal tissues (Log_2_FC = −0.64, *p*_FDR_ = 0.017 and *p* for Wilcoxon test < 0.01), which was consistent with results from logistic regression and MR analysis (Figure 4E,F and Table S17).

### 3.6 Sensitivity Analysis

After excluding individuals who have been diagnosed with lung cancer within 2 years after the baseline, several associations remained robust. GNPDA1 continued to be significantly associated with lung adenocarcinoma. DMP1, GP1BA and HEPH remained associated with lung squamous cell carcinoma. For small cell lung cancer, ACADSB, CEACAM6, COX6B1, CPXM2 and IL12RB2 continued to show significant associations (Table S18).

In the reverse MR analyses, AGRN and IL12RB2 demonstrated potential reverse causal effects, suggesting that genetic liability to lung adenocarcinoma and small cell carcinoma, respectively, may influence circulating levels of these proteins (Table S19).

Two proteins (GP1BA for squamous cell carcinoma, ACADSB for small cell carcinoma) passed all tests and were classified into tier 1. Four proteins (AGRN, ITGB2, SEPTIN3 for lung adenocarcinoma, DPP10 for squamous cell carcinoma) were classified into tier 2. Ten proteins (CD5L, GNPDA for lung adenocarcinoma, ACAN, C7, DMP1, HEPH for squamous cell carcinoma, CEACAM6, COX6B1, CPXM2, IL12RB2 for small cell carcinoma) were classified into tier 3 (Figure 1).

## 4 Discussion

This study provides a comprehensive examination of the correlations between 2911 plasma proteins and lung cancer subtypes, utilising both cohort study and two‐sample MR analyses. Through this analysis, we successfully identified specific proteins associated with different lung cancer subtypes: AGRN, CD5L, GNPDA, ITGB2, SEPTIN3 with adenocarcinoma; ACAN, C7, DMP1, DPP10, GP1BA, HEPH with squamous cell carcinoma; and ACADSB, CEACAM6, COX6B1, CPXM2, IL12RB2 with small cell carcinoma. Druggability evaluation highlighted four protein biomarkers, ITGB2, GP1BA, ACADSB and COX6B1, which are already targeted for dry eye disease, inflammation, seizure and bile acid synthesis disorder, respectively. Among the sixteen identified candidates, AGRN, ITGB2, SEPTIN3, DPP10, GP1BA and ACADSB were validated through bulk RNA sequencing data, underlining their therapeutic potential. Notably, GP1BA and ACADSB passed the sensitivity analyses and were classified as tier 1 proteins. These findings lay the groundwork for the development of more revolutionary treatment approaches.

These proteins revealed in this study can be used not only in early detection screening programmes to identify individuals at high risk for lung adenocarcinoma, squamous cell carcinoma and small cell carcinoma, enabling timely intervention and potentially improving survival rates, but also in developing novel therapeutics that potentially inhibit tumour growth, enhance the effectiveness of existing treatments, or overcome resistance to current therapies. Additionally, the repurposing of approved drugs targeting ITGB2, GP1BA, ACADSB and COX6B1 for the treatment of different lung cancer subtypes provides new therapeutic options, potentially leading to more effective treatments for patients with advanced lung cancer.

AGRN is a heparan sulphate proteoglycan that functions in the extracellular matrix, either as a transmembrane or secreted protein [23]. A previous study employing both single‐cell RNA sequencing and immunohistochemistry has demonstrated that AGRN is primarily expressed and secreted in lung adenocarcinoma cells [24]. A retrospective study of 120 lung adenocarcinoma patients provided robust clinical evidence that high AGRN expression correlates with a greater susceptibility to lymph node metastases and a poorer prognosis [24]. Additionally, an mRNA‐mining study identified AGRN as an independent indicator of poor prognosis and metastasis in lung adenocarcinoma [25]. AGRN directly interacts with NOTCH1 and increases the release of Notch intracellular structural domain 1 (NICD1), leading to the activation of the Notch pathway, which promotes the proliferation, migration and invasion of lung adenocarcinoma cells [24]. Integrins are vital cell surface receptors, composed of α and β subunits, that play an important role in cell survival, proliferation and migration [26]. The integrin‐β (ITGB) superfamily comprises eight members, ITGB1‐8 [26]. The mRNA expression and protein levels of ITGB2 are lower in lung adenocarcinoma tissues compared to normal tissues [27]. Furthermore, low expression of ITGB2 is linked to a poorer prognosis in non‐small‐cell lung cancer [27, 28]. Our study further supports the significant associations of AGRN and ITGB2 with lung adenocarcinoma by integrating a longitudinal cohort study and large‐scale MR analysis, emphasising their potential as biomarkers for lung adenocarcinoma pathogenesis, and their value in clinical assessments and targeted therapies. SEPTIN3, a member of the septin family, is primarily expressed in the brain and testis [29]. Although there is no direct evidence linking SEPTIN3 protein to lung adenocarcinoma risk, previous studies have shown that high SEPTIN3 expression promotes the progression of triple‐negative breast cancer [30, 31]. SEPTIN3 emerges as a novel target, opening new investigational avenues. Further epidemiological studies and experimental research are needed to verify our findings.

To date, DPP10 was identified to be associated with asthma [32] and lung function [33]. However, the function and mechanism of DPP10 in lung squamous cell carcinoma remain unclear. Only a xenograft mouse experiment has found the novel antisense lncRNA DPP10‐AS1 promoted lung tumour growth by upregulating DPP10 protein [34]. Our study highlights an association between DPP10 and a higher risk of lung squamous cell carcinoma, as evidenced by findings from a cohort study, MR analyses and bulk transcriptomic analyses. DPP10 could be a novel therapeutic target, offering a new avenue for lung squamous cell carcinoma therapy. Platelet glycoprotein Ib (GP1B) is a key receptor for platelet activation and aggregation [35]. Previous studies have shown homozygous or heterozygous variants in GP1BA can cause severe thrombocytopenia, giant platelets and a bleeding tendency [36, 37]. A review has provided strong evidence demonstrating the relationship between circulating platelet counts and the incidence and prognosis of various human malignancies, particularly lung cancer [38]. Currently, no study has directly shown a relationship between GP1BA protein level and lung squamous cell carcinoma risk. GP1BA is a known target for ibuprofen. Specifically, evidence suggests the potential repurposing of ibuprofen for lung cancer treatment [39].

Small cell lung cancer accounts for approximately 13%–15% of all lung cancer cases and remains one of the most lethal malignancies, with a 5‐year survival rate of less than 7% [40]. Small cell lung cancer is characterised by rapid proliferation, high angiogenesis, apoptosis imbalance and early metastasis [9]. There is an urgent need for more effective treatment strategies and early detection methods to improve the survival rates of small cell lung cancer patients. Our study identified five candidate proteins for small cell lung cancer: ACADSB, CEACAM6, COX6B1, CPXM2 and IL12RB2. These proteins could serve as targets for the development of new diagnostic and therapeutic strategies. Specifically, ACADSB, which was further validated through bulk transcriptomic analyses, may play a crucial role in small cell lung cancer pathogenesis and could be a promising target for precision medicine. ACADSB belongs to the acyl‐CoA dehydrogenase enzyme family, which plays a role in the metabolism of fatty acids and branched‐chain amino acids [41]. Previous studies have shown that ACADSB plays an important role in glioma [42], colorectal cancer [43], hepatocellular carcinoma [44], clear cell renal cell carcinoma [45] and non‐small cell lung cancer [46]. However, the association of ACADSB with small cell lung cancer has not yet been reported. This finding needs to be demonstrated by future research.

Some limitations of our analysis should be acknowledged. Firstly, we were unable to replicate the observational analyses independently in other cohorts due to the unavailability of data. Secondly, there may be a temporal gap between the collection and testing of UK Biobank plasma samples, which could affect sample quality, although evidence suggests that proteins remain stable with reasonable storage over time. Finally, this study mainly focused on proteins with available index pQTL signals at the genome‐wide significance threshold. This approach may have overlooked potential drug targets associated with proteins that did not meet these criteria.

## 5 Conclusions

In conclusion, our study elucidates the significant associations between specific proteins and distinct lung cancer subtypes, supported by comprehensive evidence from observational data, MR, transcriptomic validation and sensitivity analyses. Notably, GP1BA and ACADSB emerged as robust Tier 1 candidates. AGRN, ITGB2, SEPTIN3 and DPP10 were classified as Tier 2, while several other proteins demonstrated potential biological relevance. These findings not only spotlight novel therapeutic targets, particularly SEPTIN3, DPP10 and ACADSB, but also underscore the necessity for further mechanistic studies to fully understand their roles in the pathogenesis and treatment of specific lung cancer subtypes.

## Author Contributions

**Wen Sun:** conceptualization (equal), data curation (equal), formal analysis (equal), methodology (equal), visualization (equal), writing – original draft (lead). **Jingyang Liu:** data curation (equal), formal analysis (equal), methodology (equal), visualization (equal), writing – original draft (lead). **Jiayan Li:** data curation (equal), methodology (equal), validation (equal), writing – review and editing (equal). **Ning Li:** validation (equal), writing – review and editing (equal). **Xiaoyu Zhang:** validation (equal), writing – review and editing (equal). **Changwei Li:** writing – review and editing (equal). **Li Zhang:** writing – review and editing (equal). **Yan He:** writing – review and editing (equal). **Lijuan Wu:** data curation (equal), writing – review and editing (equal). **Jianguang Ji:** conceptualization (equal), data curation (equal), methodology (equal), supervision (equal), writing – review and editing (equal). **Deqiang Zheng:** conceptualization (equal), data curation (equal), methodology (equal), project administration (equal), supervision (equal), writing – original draft (equal), writing – review and editing (equal). **Xiao Wang:** writing – review and editing (equal).

## Ethics Statement

The UK Biobank data were obtained with approval from the North West Multi‐Center Research Ethics Committee (MREC). This research was conducted using resources from the UK Biobank under approved application number 95259.

## Consent

No patient‐identifying information is included in this article; therefore, consent to publish was not required.

## Conflicts of Interest

The authors declare no conflicts of interest.

## Supporting information


**Figure S1:** Study design for two‐sample Mendelian randomisation analysis.
**Figure S2:** Volcano plots of the univariate logistic regression results. The association between 2911 plasma proteins and the risk of (A) lung adenocarcinoma, (B) squamous cell carcinoma, (C) small cell carcinoma.
**Figure S3:** Volcano plots of the multivariate logistic regression results. The association between 2911 plasma proteins and the risk of (A) lung adenocarcinoma, (B) squamous cell carcinoma, (C) small cell carcinoma.

**Table S1:** Coding and number of lung cancer incident cases among UK Biobank participants.
**Table S2:** Definition of variables.
**Table S3:** Baseline characteristics of participants.
**Table S4:** Univariate logistic results for lung adenocarcinoma, squamous cell carcinoma and small cell carcinoma.
**Table S5:** Multivariate logistic results for lung adenocarcinoma, squamous cell carcinoma and small cell carcinoma.
**Table S6:** Two‐sample Mendelian randomisation results for lung adenocarcinoma.
**Table S7:** Two‐sample Mendelian randomisation results for lung squamous cell carcinoma.
**Table S8:** Two‐sample Mendelian randomisation results for lung small cell carcinoma.
**Table S9:**. The results of horizontal pleiotropic and heterogeneity tests for identified proteins on lung adenocarcinoma, squamous cell carcinoma and small cell carcinoma.
**Table S10:**. Results of gene ontology (GO) enrichment of five identified lung adenocarcinoma related proteins.
**Table S11:** Results of gene ontology (GO) enrichment of six identified lung squamous cell carcinoma related proteins.
**Table S12:** Results of gene ontology (GO) enrichment of five identified lung small cell carcinoma related proteins.
**Table S13:** (a) Biological functions of proteins related to lung adenocarcinoma supported by GO analysis. (b) Biological functions of proteins related to lung squamous cell carcinoma supported by GO analysis. (c) Biological functions of proteins related to small cell lung carcinoma supported by GO analysis.
**Table S14:** Characteristics of studies about drugs targeting identified proteins on lung cancer subtypes.
**Table S15:** Detailed information of the differentially expressed genes between lung adenocarcinoma and normal samples in TCGA dataset.
**Table S16:** Detailed information of the differentially expressed genes between lung squamous cell carcinoma and normal samples in TCGA dataset.
**Table S17:** Detailed information of the differentially expressed genes between lung small cell carcinoma and normal samples in GEO dataset.
**Table S18:** (a) Sensitivity analysis of logistic results for lung adenocarcinoma. (b) Sensitivity analysis of logistic results for lung squamous cell carcinoma. (c) Sensitivity analysis of logistic results for lung small cell carcinoma.
**Table S19:** Reverse Two‐sample Mendelian randomisation results for lung cancer subtypes.

## Data Availability

Data of observational study is available upon reasonable request. Data of genetic study can be downloaded from https://metabolomips.org/ukbbpgwas/ and https://www.ebi.ac.uk/gwas/.
