# Supplementary material for: Revealing Causal Protein Biomarkers and Potential Therapeutic Targets for Histologic‐Specific Lung Cancer
Source: J Cell Mol Med. 2025 Dec 3;29(23):e70866. doi: 10.1111/jcmm.70866 (PMC12675135; doi:10.1111/jcmm.70866)
Supplement: Supplementary file 1 — Figure S1: Study design for two‐sample Mendelian randomisation analysis. Figure S2: Volcano plots of the univariate logistic regression results. The association between 2911 plasma proteins and the risk of (A) lung adenocarcinoma, (B) squamous cell carcinoma, (C) small cell carcinoma. Figure S3: Volcano plots of the multivariate logistic regression results. The association between 2911 plasma proteins and the risk of (A) lung adenocarcinoma, (B) squamous cell carcinoma, (C) small cell carcinoma. [file JCMM-29-e70866-s002.docx]

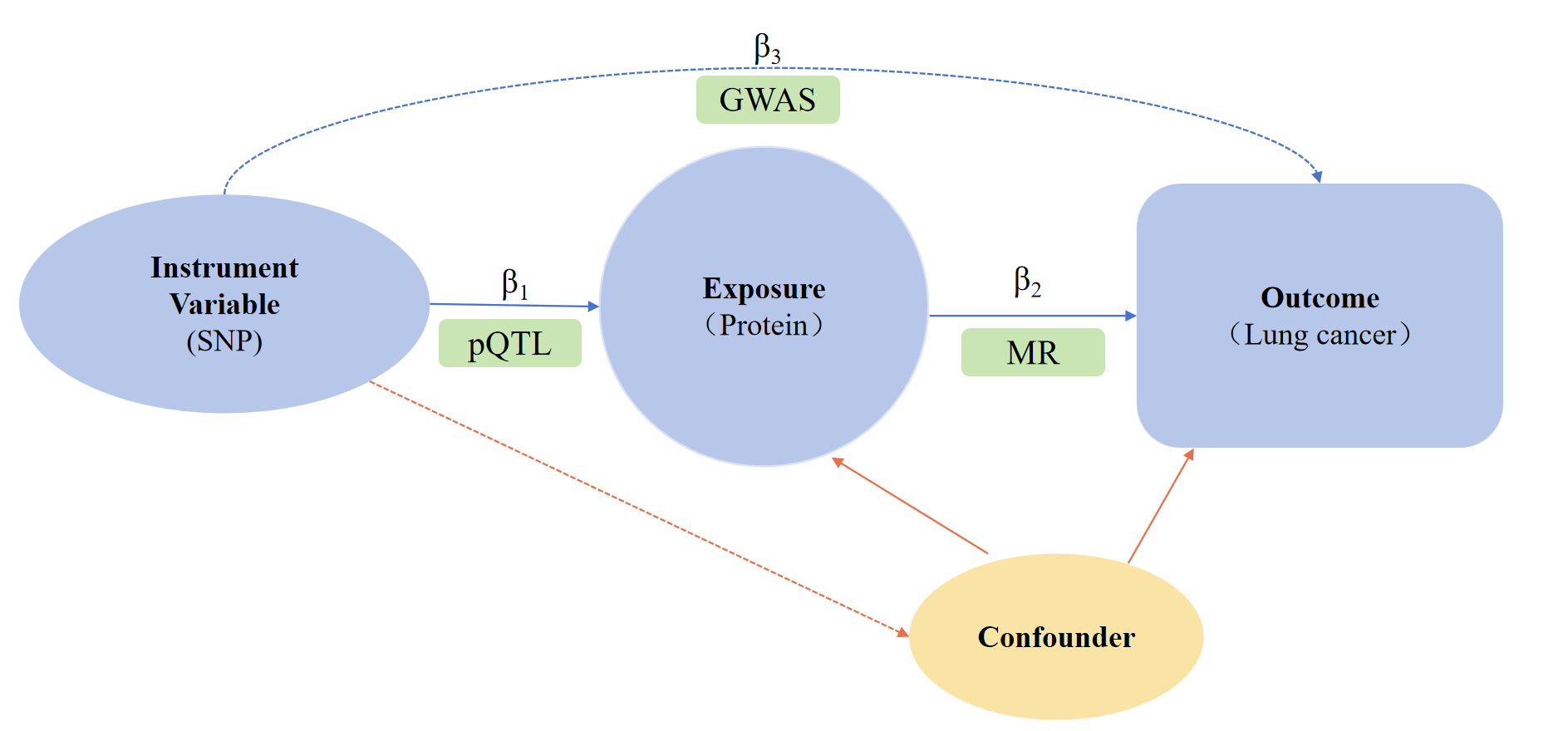


**Supplementary figure 1.** Study design for two-sample Mendelian randomization analysis.

β_1_ is the association between SNP and protein. β_3_ is the association between SNP and lung cancer outcome. β_2_ is the association between protein and lung cancer outcome.


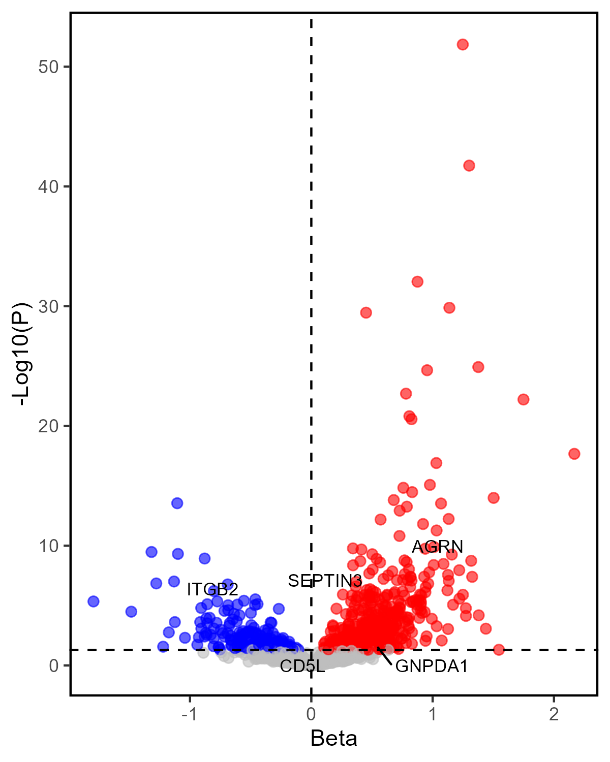

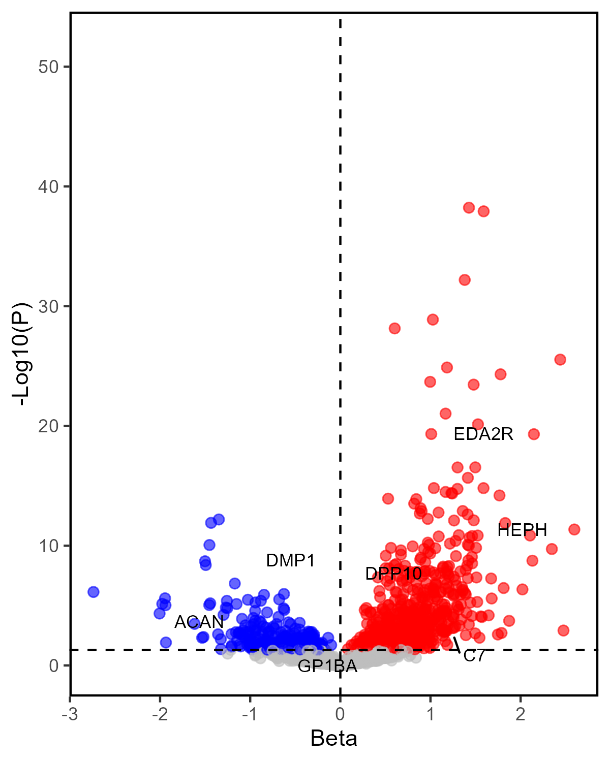

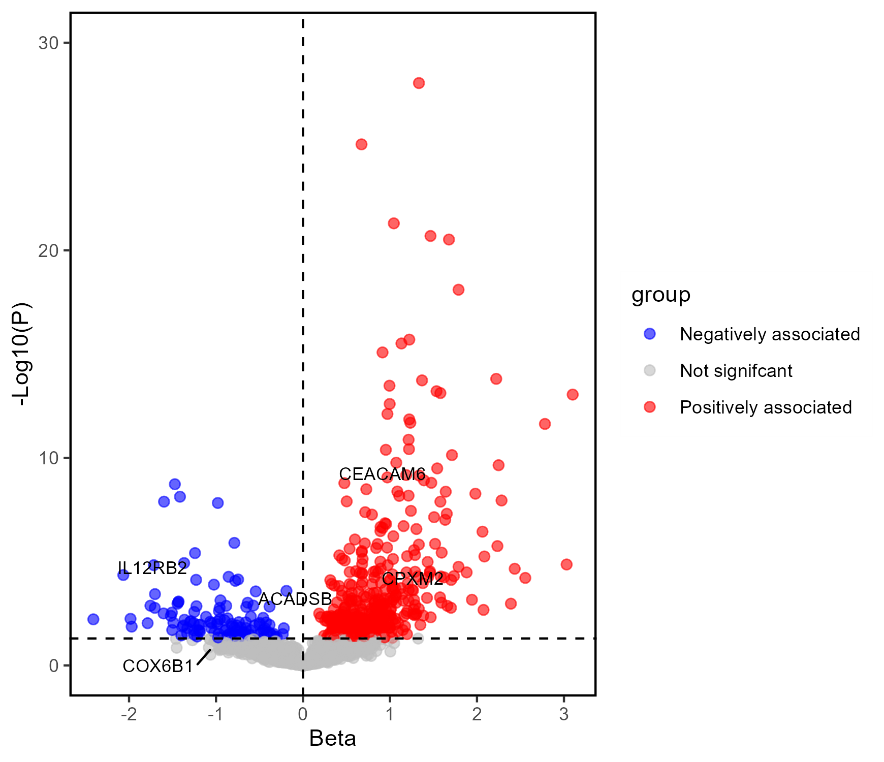


C

B

A

**Supplementary figure 2.** Volcano plots of the Univariate Logistic Regression results. The association between 2911 plasma proteins and the risk of **A** Lung adenocarcinoma, **B** Squamous cell carcinoma, **C** Small cell carcinoma.


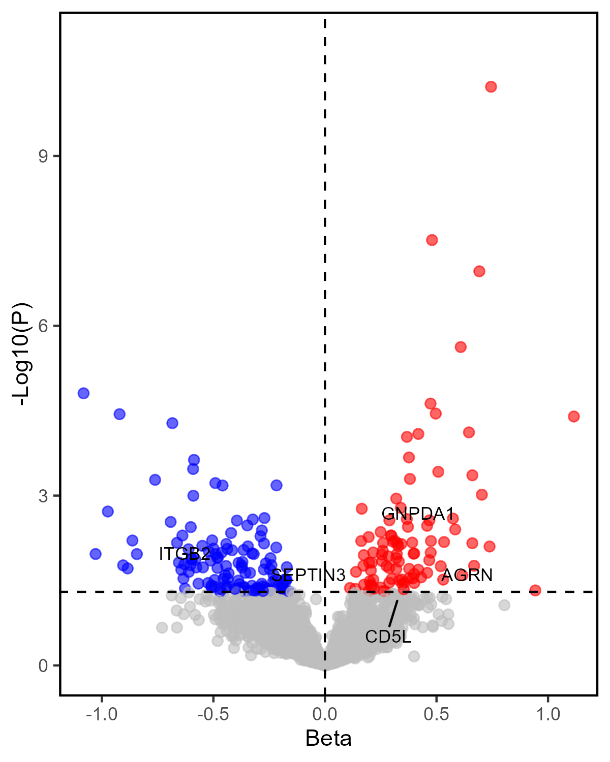

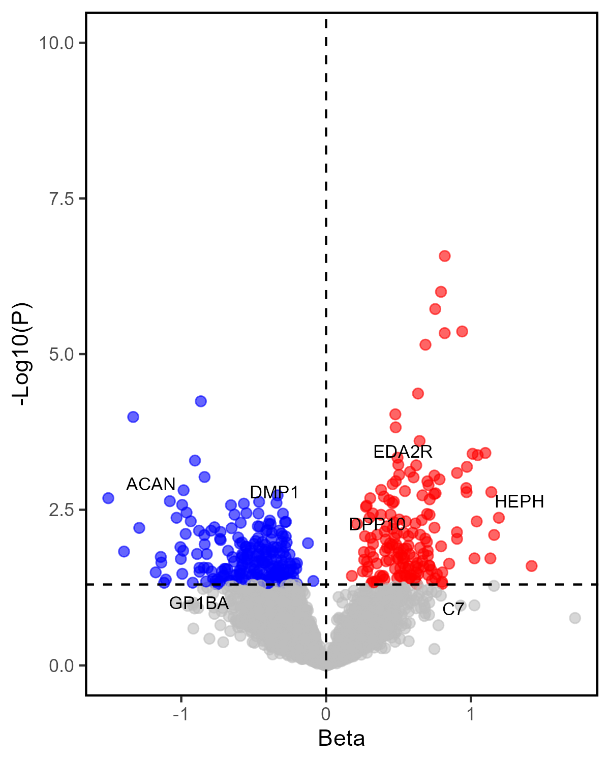

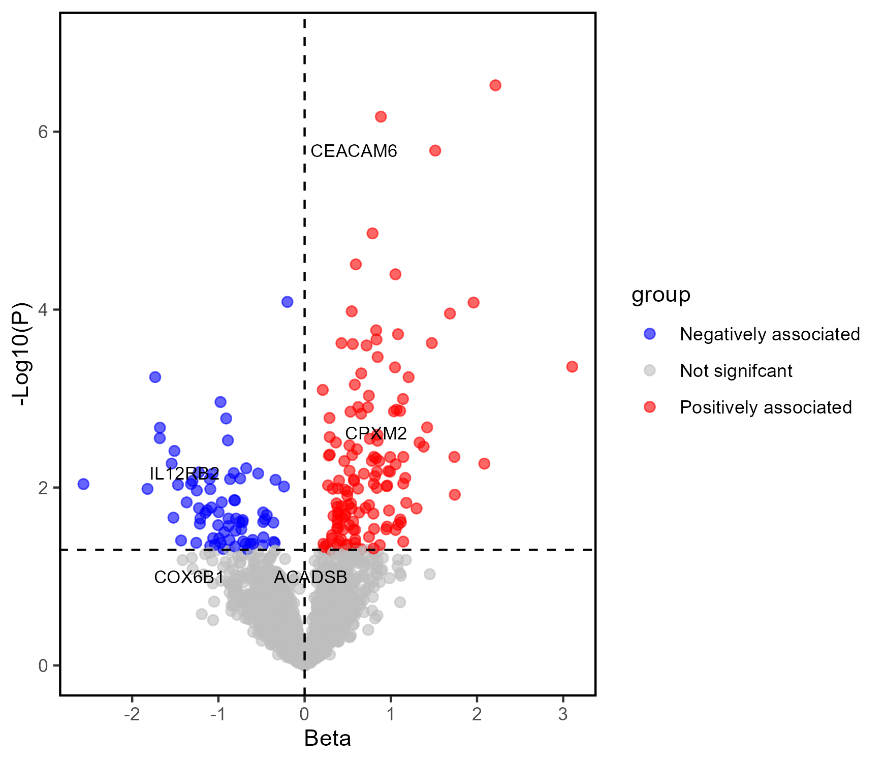


C

A

B

**Supplementary figure 3.** Volcano plots of the Multivariate Logistic Regression results. The association between 2911 plasma proteins and the risk of **A** Lung adenocarcinoma, **B** Squamous cell carcinoma, **C** Small cell carcinoma.
