# Supplementary material for: Revealing Causal Protein Biomarkers and Potential Therapeutic Targets for Histologic‐Specific Lung Cancer
Source: J Cell Mol Med. 2025 Dec 3;29(23):e70866. doi: 10.1111/jcmm.70866 (PMC12675135; doi:10.1111/jcmm.70866)
Supplement: Supplementary file 2 — Table S1: Coding and number of lung cancer incident cases among UK Biobank participants. Table S2: Definition of variables. Table S3: Baseline characteristics of participants. Table S4: Univariate logistic results for lung adenocarcinoma, squamous cell carcinoma and small cell carcinoma. Table S5: Multivariate logistic results for lung adenocarcinoma, squamous cell carcinoma and small cell carcinoma. Table S6: Two‐sample Mendelian randomisation results for lung adenocarcinoma. Table S7: Two‐sample Mendelian randomisation results for lung squamous cell carcinoma. Table S8: Two‐sample Mendelian randomisation results for lung small cell carcinoma. Table S9:. The results of horizontal pleiotropic and heterogeneity tests for identified proteins on lung adenocarcinoma, squamous cell carcinoma and small cell carcinoma. Table S10:. Results of gene ontology (GO) enrichment of five identified lung adenocarcinoma related proteins. Table S11: Results of gene ontology (GO) enrichment of six identified lung squamous cell carcinoma related proteins. Table S12: Results of gene ontology (GO) enrichment of five identified lung small cell carcinoma related proteins. Table S13: (a) Biological functions of proteins related to lung adenocarcinoma supported by GO analysis. (b) Biological functions of proteins related to lung squamous cell carcinoma supported by GO analysis. (c) Biological functions of proteins related to small cell lung carcinoma supported by GO analysis. Table S14: Characteristics of studies about drugs targeting identified proteins on lung cancer subtypes. Table S15: Detailed information of the differentially expressed genes between lung adenocarcinoma and normal samples in TCGA dataset. Table S16: Detailed information of the differentially expressed genes between lung squamous cell carcinoma and normal samples in TCGA dataset. Table S17: Detailed information of the differentially expressed genes between lung small cell carcinoma and normal samples in GEO dataset. Table [file JCMM-29-e70866-s001.docx]

**Supplemental table 1. Coding and number of lung cancer incident cases among UK Biobank participants.**

| **Cancer Site** | **ICD-10** | **ICD-O-3** | **Number of incident cases** |
| --- | --- | --- | --- |
| Adenocarcinoma | C33-C34 | 8140 8211 8230 8231 8250-8260 8323 8480-8490 8550 8551 8552 8570-8574 8576 | 175 |
| Squamous Cell Carcinoma |  | 8050-8078 8083 8084 | 78 |
| Small Cell Carcinoma |  | 8041-8045 | 53 |

| **Supplemental table 2. Definition of variables.** | | |
| --- | --- | --- |
| **Variables** | **Description** | **Details** |
| Age | Age at recruitment | Continuous variable |
| Sex | Sex | Binary variable: male, female |
| Ethnicity | Ethnic background | Polytomous variable: White, Asian, Black, Mixed, Others |
| Townsend deprivation index | Townsend deprivation index at recruitment | Polytomous variable: categorized by quintile |
| Smoking status | Smoking status | Polytomous variable: Never, Previous, Current |
| Alcohol intake | Alcohol intake frequency | Polytomous variable: almost daily, 3-4 times a week, 1-2 times a week, 1-3 times a month, special occasions only, never |
| Body mass index | Body mass index | Polytomous variable: underweight (<18.5 kg/m^2^), normal (18.5–24.9 kg/m^2^), overweight (25–29.9kg/m^2^), obese (≥30 kg/m^2^) |
| Highest educational attainment | Qualifications | Polytomous variable: college or university degree, A levels or equivalent, O levels or equivalent, CESs or equivalent, NVQ or HND or HNC or equivalent, other professional qualifications, none of the above |
| Physical activity | Summed MET minutes per week for all activity | Polytomous variable: low (<10 excess MET-hours per week), moderate: (10–49.9 excess MET-hours per week), high: (≥50 excess MET-hours per week) |
| Family history of cancer | Illnesses of father, Illnesses of mother, Illnesses of siblings, Type of cancer: ICD10, Type of cancer: ICD9, Diagnoses - ICD10, Diagnoses – ICD9, GP clinical event records, Cancer code, self-reported | Binary variable: yes, no |

| **Supplemental table 3. Baseline characteristics of participants.** | | | | | | | | | | | |
| --- | --- | --- | --- | --- | --- | --- | --- | --- | --- | --- | --- |
|  | **Covariates** | **Non-Lung cancer** |  | **Adenocarcinoma** | |  | **Squamous cell carcinoma** | |  | **Small cell carcinoma** | |
|  |  |  |  | **adenocarcinoma** | **Overall** |  | **squamous cell carcinoma** | **Overall** |  | **small cell carcinoma** | **Overall** |
| N |  | 46824 |  | 175 | 46999 |  | 78 | 46902 |  | 53 | 46877 |
| Age (mean (SD)) |  | 56.4 (8.2) |  | 61.7 (6.1) | 56.4 (8.2) |  | 62.3 (5.3) | 56.4 (8.2) |  | 61.1 (6.1) | 56.4 (8.2) |
| Sex (%) | Female | 24805 (53) |  | 88 (50.3) | 24893 (53.0) |  | 29 (37.2) | 24834 (52.9) |  | 33 (62.3) | 24838 (53) |
|  | Male | 22019 (47) |  | 87 (49.7) | 22106 (47.0) |  | 49 (62.8) | 22068 (47.1) |  | 20 (37.7) | 22039 (47) |
| Ethnicity (%) | White | 43687 (93.3) |  | 169 (96.6) | 43856 (93.3) |  | 78 (100) | 43765 (93.3) |  | 53 (100) | 43740 (93.3) |
|  | Mixed | 321 (0.7) |  | 2 (1.1) | 323 (0.7) |  | 0 (0) | 321 (0.7) |  | 0 (0) | 321 (0.7) |
|  | Asian | 1083 (2.3) |  | 3 (1.7) | 1086 (2.3) |  | 0 (0) | 1083 (2.3) |  | 0 (0) | 1083 (2.3) |
|  | Black | 1154 (2.5) |  | 1 (0.6) | 1155 (2.5) |  | 0 (0) | 1154 (2.5) |  | 0 (0) | 1154 (2.5) |
|  | Other | 579 (1.2) |  | 0 (0) | 579 (1.2) |  | 0 (0) | 579 (1.2) |  | 0 (0) | 579 (1.2) |
| Alcohol intake frequency (%) | Daily or almost daily | 9411 (20.1) |  | 40 (22.9) | 9451 (20.1) |  | 23 (29.5) | 9434 (20.1) |  | 12 (22.6) | 9423 (20.1) |
|  | Three or four times a week | 10693 (22.8) |  | 39 (22.3) | 10732 (22.8) |  | 14 (17.9) | 10707 (22.8) |  | 8 (15.1) | 10701 (22.8) |
|  | Once or twice a week | 12130 (25.9) |  | 48 (27.4) | 12178 (25.9) |  | 13 (16.7) | 12143 (25.9) |  | 11 (20.8) | 12141 (25.9) |
|  | One to three times a month | 5105 (10.9) |  | 13 (7.4) | 5118 (10.9) |  | 6 (7.7) | 5111 (10.9) |  | 8 (15.1) | 5113 (10.9) |
|  | Special occasions only | 5440 (11.6) |  | 23 (13.1) | 5463 (11.6) |  | 9 (11.5) | 5449 (11.6) |  | 8 (15.1) | 5448 (11.6) |
|  | Never | 4045 (8.6) |  | 12 (6.9) | 4057 (8.6) |  | 13 (16.7) | 4058 (8.7) |  | 6 (11.3) | 4051 (8.6) |
| Smoking status (%) | Never | 25914 (55.3) |  | 31 (17.7) | 25945 (55.2) |  | 4 (5.1) | 25918 (55.3) |  | 2 (3.8) | 25916 (55.3) |
|  | Previous | 16122 (34.4) |  | 75 (42.9) | 16197 (34.5) |  | 28 (35.9) | 16150 (34.4) |  | 19 (35.8) | 16141 (34.4) |
|  | Current | 4788 (10.2) |  | 69 (39.4) | 4857 (10.3) |  | 46 (59) | 4834 (10.3) |  | 32 (60.4) | 4820 (10.3) |
| Family history of cancer (%) | No | 30211 (64.5) |  | 86 (49.1) | 30297 (64.5) |  | 46 (59) | 30257 (64.5) |  | 35 (66) | 30246 (64.5) |
|  | Yes | 16613 (35.5) |  | 89 (50.9) | 16702 (35.5) |  | 32 (41) | 16645 (35.5) |  | 18 (34) | 16631 (35.5) |
| Education background (%) | College or University degree | 15634 (33.4) |  | 31 (17.7) | 15665 (33.3) |  | 10 (12.8) | 15644 (33.4) |  | 7 (13.2) | 15641 (33.4) |
|  | A levels/AS levels or equivalent | 5235 (11.2) |  | 12 (6.9) | 5247 (11.2) |  | 4 (5.1) | 5239 (11.2) |  | 5 (9.4) | 5240 (11.2) |
|  | O levels/GCSEs or equivalent | 9784 (20.9) |  | 43 (24.6) | 9827 (20.9) |  | 10 (12.8) | 9794 (20.9) |  | 9 (17) | 9793 (20.9) |
|  | CSEs or equivalent | 2584 (5.5) |  | 6 (3.4) | 2590 (5.5) |  | 1 (1.3) | 2585 (5.5) |  | 3 (5.7) | 2587 (5.5) |
|  | NVQ or HND or HNC or equivalent | 3108 (6.6) |  | 7 (4) | 3115 (6.6) |  | 10 (12.8) | 3118 (6.6) |  | 9 (17) | 3117 (6.6) |
|  | Other professional qualifications | 2431 (5.2) |  | 12 (6.9) | 2443 (5.2) |  | 3 (3.8) | 2434 (5.2) |  | 2 (3.8) | 2433 (5.2) |
|  | None of the above | 8048 (17.2) |  | 64 (36.6) | 8112 (17.3) |  | 40 (51.3) | 8088 (17.2) |  | 18 (34) | 8066 (17.2) |
| Townsend deprivation index (%) | (-6.26~-3.93) | 9291 (19.8) |  | 23 (13.1) | 9314 (19.8) |  | 10 (12.8) | 9301 (19.8) |  | 4 (7.5) | 9295 (19.8) |
|  | (-3.93~-2.76) | 9219 (19.7) |  | 24 (13.7) | 9243 (19.7) |  | 8 (10.3) | 9227 (19.7) |  | 7 (13.2) | 9226 (19.7) |
|  | (-2.76~-1.30) | 8843 (18.9) |  | 33 (18.9) | 8876 (18.9) |  | 15 (19.2) | 8858 (18.9) |  | 11 (20.8) | 8854 (18.9) |
|  | (-1.29~1.348) | 9537 (20.4) |  | 35 (20) | 9572 (20.4) |  | 14 (17.9) | 9551 (20.4) |  | 11 (20.8) | 9548 (20.4) |
|  | (1.349~11.00) | 9934 (21.2) |  | 60 (34.3) | 9994 (21.3) |  | 31 (39.7) | 9965 (21.2) |  | 20 (37.7) | 9954 (21.2) |
| BMI (%) | 18.5~24.9 | 14857 (31.7) |  | 68 (38.9) | 14925 (31.8) |  | 22 (28.2) | 14879 (31.7) |  | 15 (28.3) | 14872 (31.7) |
|  | <18.5 | 234 (0.5) |  | 3 (1.7) | 237 (0.5) |  | 0 (0) | 234 (0.5) |  | 1 (1.9) | 235 (0.5) |
|  | 25~30 | 20243 (43.2) |  | 67 (38.3) | 20310 (43.2) |  | 27 (34.6) | 20270 (43.2) |  | 25 (47.2) | 20268 (43.2) |
|  | ≥30 | 11490 (24.5) |  | 37 (21.1) | 11527 (24.5) |  | 29 (37.2) | 11519 (24.6) |  | 12 (22.6) | 11502 (24.5) |
| Physical activity (%) | Low | 7306 (15.6) |  | 33 (18.9) | 7339 (15.6) |  | 19 (24.4) | 7325 (15.6) |  | 9 (17) | 7315 (15.6) |
|  | Moderate | 24845 (53.1) |  | 91 (52) | 24936 (53.1) |  | 37 (47.4) | 24882 (53.1) |  | 27 (50.9) | 24872 (53.1) |
|  | High | 14673 (31.3) |  | 51 (29.1) | 14724 (31.3) |  | 22 (28.2) | 14695 (31.3) |  | 17 (32.1) | 14690 (31.3) |

| **Supplementary table 4. Univariate logistic results for lung adenocarcinoma, squamous cell carcinoma and small cell carcinoma.** | | | | | | | | | | |
| --- | --- | --- | --- | --- | --- | --- | --- | --- | --- | --- |
| **Protein** | **Adenocarcinoma** | |  | **Protein** | **Squamous Cell Carcinoma** | |  | **Protein** | **Small Cell Carcinoma** | |
|  | **OR (95%CI)** | ***P*** |  |  | **OR (95%CI)** | ***P*** |  |  | **OR (95%CI)** | ***P*** |
| CXCL17 | 3.48 (2.97-4.09) | 1.41E-52 |  | CXCL17 | 4.17 (3.36-5.16) | 5.96E-39 |  | CXCL17 | 3.79 (3-4.79) | 8.77E-29 |
| WFDC2 | 3.67 (3.05-4.43) | 1.82E-42 |  | WFDC2 | 4.9 (3.86-6.23) | 1.20E-38 |  | ALPP | 1.96 (1.73-2.22) | 7.87E-26 |
| CEACAM5 | 2.4 (2.08-2.77) | 9.03E-33 |  | MMP12 | 3.97 (3.17-4.98) | 6.41E-33 |  | CEACAM5 | 2.84 (2.3-3.51) | 5.03E-22 |
| LAMP3 | 3.12 (2.57-3.79) | 1.36E-30 |  | GDF15 | 2.79 (2.34-3.33) | 1.32E-29 |  | WFDC2 | 4.33 (3.2-5.86) | 2.04E-21 |
| ALPP | 1.57 (1.45-1.7) | 3.49E-30 |  | ALPP | 1.83 (1.64-2.03) | 7.19E-29 |  | LAMP3 | 5.36 (3.78-7.58) | 3.03E-21 |
| PIGR | 3.96 (3.06-5.12) | 1.21E-25 |  | PLAUR | 11.47 (7.31-18.01) | 2.90E-26 |  | PIGR | 5.98 (4.03-8.88) | 7.94E-19 |
| MMP12 | 2.6 (2.17-3.11) | 2.23E-25 |  | MSLN | 3.26 (2.62-4.08) | 1.32E-25 |  | MMP12 | 3.39 (2.54-4.54) | 2.01E-16 |
| GDF15 | 2.18 (1.87-2.54) | 2.01E-23 |  | PIGR | 5.92 (4.22-8.29) | 4.89E-25 |  | MSLN | 3.1 (2.36-4.07) | 3.08E-16 |
| PLAUR | 5.75 (4.06-8.14) | 6.20E-23 |  | CEACAM5 | 2.71 (2.24-3.28) | 2.08E-24 |  | GDF15 | 2.49 (2-3.12) | 8.31E-16 |
| MSLN | 2.24 (1.9-2.65) | 1.54E-21 |  | LAMP3 | 4.39 (3.3-5.84) | 3.56E-24 |  | PLAUR | 9.22 (5.23-16.25) | 1.56E-14 |
| SFTPA2 | 2.28 (1.93-2.71) | 2.73E-21 |  | CDCP1 | 3.21 (2.53-4.08) | 9.36E-22 |  | SUSD2 | 3.93 (2.77-5.57) | 1.85E-14 |
| SCGB3A1 | 8.74 (5.38-14.21) | 2.14E-18 |  | FUT3_FUT5 | 4.6 (3.35-6.34) | 7.33E-21 |  | SFTPA2 | 2.7 (2.09-3.49) | 3.34E-14 |
| CEACAM6 | 2.8 (2.21-3.55) | 1.25E-17 |  | ACVRL1 | 8.56 (5.41-13.56) | 4.91E-20 |  | SPINT1 | 4.64 (3.11-6.93) | 6.15E-14 |
| EDIL3 | 2.66 (2.09-3.37) | 8.35E-16 |  | SFTPA2 | 2.74 (2.21-3.4) | 4.70E-20 |  | PRSS8 | 4.85 (3.21-7.34) | 7.56E-14 |
| CDCP1 | 2.14 (1.77-2.57) | 1.46E-15 |  | EDA2R | 3.67 (2.71-4.96) | 2.93E-17 |  | HEPH | 22.2 (9.83-50.16) | 8.94E-14 |
| TREM2 | 2.3 (1.87-2.82) | 3.37E-15 |  | PRSS8 | 4.47 (3.16-6.32) | 2.89E-17 |  | TFF2 | 2.71 (2.07-3.54) | 2.53E-13 |
| ACVRL1 | 4.49 (3.07-6.58) | 1.02E-14 |  | IFI30 | 4.12 (2.94-5.77) | 2.15E-16 |  | CCL22 | 2.64 (2.02-3.44) | 7.67E-13 |
| TFF2 | 1.97 (1.66-2.35) | 1.54E-14 |  | CD83 | 4.9 (3.31-7.23) | 1.56E-15 |  | TREM2 | 3.39 (2.42-4.76) | 1.40E-12 |
| TNR | 0.33 (0.25-0.44) | 2.85E-14 |  | IGFBP4 | 2.82 (2.19-3.64) | 1.54E-15 |  | SFTPD | 3.43 (2.44-4.84) | 2.03E-12 |
| SUSD2 | 2.91 (2.21-3.84) | 2.99E-14 |  | SUSD2 | 3.67 (2.66-5.05) | 1.80E-15 |  | SCGB3A1 | 16.14 (7.42-35.11) | 2.32E-12 |
| IGFBP4 | 2.2 (1.79-2.7) | 5.52E-14 |  | EDIL3 | 3.21 (2.4-4.3) | 3.25E-15 |  | CEACAM6 | 3.37 (2.37-4.79) | 1.34E-11 |
| SFTPD | 2.07 (1.71-2.51) | 1.19E-13 |  | NPC2 | 3.43 (2.52-4.66) | 4.06E-15 |  | NPC2 | 3.38 (2.36-4.85) | 3.80E-11 |
| CD83 | 3.1 (2.28-4.22) | 5.84E-13 |  | CEACAM6 | 3.46 (2.54-4.72) | 4.39E-15 |  | LTA4H | 2.59 (1.95-3.44) | 4.09E-11 |
| HAVCR1 | 1.77 (1.51-2.07) | 6.57E-13 |  | CLEC5A | 5.85 (3.75-9.11) | 6.41E-15 |  | ICAM1 | 5.54 (3.31-9.28) | 7.36E-11 |
| PRSS8 | 2.51 (1.95-3.24) | 1.55E-12 |  | TNFRSF10B | 1.7 (1.48-1.94) | 1.19E-14 |  | TNFRSF10A | 2.92 (2.1-4.06) | 1.71E-10 |
| RSPO1 | 2.81 (2.09-3.77) | 5.37E-12 |  | TNFRSF6B | 2.32 (1.87-2.88) | 1.30E-14 |  | SERPINA3 | 9.47 (4.73-18.98) | 2.26E-10 |
| MMP9 | 2.07 (1.67-2.55) | 1.54E-11 |  | KRT19 | 2.27 (1.84-2.8) | 3.07E-14 |  | TNFSF13B | 4.68 (2.89-7.57) | 3.22E-10 |
| ELN | 2.75 (2.02-3.74) | 1.28E-10 |  | TFF2 | 2.44 (1.93-3.08) | 7.40E-14 |  | DPP10 | 3.27 (2.25-4.77) | 6.73E-10 |
| SCGB3A2 | 1.41 (1.27-1.57) | 1.68E-10 |  | ICAM5 | 3.89 (2.71-5.57) | 1.27E-13 |  | ICAM5 | 3.8 (2.48-5.81) | 7.08E-10 |
| IFI30 | 2.56 (1.92-3.41) | 1.79E-10 |  | LTA4H | 2.46 (1.94-3.13) | 1.31E-13 |  | CDCP1 | 2.64 (1.93-3.59) | 8.74E-10 |
| TNFRSF10B | 1.51 (1.33-1.72) | 2.08E-10 |  | TREM2 | 2.97 (2.22-3.97) | 1.71E-13 |  | ANGPT2 | 4.02 (2.57-6.29) | 1.21E-09 |
| IGDCC4 | 0.27 (0.18-0.4) | 3.36E-10 |  | VSIG2 | 2.41 (1.91-3.05) | 2.05E-13 |  | CD83 | 4.37 (2.71-7.06) | 1.59E-09 |
| BCAN | 0.33 (0.24-0.47) | 4.85E-10 |  | SPINT1 | 4.1 (2.81-5.99) | 2.46E-13 |  | SCGB3A2 | 1.61 (1.38-1.88) | 1.65E-09 |
| IL22 | 1.65 (1.41-1.94) | 5.16E-10 |  | CLEC4D | 2.64 (2.03-3.43) | 5.79E-13 |  | FASLG | 0.23 (0.14-0.37) | 1.86E-09 |
| CLEC5A | 3.18 (2.21-4.59) | 5.55E-10 |  | RAB6A | 0.26 (0.18-0.38) | 6.47E-13 |  | LECT2 | 2.07 (1.63-2.63) | 3.25E-09 |
| SLITRK2 | 0.42 (0.31-0.55) | 1.18E-09 |  | EFNA4 | 4.39 (2.93-6.59) | 7.64E-13 |  | EDIL3 | 2.96 (2.06-4.25) | 4.18E-09 |
| OSM | 1.71 (1.44-2.04) | 1.26E-09 |  | HGF | 3.53 (2.5-4.99) | 7.88E-13 |  | TNFRSF11B | 5.16 (2.98-8.92) | 4.26E-09 |
| EDA2R | 2.16 (1.68-2.77) | 1.68E-09 |  | TNR | 0.24 (0.16-0.35) | 1.24E-12 |  | LRG1 | 7.25 (3.73-14.11) | 5.33E-09 |
| TNFSF13 | 3.74 (2.43-5.74) | 1.82E-09 |  | EDN1 | 6.23 (3.76-10.33) | 1.29E-12 |  | OCLN | 3.02 (2.08-4.4) | 6.78E-09 |
| TFF1 | 1.5 (1.31-1.71) | 1.96E-09 |  | HEPH | 13.41 (6.43-27.98) | 4.44E-12 |  | SPON1 | 3.36 (2.23-5.07) | 6.61E-09 |
| FUT3_FUT5 | 2.19 (1.7-2.84) | 2.34E-09 |  | ANGPT2 | 3.72 (2.54-5.45) | 1.32E-11 |  | TNR | 0.24 (0.15-0.39) | 7.44E-09 |
| CAPG | 1.76 (1.46-2.12) | 2.50E-09 |  | ADM | 8.2 (4.45-15.1) | 1.41E-11 |  | NHLRC3 | 9.8 (4.48-21.45) | 1.13E-08 |
| RSPO3 | 2.97 (2.07-4.27) | 3.31E-09 |  | PIK3IP1 | 4.59 (2.95-7.14) | 1.42E-11 |  | FAM20A | 4.85 (2.82-8.36) | 1.29E-08 |
| ITIH3 | 2.74 (1.96-3.84) | 4.11E-09 |  | TNFRSF1A | 4.32 (2.82-6.61) | 1.55E-11 |  | IGDCC4 | 0.2 (0.12-0.35) | 1.29E-08 |
| IL6 | 1.41 (1.26-1.58) | 4.34E-09 |  | OCLN | 2.98 (2.17-4.09) | 1.69E-11 |  | TNFRSF10B | 1.65 (1.39-1.96) | 1.25E-08 |
| ICAM5 | 2.24 (1.7-2.94) | 9.33E-09 |  | CD302 | 4.2 (2.76-6.38) | 2.10E-11 |  | CA6 | 0.38 (0.27-0.53) | 1.49E-08 |
| MSMB | 1.66 (1.39-1.97) | 9.97E-09 |  | SCARB2 | 3.59 (2.46-5.25) | 3.99E-11 |  | IFI30 | 3.46 (2.22-5.37) | 3.57E-08 |
| PTPRB | 3.38 (2.23-5.14) | 1.12E-08 |  | TNFRSF9 | 2.64 (1.97-3.53) | 5.58E-11 |  | HAVCR1 | 2.04 (1.58-2.64) | 4.14E-08 |
| TGFA | 1.95 (1.55-2.46) | 1.41E-08 |  | TNFRSF4 | 3.37 (2.34-4.86) | 6.71E-11 |  | SIGLEC7 | 5.22 (2.88-9.46) | 4.94E-08 |
| CD302 | 2.64 (1.89-3.7) | 1.67E-08 |  | VWC2 | 4.11 (2.68-6.3) | 8.26E-11 |  | MSMB | 2.21 (1.66-2.94) | 5.42E-08 |
| OLR1 | 1.61 (1.36-1.91) | 2.07E-08 |  | CHCHD10 | 2.68 (1.99-3.6) | 8.48E-11 |  | CD80 | 4.51 (2.61-7.81) | 7.20E-08 |
| TNFRSF10A | 2.16 (1.65-2.83) | 2.33E-08 |  | IGFBP3 | 0.23 (0.15-0.36) | 8.78E-11 |  | PTPRB | 5.13 (2.81-9.35) | 9.63E-08 |
| COLEC12 | 3.09 (2.07-4.59) | 2.77E-08 |  | TNFSF13B | 4.16 (2.7-6.41) | 9.94E-11 |  | FGL1 | 2.57 (1.81-3.65) | 1.41E-07 |
| CKB | 1.88 (1.5-2.35) | 3.00E-08 |  | CAPG | 2.23 (1.75-2.85) | 1.14E-10 |  | BPIFB1 | 2.6 (1.82-3.72) | 1.53E-07 |
| CCDC80 | 2.26 (1.69-3.03) | 3.90E-08 |  | CST3 | 4.51 (2.84-7.15) | 1.50E-10 |  | HGF | 3.18 (2.06-4.91) | 1.94E-07 |
| SHISA5 | 2.54 (1.82-3.53) | 3.94E-08 |  | REN | 1.75 (1.48-2.08) | 1.53E-10 |  | RNASE6 | 2.44 (1.74-3.42) | 2.12E-07 |
| LRG1 | 3.77 (2.35-6.04) | 3.99E-08 |  | LCN2 | 2.87 (2.08-3.96) | 1.64E-10 |  | GRP | 2.5 (1.77-3.54) | 2.36E-07 |
| PGF | 2.27 (1.69-3.04) | 4.18E-08 |  | BSG | 10.45 (5.08-21.52) | 1.91E-10 |  | C9 | 3.68 (2.24-6.05) | 2.68E-07 |
| CEACAM8 | 1.81 (1.46-2.25) | 5.97E-08 |  | SFTPD | 2.53 (1.9-3.37) | 1.88E-10 |  | IGFBP4 | 2.44 (1.73-3.43) | 3.37E-07 |
| CD27 | 2.13 (1.62-2.8) | 7.37E-08 |  | CHIT1 | 1.96 (1.59-2.41) | 2.45E-10 |  | HEG1 | 7.87 (3.56-17.42) | 3.60E-07 |
| RNASE6 | 2.03 (1.57-2.62) | 7.55E-08 |  | MZB1 | 2.65 (1.95-3.59) | 3.41E-10 |  | EDA2R | 2.83 (1.88-4.25) | 5.96E-07 |
| HGF | 2.21 (1.66-2.96) | 7.65E-08 |  | SMPD1 | 2.71 (1.98-3.71) | 4.49E-10 |  | CCL17 | 1.82 (1.43-2.3) | 8.56E-07 |
| AGRN | 2.28 (1.69-3.09) | 8.19E-08 |  | KLK4 | 1.96 (1.58-2.42) | 5.57E-10 |  | SCGB1A1 | 0.45 (0.33-0.62) | 1.25E-06 |
| ADM | 3.1 (2.05-4.7) | 8.68E-08 |  | ADGRG1 | 1.65 (1.41-1.94) | 6.87E-10 |  | TNFRSF6B | 2.04 (1.53-2.71) | 1.33E-06 |
| RGMA | 0.32 (0.21-0.49) | 9.77E-08 |  | HAVCR2 | 3.61 (2.4-5.43) | 7.41E-10 |  | FLT3LG | 4.57 (2.46-8.46) | 1.39E-06 |
| ALDH3A1 | 1.45 (1.26-1.66) | 1.05E-07 |  | RELT | 4.3 (2.7-6.86) | 8.61E-10 |  | PLAU | 2.36 (1.66-3.34) | 1.42E-06 |
| ACHE | 0.28 (0.17-0.45) | 1.42E-07 |  | AREG | 2.45 (1.84-3.26) | 9.84E-10 |  | CPXM2 | 3.79 (2.2-6.52) | 1.51E-06 |
| TNFSF13B | 2.62 (1.83-3.76) | 1.48E-07 |  | RNASE6 | 2.43 (1.82-3.23) | 1.43E-09 |  | ORM1 | 9.35 (3.74-23.39) | 1.78E-06 |
| APLP1 | 0.5 (0.39-0.65) | 1.72E-07 |  | RSPO3 | 4.14 (2.61-6.57) | 1.69E-09 |  | INHBB | 2.63 (1.76-3.92) | 2.11E-06 |
| CCL17 | 1.44 (1.25-1.66) | 2.59E-07 |  | COL18A1 | 8.42 (4.21-16.87) | 1.79E-09 |  | CTSD | 2.32 (1.64-3.28) | 2.21E-06 |
| NPC2 | 2.26 (1.66-3.09) | 2.71E-07 |  | PON1 | 0.22 (0.14-0.36) | 2.00E-09 |  | TFF1 | 1.71 (1.37-2.13) | 2.39E-06 |
| RNASE1 | 2.57 (1.79-3.68) | 2.90E-07 |  | TGFA | 2.56 (1.88-3.49) | 2.43E-09 |  | LTBP2 | 3.58 (2.1-6.11) | 2.79E-06 |
| TNFRSF6B | 1.62 (1.35-1.96) | 4.29E-07 |  | ICAM1 | 4.32 (2.66-7) | 3.00E-09 |  | ADAMTS16 | 1.97 (1.48-2.61) | 3.10E-06 |
| TFF3 | 1.58 (1.32-1.89) | 5.10E-07 |  | IL22 | 1.86 (1.51-2.28) | 3.38E-09 |  | REG3A | 1.96 (1.48-2.61) | 3.51E-06 |
| EDN1 | 3.07 (1.98-4.76) | 5.35E-07 |  | BCAN | 0.22 (0.14-0.37) | 4.12E-09 |  | FGA | 4.92 (2.51-9.67) | 3.68E-06 |
| VSIG2 | 1.66 (1.36-2.03) | 6.14E-07 |  | IL19 | 1.82 (1.49-2.23) | 4.18E-09 |  | KITLG | 0.29 (0.17-0.49) | 3.87E-06 |
| FASLG | 0.45 (0.33-0.61) | 6.14E-07 |  | CCL11 | 4.09 (2.55-6.55) | 4.65E-09 |  | GKN1 | 3.63 (2.09-6.3) | 4.79E-06 |
| ANGPT2 | 2.12 (1.58-2.85) | 6.80E-07 |  | CCL21 | 2.05 (1.61-2.61) | 4.71E-09 |  | IL6 | 1.52 (1.27-1.82) | 4.97E-06 |
| OCLN | 1.99 (1.51-2.61) | 7.59E-07 |  | RNASE1 | 3.34 (2.23-5) | 4.90E-09 |  | HLA.DRA | 3.31 (1.98-5.54) | 5.47E-06 |
| CD80 | 2.56 (1.76-3.72) | 7.68E-07 |  | RSPO1 | 3.13 (2.13-4.59) | 5.59E-09 |  | SMAD5 | 8.05 (3.27-19.8) | 5.64E-06 |
| CCL21 | 1.7 (1.38-2.1) | 8.14E-07 |  | NECTIN4 | 3.28 (2.2-4.89) | 5.84E-09 |  | CCL18 | 1.57 (1.29-1.9) | 7.13E-06 |
| LCN2 | 1.98 (1.5-2.6) | 1.06E-06 |  | MMP7 | 3.13 (2.13-4.6) | 7.06E-09 |  | GCHFR | 1.98 (1.47-2.68) | 7.42E-06 |
| CLEC10A | 2.45 (1.71-3.52) | 1.19E-06 |  | ALDH3A1 | 1.65 (1.39-1.96) | 7.67E-09 |  | IL2RA | 2.82 (1.79-4.43) | 7.44E-06 |
| CCL28 | 1.61 (1.33-1.94) | 1.19E-06 |  | TFF1 | 1.71 (1.43-2.06) | 7.81E-09 |  | ALDH3A1 | 1.62 (1.31-2) | 8.78E-06 |
| NTproBNP | 1.3 (1.17-1.44) | 1.20E-06 |  | HAVCR1 | 1.91 (1.53-2.38) | 8.95E-09 |  | SPINK1 | 2.43 (1.64-3.61) | 9.93E-06 |
| SPINK1 | 1.87 (1.45-2.4) | 1.23E-06 |  | CD80 | 4.02 (2.49-6.47) | 1.06E-08 |  | CRELD1 | 4.19 (2.22-7.92) | 1.03E-05 |
| COL18A1 | 3.49 (2.1-5.78) | 1.25E-06 |  | FCAR | 2.57 (1.85-3.55) | 1.28E-08 |  | BCAN | 0.25 (0.14-0.47) | 1.17E-05 |
| LECT2 | 1.55 (1.3-1.85) | 1.34E-06 |  | MSMB | 2.03 (1.59-2.6) | 1.27E-08 |  | MANSC4 | 1.97 (1.45-2.68) | 1.30E-05 |
| CST3 | 2.48 (1.71-3.58) | 1.40E-06 |  | PGF | 2.69 (1.91-3.79) | 1.28E-08 |  | SERPINA1 | 20.71 (5.28-81.18) | 1.37E-05 |
| REN | 1.4 (1.22-1.6) | 1.41E-06 |  | WNT9A | 3.27 (2.17-4.93) | 1.35E-08 |  | IGSF21 | 0.18 (0.08-0.39) | 1.48E-05 |
| IGFBP2 | 1.62 (1.33-1.96) | 1.45E-06 |  | CLEC6A | 2.54 (1.84-3.51) | 1.39E-08 |  | MSR1 | 2.65 (1.7-4.11) | 1.47E-05 |
| VWC2 | 2.15 (1.58-2.94) | 1.46E-06 |  | IL18BP | 3.79 (2.39-6) | 1.41E-08 |  | ROBO2 | 5.98 (2.64-13.52) | 1.77E-05 |
| TNFRSF9 | 1.87 (1.45-2.41) | 1.50E-06 |  | PGA4 | 2.16 (1.66-2.82) | 1.48E-08 |  | CD101 | 2.44 (1.62-3.69) | 2.15E-05 |
| MMP8 | 1.46 (1.25-1.71) | 1.64E-06 |  | RBFOX3 | 2.35 (1.75-3.16) | 1.50E-08 |  | CFI | 11.4 (3.71-35.08) | 2.20E-05 |
| FGL1 | 1.69 (1.36-2.1) | 2.04E-06 |  | LAIR1 | 2.3 (1.72-3.06) | 1.64E-08 |  | KRT19 | 1.84 (1.38-2.45) | 2.86E-05 |
| CCL7 | 1.47 (1.25-1.73) | 2.10E-06 |  | KLK13 | 2.82 (1.97-4.04) | 1.73E-08 |  | TGFA | 2.3 (1.56-3.4) | 2.88E-05 |
| CCL27 | 1.66 (1.35-2.05) | 2.10E-06 |  | LAYN | 3.21 (2.14-4.83) | 1.84E-08 |  | AOC3 | 4.31 (2.17-8.54) | 2.93E-05 |
| CRIP2 | 1.83 (1.43-2.35) | 2.18E-06 |  | SHISA5 | 3.04 (2.06-4.48) | 1.97E-08 |  | VNN2 | 3.63 (1.98-6.65) | 3.07E-05 |
| CLEC4D | 1.61 (1.32-1.96) | 2.26E-06 |  | BPIFB1 | 2.32 (1.73-3.12) | 2.12E-08 |  | MMP9 | 2.25 (1.54-3.3) | 3.11E-05 |
| HEG1 | 3.39 (2.04-5.64) | 2.66E-06 |  | CEACAM8 | 2.23 (1.68-2.95) | 2.27E-08 |  | QSOX1 | 1.71 (1.33-2.2) | 3.15E-05 |
| OMG | 0.63 (0.52-0.77) | 3.03E-06 |  | DSC2 | 3.3 (2.17-5.02) | 2.29E-08 |  | CD302 | 3.29 (1.87-5.76) | 3.29E-05 |
| REG4 | 1.75 (1.38-2.22) | 3.19E-06 |  | TNFRSF10A | 2.55 (1.83-3.53) | 2.26E-08 |  | OSMR | 6.56 (2.7-15.94) | 3.29E-05 |
| ASGR1 | 2.37 (1.65-3.41) | 3.38E-06 |  | CD27 | 2.87 (1.98-4.17) | 2.85E-08 |  | PGLYRP1 | 2.37 (1.58-3.56) | 3.33E-05 |
| SFTPA1 | 1.45 (1.24-1.69) | 3.54E-06 |  | REG4 | 2.47 (1.79-3.41) | 3.59E-08 |  | ACVRL1 | 4.3 (2.16-8.57) | 3.44E-05 |
| RNF149 | 2.46 (1.68-3.6) | 3.61E-06 |  | TNFRSF1B | 1.91 (1.52-2.4) | 3.61E-08 |  | OSM | 1.92 (1.41-2.62) | 3.46E-05 |
| BPIFB1 | 1.59 (1.31-1.94) | 3.81E-06 |  | PILRA | 3.33 (2.17-5.12) | 3.68E-08 |  | RARRES2 | 1.97 (1.43-2.73) | 3.60E-05 |
| TIMP1 | 2.42 (1.66-3.53) | 3.83E-06 |  | CD74 | 3.52 (2.25-5.51) | 3.79E-08 |  | VSIG2 | 1.97 (1.43-2.73) | 3.92E-05 |
| FCN1 | 2.05 (1.51-2.78) | 3.88E-06 |  | IL6 | 1.52 (1.31-1.76) | 4.49E-08 |  | TNFRSF12A | 2.8 (1.71-4.57) | 4.12E-05 |
| IL7R | 0.57 (0.45-0.73) | 3.97E-06 |  | PTPRB | 4.53 (2.64-7.77) | 4.49E-08 |  | GSN | 0.13 (0.05-0.34) | 4.41E-05 |
| EFNA4 | 2.31 (1.62-3.3) | 4.23E-06 |  | TPP1 | 2.17 (1.64-2.86) | 4.42E-08 |  | SERPIND1 | 5.67 (2.45-13.11) | 4.98E-05 |
| BLMH | 0.46 (0.33-0.64) | 4.50E-06 |  | GALNT5 | 4.11 (2.47-6.83) | 5.13E-08 |  | CFB | 4.87 (2.26-10.49) | 5.17E-05 |
| EGFR | 0.17 (0.08-0.36) | 4.54E-06 |  | CALCA | 1.74 (1.42-2.12) | 5.23E-08 |  | GALNT5 | 3.74 (1.98-7.09) | 5.16E-05 |
| CHI3L1 | 1.39 (1.21-1.6) | 4.62E-06 |  | TNFSF13 | 5.33 (2.91-9.75) | 5.52E-08 |  | IL7R | 0.43 (0.28-0.64) | 5.37E-05 |
| CHRDL1 | 2.31 (1.61-3.31) | 5.09E-06 |  | PTGDS | 3.19 (2.09-4.86) | 6.55E-08 |  | EDN1 | 4.22 (2.1-8.48) | 5.44E-05 |
| TNFRSF1A | 2.28 (1.6-3.24) | 5.11E-06 |  | LRIG1 | 3.58 (2.25-5.69) | 7.33E-08 |  | ITGA5 | 5.06 (2.3-11.15) | 5.72E-05 |
| LTA4H | 1.56 (1.29-1.89) | 5.54E-06 |  | LIPF | 2.08 (1.59-2.72) | 7.62E-08 |  | REG4 | 2.27 (1.52-3.38) | 5.72E-05 |
| CCL22 | 1.65 (1.33-2.04) | 5.69E-06 |  | GCHFR | 1.98 (1.54-2.54) | 7.78E-08 |  | C1RL | 12.88 (3.69-44.91) | 6.08E-05 |
| ICAM1 | 2.47 (1.67-3.65) | 5.70E-06 |  | CHI3L1 | 1.67 (1.39-2.02) | 9.00E-08 |  | GAL | 0.47 (0.33-0.69) | 7.24E-05 |
| LAYN | 2.09 (1.51-2.87) | 7.02E-06 |  | CA9 | 2.32 (1.7-3.16) | 1.04E-07 |  | CA14 | 0.29 (0.16-0.54) | 7.51E-05 |
| GCHFR | 1.63 (1.32-2.02) | 7.44E-06 |  | OSM | 1.99 (1.54-2.57) | 1.10E-07 |  | NPTX1 | 2.71 (1.66-4.45) | 7.51E-05 |
| CA6 | 0.64 (0.53-0.78) | 7.50E-06 |  | COL6A3 | 2.87 (1.94-4.25) | 1.37E-07 |  | CXCL16 | 5.49 (2.36-12.77) | 7.60E-05 |
| PGLYRP1 | 1.8 (1.39-2.33) | 7.72E-06 |  | VSIG4 | 2.6 (1.82-3.71) | 1.42E-07 |  | GAST | 1.37 (1.17-1.6) | 7.83E-05 |
| DPP6 | 0.42 (0.29-0.62) | 7.73E-06 |  | FASLG | 0.31 (0.2-0.48) | 1.44E-07 |  | AGRN | 2.72 (1.65-4.46) | 8.27E-05 |
| CFHR5 | 2.38 (1.63-3.48) | 8.01E-06 |  | CKAP4 | 3.26 (2.1-5.07) | 1.50E-07 |  | TNFSF11 | 0.46 (0.31-0.68) | 8.38E-05 |
| CR2 | 0.54 (0.42-0.71) | 8.36E-06 |  | AGRN | 2.89 (1.94-4.31) | 1.64E-07 |  | CLEC4D | 1.98 (1.41-2.79) | 9.46E-05 |
| ASGR2 | 3.22 (1.92-5.38) | 8.57E-06 |  | NPDC1 | 2.88 (1.93-4.28) | 1.93E-07 |  | FUT3_FUT5 | 2.45 (1.56-3.85) | 1.05E-04 |
| SCGB1A1 | 0.63 (0.51-0.77) | 9.06E-06 |  | CXCL9 | 1.73 (1.41-2.12) | 1.97E-07 |  | B3GNT7 | 3.96 (1.97-7.96) | 1.08E-04 |
| NCAN | 0.5 (0.37-0.68) | 9.37E-06 |  | EFEMP1 | 3.63 (2.23-5.9) | 2.17E-07 |  | BMPER | 3.33 (1.81-6.14) | 1.13E-04 |
| FOLR1 | 2.46 (1.65-3.67) | 1.00E-05 |  | EFNA1 | 3.86 (2.3-6.46) | 2.84E-07 |  | FGF21 | 1.39 (1.18-1.64) | 1.18E-04 |
| MMP1 | 1.43 (1.22-1.67) | 1.03E-05 |  | GPR15L | 2.11 (1.59-2.81) | 3.04E-07 |  | TNFRSF9 | 2.21 (1.48-3.31) | 1.19E-04 |
| CSTB | 1.83 (1.4-2.4) | 1.08E-05 |  | RNF149 | 3.32 (2.1-5.26) | 3.09E-07 |  | IL22 | 1.71 (1.3-2.24) | 1.21E-04 |
| NPL | 1.85 (1.41-2.44) | 1.11E-05 |  | HEG1 | 6.12 (3.05-12.28) | 3.44E-07 |  | ODAM | 0.36 (0.21-0.61) | 1.28E-04 |
| CHGB | 1.93 (1.44-2.6) | 1.23E-05 |  | CCDC80 | 2.8 (1.89-4.17) | 3.51E-07 |  | LIPF | 1.96 (1.39-2.76) | 1.34E-04 |
| SPON1 | 2.07 (1.49-2.87) | 1.40E-05 |  | ELN | 3.21 (2.05-5.03) | 3.74E-07 |  | JMJD1C | 1.75 (1.31-2.33) | 1.38E-04 |
| CSPG4 | 0.4 (0.27-0.61) | 1.59E-05 |  | IL2RA | 2.7 (1.84-3.96) | 3.79E-07 |  | CCL20 | 1.44 (1.19-1.73) | 1.42E-04 |
| AMBP | 3.57 (2-6.38) | 1.66E-05 |  | SPINK1 | 2.36 (1.69-3.29) | 3.95E-07 |  | AGR2 | 1.51 (1.22-1.88) | 1.50E-04 |
| LTBP2 | 2.08 (1.49-2.9) | 1.71E-05 |  | AMBP | 7.54 (3.44-16.53) | 4.49E-07 |  | HAVCR2 | 2.81 (1.64-4.82) | 1.66E-04 |
| MDK | 1.54 (1.26-1.88) | 1.77E-05 |  | PSAP | 2.81 (1.88-4.2) | 4.69E-07 |  | ELN | 2.88 (1.66-5.01) | 1.81E-04 |
| GAST | 1.23 (1.12-1.35) | 1.82E-05 |  | FAM20A | 3.89 (2.29-6.6) | 4.73E-07 |  | TIMP1 | 3.08 (1.71-5.57) | 1.89E-04 |
| INHBB | 1.76 (1.36-2.28) | 1.84E-05 |  | SPINK6 | 2.54 (1.77-3.66) | 5.04E-07 |  | TNFRSF10C | 2.79 (1.62-4.79) | 2.03E-04 |
| BGN | 0.76 (0.68-0.86) | 1.89E-05 |  | MMP9 | 2.24 (1.64-3.07) | 5.12E-07 |  | ADAMTS15 | 2.57 (1.56-4.24) | 2.24E-04 |
| EFNA1 | 2.4 (1.6-3.58) | 1.97E-05 |  | FABP1 | 1.54 (1.3-1.83) | 6.04E-07 |  | FOLR1 | 3.51 (1.8-6.85) | 2.23E-04 |
| SFRP1 | 1.68 (1.32-2.14) | 2.22E-05 |  | DLL1 | 3.89 (2.28-6.64) | 6.13E-07 |  | FSTL3 | 2.82 (1.62-4.89) | 2.31E-04 |
| REG3A | 1.48 (1.23-1.77) | 2.30E-05 |  | TNFRSF12A | 2.8 (1.87-4.21) | 6.82E-07 |  | PTPRZ1 | 3.37 (1.77-6.43) | 2.30E-04 |
| CTSV | 0.5 (0.37-0.69) | 2.45E-05 |  | REG3A | 1.85 (1.45-2.35) | 6.97E-07 |  | GFRA1 | 3.54 (1.8-6.97) | 2.46E-04 |
| CCL11 | 2.03 (1.46-2.81) | 2.45E-05 |  | EGFR | 0.06 (0.02-0.19) | 7.34E-07 |  | CHI3L1 | 1.56 (1.23-1.98) | 2.51E-04 |
| SIGLEC7 | 2.46 (1.62-3.75) | 2.47E-05 |  | COL24A1 | 3.03 (1.95-4.7) | 7.48E-07 |  | PSCA | 0.83 (0.75-0.92) | 2.56E-04 |
| FSTL3 | 2.04 (1.46-2.85) | 2.52E-05 |  | FSTL3 | 3.06 (1.96-4.77) | 7.73E-07 |  | PRL | 0.58 (0.43-0.78) | 2.72E-04 |
| S100A11 | 1.52 (1.25-1.85) | 2.53E-05 |  | EPHA2 | 2.81 (1.86-4.24) | 7.95E-07 |  | PGA4 | 1.89 (1.34-2.67) | 2.80E-04 |
| EGFL7 | 1.82 (1.38-2.4) | 2.66E-05 |  | LGALS9 | 3.33 (2.06-5.36) | 7.99E-07 |  | IGFBP7 | 2.34 (1.47-3.7) | 3.08E-04 |
| CDA | 0.49 (0.35-0.68) | 2.74E-05 |  | MSR1 | 2.52 (1.75-3.64) | 8.05E-07 |  | TIMP4 | 2.26 (1.45-3.52) | 3.19E-04 |
| CLEC3B | 0.23 (0.11-0.46) | 3.24E-05 |  | PRND | 2.36 (1.68-3.32) | 8.20E-07 |  | CBLIF | 1.75 (1.29-2.38) | 3.22E-04 |
| FAM20A | 2.55 (1.64-3.96) | 3.43E-05 |  | PI3 | 1.87 (1.46-2.4) | 8.58E-07 |  | IGSF3 | 2.15 (1.41-3.28) | 3.48E-04 |
| CXCL9 | 1.4 (1.19-1.64) | 3.50E-05 |  | ULBP2 | 2.83 (1.87-4.28) | 8.54E-07 |  | BHMT2 | 0.18 (0.07-0.46) | 3.66E-04 |
| CDH2 | 1.99 (1.43-2.76) | 3.65E-05 |  | SIGLEC7 | 3.98 (2.3-6.91) | 8.87E-07 |  | CST3 | 3.13 (1.67-5.85) | 3.65E-04 |
| CCN3 | 1.96 (1.42-2.69) | 3.68E-05 |  | CCL22 | 2.01 (1.52-2.66) | 1.03E-06 |  | TNFRSF1A | 2.92 (1.61-5.27) | 3.96E-04 |
| HAVCR2 | 1.99 (1.43-2.76) | 3.75E-05 |  | DMP1 | 0.53 (0.42-0.69) | 1.08E-06 |  | ADAMTSL2 | 2.9 (1.6-5.25) | 4.33E-04 |
| NEFL | 1.64 (1.3-2.08) | 3.86E-05 |  | TNFRSF14 | 3.49 (2.11-5.77) | 1.08E-06 |  | SFTPA1 | 1.56 (1.22-2) | 4.33E-04 |
| DTX3 | 2.49 (1.61-3.85) | 4.13E-05 |  | CHRDL1 | 3.55 (2.13-5.91) | 1.18E-06 |  | AREG | 2.01 (1.36-2.97) | 4.58E-04 |
| MSTN | 0.61 (0.48-0.77) | 4.14E-05 |  | COLEC12 | 4.02 (2.29-7.04) | 1.19E-06 |  | IL1RN | 1.68 (1.26-2.24) | 4.63E-04 |
| TNFRSF12A | 1.91 (1.4-2.61) | 4.37E-05 |  | FOLR1 | 3.79 (2.22-6.5) | 1.20E-06 |  | RBFOX3 | 2.07 (1.37-3.12) | 5.08E-04 |
| PLAT | 1.6 (1.28-2.01) | 4.38E-05 |  | IL7R | 0.43 (0.3-0.6) | 1.26E-06 |  | COL18A1 | 4.83 (1.98-11.77) | 5.25E-04 |
| TGFBR2 | 1.96 (1.42-2.7) | 4.40E-05 |  | PRAP1 | 3.5 (2.1-5.83) | 1.47E-06 |  | LGALS9 | 2.86 (1.58-5.19) | 5.33E-04 |
| LMOD1 | 1.82 (1.36-2.42) | 4.67E-05 |  | CD5 | 2.54 (1.73-3.71) | 1.69E-06 |  | CRHBP | 4.16 (1.85-9.34) | 5.58E-04 |
| WFIKKN1 | 0.53 (0.39-0.72) | 4.91E-05 |  | ST6GAL1 | 2.86 (1.86-4.41) | 1.82E-06 |  | RNASE1 | 2.72 (1.54-4.81) | 5.69E-04 |
| LGALS9 | 2.05 (1.45-2.9) | 4.94E-05 |  | CHGA | 1.56 (1.3-1.87) | 1.97E-06 |  | PSAP | 2.55 (1.5-4.35) | 5.83E-04 |
| ANXA10 | 1.33 (1.16-1.53) | 5.17E-05 |  | CEACAM19 | 2.47 (1.7-3.6) | 2.19E-06 |  | FABP4 | 1.75 (1.27-2.4) | 5.96E-04 |
| TPP1 | 1.6 (1.28-2.02) | 5.44E-05 |  | LILRB4 | 2.49 (1.71-3.64) | 2.23E-06 |  | ACP5 | 2.37 (1.45-3.89) | 6.22E-04 |
| SEPTIN3 | 1.4 (1.19-1.66) | 5.51E-05 |  | NTRK3 | 0.14 (0.06-0.32) | 2.48E-06 |  | EFNA4 | 2.82 (1.55-5.12) | 6.67E-04 |
| EFCAB14 | 2.02 (1.43-2.85) | 5.86E-05 |  | TNFRSF11A | 2.54 (1.72-3.73) | 2.54E-06 |  | F9 | 6.97 (2.27-21.4) | 6.86E-04 |
| MSR1 | 1.71 (1.31-2.21) | 5.89E-05 |  | CXCL16 | 5.36 (2.66-10.79) | 2.56E-06 |  | CSF1 | 3.59 (1.71-7.5) | 6.97E-04 |
| VWA1 | 1.81 (1.36-2.43) | 5.97E-05 |  | TAFA5 | 2.83 (1.83-4.38) | 2.66E-06 |  | BPIFB2 | 1.69 (1.25-2.29) | 7.07E-04 |
| IFNLR1 | 1.66 (1.29-2.12) | 6.17E-05 |  | IL15 | 2.93 (1.87-4.61) | 3.00E-06 |  | TNFRSF4 | 2.39 (1.44-3.96) | 7.15E-04 |
| SERPINA3 | 3.97 (2.02-7.82) | 6.52E-05 |  | OMG | 0.51 (0.38-0.67) | 3.05E-06 |  | HSD11B1 | 0.39 (0.22-0.67) | 7.31E-04 |
| ODAM | 0.55 (0.41-0.74) | 6.58E-05 |  | INHBB | 2.31 (1.62-3.28) | 3.10E-06 |  | SHISA5 | 2.55 (1.48-4.38) | 7.31E-04 |
| TNFRSF4 | 1.85 (1.37-2.5) | 6.84E-05 |  | NPPC | 1.99 (1.49-2.67) | 3.10E-06 |  | JCHAIN | 1.84 (1.29-2.61) | 7.38E-04 |
| ST6GAL1 | 2.1 (1.46-3.03) | 6.99E-05 |  | ODAM | 0.36 (0.23-0.55) | 3.28E-06 |  | CD14 | 2.27 (1.41-3.67) | 7.48E-04 |
| SMAD5 | 3.58 (1.91-6.71) | 7.30E-05 |  | JCHAIN | 1.95 (1.47-2.59) | 3.40E-06 |  | EFNA1 | 3.16 (1.62-6.18) | 7.51E-04 |
| PILRA | 1.85 (1.36-2.52) | 7.94E-05 |  | WFDC12 | 1.83 (1.42-2.36) | 3.49E-06 |  | COLEC12 | 3.36 (1.66-6.82) | 7.62E-04 |
| IL19 | 1.39 (1.18-1.64) | 8.01E-05 |  | SPON1 | 2.64 (1.75-3.99) | 3.86E-06 |  | MMP10 | 1.75 (1.26-2.43) | 7.68E-04 |
| CD74 | 1.95 (1.4-2.73) | 8.78E-05 |  | OMD | 0.41 (0.28-0.6) | 3.96E-06 |  | REG1A | 1.86 (1.3-2.68) | 7.93E-04 |
| PGA4 | 1.51 (1.23-1.86) | 8.81E-05 |  | HPGDS | 0.28 (0.17-0.48) | 4.02E-06 |  | IL12RB2 | 0.24 (0.1-0.55) | 8.35E-04 |
| SCARB2 | 1.98 (1.4-2.78) | 9.31E-05 |  | CA4 | 4.57 (2.38-8.77) | 4.99E-06 |  | REG1B | 1.72 (1.25-2.36) | 8.55E-04 |
| PIK3IP1 | 2.2 (1.48-3.26) | 9.47E-05 |  | BLMH | 0.39 (0.26-0.58) | 5.53E-06 |  | LGMN | 2.76 (1.52-5.03) | 8.70E-04 |
| TNFRSF19 | 1.99 (1.41-2.82) | 9.50E-05 |  | PLA2G15 | 4.23 (2.27-7.89) | 5.56E-06 |  | SPARCL1 | 0.24 (0.1-0.56) | 8.99E-04 |
| RNASE4 | 2.23 (1.49-3.35) | 1.05E-04 |  | KLK11 | 2.26 (1.59-3.22) | 6.22E-06 |  | CHGA | 1.48 (1.17-1.86) | 9.19E-04 |
| ITGAM | 0.43 (0.28-0.66) | 1.05E-04 |  | SLITRK1 | 0.24 (0.13-0.44) | 6.50E-06 |  | C1S | 4.91 (1.91-12.62) | 9.45E-04 |
| NBL1 | 2.44 (1.55-3.82) | 1.09E-04 |  | GSN | 0.14 (0.06-0.33) | 7.21E-06 |  | POMC | 0.53 (0.36-0.77) | 9.54E-04 |
| SLITRK1 | 0.42 (0.27-0.65) | 1.17E-04 |  | APOC1 | 0.32 (0.19-0.52) | 7.41E-06 |  | CHRDL1 | 2.92 (1.54-5.5) | 9.60E-04 |
| CLEC6A | 1.66 (1.28-2.14) | 1.25E-04 |  | TIMP1 | 3.09 (1.89-5.07) | 7.55E-06 |  | CD27 | 2.28 (1.4-3.71) | 9.67E-04 |
| SERPIND1 | 2.7 (1.62-4.48) | 1.26E-04 |  | BTN2A1 | 3.81 (2.12-6.87) | 8.20E-06 |  | PGF | 2.28 (1.39-3.72) | 1.01E-03 |
| MZB1 | 1.58 (1.25-1.99) | 1.39E-04 |  | RNASET2 | 3.18 (1.91-5.28) | 8.19E-06 |  | A1BG | 10.91 (2.61-45.63) | 1.06E-03 |
| LRRN1 | 0.52 (0.38-0.73) | 1.41E-04 |  | IGFBP2 | 1.97 (1.46-2.66) | 8.40E-06 |  | FLT3 | 0.24 (0.1-0.56) | 1.06E-03 |
| CCL23 | 1.78 (1.32-2.4) | 1.57E-04 |  | CKB | 2.12 (1.52-2.95) | 8.74E-06 |  | ST6GAL1 | 2.53 (1.45-4.42) | 1.06E-03 |
| B3GNT7 | 2.22 (1.47-3.35) | 1.63E-04 |  | OLR1 | 1.71 (1.35-2.16) | 8.69E-06 |  | ADGRD1 | 2.54 (1.45-4.45) | 1.08E-03 |
| CD4 | 1.76 (1.31-2.36) | 1.70E-04 |  | PLAU | 2.17 (1.54-3.06) | 8.77E-06 |  | CLEC6A | 2.05 (1.33-3.16) | 1.16E-03 |
| IL18BP | 2.04 (1.41-2.97) | 1.77E-04 |  | GNPDA2 | 0.23 (0.12-0.44) | 9.09E-06 |  | MRC1 | 3.63 (1.66-7.93) | 1.23E-03 |
| SEL1L | 1.68 (1.28-2.21) | 1.85E-04 |  | APOA1 | 0.14 (0.06-0.34) | 9.40E-06 |  | IL10RB | 2.41 (1.41-4.1) | 1.26E-03 |
| BLNK | 1.7 (1.28-2.24) | 1.99E-04 |  | TFF3 | 1.71 (1.35-2.16) | 9.40E-06 |  | SORCS2 | 2.52 (1.44-4.42) | 1.27E-03 |
| ANGPTL4 | 1.7 (1.28-2.24) | 2.09E-04 |  | NFASC | 3.65 (2.05-6.47) | 9.98E-06 |  | PRCP | 2.45 (1.42-4.23) | 1.29E-03 |
| RET | 0.54 (0.39-0.75) | 2.10E-04 |  | SPINK2 | 2.59 (1.7-3.95) | 1.06E-05 |  | OMG | 0.56 (0.4-0.8) | 1.31E-03 |
| NPDC1 | 1.89 (1.35-2.66) | 2.23E-04 |  | LGALS4 | 1.95 (1.45-2.63) | 1.10E-05 |  | PRAME | 0.17 (0.06-0.5) | 1.32E-03 |
| LTBR | 2.17 (1.44-3.27) | 2.26E-04 |  | BCAM | 3.67 (2.05-6.58) | 1.25E-05 |  | IL13RA1 | 5.25 (1.91-14.49) | 1.35E-03 |
| GHR | 0.4 (0.25-0.65) | 2.40E-04 |  | DPP10 | 2.41 (1.62-3.59) | 1.35E-05 |  | WFIKKN1 | 0.41 (0.24-0.71) | 1.35E-03 |
| SELL | 0.32 (0.18-0.59) | 2.41E-04 |  | IGDCC4 | 0.28 (0.16-0.5) | 1.48E-05 |  | PLAT | 1.93 (1.29-2.88) | 1.36E-03 |
| IL2RA | 1.75 (1.3-2.35) | 2.44E-04 |  | CLEC10A | 3.24 (1.9-5.52) | 1.56E-05 |  | RGMA | 0.29 (0.14-0.62) | 1.40E-03 |
| CEACAM16 | 0.72 (0.6-0.86) | 2.56E-04 |  | GAST | 1.33 (1.17-1.52) | 1.60E-05 |  | SH3GL3 | 0.68 (0.54-0.86) | 1.46E-03 |
| NCAM1 | 0.46 (0.3-0.7) | 2.73E-04 |  | CNTN1 | 0.29 (0.16-0.5) | 1.64E-05 |  | OLR1 | 1.62 (1.2-2.19) | 1.49E-03 |
| PRL | 0.72 (0.6-0.86) | 2.79E-04 |  | CCL7 | 1.6 (1.29-1.98) | 1.65E-05 |  | KAZALD1 | 0.52 (0.35-0.78) | 1.52E-03 |
| SPINK6 | 1.55 (1.22-1.97) | 2.87E-04 |  | CA6 | 0.53 (0.4-0.71) | 1.71E-05 |  | VWA1 | 2.29 (1.37-3.83) | 1.61E-03 |
| LIPF | 1.52 (1.21-1.91) | 2.89E-04 |  | OGN | 2.29 (1.57-3.35) | 1.72E-05 |  | ALCAM | 5.48 (1.9-15.83) | 1.67E-03 |
| HEPACAM2 | 0.51 (0.35-0.73) | 2.92E-04 |  | B2M | 1.77 (1.36-2.29) | 1.76E-05 |  | BCAT1 | 2.45 (1.4-4.29) | 1.67E-03 |
| ITGB2 | 0.42 (0.27-0.68) | 3.06E-04 |  | TGFBR2 | 2.54 (1.66-3.89) | 1.83E-05 |  | KIR2DL2 | 1.63 (1.2-2.2) | 1.67E-03 |
| CLEC14A | 2.01 (1.38-2.94) | 3.08E-04 |  | SCRG1 | 2.78 (1.74-4.45) | 1.89E-05 |  | KLK4 | 1.61 (1.2-2.17) | 1.64E-03 |
| COL4A1 | 1.76 (1.29-2.4) | 3.17E-04 |  | PRCP | 2.59 (1.67-4.02) | 1.99E-05 |  | PROCR | 0.18 (0.06-0.53) | 1.68E-03 |
| CLC | 1.36 (1.15-1.61) | 3.30E-04 |  | ANGPTL4 | 2.44 (1.62-3.68) | 2.03E-05 |  | SMAD3 | 0.38 (0.21-0.7) | 1.68E-03 |
| FST | 1.56 (1.22-1.98) | 3.53E-04 |  | VEGFA | 1.9 (1.41-2.55) | 2.06E-05 |  | CD5 | 2.19 (1.34-3.59) | 1.81E-03 |
| S100A12 | 1.36 (1.15-1.61) | 3.57E-04 |  | FGA | 3.71 (2.03-6.8) | 2.10E-05 |  | COL24A1 | 2.35 (1.37-4.03) | 1.80E-03 |
| DEFA1_DEFA1B | 1.44 (1.18-1.76) | 3.68E-04 |  | MMP8 | 1.6 (1.29-1.99) | 2.11E-05 |  | VEGFD | 2.75 (1.46-5.2) | 1.84E-03 |
| ACP5 | 1.86 (1.32-2.61) | 3.77E-04 |  | SCGB1A1 | 0.54 (0.4-0.72) | 2.31E-05 |  | GRN | 3.79 (1.64-8.76) | 1.88E-03 |
| S100G | 1.65 (1.25-2.17) | 3.87E-04 |  | TNFRSF19 | 2.69 (1.7-4.25) | 2.34E-05 |  | LGALS4 | 1.8 (1.24-2.6) | 1.91E-03 |
| TNFRSF1B | 1.53 (1.21-1.93) | 3.97E-04 |  | ASGR1 | 2.95 (1.79-4.88) | 2.37E-05 |  | PCSK9 | 2.45 (1.39-4.32) | 1.96E-03 |
| SORCS2 | 1.85 (1.31-2.6) | 4.09E-04 |  | DEFB4A_DEFB4B | 1.31 (1.16-1.48) | 2.36E-05 |  | HYAL1 | 3.57 (1.59-8.01) | 1.97E-03 |
| TNFRSF11B | 2.01 (1.36-2.97) | 4.24E-04 |  | CCN3 | 2.54 (1.65-3.93) | 2.49E-05 |  | ADGRG1 | 1.43 (1.14-1.78) | 2.01E-03 |
| DDC | 0.61 (0.46-0.8) | 4.34E-04 |  | CD300LF | 2.25 (1.54-3.29) | 2.58E-05 |  | KLK10 | 1.97 (1.28-3.02) | 2.01E-03 |
| FGF21 | 1.19 (1.08-1.32) | 4.46E-04 |  | PLAT | 2.03 (1.46-2.82) | 2.59E-05 |  | SERPING1 | 7.97 (2.12-29.91) | 2.11E-03 |
| ERI1 | 1.89 (1.32-2.69) | 4.61E-04 |  | CFHR5 | 3.24 (1.87-5.62) | 2.71E-05 |  | C1R | 2.73 (1.44-5.19) | 2.14E-03 |
| PCDH9 | 0.51 (0.35-0.74) | 4.62E-04 |  | NCAN | 0.39 (0.25-0.6) | 2.76E-05 |  | RNF149 | 2.6 (1.41-4.79) | 2.22E-03 |
| FGA | 2.23 (1.42-3.49) | 4.67E-04 |  | IL15RA | 3.1 (1.83-5.27) | 2.79E-05 |  | HJV | 1.74 (1.22-2.48) | 2.33E-03 |
| VSIG4 | 1.65 (1.25-2.18) | 4.69E-04 |  | GIPC2 | 3.18 (1.85-5.48) | 2.92E-05 |  | L1CAM | 3.32 (1.53-7.2) | 2.40E-03 |
| CD5 | 1.8 (1.3-2.51) | 4.76E-04 |  | LTBP3 | 1.64 (1.3-2.07) | 3.03E-05 |  | SLC4A1 | 0.38 (0.2-0.71) | 2.42E-03 |
| EPHA2 | 1.89 (1.32-2.71) | 4.99E-04 |  | SIT1 | 2 (1.44-2.78) | 3.21E-05 |  | THBS2 | 2.14 (1.31-3.51) | 2.42E-03 |
| OGN | 1.63 (1.24-2.15) | 5.04E-04 |  | SFRP1 | 1.95 (1.42-2.67) | 3.29E-05 |  | AHNAK | 2.49 (1.38-4.51) | 2.60E-03 |
| ADAMTSL2 | 1.96 (1.34-2.85) | 5.04E-04 |  | IGFBP7 | 2.29 (1.55-3.39) | 3.44E-05 |  | TPM3 | 0.29 (0.13-0.65) | 2.64E-03 |
| SEPTIN8 | 1.88 (1.32-2.69) | 5.05E-04 |  | SCGB3A2 | 1.4 (1.19-1.64) | 3.50E-05 |  | CSF2RA | 1.59 (1.17-2.15) | 2.65E-03 |
| PILRB | 1.55 (1.21-1.98) | 5.19E-04 |  | IGLC2 | 2.11 (1.48-3) | 3.54E-05 |  | COL6A3 | 2.22 (1.32-3.75) | 2.72E-03 |
| VWF | 1.36 (1.14-1.63) | 5.24E-04 |  | PGLYRP1 | 2.12 (1.48-3.03) | 3.68E-05 |  | SLAMF8 | 1.96 (1.26-3.04) | 2.85E-03 |
| HEPH | 2.81 (1.57-5.04) | 5.32E-04 |  | CXCL14 | 1.48 (1.23-1.77) | 3.70E-05 |  | TUBB3 | 0.22 (0.08-0.6) | 2.86E-03 |
| LAIR1 | 1.58 (1.22-2.05) | 5.49E-04 |  | CPXM2 | 3.08 (1.8-5.24) | 3.73E-05 |  | PLA2G15 | 3.28 (1.49-7.21) | 3.07E-03 |
| NPPB | 1.2 (1.08-1.33) | 5.59E-04 |  | APLP1 | 0.44 (0.3-0.65) | 3.75E-05 |  | GAD1 | 0.2 (0.07-0.58) | 3.12E-03 |
| TFPI2 | 1.45 (1.17-1.79) | 5.62E-04 |  | CD59 | 3.24 (1.85-5.68) | 3.88E-05 |  | IL18RAP | 1.2 (1.06-1.36) | 3.17E-03 |
| LILRA5 | 1.96 (1.34-2.87) | 5.64E-04 |  | CCL20 | 1.4 (1.19-1.64) | 3.92E-05 |  | FCN1 | 2.29 (1.32-3.96) | 3.24E-03 |
| RELT | 1.98 (1.34-2.91) | 5.67E-04 |  | NOS3 | 1.65 (1.3-2.09) | 3.98E-05 |  | FGFR2 | 3.68 (1.54-8.77) | 3.28E-03 |
| LAMA4 | 1.9 (1.32-2.74) | 5.88E-04 |  | CRHBP | 4.05 (2.08-7.91) | 4.05E-05 |  | CTSL | 3.54 (1.52-8.26) | 3.37E-03 |
| DCBLD2 | 1.86 (1.31-2.66) | 6.03E-04 |  | ANXA5 | 2.6 (1.64-4.1) | 4.23E-05 |  | ITGBL1 | 2.2 (1.3-3.73) | 3.36E-03 |
| LGMN | 1.83 (1.29-2.59) | 6.63E-04 |  | CSTB | 2.19 (1.5-3.2) | 4.36E-05 |  | PTN | 1.81 (1.22-2.69) | 3.34E-03 |
| COCH | 1.67 (1.24-2.25) | 7.13E-04 |  | YAP1 | 2.98 (1.76-5.02) | 4.44E-05 |  | RBP2 | 1.52 (1.15-2.02) | 3.37E-03 |
| OMD | 0.62 (0.47-0.82) | 7.20E-04 |  | CLEC3B | 0.13 (0.05-0.35) | 4.61E-05 |  | ERBB2 | 3.65 (1.53-8.7) | 3.44E-03 |
| MLN | 1.28 (1.11-1.47) | 7.21E-04 |  | FLT3LG | 3.05 (1.78-5.23) | 4.80E-05 |  | IMMT | 1.8 (1.21-2.66) | 3.55E-03 |
| MRC1 | 2.24 (1.4-3.59) | 8.10E-04 |  | CHGB | 2.4 (1.57-3.66) | 4.92E-05 |  | RCC1 | 1.6 (1.16-2.19) | 3.77E-03 |
| NELL2 | 0.45 (0.28-0.72) | 8.17E-04 |  | FABP3 | 1.86 (1.38-2.51) | 4.98E-05 |  | GBP2 | 1.57 (1.16-2.13) | 3.79E-03 |
| ADAMTS13 | 0.4 (0.24-0.69) | 8.33E-04 |  | GFRA1 | 3.24 (1.83-5.71) | 5.02E-05 |  | C8B | 3.61 (1.51-8.63) | 3.84E-03 |
| A1BG | 4.22 (1.81-9.82) | 8.53E-04 |  | TIGIT | 1.84 (1.37-2.48) | 5.03E-05 |  | CLEC5A | 2.74 (1.38-5.44) | 3.91E-03 |
| CYB5R2 | 1.51 (1.19-1.93) | 8.64E-04 |  | CD274 | 2.34 (1.55-3.53) | 5.11E-05 |  | RSPO3 | 2.67 (1.37-5.22) | 3.91E-03 |
| F9 | 3.09 (1.59-6.01) | 9.07E-04 |  | FGF21 | 1.34 (1.16-1.54) | 5.32E-05 |  | FBP1 | 1.5 (1.14-1.97) | 3.93E-03 |
| SPINT2 | 1.73 (1.25-2.4) | 9.19E-04 |  | CSF1 | 3.51 (1.91-6.47) | 5.67E-05 |  | ERBB4 | 3.99 (1.55-10.22) | 3.99E-03 |
| VNN2 | 1.69 (1.24-2.31) | 9.26E-04 |  | SIRPB1 | 2.26 (1.52-3.36) | 5.74E-05 |  | CCL11 | 2.38 (1.32-4.31) | 4.05E-03 |
| ZBTB16 | 0.7 (0.57-0.87) | 9.42E-04 |  | DPP4 | 0.27 (0.15-0.52) | 5.99E-05 |  | CFHR5 | 2.72 (1.37-5.37) | 4.09E-03 |
| CASP1 | 1.31 (1.12-1.54) | 9.56E-04 |  | IL18 | 2.04 (1.44-2.88) | 6.18E-05 |  | DDR1 | 3.19 (1.45-7.05) | 4.04E-03 |
| CXCL12 | 1.68 (1.23-2.27) | 9.57E-04 |  | CCN5 | 2.34 (1.54-3.55) | 6.33E-05 |  | IL18BP | 2.55 (1.35-4.82) | 4.06E-03 |
| CKAP4 | 1.96 (1.31-2.93) | 9.96E-04 |  | LMNB2 | 2.92 (1.73-4.95) | 6.52E-05 |  | NPR1 | 0.22 (0.08-0.62) | 4.09E-03 |
| CCN5 | 1.63 (1.22-2.18) | 1.01E-03 |  | SERPINA3 | 5.18 (2.31-11.63) | 6.56E-05 |  | CRIP2 | 1.87 (1.22-2.88) | 4.12E-03 |
| DPP10 | 1.71 (1.24-2.36) | 1.02E-03 |  | PRSS22 | 2.1 (1.46-3.02) | 6.80E-05 |  | RNASE4 | 2.6 (1.35-4.98) | 4.14E-03 |
| PTGDS | 1.84 (1.28-2.65) | 1.05E-03 |  | FGF5 | 2.42 (1.56-3.74) | 7.48E-05 |  | CCL5 | 1.39 (1.11-1.74) | 4.21E-03 |
| KLK4 | 1.35 (1.13-1.62) | 1.05E-03 |  | SERPIND1 | 4.3 (2.09-8.86) | 7.62E-05 |  | LYSMD3 | 1.32 (1.09-1.6) | 4.33E-03 |
| CXCL14 | 1.3 (1.11-1.51) | 1.05E-03 |  | MDK | 1.69 (1.3-2.2) | 7.97E-05 |  | IL12B | 0.55 (0.36-0.83) | 4.44E-03 |
| DPP4 | 0.47 (0.3-0.74) | 1.06E-03 |  | CD99 | 4.75 (2.19-10.31) | 8.07E-05 |  | MLN | 1.46 (1.12-1.88) | 4.43E-03 |
| C9 | 1.65 (1.22-2.24) | 1.09E-03 |  | APCS | 3.48 (1.87-6.48) | 8.47E-05 |  | LTBP3 | 1.55 (1.15-2.1) | 4.50E-03 |
| ULBP2 | 1.7 (1.24-2.35) | 1.10E-03 |  | CNDP1 | 0.5 (0.36-0.71) | 8.71E-05 |  | ITGAM | 0.33 (0.15-0.71) | 4.52E-03 |
| TNFRSF10C | 1.6 (1.21-2.13) | 1.15E-03 |  | MLN | 1.53 (1.24-1.9) | 8.85E-05 |  | CD3G | 1.32 (1.09-1.6) | 4.63E-03 |
| IL12B | 0.68 (0.54-0.86) | 1.18E-03 |  | SLAMF8 | 2.05 (1.43-2.94) | 8.88E-05 |  |  |  |  |
| PSAP | 1.93 (1.3-2.87) | 1.18E-03 |  | SCGB3A1 | 4.81 (2.19-10.55) | 9.00E-05 |  |  |  |  |
| IL4R | 1.73 (1.24-2.41) | 1.24E-03 |  | HSPB6 | 2.14 (1.46-3.14) | 9.27E-05 |  |  |  |  |
| CFB | 2.06 (1.33-3.19) | 1.28E-03 |  | CD300E | 2.2 (1.48-3.28) | 9.41E-05 |  |  |  |  |
| CCL26 | 1.21 (1.08-1.36) | 1.32E-03 |  | HLA.E | 2.9 (1.7-4.94) | 9.48E-05 |  |  |  |  |
| CA14 | 0.57 (0.4-0.8) | 1.35E-03 |  | CBLIF | 1.67 (1.29-2.16) | 9.65E-05 |  |  |  |  |
| IL1B | 1.38 (1.13-1.68) | 1.36E-03 |  | ACE2 | 1.74 (1.32-2.29) | 9.79E-05 |  |  |  |  |
| IL17D | 1.5 (1.17-1.93) | 1.37E-03 |  | NBL1 | 3.51 (1.86-6.62) | 1.01E-04 |  |  |  |  |
| CD22 | 0.6 (0.44-0.82) | 1.38E-03 |  | PILRB | 2.12 (1.45-3.09) | 1.06E-04 |  |  |  |  |
| DLL1 | 1.94 (1.29-2.92) | 1.41E-03 |  | PTPRN2 | 2.71 (1.64-4.49) | 1.07E-04 |  |  |  |  |
| CD274 | 1.71 (1.23-2.39) | 1.46E-03 |  | ADGRG2 | 0.38 (0.23-0.62) | 1.08E-04 |  |  |  |  |
| IGFBP1 | 1.17 (1.06-1.29) | 1.48E-03 |  | EFCAB14 | 2.29 (1.5-3.5) | 1.16E-04 |  |  |  |  |
| MANSC4 | 1.4 (1.14-1.73) | 1.50E-03 |  | LGMN | 2.65 (1.62-4.36) | 1.16E-04 |  |  |  |  |
| IGFBPL1 | 1.73 (1.23-2.43) | 1.58E-03 |  | RETN | 2.04 (1.42-2.94) | 1.19E-04 |  |  |  |  |
| PTPRH | 0.59 (0.43-0.82) | 1.59E-03 |  | EGFL7 | 2.12 (1.45-3.12) | 1.24E-04 |  |  |  |  |
| FCRL1 | 0.62 (0.46-0.83) | 1.59E-03 |  | ISLR2 | 2.53 (1.57-4.06) | 1.25E-04 |  |  |  |  |
| CELA2A | 1.5 (1.17-1.94) | 1.59E-03 |  | DPP6 | 0.34 (0.19-0.59) | 1.28E-04 |  |  |  |  |
| TNFRSF13B | 1.69 (1.22-2.35) | 1.70E-03 |  | FAM3C | 3.03 (1.71-5.37) | 1.44E-04 |  |  |  |  |
| CXCL16 | 2.29 (1.37-3.85) | 1.70E-03 |  | SIGLEC1 | 2.16 (1.45-3.21) | 1.44E-04 |  |  |  |  |
| INSL4 | 1.36 (1.12-1.64) | 1.74E-03 |  | CD101 | 2.15 (1.45-3.19) | 1.45E-04 |  |  |  |  |
| IL15 | 1.8 (1.24-2.59) | 1.74E-03 |  | RARRES2 | 1.78 (1.32-2.39) | 1.45E-04 |  |  |  |  |
| GSN | 0.31 (0.15-0.64) | 1.74E-03 |  | CLEC14A | 2.81 (1.65-4.78) | 1.48E-04 |  |  |  |  |
| VEGFD | 1.77 (1.24-2.53) | 1.78E-03 |  | PLXNB2 | 3.82 (1.91-7.64) | 1.48E-04 |  |  |  |  |
| SCARF2 | 1.89 (1.27-2.81) | 1.80E-03 |  | TNF | 1.68 (1.29-2.2) | 1.52E-04 |  |  |  |  |
| HPGDS | 0.55 (0.38-0.8) | 1.81E-03 |  | CCL17 | 1.49 (1.21-1.83) | 1.54E-04 |  |  |  |  |
| CPXM2 | 2.13 (1.32-3.43) | 1.84E-03 |  | SCARF2 | 2.96 (1.69-5.19) | 1.54E-04 |  |  |  |  |
| CSNK1D | 0.63 (0.47-0.84) | 1.87E-03 |  | KLK10 | 1.98 (1.39-2.83) | 1.60E-04 |  |  |  |  |
| CELSR2 | 0.42 (0.24-0.73) | 1.89E-03 |  | REG1A | 1.8 (1.33-2.45) | 1.61E-04 |  |  |  |  |
| TP53BP1 | 0.73 (0.6-0.89) | 1.93E-03 |  | CTSD | 1.93 (1.37-2.71) | 1.62E-04 |  |  |  |  |
| MEGF10 | 0.59 (0.42-0.82) | 1.94E-03 |  | MMP10 | 1.69 (1.29-2.23) | 1.72E-04 |  |  |  |  |
| PTPRN2 | 1.83 (1.25-2.69) | 1.96E-03 |  | IL17D | 1.73 (1.3-2.31) | 1.79E-04 |  |  |  |  |
| SHBG | 1.51 (1.16-1.97) | 1.98E-03 |  | UNC79 | 0.39 (0.24-0.64) | 1.86E-04 |  |  |  |  |
| ADGRE2 | 0.48 (0.3-0.77) | 2.01E-03 |  | CTSV | 0.41 (0.25-0.65) | 1.88E-04 |  |  |  |  |
| DNPEP | 0.69 (0.55-0.88) | 2.06E-03 |  | CFI | 6.51 (2.43-17.39) | 1.89E-04 |  |  |  |  |
| FURIN | 1.74 (1.22-2.47) | 2.06E-03 |  | CD38 | 2.5 (1.54-4.04) | 1.90E-04 |  |  |  |  |
| CNTN1 | 0.5 (0.32-0.78) | 2.06E-03 |  | TPSAB1 | 2.05 (1.41-2.99) | 1.95E-04 |  |  |  |  |
| RARRES2 | 1.43 (1.14-1.8) | 2.09E-03 |  | C7 | 3.32 (1.77-6.25) | 1.97E-04 |  |  |  |  |
| ACTA2 | 1.44 (1.14-1.81) | 2.13E-03 |  | CD207 | 2.5 (1.54-4.06) | 2.01E-04 |  |  |  |  |
| RAB6A | 0.63 (0.47-0.85) | 2.17E-03 |  | MPO | 1.85 (1.34-2.56) | 2.06E-04 |  |  |  |  |
| OSCAR | 1.85 (1.25-2.73) | 2.20E-03 |  | ADAMTSL2 | 2.62 (1.58-4.37) | 2.08E-04 |  |  |  |  |
| NECTIN4 | 1.78 (1.23-2.59) | 2.22E-03 |  | FAM3B | 2.98 (1.67-5.32) | 2.12E-04 |  |  |  |  |
| CD101 | 1.66 (1.2-2.3) | 2.25E-03 |  | DCBLD2 | 2.56 (1.56-4.2) | 2.13E-04 |  |  |  |  |
| HLA.E | 1.89 (1.25-2.83) | 2.27E-03 |  | CCL25 | 2.03 (1.39-2.95) | 2.30E-04 |  |  |  |  |
| CD207 | 1.7 (1.21-2.39) | 2.32E-03 |  | KITLG | 0.41 (0.26-0.66) | 2.43E-04 |  |  |  |  |
| DKK4 | 0.62 (0.46-0.84) | 2.34E-03 |  | B3GNT7 | 3.07 (1.69-5.6) | 2.46E-04 |  |  |  |  |
| CSF2RB | 0.58 (0.41-0.82) | 2.40E-03 |  | UMOD | 0.56 (0.41-0.77) | 2.53E-04 |  |  |  |  |
| SMPD1 | 1.47 (1.15-1.88) | 2.46E-03 |  | SEPTIN3 | 1.53 (1.22-1.91) | 2.59E-04 |  |  |  |  |
| PI3 | 1.38 (1.12-1.69) | 2.55E-03 |  | CRH | 0.64 (0.5-0.81) | 2.76E-04 |  |  |  |  |
| VEGFA | 1.38 (1.12-1.71) | 2.63E-03 |  | TGFB1 | 2.13 (1.42-3.21) | 2.83E-04 |  |  |  |  |
| ITGA11 | 0.56 (0.38-0.82) | 2.65E-03 |  | CDH2 | 2.42 (1.5-3.9) | 2.87E-04 |  |  |  |  |
| TNFRSF14 | 1.83 (1.23-2.72) | 2.66E-03 |  | SPINT2 | 2.4 (1.49-3.85) | 2.98E-04 |  |  |  |  |
| NTRK3 | 0.42 (0.24-0.74) | 2.75E-03 |  | HSPG2 | 2.71 (1.58-4.67) | 3.07E-04 |  |  |  |  |
| HRC | 0.44 (0.25-0.75) | 2.75E-03 |  | BTN3A2 | 2.08 (1.4-3.1) | 3.18E-04 |  |  |  |  |
| GNPDA1 | 1.6 (1.17-2.17) | 2.79E-03 |  | ANXA10 | 1.43 (1.18-1.74) | 3.21E-04 |  |  |  |  |
| REG1B | 1.36 (1.11-1.66) | 2.83E-03 |  | CCL28 | 1.67 (1.26-2.21) | 3.27E-04 |  |  |  |  |
| AOC3 | 1.9 (1.25-2.89) | 2.84E-03 |  | ACRV1 | 1.34 (1.14-1.58) | 3.34E-04 |  |  |  |  |
| AFP | 1.33 (1.1-1.61) | 2.87E-03 |  | SORCS2 | 2.38 (1.48-3.82) | 3.37E-04 |  |  |  |  |
| ENO3 | 0.66 (0.5-0.87) | 3.02E-03 |  | LRTM2 | 0.2 (0.08-0.48) | 3.39E-04 |  |  |  |  |
| WNT9A | 1.8 (1.22-2.64) | 3.04E-03 |  | GAL | 0.58 (0.43-0.78) | 3.43E-04 |  |  |  |  |
| CD99L2 | 2.17 (1.3-3.63) | 3.09E-03 |  | IL18R1 | 2.61 (1.54-4.41) | 3.43E-04 |  |  |  |  |
| KLK13 | 1.54 (1.15-2.05) | 3.26E-03 |  | MAG | 0.49 (0.33-0.73) | 3.50E-04 |  |  |  |  |
| ADAMTS16 | 1.49 (1.14-1.95) | 3.41E-03 |  | GDF2 | 0.38 (0.23-0.65) | 3.54E-04 |  |  |  |  |
| GPC1 | 0.52 (0.33-0.8) | 3.41E-03 |  | CLEC1A | 2.65 (1.55-4.52) | 3.58E-04 |  |  |  |  |
| ADAMTSL5 | 0.56 (0.38-0.83) | 3.44E-03 |  | CXCL13 | 1.43 (1.17-1.74) | 3.75E-04 |  |  |  |  |
| SDHB | 0.66 (0.5-0.87) | 3.44E-03 |  | CYTL1 | 4.16 (1.9-9.13) | 3.76E-04 |  |  |  |  |
| LRIG1 | 1.73 (1.2-2.49) | 3.55E-03 |  | LY6D | 2.06 (1.38-3.08) | 3.79E-04 |  |  |  |  |
| REG1A | 1.42 (1.12-1.79) | 3.59E-03 |  | MRC1 | 3.31 (1.71-6.39) | 3.78E-04 |  |  |  |  |
| CANT1 | 2.59 (1.36-4.92) | 3.76E-03 |  | TEX101 | 1.65 (1.25-2.17) | 4.34E-04 |  |  |  |  |
| COL6A3 | 1.6 (1.16-2.2) | 3.80E-03 |  | CSPG4 | 0.35 (0.19-0.63) | 4.48E-04 |  |  |  |  |
| ENPEP | 1.42 (1.12-1.79) | 3.81E-03 |  | IL10RB | 2.3 (1.44-3.66) | 4.66E-04 |  |  |  |  |
| LRTM2 | 0.42 (0.23-0.75) | 3.83E-03 |  | ASGR2 | 3.75 (1.79-7.87) | 4.76E-04 |  |  |  |  |
| TAFA5 | 1.65 (1.18-2.32) | 3.88E-03 |  | FGL1 | 1.77 (1.28-2.43) | 5.03E-04 |  |  |  |  |
| AXL | 0.49 (0.3-0.8) | 3.95E-03 |  | TIMD4 | 2.09 (1.38-3.17) | 5.04E-04 |  |  |  |  |
| CCN1 | 1.3 (1.09-1.56) | 4.04E-03 |  | IL1RN | 1.56 (1.22-2.01) | 5.09E-04 |  |  |  |  |
| RNASET2 | 1.89 (1.22-2.92) | 4.13E-03 |  | PCDH9 | 0.42 (0.26-0.69) | 5.29E-04 |  |  |  |  |
| STOML2 | 0.74 (0.61-0.91) | 4.17E-03 |  | GM2A | 1.74 (1.27-2.38) | 5.38E-04 |  |  |  |  |
| IGFBP7 | 1.65 (1.17-2.33) | 4.40E-03 |  | AMOT | 0.37 (0.21-0.65) | 5.45E-04 |  |  |  |  |
| JCHAIN | 1.37 (1.1-1.69) | 4.42E-03 |  | HLA.A | 3.58 (1.74-7.38) | 5.44E-04 |  |  |  |  |
| SERPINA4 | 0.4 (0.21-0.75) | 4.42E-03 |  | ANG | 2.28 (1.43-3.65) | 5.52E-04 |  |  |  |  |
| TFRC | 0.6 (0.42-0.85) | 4.49E-03 |  | SFTPA1 | 1.48 (1.18-1.85) | 5.83E-04 |  |  |  |  |
| TFPI | 1.64 (1.17-2.32) | 4.57E-03 |  | SNAPIN | 0.48 (0.31-0.73) | 5.86E-04 |  |  |  |  |
| ADAMTS4 | 1.68 (1.17-2.41) | 4.57E-03 |  | ADAM8 | 2.75 (1.54-4.9) | 5.90E-04 |  |  |  |  |
| CRHBP | 1.93 (1.22-3.04) | 4.58E-03 |  | ADAMTS15 | 2.11 (1.38-3.24) | 6.12E-04 |  |  |  |  |
| FCRLB | 1.3 (1.08-1.56) | 4.62E-03 |  | NEFL | 1.8 (1.28-2.52) | 6.25E-04 |  |  |  |  |
| C7 | 1.97 (1.23-3.16) | 4.67E-03 |  | TNFRSF10C | 2.13 (1.38-3.3) | 6.93E-04 |  |  |  |  |
| ADGRG2 | 0.55 (0.37-0.83) | 4.72E-03 |  | PLTP | 0.44 (0.27-0.71) | 6.97E-04 |  |  |  |  |
| KLK10 | 1.53 (1.14-2.06) | 4.77E-03 |  | CCL27 | 1.72 (1.26-2.35) | 7.06E-04 |  |  |  |  |
| RBP7 | 1.34 (1.09-1.65) | 4.81E-03 |  | CD99L2 | 3.53 (1.7-7.33) | 7.15E-04 |  |  |  |  |
| GALNT5 | 2.03 (1.24-3.33) | 4.88E-03 |  | CLMP | 3.48 (1.68-7.18) | 7.61E-04 |  |  |  |  |
| CD58 | 0.35 (0.17-0.73) | 4.91E-03 |  | NECTIN2 | 2.64 (1.5-4.65) | 7.63E-04 |  |  |  |  |
| SPINT1 | 1.88 (1.21-2.93) | 4.92E-03 |  | IL4R | 2.18 (1.38-3.44) | 7.72E-04 |  |  |  |  |
| LY6D | 1.56 (1.14-2.13) | 5.09E-03 |  | SLITRK2 | 0.48 (0.31-0.74) | 7.82E-04 |  |  |  |  |
| GFRA1 | 1.81 (1.2-2.75) | 5.13E-03 |  | TRAF2 | 0.61 (0.45-0.81) | 8.40E-04 |  |  |  |  |
| MYOM3 | 0.79 (0.67-0.93) | 5.18E-03 |  | TIMP4 | 1.95 (1.32-2.89) | 8.45E-04 |  |  |  |  |
| KRT19 | 1.3 (1.08-1.56) | 5.22E-03 |  | DDR1 | 3.1 (1.6-6.03) | 8.48E-04 |  |  |  |  |
| CHGA | 1.24 (1.07-1.44) | 5.28E-03 |  | PPP1R14D | 1.93 (1.31-2.84) | 8.64E-04 |  |  |  |  |
| CLEC7A | 1.48 (1.12-1.95) | 5.39E-03 |  | LTBR | 2.68 (1.5-4.78) | 8.83E-04 |  |  |  |  |
| PLB1 | 0.71 (0.56-0.91) | 5.41E-03 |  | CPM | 2.26 (1.39-3.66) | 9.25E-04 |  |  |  |  |
| BOC | 0.45 (0.25-0.79) | 5.43E-03 |  | DEFA1_DEFA1B | 1.58 (1.21-2.08) | 9.31E-04 |  |  |  |  |
| ADGRE5 | 0.53 (0.34-0.83) | 5.43E-03 |  | GPR37 | 1.63 (1.22-2.18) | 9.33E-04 |  |  |  |  |
| FABP3 | 1.4 (1.1-1.78) | 5.49E-03 |  | GUCA2A | 2.38 (1.43-3.98) | 9.30E-04 |  |  |  |  |
| PRSS2 | 1.37 (1.1-1.72) | 5.62E-03 |  | ESM1 | 0.39 (0.22-0.68) | 9.48E-04 |  |  |  |  |
| ESAM | 1.73 (1.17-2.54) | 5.64E-03 |  | ITPR1 | 1.46 (1.17-1.83) | 9.52E-04 |  |  |  |  |
| CNDP1 | 0.69 (0.53-0.9) | 5.71E-03 |  | CHCHD6 | 2.17 (1.37-3.45) | 9.56E-04 |  |  |  |  |
| CSF3 | 0.67 (0.51-0.89) | 5.75E-03 |  | CCL15 | 1.89 (1.29-2.76) | 9.99E-04 |  |  |  |  |
| ADGRF5 | 2.11 (1.24-3.59) | 5.75E-03 |  | CTHRC1 | 2.76 (1.51-5.06) | 1.00E-03 |  |  |  |  |
| CA4 | 2.1 (1.24-3.56) | 5.93E-03 |  | CRNN | 0.64 (0.48-0.83) | 1.00E-03 |  |  |  |  |
| FGFBP2 | 0.64 (0.47-0.88) | 6.04E-03 |  | SEMA3F | 3.07 (1.57-5.99) | 1.02E-03 |  |  |  |  |
| LSP1 | 1.53 (1.13-2.06) | 6.05E-03 |  | REG1B | 1.59 (1.2-2.09) | 1.02E-03 |  |  |  |  |
| IL2RB | 0.55 (0.35-0.84) | 6.09E-03 |  | PTP4A3 | 1.65 (1.22-2.23) | 1.06E-03 |  |  |  |  |
| NCF2 | 1.21 (1.05-1.38) | 6.16E-03 |  | MMP3 | 1.65 (1.22-2.24) | 1.06E-03 |  |  |  |  |
| LMNB2 | 1.87 (1.19-2.92) | 6.25E-03 |  | CSF2RA | 1.54 (1.19-1.99) | 1.07E-03 |  |  |  |  |
| ANGPT1 | 1.26 (1.07-1.48) | 6.27E-03 |  | HADH | 1.4 (1.14-1.71) | 1.10E-03 |  |  |  |  |
| EIF2AK3 | 0.69 (0.52-0.9) | 6.69E-03 |  | ACTA2 | 1.66 (1.22-2.25) | 1.13E-03 |  |  |  |  |
| LILRB4 | 1.47 (1.11-1.95) | 6.71E-03 |  | ADA2 | 1.98 (1.31-2.99) | 1.13E-03 |  |  |  |  |
| MMP7 | 1.46 (1.11-1.93) | 6.77E-03 |  | SIGLEC5 | 1.48 (1.17-1.88) | 1.15E-03 |  |  |  |  |
| BTN2A1 | 1.9 (1.19-3.03) | 6.80E-03 |  | APOM | 0.34 (0.18-0.65) | 1.18E-03 |  |  |  |  |
| FLT3 | 0.51 (0.32-0.83) | 6.84E-03 |  | EZR | 3.07 (1.56-6.05) | 1.20E-03 |  |  |  |  |
| KLK14 | 1.45 (1.11-1.91) | 6.85E-03 |  | GCNT1 | 2.38 (1.41-4.02) | 1.20E-03 |  |  |  |  |
| SORD | 0.78 (0.65-0.93) | 6.96E-03 |  | MCAM | 0.39 (0.22-0.69) | 1.21E-03 |  |  |  |  |
| DLL4 | 1.17 (1.04-1.31) | 7.04E-03 |  | VWA1 | 2.04 (1.32-3.13) | 1.20E-03 |  |  |  |  |
| CCL16 | 1.4 (1.1-1.78) | 7.12E-03 |  | SERPINA1 | 11.89 (2.65-53.31) | 1.22E-03 |  |  |  |  |
| EFEMP1 | 1.66 (1.15-2.4) | 7.18E-03 |  | LECT2 | 1.55 (1.19-2.01) | 1.24E-03 |  |  |  |  |
| SPP1 | 1.46 (1.11-1.92) | 7.24E-03 |  | CTSL | 3.22 (1.58-6.55) | 1.26E-03 |  |  |  |  |
| PTPRZ1 | 1.77 (1.17-2.69) | 7.43E-03 |  | HYAL1 | 3.18 (1.57-6.42) | 1.26E-03 |  |  |  |  |
| CD248 | 0.61 (0.42-0.88) | 7.44E-03 |  | NEXN | 0.54 (0.38-0.79) | 1.29E-03 |  |  |  |  |
| NECTIN2 | 1.76 (1.16-2.67) | 7.46E-03 |  | TLR2 | 3.26 (1.58-6.69) | 1.33E-03 |  |  |  |  |
| SYNGAP1 | 1.33 (1.08-1.65) | 7.54E-03 |  | BAG6 | 0.39 (0.22-0.69) | 1.35E-03 |  |  |  |  |
| PDGFB | 1.24 (1.06-1.45) | 7.64E-03 |  | NPL | 1.94 (1.29-2.9) | 1.36E-03 |  |  |  |  |
| KLK11 | 1.61 (1.14-2.3) | 7.64E-03 |  | CRIP2 | 1.82 (1.26-2.63) | 1.37E-03 |  |  |  |  |
| CD244 | 0.52 (0.32-0.84) | 7.76E-03 |  | RET | 0.45 (0.28-0.74) | 1.38E-03 |  |  |  |  |
| ENPP5 | 0.72 (0.57-0.92) | 7.77E-03 |  | SIAE | 1.91 (1.28-2.84) | 1.40E-03 |  |  |  |  |
| MBL2 | 1.22 (1.05-1.42) | 7.83E-03 |  | PAG1 | 1.62 (1.2-2.17) | 1.41E-03 |  |  |  |  |
| LY96 | 1.56 (1.12-2.16) | 8.00E-03 |  | TNFRSF21 | 2.86 (1.5-5.45) | 1.45E-03 |  |  |  |  |
| POLR2F | 1.47 (1.1-1.95) | 8.13E-03 |  | TAB2 | 0.7 (0.56-0.87) | 1.50E-03 |  |  |  |  |
| COL24A1 | 1.48 (1.11-1.98) | 8.19E-03 |  | CTSO | 2.46 (1.41-4.3) | 1.52E-03 |  |  |  |  |
| KEL | 0.61 (0.42-0.88) | 8.20E-03 |  | NHLRC3 | 3.12 (1.54-6.3) | 1.54E-03 |  |  |  |  |
| CCL14 | 1.47 (1.11-1.96) | 8.20E-03 |  | APPL2 | 0.73 (0.6-0.89) | 1.59E-03 |  |  |  |  |
| USP28 | 1.5 (1.11-2.03) | 8.21E-03 |  | DDX1 | 0.35 (0.18-0.67) | 1.58E-03 |  |  |  |  |
| NPTX1 | 1.51 (1.11-2.05) | 8.21E-03 |  | IGHMBP2 | 0.42 (0.25-0.72) | 1.59E-03 |  |  |  |  |
| SERPING1 | 2.93 (1.32-6.51) | 8.32E-03 |  | ITGAM | 0.36 (0.19-0.68) | 1.58E-03 |  |  |  |  |
| CST5 | 1.36 (1.08-1.71) | 8.48E-03 |  | NUCB2 | 2.17 (1.34-3.51) | 1.60E-03 |  |  |  |  |
| ARHGAP1 | 0.68 (0.51-0.91) | 8.53E-03 |  | LRG1 | 3.19 (1.55-6.57) | 1.63E-03 |  |  |  |  |
| B2M | 1.45 (1.1-1.92) | 8.56E-03 |  | LRRN1 | 0.45 (0.28-0.74) | 1.64E-03 |  |  |  |  |
| ORM1 | 2.56 (1.27-5.17) | 8.68E-03 |  | ARAF | 0.52 (0.34-0.78) | 1.65E-03 |  |  |  |  |
| CD14 | 1.51 (1.11-2.05) | 8.78E-03 |  | GHR | 0.31 (0.15-0.65) | 1.65E-03 |  |  |  |  |
| ANXA2 | 1.29 (1.06-1.55) | 9.27E-03 |  | ACP6 | 1.78 (1.24-2.56) | 1.67E-03 |  |  |  |  |
| LRPAP1 | 1.29 (1.07-1.57) | 9.30E-03 |  | DTX3 | 2.73 (1.46-5.11) | 1.67E-03 |  |  |  |  |
| NDUFB7 | 0.79 (0.66-0.94) | 9.33E-03 |  | TRIM25 | 0.71 (0.58-0.88) | 1.68E-03 |  |  |  |  |
| CPXM1 | 1.28 (1.06-1.55) | 9.38E-03 |  | SMOC2 | 1.99 (1.29-3.05) | 1.74E-03 |  |  |  |  |
| GIPC2 | 1.8 (1.16-2.82) | 9.44E-03 |  | BCL2L11 | 2.37 (1.38-4.07) | 1.77E-03 |  |  |  |  |
| IL18 | 1.41 (1.09-1.84) | 9.45E-03 |  | COQ7 | 0.33 (0.16-0.66) | 1.78E-03 |  |  |  |  |
| CD70 | 1.48 (1.1-2) | 9.46E-03 |  | RBP5 | 1.58 (1.18-2.1) | 1.84E-03 |  |  |  |  |
| PINLYP | 0.75 (0.6-0.93) | 9.46E-03 |  | C1RL | 5.98 (1.93-18.54) | 1.94E-03 |  |  |  |  |
| EGFLAM | 1.73 (1.14-2.61) | 9.86E-03 |  | ADD1 | 0.6 (0.43-0.83) | 2.00E-03 |  |  |  |  |
| FNDC1 | 1.54 (1.11-2.13) | 9.89E-03 |  | TRIM21 | 0.71 (0.57-0.88) | 2.00E-03 |  |  |  |  |
| CLEC4G | 1.67 (1.13-2.46) | 9.97E-03 |  | ACP5 | 2.08 (1.31-3.31) | 2.02E-03 |  |  |  |  |
| IL15RA | 1.65 (1.13-2.41) | 1.00E-02 |  | CXCL8 | 1.39 (1.13-1.71) | 2.02E-03 |  |  |  |  |
| EZR | 1.92 (1.17-3.17) | 1.01E-02 |  | FLT3 | 0.32 (0.16-0.66) | 2.05E-03 |  |  |  |  |
| SMNDC1 | 1.28 (1.06-1.54) | 1.01E-02 |  | ESAM | 2.36 (1.37-4.07) | 2.07E-03 |  |  |  |  |
| IGSF21 | 0.56 (0.36-0.87) | 1.02E-02 |  | VNN2 | 2.12 (1.31-3.41) | 2.09E-03 |  |  |  |  |
| HLA.DRA | 1.59 (1.11-2.25) | 1.04E-02 |  | LY96 | 1.98 (1.28-3.06) | 2.11E-03 |  |  |  |  |
| CCL19 | 1.12 (1.03-1.23) | 1.04E-02 |  | ORM1 | 4.46 (1.72-11.59) | 2.12E-03 |  |  |  |  |
| PPP1R9B | 0.83 (0.72-0.96) | 1.06E-02 |  | CD300C | 2.25 (1.34-3.77) | 2.13E-03 |  |  |  |  |
| APOA2 | 1.48 (1.1-2) | 1.06E-02 |  | HNMT | 1.78 (1.23-2.57) | 2.13E-03 |  |  |  |  |
| KIR2DL2 | 1.25 (1.05-1.49) | 1.11E-02 |  | OSCAR | 2.49 (1.39-4.45) | 2.17E-03 |  |  |  |  |
| FABP1 | 1.18 (1.04-1.34) | 1.11E-02 |  | BMP10 | 0.33 (0.16-0.67) | 2.18E-03 |  |  |  |  |
| GHRL | 1.22 (1.05-1.43) | 1.11E-02 |  | CTSZ | 2.46 (1.38-4.38) | 2.20E-03 |  |  |  |  |
| RNF41 | 0.76 (0.62-0.94) | 1.12E-02 |  | LILRB2 | 2.53 (1.39-4.59) | 2.29E-03 |  |  |  |  |
| TIMP4 | 1.46 (1.09-1.95) | 1.14E-02 |  | MMP1 | 1.45 (1.14-1.84) | 2.31E-03 |  |  |  |  |
| ADAM8 | 1.77 (1.14-2.76) | 1.16E-02 |  | ITGA11 | 0.42 (0.24-0.74) | 2.34E-03 |  |  |  |  |
| LCN15 | 1.29 (1.06-1.57) | 1.20E-02 |  | KLK14 | 1.84 (1.24-2.73) | 2.35E-03 |  |  |  |  |
| GNPDA2 | 0.57 (0.37-0.88) | 1.21E-02 |  | RBP7 | 1.54 (1.16-2.02) | 2.35E-03 |  |  |  |  |
| PARP1 | 1.25 (1.05-1.49) | 1.21E-02 |  | SELE | 1.78 (1.23-2.57) | 2.34E-03 |  |  |  |  |
| BDNF | 1.28 (1.06-1.55) | 1.22E-02 |  | B4GAT1 | 0.3 (0.14-0.65) | 2.41E-03 |  |  |  |  |
| SERPINE2 | 1.34 (1.07-1.69) | 1.22E-02 |  | IL24 | 1.54 (1.17-2.04) | 2.42E-03 |  |  |  |  |
| PTGR1 | 0.79 (0.65-0.95) | 1.24E-02 |  | LTBP2 | 2.15 (1.31-3.52) | 2.46E-03 |  |  |  |  |
| SERPINB8 | 1.25 (1.05-1.5) | 1.24E-02 |  | SUGP1 | 0.35 (0.18-0.69) | 2.50E-03 |  |  |  |  |
| TNFSF14 | 1.33 (1.06-1.66) | 1.25E-02 |  | COMMD9 | 0.33 (0.16-0.68) | 2.50E-03 |  |  |  |  |
| PDGFA | 1.27 (1.05-1.52) | 1.25E-02 |  | MENT | 0.26 (0.11-0.62) | 2.53E-03 |  |  |  |  |
| SPARC | 1.29 (1.06-1.58) | 1.25E-02 |  | CILP | 1.85 (1.24-2.76) | 2.58E-03 |  |  |  |  |
| SMOC2 | 1.48 (1.09-2.01) | 1.26E-02 |  | CTSH | 1.5 (1.15-1.95) | 2.59E-03 |  |  |  |  |
| FAM3C | 1.7 (1.12-2.57) | 1.26E-02 |  | DLG4 | 0.57 (0.4-0.82) | 2.59E-03 |  |  |  |  |
| GAL | 0.78 (0.64-0.95) | 1.27E-02 |  | ACAN | 0.37 (0.2-0.71) | 2.61E-03 |  |  |  |  |
| PTH1R | 1.37 (1.07-1.77) | 1.30E-02 |  | SERPING1 | 5.74 (1.84-17.92) | 2.61E-03 |  |  |  |  |
| HSPG2 | 1.73 (1.12-2.67) | 1.32E-02 |  | CALCOCO1 | 0.75 (0.62-0.9) | 2.75E-03 |  |  |  |  |
| DSG3 | 0.62 (0.43-0.91) | 1.33E-02 |  | IGFBP6 | 2.11 (1.29-3.44) | 2.78E-03 |  |  |  |  |
| CD72 | 0.63 (0.43-0.91) | 1.34E-02 |  | CCL3 | 1.31 (1.1-1.56) | 2.79E-03 |  |  |  |  |
| FSTL1 | 2.18 (1.18-4.06) | 1.35E-02 |  | EVI5 | 0.66 (0.51-0.87) | 2.80E-03 |  |  |  |  |
| CCL15 | 1.41 (1.07-1.85) | 1.36E-02 |  | IL3RA | 2.26 (1.32-3.87) | 2.83E-03 |  |  |  |  |
| CD6 | 1.36 (1.06-1.73) | 1.37E-02 |  | ENTPD2 | 0.53 (0.35-0.8) | 2.92E-03 |  |  |  |  |
| PLA2G15 | 1.8 (1.13-2.87) | 1.40E-02 |  | PTK7 | 1.93 (1.25-2.98) | 2.93E-03 |  |  |  |  |
| AGR2 | 1.18 (1.03-1.35) | 1.42E-02 |  | SMAD5 | 4.02 (1.61-10.08) | 2.96E-03 |  |  |  |  |
| PRG2 | 1.42 (1.07-1.87) | 1.42E-02 |  | WASL | 0.47 (0.28-0.77) | 2.96E-03 |  |  |  |  |
| LGALS4 | 1.32 (1.06-1.65) | 1.42E-02 |  | CDHR2 | 1.41 (1.12-1.77) | 2.98E-03 |  |  |  |  |
| DEFB4A_DEFB4B | 1.11 (1.02-1.21) | 1.51E-02 |  | TNFRSF11B | 2.36 (1.34-4.15) | 3.01E-03 |  |  |  |  |
| VIT | 1.57 (1.09-2.25) | 1.52E-02 |  | ADAMTS4 | 2.02 (1.27-3.21) | 3.09E-03 |  |  |  |  |
| STX16 | 0.71 (0.54-0.94) | 1.52E-02 |  | PRSS2 | 1.58 (1.17-2.15) | 3.09E-03 |  |  |  |  |
| LGALS7_LGALS7B | 1.4 (1.07-1.85) | 1.53E-02 |  | CD14 | 1.91 (1.24-2.93) | 3.11E-03 |  |  |  |  |
| IFNGR2 | 1.35 (1.06-1.71) | 1.53E-02 |  | CD79B | 1.78 (1.21-2.61) | 3.17E-03 |  |  |  |  |
| CLASP1 | 0.72 (0.55-0.94) | 1.54E-02 |  | PTGR1 | 1.45 (1.13-1.86) | 3.16E-03 |  |  |  |  |
| CTSO | 1.64 (1.1-2.45) | 1.54E-02 |  | SLAMF1 | 1.65 (1.18-2.3) | 3.17E-03 |  |  |  |  |
| TXNDC15 | 1.77 (1.11-2.81) | 1.54E-02 |  | USP47 | 0.57 (0.4-0.83) | 3.17E-03 |  |  |  |  |
| C1S | 2.29 (1.17-4.49) | 1.55E-02 |  | LGALS7_LGALS7B | 1.72 (1.2-2.47) | 3.21E-03 |  |  |  |  |
| PRND | 1.42 (1.07-1.88) | 1.56E-02 |  | C1QA | 3.12 (1.46-6.66) | 3.31E-03 |  |  |  |  |
| L1CAM | 1.73 (1.11-2.69) | 1.58E-02 |  | PRR4 | 1.54 (1.15-2.06) | 3.31E-03 |  |  |  |  |
| CXADR | 1.35 (1.06-1.71) | 1.58E-02 |  | MAP3K5 | 0.71 (0.57-0.89) | 3.38E-03 |  |  |  |  |
| CHCHD10 | 1.46 (1.07-1.99) | 1.59E-02 |  | TGFBR3 | 2.57 (1.37-4.84) | 3.44E-03 |  |  |  |  |
| MSLNL | 0.56 (0.34-0.9) | 1.65E-02 |  | GGH | 1.96 (1.25-3.08) | 3.51E-03 |  |  |  |  |
| SMAD3 | 0.68 (0.49-0.93) | 1.65E-02 |  | OGT | 0.45 (0.26-0.77) | 3.50E-03 |  |  |  |  |
| SV2A | 0.7 (0.52-0.94) | 1.67E-02 |  | DDC | 0.54 (0.36-0.82) | 3.57E-03 |  |  |  |  |
| SORT1 | 1.52 (1.08-2.15) | 1.67E-02 |  | NOS1 | 1.57 (1.16-2.13) | 3.60E-03 |  |  |  |  |
| BCL2L11 | 1.51 (1.08-2.12) | 1.68E-02 |  | LGALS1 | 2.04 (1.26-3.3) | 3.65E-03 |  |  |  |  |
| TNFRSF11A | 1.45 (1.07-1.98) | 1.69E-02 |  | CLEC4A | 0.49 (0.3-0.79) | 3.75E-03 |  |  |  |  |
| KAZALD1 | 0.72 (0.55-0.94) | 1.71E-02 |  | SDCCAG8 | 0.74 (0.61-0.91) | 3.77E-03 |  |  |  |  |
| CTHRC1 | 1.73 (1.1-2.72) | 1.74E-02 |  | TNFRSF13B | 1.92 (1.24-3) | 3.78E-03 |  |  |  |  |
| PRCP | 1.56 (1.08-2.26) | 1.76E-02 |  | CFB | 2.62 (1.36-5.02) | 3.85E-03 |  |  |  |  |
| VEGFC | 1.34 (1.05-1.7) | 1.78E-02 |  | LILRA5 | 2.28 (1.3-3.99) | 4.02E-03 |  |  |  |  |
| TMPRSS11D | 1.52 (1.08-2.15) | 1.78E-02 |  | UGDH | 0.53 (0.35-0.82) | 4.06E-03 |  |  |  |  |
| TARM1 | 1.22 (1.03-1.44) | 1.79E-02 |  | JAM2 | 2.52 (1.34-4.73) | 4.09E-03 |  |  |  |  |
| CARHSP1 | 1.34 (1.05-1.7) | 1.79E-02 |  | DCTN2 | 0.72 (0.57-0.9) | 4.13E-03 |  |  |  |  |
| GLRX | 1.3 (1.05-1.62) | 1.80E-02 |  | SIGLEC15 | 1.32 (1.09-1.59) | 4.14E-03 |  |  |  |  |
| CA5A | 0.84 (0.72-0.97) | 1.82E-02 |  | PDCD1 | 1.77 (1.2-2.62) | 4.17E-03 |  |  |  |  |
| CD300C | 1.55 (1.08-2.22) | 1.83E-02 |  | ITGA2 | 0.39 (0.2-0.74) | 4.21E-03 |  |  |  |  |
| SLAMF1 | 1.32 (1.05-1.67) | 1.86E-02 |  | CD4 | 1.82 (1.21-2.75) | 4.23E-03 |  |  |  |  |
| CCL13 | 1.25 (1.04-1.5) | 1.88E-02 |  | ICOSLG | 0.22 (0.08-0.62) | 4.23E-03 |  |  |  |  |
| LYVE1 | 0.39 (0.18-0.86) | 1.89E-02 |  | RABEP1 | 0.64 (0.47-0.87) | 4.24E-03 |  |  |  |  |
| BCL2L15 | 1.3 (1.04-1.62) | 1.90E-02 |  | SCN4B | 2.05 (1.25-3.35) | 4.35E-03 |  |  |  |  |
| DHODH | 0.61 (0.4-0.92) | 1.90E-02 |  | IL17C | 1.4 (1.11-1.76) | 4.44E-03 |  |  |  |  |
| FGF5 | 1.54 (1.07-2.21) | 1.90E-02 |  | RAB11FIP3 | 0.74 (0.6-0.91) | 4.45E-03 |  |  |  |  |
| ARAF | 0.73 (0.56-0.95) | 1.91E-02 |  | PSPN | 1.25 (1.07-1.45) | 4.56E-03 |  |  |  |  |
| FRMD4B | 0.75 (0.59-0.95) | 1.92E-02 |  | CHAD | 0.54 (0.35-0.83) | 4.62E-03 |  |  |  |  |
| IL10RB | 1.61 (1.08-2.39) | 1.92E-02 |  | COCH | 1.86 (1.21-2.87) | 4.74E-03 |  |  |  |  |
| NUCB2 | 1.49 (1.07-2.09) | 1.94E-02 |  | AGR2 | 1.31 (1.09-1.59) | 4.79E-03 |  |  |  |  |
| IGLC2 | 1.57 (1.08-2.29) | 1.94E-02 |  | CFD | 2.77 (1.36-5.62) | 4.80E-03 |  |  |  |  |
| CCN4 | 1.43 (1.06-1.94) | 1.94E-02 |  | DPY30 | 1.47 (1.12-1.92) | 4.84E-03 |  |  |  |  |
| SERPINE1 | 1.27 (1.04-1.55) | 1.95E-02 |  | ENG | 4.68 (1.6-13.72) | 4.87E-03 |  |  |  |  |
| WFDC12 | 1.24 (1.04-1.49) | 1.95E-02 |  | CRACR2A | 0.68 (0.52-0.89) | 4.92E-03 |  |  |  |  |
| TMSB10 | 1.22 (1.03-1.45) | 2.00E-02 |  | ITGAV | 0.22 (0.07-0.63) | 4.90E-03 |  |  |  |  |
| WFIKKN2 | 0.65 (0.45-0.93) | 2.00E-02 |  | MUC16 | 0.87 (0.8-0.96) | 4.91E-03 |  |  |  |  |
| CLNS1A | 1.55 (1.07-2.23) | 2.00E-02 |  | OPHN1 | 0.74 (0.6-0.91) | 4.92E-03 |  |  |  |  |
| EPHA4 | 0.52 (0.3-0.9) | 2.02E-02 |  | SERPINB8 | 1.39 (1.11-1.76) | 5.01E-03 |  |  |  |  |
| TGFB1 | 1.46 (1.06-2.01) | 2.06E-02 |  | BAMBI | 2.16 (1.26-3.69) | 5.03E-03 |  |  |  |  |
| CDHR1 | 0.65 (0.46-0.94) | 2.07E-02 |  | BRSK2 | 0.35 (0.16-0.73) | 5.12E-03 |  |  |  |  |
| CD300LG | 0.66 (0.47-0.94) | 2.07E-02 |  | EPHB4 | 2.69 (1.34-5.37) | 5.13E-03 |  |  |  |  |
| DNER | 0.56 (0.34-0.91) | 2.07E-02 |  | IL10 | 1.3 (1.08-1.57) | 5.12E-03 |  |  |  |  |
| CD3G | 1.17 (1.02-1.34) | 2.08E-02 |  | NFAT5 | 0.63 (0.46-0.87) | 5.14E-03 |  |  |  |  |
| LGALS1 | 1.5 (1.06-2.12) | 2.10E-02 |  | SEPTIN8 | 2.04 (1.24-3.35) | 5.28E-03 |  |  |  |  |
| GORASP2 | 0.67 (0.47-0.94) | 2.11E-02 |  | TEX33 | 1.59 (1.15-2.2) | 5.29E-03 |  |  |  |  |
| MDGA1 | 0.8 (0.67-0.97) | 2.12E-02 |  | CAMSAP1 | 0.73 (0.59-0.91) | 5.66E-03 |  |  |  |  |
| KIR2DL3 | 1.22 (1.03-1.45) | 2.14E-02 |  | IDUA | 1.88 (1.2-2.94) | 5.69E-03 |  |  |  |  |
| RBP2 | 1.21 (1.03-1.43) | 2.25E-02 |  | ADAM12 | 1.69 (1.16-2.45) | 5.70E-03 |  |  |  |  |
| BSND | 0.52 (0.29-0.91) | 2.26E-02 |  | RGCC | 0.65 (0.48-0.88) | 5.77E-03 |  |  |  |  |
| SIT1 | 1.31 (1.04-1.65) | 2.28E-02 |  | CEACAM20 | 1.63 (1.15-2.31) | 5.80E-03 |  |  |  |  |
| TRIM21 | 0.83 (0.71-0.97) | 2.29E-02 |  | AKT3 | 0.59 (0.41-0.86) | 5.90E-03 |  |  |  |  |
| CTBS | 1.92 (1.09-3.39) | 2.31E-02 |  | OPTC | 0.52 (0.33-0.83) | 6.33E-03 |  |  |  |  |
| EPHA1 | 1.53 (1.06-2.22) | 2.33E-02 |  | CLSTN2 | 1.77 (1.17-2.66) | 6.39E-03 |  |  |  |  |
| SPON2 | 1.52 (1.06-2.18) | 2.34E-02 |  | GALNT2 | 0.36 (0.18-0.75) | 6.40E-03 |  |  |  |  |
| EPHB4 | 1.78 (1.08-2.95) | 2.35E-02 |  | UNC5D | 0.27 (0.1-0.69) | 6.43E-03 |  |  |  |  |
| DBN1 | 0.68 (0.49-0.95) | 2.36E-02 |  | NCR3LG1 | 2.16 (1.24-3.77) | 6.48E-03 |  |  |  |  |
| CLSTN1 | 0.71 (0.53-0.96) | 2.38E-02 |  | SCG2 | 2.24 (1.25-4.01) | 6.52E-03 |  |  |  |  |
| VAMP5 | 0.7 (0.51-0.95) | 2.42E-02 |  | SCP2 | 0.59 (0.4-0.86) | 6.55E-03 |  |  |  |  |
| SEMA3F | 1.7 (1.07-2.71) | 2.43E-02 |  | PPP1CC | 0.66 (0.49-0.89) | 6.62E-03 |  |  |  |  |
| CCN2 | 1.3 (1.03-1.64) | 2.48E-02 |  | HMGCS1 | 0.41 (0.22-0.78) | 6.72E-03 |  |  |  |  |
| SLMAP | 0.8 (0.66-0.97) | 2.49E-02 |  | MAEA | 0.44 (0.24-0.8) | 6.73E-03 |  |  |  |  |
| ICOSLG | 0.45 (0.22-0.9) | 2.49E-02 |  | ENPP5 | 0.64 (0.47-0.89) | 6.76E-03 |  |  |  |  |
| CEACAM3 | 1.32 (1.04-1.68) | 2.51E-02 |  | FCAMR | 1.46 (1.11-1.92) | 7.14E-03 |  |  |  |  |
| GATD3 | 0.78 (0.63-0.97) | 2.52E-02 |  | ITGAL | 2.14 (1.23-3.71) | 7.12E-03 |  |  |  |  |
| TACSTD2 | 0.57 (0.35-0.93) | 2.54E-02 |  | L1CAM | 2.45 (1.28-4.7) | 7.12E-03 |  |  |  |  |
| APOF | 2.03 (1.09-3.76) | 2.55E-02 |  | TMPRSS11D | 1.84 (1.18-2.86) | 7.15E-03 |  |  |  |  |
| PF4 | 1.19 (1.02-1.39) | 2.55E-02 |  | CCL16 | 1.65 (1.15-2.38) | 7.18E-03 |  |  |  |  |
| GALNT10 | 1.68 (1.06-2.64) | 2.58E-02 |  | TRIM58 | 0.69 (0.53-0.9) | 7.17E-03 |  |  |  |  |
| SPTLC1 | 0.54 (0.32-0.93) | 2.59E-02 |  | WWP2 | 0.59 (0.4-0.87) | 7.20E-03 |  |  |  |  |
| CYTL1 | 1.97 (1.08-3.58) | 2.60E-02 |  | SOX2 | 2.53 (1.28-5.01) | 7.48E-03 |  |  |  |  |
| PPY | 1.15 (1.02-1.3) | 2.62E-02 |  | S100G | 1.73 (1.16-2.58) | 7.51E-03 |  |  |  |  |
| CHRDL2 | 0.75 (0.59-0.97) | 2.67E-02 |  | TNFSF11 | 0.65 (0.47-0.89) | 7.61E-03 |  |  |  |  |
| ASAH2 | 0.78 (0.63-0.97) | 2.71E-02 |  | SLURP1 | 1.69 (1.15-2.49) | 7.64E-03 |  |  |  |  |
| SEZ6 | 0.55 (0.32-0.93) | 2.71E-02 |  | SPON2 | 2.02 (1.21-3.39) | 7.64E-03 |  |  |  |  |
| SERPINF2 | 0.29 (0.1-0.87) | 2.72E-02 |  | GIT1 | 0.72 (0.56-0.92) | 7.79E-03 |  |  |  |  |
| GSR | 0.47 (0.24-0.92) | 2.76E-02 |  | NT5C1A | 1.82 (1.17-2.84) | 7.89E-03 |  |  |  |  |
| NENF | 0.82 (0.68-0.98) | 2.77E-02 |  | AZI2 | 0.54 (0.34-0.85) | 8.06E-03 |  |  |  |  |
| PTN | 1.32 (1.03-1.69) | 2.77E-02 |  | DNER | 0.37 (0.17-0.77) | 8.07E-03 |  |  |  |  |
| GPR37 | 1.25 (1.02-1.52) | 2.79E-02 |  | WAS | 0.64 (0.47-0.89) | 8.06E-03 |  |  |  |  |
| TSPAN7 | 0.79 (0.63-0.97) | 2.79E-02 |  | IL1R1 | 2.8 (1.31-6) | 8.11E-03 |  |  |  |  |
| CXCL6 | 0.82 (0.69-0.98) | 2.82E-02 |  | GUSB | 1.45 (1.1-1.91) | 8.15E-03 |  |  |  |  |
| TRIM5 | 0.84 (0.72-0.98) | 2.83E-02 |  | KLKB1 | 0.32 (0.14-0.74) | 8.14E-03 |  |  |  |  |
| GGT5 | 1.81 (1.06-3.09) | 2.84E-02 |  | CSDE1 | 0.7 (0.54-0.91) | 8.17E-03 |  |  |  |  |
| CCL4 | 1.2 (1.02-1.41) | 2.87E-02 |  | ANKMY2 | 0.66 (0.49-0.9) | 8.32E-03 |  |  |  |  |
| FOLR2 | 1.6 (1.05-2.44) | 2.87E-02 |  | RPS10 | 0.37 (0.18-0.77) | 8.40E-03 |  |  |  |  |
| DLG4 | 0.77 (0.61-0.97) | 2.87E-02 |  | GIP | 1.64 (1.14-2.38) | 8.45E-03 |  |  |  |  |
| PSMA1 | 0.69 (0.49-0.96) | 2.90E-02 |  | POF1B | 1.66 (1.14-2.41) | 8.47E-03 |  |  |  |  |
| PRAP1 | 1.45 (1.04-2.03) | 2.90E-02 |  | MUC13 | 1.63 (1.13-2.33) | 8.49E-03 |  |  |  |  |
| CBS | 0.68 (0.48-0.96) | 2.91E-02 |  | IL12B | 0.63 (0.45-0.89) | 8.79E-03 |  |  |  |  |
| PAFAH1B3 | 0.83 (0.69-0.98) | 2.92E-02 |  | MB | 1.46 (1.1-1.94) | 8.80E-03 |  |  |  |  |
| CDHR2 | 1.18 (1.02-1.37) | 2.93E-02 |  | NFE2 | 0.64 (0.46-0.9) | 8.97E-03 |  |  |  |  |
| MAN1A2 | 1.92 (1.07-3.47) | 2.93E-02 |  | NPTX2 | 2.15 (1.21-3.82) | 8.96E-03 |  |  |  |  |
| BSG | 2.02 (1.07-3.79) | 2.94E-02 |  | VMO1 | 1.53 (1.11-2.11) | 8.99E-03 |  |  |  |  |
| CDC42BPB | 0.84 (0.72-0.98) | 2.94E-02 |  | DPP7 | 1.53 (1.11-2.1) | 9.13E-03 |  |  |  |  |
| IL1RN | 1.24 (1.02-1.51) | 2.95E-02 |  | TSPAN1 | 1.36 (1.08-1.72) | 9.14E-03 |  |  |  |  |
| SPINK2 | 1.48 (1.04-2.12) | 2.96E-02 |  | BIRC2 | 0.64 (0.45-0.89) | 9.17E-03 |  |  |  |  |
| ERBB2 | 1.74 (1.06-2.86) | 2.98E-02 |  | TSC22D1 | 0.6 (0.41-0.88) | 9.22E-03 |  |  |  |  |
| NID2 | 1.21 (1.02-1.45) | 2.99E-02 |  | LAMA4 | 2.04 (1.19-3.49) | 9.26E-03 |  |  |  |  |
| ARNT | 0.63 (0.41-0.96) | 2.99E-02 |  | LPL | 0.64 (0.46-0.9) | 9.29E-03 |  |  |  |  |
| CALCA | 1.2 (1.02-1.42) | 3.02E-02 |  | SDC1 | 1.71 (1.14-2.56) | 9.35E-03 |  |  |  |  |
| SMOC1 | 1.5 (1.04-2.15) | 3.06E-02 |  | GSAP | 0.6 (0.41-0.88) | 9.43E-03 |  |  |  |  |
| TYRP1 | 1.33 (1.03-1.73) | 3.06E-02 |  | KLK3 | 1.2 (1.05-1.38) | 9.55E-03 |  |  |  |  |
| ANXA1 | 1.36 (1.03-1.8) | 3.09E-02 |  | OSMR | 3.2 (1.33-7.71) | 9.63E-03 |  |  |  |  |
| STX1B | 1.35 (1.03-1.76) | 3.10E-02 |  | RNASE4 | 2.22 (1.21-4.06) | 9.78E-03 |  |  |  |  |
| HLA.A | 1.7 (1.05-2.76) | 3.10E-02 |  | TBC1D5 | 0.69 (0.52-0.91) | 9.77E-03 |  |  |  |  |
| CLEC4A | 0.68 (0.47-0.97) | 3.14E-02 |  | CCL4 | 1.32 (1.07-1.63) | 9.89E-03 |  |  |  |  |
| SIAE | 1.35 (1.03-1.78) | 3.14E-02 |  | APOL1 | 0.55 (0.34-0.86) | 9.95E-03 |  |  |  |  |
| DSG2 | 0.59 (0.36-0.95) | 3.15E-02 |  | MSLNL | 0.38 (0.19-0.8) | 1.01E-02 |  |  |  |  |
| NHLRC3 | 1.69 (1.05-2.73) | 3.15E-02 |  | PPY | 1.27 (1.06-1.53) | 1.01E-02 |  |  |  |  |
| GFRAL | 0.76 (0.59-0.98) | 3.17E-02 |  | ADAMTS13 | 0.36 (0.16-0.78) | 1.02E-02 |  |  |  |  |
| TIMP3 | 1.11 (1.01-1.23) | 3.17E-02 |  | RAD23B | 0.54 (0.34-0.87) | 1.04E-02 |  |  |  |  |
| SELENOP | 0.47 (0.23-0.94) | 3.25E-02 |  | FDX1 | 1.4 (1.08-1.82) | 1.05E-02 |  |  |  |  |
| SFRP4 | 0.67 (0.46-0.97) | 3.27E-02 |  | TNFRSF8 | 1.64 (1.12-2.39) | 1.06E-02 |  |  |  |  |
| CLMP | 1.77 (1.05-2.99) | 3.30E-02 |  | TRIM26 | 0.5 (0.29-0.85) | 1.06E-02 |  |  |  |  |
| HBZ | 1.16 (1.01-1.34) | 3.30E-02 |  | CDNF | 1.74 (1.14-2.68) | 1.07E-02 |  |  |  |  |
| CLEC4M | 1.41 (1.03-1.93) | 3.30E-02 |  | WFIKKN2 | 0.51 (0.31-0.86) | 1.08E-02 |  |  |  |  |
| CLIP2 | 0.88 (0.79-0.99) | 3.32E-02 |  | FGF23 | 1.38 (1.08-1.77) | 1.09E-02 |  |  |  |  |
| ESYT2 | 0.81 (0.67-0.98) | 3.33E-02 |  | RBP2 | 1.36 (1.07-1.73) | 1.09E-02 |  |  |  |  |
| CREG1 | 1.42 (1.03-1.96) | 3.34E-02 |  | TSPAN7 | 0.6 (0.41-0.89) | 1.10E-02 |  |  |  |  |
| MAPRE3 | 0.86 (0.75-0.99) | 3.34E-02 |  | AOC3 | 2.24 (1.2-4.18) | 1.12E-02 |  |  |  |  |
| SYT1 | 1.37 (1.02-1.83) | 3.35E-02 |  | STAB2 | 2.73 (1.26-5.95) | 1.12E-02 |  |  |  |  |
| RTN4R | 0.67 (0.47-0.97) | 3.36E-02 |  | XCL1 | 1.42 (1.08-1.86) | 1.13E-02 |  |  |  |  |
| FAP | 0.6 (0.37-0.96) | 3.39E-02 |  | ATRAID | 1.67 (1.12-2.49) | 1.13E-02 |  |  |  |  |
| SPARCL1 | 0.59 (0.36-0.96) | 3.43E-02 |  | TG | 1.26 (1.05-1.5) | 1.17E-02 |  |  |  |  |
| SPINK4 | 1.23 (1.02-1.49) | 3.44E-02 |  | ARHGAP1 | 0.57 (0.37-0.88) | 1.19E-02 |  |  |  |  |
| ROBO2 | 1.78 (1.04-3.04) | 3.48E-02 |  | IFNAR1 | 2.31 (1.2-4.45) | 1.19E-02 |  |  |  |  |
| NPHS2 | 1.15 (1.01-1.32) | 3.53E-02 |  | IGSF21 | 0.43 (0.22-0.83) | 1.20E-02 |  |  |  |  |
| PEPD | 0.53 (0.3-0.96) | 3.53E-02 |  | NARS1 | 0.56 (0.35-0.88) | 1.21E-02 |  |  |  |  |
| ERBB4 | 1.78 (1.04-3.06) | 3.53E-02 |  | ENPP6 | 0.48 (0.27-0.85) | 1.21E-02 |  |  |  |  |
| CCL8 | 1.26 (1.02-1.57) | 3.57E-02 |  | PAGR1 | 0.44 (0.24-0.84) | 1.21E-02 |  |  |  |  |
| DCUN1D1 | 0.75 (0.57-0.98) | 3.59E-02 |  | SERPINF2 | 0.14 (0.03-0.66) | 1.22E-02 |  |  |  |  |
| VMO1 | 1.25 (1.01-1.54) | 3.61E-02 |  | ELOB | 1.58 (1.1-2.25) | 1.24E-02 |  |  |  |  |
| APP | 1.24 (1.01-1.51) | 3.62E-02 |  | FABP4 | 1.42 (1.08-1.88) | 1.26E-02 |  |  |  |  |
| MAP3K5 | 0.86 (0.74-0.99) | 3.63E-02 |  | CRTAP | 0.43 (0.22-0.83) | 1.27E-02 |  |  |  |  |
| IDUA | 1.37 (1.02-1.83) | 3.63E-02 |  | MARS1 | 0.7 (0.53-0.93) | 1.28E-02 |  |  |  |  |
| HSBP1 | 1.19 (1.01-1.4) | 3.70E-02 |  | MEP1B | 0.82 (0.7-0.96) | 1.28E-02 |  |  |  |  |
| LAMA1 | 1.27 (1.01-1.6) | 3.73E-02 |  | RANGAP1 | 0.51 (0.3-0.87) | 1.29E-02 |  |  |  |  |
| RTBDN | 1.57 (1.03-2.39) | 3.74E-02 |  | TMSB10 | 1.38 (1.07-1.77) | 1.29E-02 |  |  |  |  |
| TSHB | 0.83 (0.7-0.99) | 3.75E-02 |  | GTPBP2 | 0.68 (0.5-0.92) | 1.30E-02 |  |  |  |  |
| FGFBP1 | 0.66 (0.44-0.98) | 3.75E-02 |  | PTPRR | 0.46 (0.25-0.85) | 1.30E-02 |  |  |  |  |
| LRP11 | 1.45 (1.02-2.07) | 3.75E-02 |  | CEP85 | 0.65 (0.46-0.91) | 1.31E-02 |  |  |  |  |
| CHIT1 | 1.12 (1.01-1.25) | 3.76E-02 |  | CNTN5 | 0.52 (0.31-0.87) | 1.30E-02 |  |  |  |  |
| POMC | 0.79 (0.64-0.99) | 3.78E-02 |  | RNF168 | 0.4 (0.19-0.82) | 1.31E-02 |  |  |  |  |
| CD5L | 1.4 (1.02-1.94) | 3.79E-02 |  | SFRP4 | 0.5 (0.29-0.86) | 1.31E-02 |  |  |  |  |
| CD93 | 1.57 (1.02-2.39) | 3.83E-02 |  | SPINK4 | 1.41 (1.08-1.86) | 1.31E-02 |  |  |  |  |
| CFD | 1.9 (1.04-3.5) | 3.83E-02 |  | KRT18 | 1.28 (1.05-1.57) | 1.33E-02 |  |  |  |  |
| MN1 | 0.7 (0.5-0.98) | 3.88E-02 |  | PLA2G2A | 1.39 (1.07-1.8) | 1.33E-02 |  |  |  |  |
| TNN | 0.69 (0.48-0.98) | 3.89E-02 |  | TNC | 1.66 (1.11-2.48) | 1.34E-02 |  |  |  |  |
| SIGLEC10 | 1.5 (1.02-2.19) | 3.92E-02 |  | DDHD2 | 0.64 (0.45-0.91) | 1.34E-02 |  |  |  |  |
| EGLN1 | 1.22 (1.01-1.47) | 3.93E-02 |  | MEP1A | 1.43 (1.08-1.89) | 1.34E-02 |  |  |  |  |
| CEACAM20 | 1.36 (1.01-1.81) | 3.95E-02 |  | ENO3 | 0.59 (0.38-0.9) | 1.34E-02 |  |  |  |  |
| KIF1C | 1.42 (1.02-1.98) | 3.95E-02 |  | AGER | 0.55 (0.34-0.88) | 1.35E-02 |  |  |  |  |
| VAMP8 | 0.82 (0.69-0.99) | 3.96E-02 |  | FGFR2 | 2.62 (1.22-5.62) | 1.36E-02 |  |  |  |  |
| PENK | 1.57 (1.02-2.41) | 3.96E-02 |  | IGFBPL1 | 1.87 (1.14-3.09) | 1.38E-02 |  |  |  |  |
| SLC39A5 | 1.24 (1.01-1.52) | 3.97E-02 |  | IL2RB | 0.44 (0.23-0.85) | 1.40E-02 |  |  |  |  |
| GALNT2 | 0.59 (0.36-0.98) | 3.98E-02 |  | CYB5R2 | 1.57 (1.09-2.24) | 1.41E-02 |  |  |  |  |
| RLN2 | 0.81 (0.66-0.99) | 4.03E-02 |  | LELP1 | 1.32 (1.06-1.64) | 1.42E-02 |  |  |  |  |
| MICALL2 | 0.76 (0.59-0.99) | 4.09E-02 |  | TSC1 | 0.53 (0.31-0.88) | 1.44E-02 |  |  |  |  |
| RASSF2 | 1.22 (1.01-1.47) | 4.12E-02 |  | CST7 | 1.22 (1.04-1.44) | 1.46E-02 |  |  |  |  |
| IGHMBP2 | 0.71 (0.52-0.99) | 4.16E-02 |  | GPKOW | 1.72 (1.11-2.66) | 1.46E-02 |  |  |  |  |
| CCL3 | 1.17 (1.01-1.37) | 4.16E-02 |  | LRIG3 | 1.87 (1.13-3.09) | 1.46E-02 |  |  |  |  |
| ADH1B | 0.8 (0.65-0.99) | 4.16E-02 |  | NPTX1 | 1.74 (1.12-2.72) | 1.46E-02 |  |  |  |  |
| DCXR | 0.78 (0.62-0.99) | 4.17E-02 |  | PDLIM5 | 0.79 (0.66-0.96) | 1.46E-02 |  |  |  |  |
| CSDE1 | 0.85 (0.73-0.99) | 4.18E-02 |  | RGMA | 0.46 (0.25-0.86) | 1.46E-02 |  |  |  |  |
| CFI | 2.05 (1.03-4.1) | 4.18E-02 |  | STC1 | 1.62 (1.1-2.38) | 1.48E-02 |  |  |  |  |
| TIGAR | 1.32 (1.01-1.72) | 4.19E-02 |  | ACHE | 0.41 (0.2-0.84) | 1.48E-02 |  |  |  |  |
| GLA | 1.48 (1.01-2.16) | 4.23E-02 |  | ZBTB16 | 0.67 (0.49-0.93) | 1.49E-02 |  |  |  |  |
| HYAL1 | 1.77 (1.02-3.06) | 4.24E-02 |  | IL36G | 1.37 (1.06-1.77) | 1.50E-02 |  |  |  |  |
| ATOX1 | 1.22 (1.01-1.47) | 4.25E-02 |  | C9 | 1.75 (1.11-2.75) | 1.56E-02 |  |  |  |  |
| LSM8 | 0.78 (0.62-0.99) | 4.29E-02 |  | ADAM9 | 2.18 (1.16-4.11) | 1.57E-02 |  |  |  |  |
| SEZ6L | 0.63 (0.4-0.99) | 4.31E-02 |  | IKBKG | 0.77 (0.62-0.95) | 1.57E-02 |  |  |  |  |
| TTN | 0.74 (0.55-0.99) | 4.31E-02 |  | FSTL1 | 2.82 (1.22-6.55) | 1.58E-02 |  |  |  |  |
| TPK1 | 1.75 (1.02-3.01) | 4.33E-02 |  | ITGB2 | 0.42 (0.21-0.85) | 1.60E-02 |  |  |  |  |
| CRACR2A | 0.84 (0.71-1) | 4.36E-02 |  | TMPRSS15 | 1.3 (1.05-1.62) | 1.61E-02 |  |  |  |  |
| WASF1 | 0.86 (0.74-1) | 4.37E-02 |  | VEGFB | 2.39 (1.17-4.86) | 1.63E-02 |  |  |  |  |
| SDCCAG8 | 0.87 (0.76-1) | 4.44E-02 |  | SUSD5 | 0.47 (0.26-0.87) | 1.64E-02 |  |  |  |  |
| IGFBP6 | 1.46 (1.01-2.11) | 4.44E-02 |  | GP1BA | 0.46 (0.25-0.87) | 1.67E-02 |  |  |  |  |
| LHPP | 1.24 (1.01-1.53) | 4.48E-02 |  | DCUN1D1 | 0.56 (0.35-0.9) | 1.68E-02 |  |  |  |  |
| AMN | 1.32 (1.01-1.72) | 4.48E-02 |  | KAZN | 0.77 (0.62-0.95) | 1.68E-02 |  |  |  |  |
| ASS1 | 0.82 (0.67-1) | 4.52E-02 |  | BGN | 0.8 (0.67-0.96) | 1.69E-02 |  |  |  |  |
| SIRPB1 | 1.34 (1.01-1.77) | 4.52E-02 |  | GASK1A | 0.52 (0.3-0.89) | 1.71E-02 |  |  |  |  |
| ZPR1 | 1.15 (1-1.32) | 4.54E-02 |  | PRKG1 | 0.8 (0.67-0.96) | 1.73E-02 |  |  |  |  |
| LTA | 0.71 (0.51-0.99) | 4.57E-02 |  | S100A12 | 1.36 (1.06-1.75) | 1.73E-02 |  |  |  |  |
| DYNC1H1 | 0.65 (0.43-0.99) | 4.58E-02 |  | QPCT | 2.32 (1.16-4.64) | 1.75E-02 |  |  |  |  |
| PPBP | 1.21 (1-1.45) | 4.59E-02 |  | DNAJB6 | 0.71 (0.53-0.94) | 1.75E-02 |  |  |  |  |
| RNASEH2A | 0.68 (0.47-0.99) | 4.63E-02 |  | NFX1 | 0.63 (0.44-0.92) | 1.76E-02 |  |  |  |  |
| STX6 | 0.79 (0.63-1) | 4.66E-02 |  | CCL19 | 1.17 (1.03-1.32) | 1.78E-02 |  |  |  |  |
| ITGAV | 0.47 (0.22-0.99) | 4.68E-02 |  | LRRFIP1 | 0.53 (0.31-0.89) | 1.79E-02 |  |  |  |  |
| SSC4D | 0.9 (0.81-1) | 4.73E-02 |  | ITGB1 | 0.33 (0.13-0.83) | 1.79E-02 |  |  |  |  |
| PRSS27 | 1.39 (1-1.93) | 4.75E-02 |  | FCRL1 | 0.58 (0.37-0.91) | 1.80E-02 |  |  |  |  |
| GKN1 | 1.61 (1.01-2.59) | 4.75E-02 |  | HS6ST2 | 2.02 (1.13-3.61) | 1.81E-02 |  |  |  |  |
| TOP1 | 0.87 (0.76-1) | 4.80E-02 |  | RNF41 | 0.68 (0.49-0.94) | 1.81E-02 |  |  |  |  |
| CA11 | 0.55 (0.31-1) | 4.83E-02 |  | HCG22 | 1.35 (1.05-1.73) | 1.81E-02 |  |  |  |  |
| TAB2 | 0.86 (0.74-1) | 4.84E-02 |  | PEPD | 0.36 (0.15-0.84) | 1.82E-02 |  |  |  |  |
| TNFRSF21 | 1.6 (1-2.54) | 4.86E-02 |  | ARNT | 0.45 (0.23-0.87) | 1.83E-02 |  |  |  |  |
| BHLHE40 | 1.21 (1-1.47) | 4.87E-02 |  | ERBB2 | 2.43 (1.16-5.07) | 1.84E-02 |  |  |  |  |
| PTPRF | 1.57 (1-2.46) | 4.91E-02 |  | MTSS1 | 0.78 (0.63-0.96) | 1.84E-02 |  |  |  |  |
| RPS10 | 0.62 (0.39-1) | 4.91E-02 |  | EIF4G3 | 0.63 (0.42-0.92) | 1.84E-02 |  |  |  |  |
| SERPINA1 | 4.69 (1.01-21.9) | 4.91E-02 |  | MAP2K6 | 0.79 (0.65-0.96) | 1.85E-02 |  |  |  |  |
| MANSC1 | 1.61 (1-2.6) | 4.94E-02 |  | WASF1 | 0.76 (0.6-0.95) | 1.86E-02 |  |  |  |  |
|  |  |  |  | DHODH | 0.46 (0.25-0.88) | 1.88E-02 |  |  |  |  |
|  |  |  |  | TAX1BP1 | 0.61 (0.41-0.92) | 1.87E-02 |  |  |  |  |
|  |  |  |  | SOD3 | 0.45 (0.23-0.88) | 1.88E-02 |  |  |  |  |
|  |  |  |  | KAZALD1 | 0.64 (0.44-0.93) | 1.88E-02 |  |  |  |  |
|  |  |  |  | EXTL1 | 0.46 (0.24-0.88) | 1.89E-02 |  |  |  |  |
|  |  |  |  | CTLA4 | 0.39 (0.18-0.86) | 1.89E-02 |  |  |  |  |
|  |  |  |  | CDC42BPB | 0.75 (0.6-0.95) | 1.90E-02 |  |  |  |  |
|  |  |  |  | F9 | 3.28 (1.21-8.84) | 1.91E-02 |  |  |  |  |
|  |  |  |  | ITGBL1 | 1.8 (1.1-2.94) | 1.93E-02 |  |  |  |  |
|  |  |  |  | BIN2 | 0.76 (0.61-0.96) | 1.98E-02 |  |  |  |  |
|  |  |  |  | TNIP1 | 0.73 (0.56-0.95) | 1.98E-02 |  |  |  |  |
|  |  |  |  | RAPGEF2 | 0.55 (0.33-0.91) | 1.99E-02 |  |  |  |  |
|  |  |  |  | PSCA | 1.13 (1.02-1.25) | 1.99E-02 |  |  |  |  |
|  |  |  |  | ZP3 | 0.9 (0.83-0.98) | 2.00E-02 |  |  |  |  |
|  |  |  |  | EFHD1 | 1.75 (1.09-2.81) | 2.02E-02 |  |  |  |  |
|  |  |  |  | BRAP | 0.79 (0.64-0.96) | 2.02E-02 |  |  |  |  |
|  |  |  |  | CLIP2 | 0.82 (0.69-0.97) | 2.03E-02 |  |  |  |  |
|  |  |  |  | DNM1 | 0.79 (0.65-0.97) | 2.04E-02 |  |  |  |  |
|  |  |  |  | SIGLEC10 | 1.92 (1.1-3.33) | 2.07E-02 |  |  |  |  |
|  |  |  |  | FRMD4B | 0.64 (0.44-0.94) | 2.09E-02 |  |  |  |  |
|  |  |  |  | LPP | 0.65 (0.45-0.94) | 2.11E-02 |  |  |  |  |
|  |  |  |  | GGT5 | 2.37 (1.14-4.94) | 2.11E-02 |  |  |  |  |
|  |  |  |  | MAPK13 | 1.53 (1.07-2.18) | 2.12E-02 |  |  |  |  |
|  |  |  |  | CLASP1 | 0.61 (0.4-0.93) | 2.13E-02 |  |  |  |  |
|  |  |  |  | NPR1 | 0.39 (0.17-0.87) | 2.13E-02 |  |  |  |  |
|  |  |  |  | MFAP4 | 0.43 (0.21-0.88) | 2.14E-02 |  |  |  |  |
|  |  |  |  | S100A4 | 0.67 (0.48-0.94) | 2.15E-02 |  |  |  |  |
|  |  |  |  | ZNF75D | 1.36 (1.05-1.76) | 2.16E-02 |  |  |  |  |
|  |  |  |  | IMMT | 1.56 (1.07-2.28) | 2.19E-02 |  |  |  |  |
|  |  |  |  | CBLN4 | 0.49 (0.26-0.9) | 2.21E-02 |  |  |  |  |
|  |  |  |  | VAT1 | 0.3 (0.11-0.84) | 2.22E-02 |  |  |  |  |
|  |  |  |  | ATP5PO | 1.43 (1.05-1.94) | 2.25E-02 |  |  |  |  |
|  |  |  |  | CAPS | 1.22 (1.03-1.44) | 2.25E-02 |  |  |  |  |
|  |  |  |  | AXL | 0.43 (0.21-0.89) | 2.26E-02 |  |  |  |  |
|  |  |  |  | LILRB1 | 2.25 (1.12-4.51) | 2.26E-02 |  |  |  |  |
|  |  |  |  | TCN2 | 1.88 (1.09-3.23) | 2.28E-02 |  |  |  |  |
|  |  |  |  | SPTLC1 | 0.37 (0.16-0.87) | 2.29E-02 |  |  |  |  |
|  |  |  |  | AP2B1 | 0.51 (0.29-0.91) | 2.30E-02 |  |  |  |  |
|  |  |  |  | PRG2 | 1.6 (1.07-2.4) | 2.31E-02 |  |  |  |  |
|  |  |  |  | CCL18 | 1.29 (1.03-1.6) | 2.33E-02 |  |  |  |  |
|  |  |  |  | MAD1L1 | 1.39 (1.05-1.84) | 2.34E-02 |  |  |  |  |
|  |  |  |  | DTX2 | 0.56 (0.34-0.93) | 2.36E-02 |  |  |  |  |
|  |  |  |  | OGA | 0.68 (0.48-0.95) | 2.38E-02 |  |  |  |  |
|  |  |  |  | PARD3 | 0.65 (0.44-0.94) | 2.38E-02 |  |  |  |  |
|  |  |  |  | EVI2B | 1.52 (1.06-2.18) | 2.41E-02 |  |  |  |  |
|  |  |  |  | MMUT | 0.53 (0.31-0.92) | 2.42E-02 |  |  |  |  |
|  |  |  |  | EIF2AK3 | 0.62 (0.41-0.94) | 2.43E-02 |  |  |  |  |
|  |  |  |  | APOE | 0.58 (0.37-0.93) | 2.45E-02 |  |  |  |  |
|  |  |  |  | ATP5F1D | 0.48 (0.26-0.91) | 2.44E-02 |  |  |  |  |
|  |  |  |  | FABP5 | 1.36 (1.04-1.78) | 2.45E-02 |  |  |  |  |
|  |  |  |  | VWF | 1.35 (1.04-1.76) | 2.45E-02 |  |  |  |  |
|  |  |  |  | ITIH3 | 1.78 (1.08-2.96) | 2.47E-02 |  |  |  |  |
|  |  |  |  | LZTFL1 | 0.68 (0.48-0.95) | 2.48E-02 |  |  |  |  |
|  |  |  |  | MNAT1 | 0.47 (0.25-0.91) | 2.48E-02 |  |  |  |  |
|  |  |  |  | PPCDC | 0.61 (0.4-0.94) | 2.49E-02 |  |  |  |  |
|  |  |  |  | VSTM2B | 0.38 (0.17-0.89) | 2.50E-02 |  |  |  |  |
|  |  |  |  | MYO9B | 0.67 (0.47-0.95) | 2.52E-02 |  |  |  |  |
|  |  |  |  | IGF2BP3 | 0.45 (0.22-0.91) | 2.52E-02 |  |  |  |  |
|  |  |  |  | CCL24 | 1.3 (1.03-1.64) | 2.58E-02 |  |  |  |  |
|  |  |  |  | GSR | 0.33 (0.13-0.87) | 2.58E-02 |  |  |  |  |
|  |  |  |  | HYOU1 | 2.67 (1.13-6.31) | 2.58E-02 |  |  |  |  |
|  |  |  |  | SCARA5 | 1.95 (1.08-3.5) | 2.60E-02 |  |  |  |  |
|  |  |  |  | ST13 | 0.63 (0.41-0.95) | 2.60E-02 |  |  |  |  |
|  |  |  |  | TCTN3 | 2.29 (1.1-4.76) | 2.62E-02 |  |  |  |  |
|  |  |  |  | ARHGAP45 | 0.72 (0.54-0.96) | 2.62E-02 |  |  |  |  |
|  |  |  |  | CSNK1D | 0.61 (0.4-0.94) | 2.63E-02 |  |  |  |  |
|  |  |  |  | TSPAN8 | 1.28 (1.03-1.6) | 2.65E-02 |  |  |  |  |
|  |  |  |  | THY1 | 2.01 (1.08-3.73) | 2.66E-02 |  |  |  |  |
|  |  |  |  | AKR1B10 | 1.4 (1.04-1.88) | 2.67E-02 |  |  |  |  |
|  |  |  |  | CTSF | 1.71 (1.06-2.74) | 2.68E-02 |  |  |  |  |
|  |  |  |  | DAPK2 | 0.56 (0.33-0.94) | 2.68E-02 |  |  |  |  |
|  |  |  |  | SNU13 | 0.53 (0.31-0.93) | 2.68E-02 |  |  |  |  |
|  |  |  |  | LTA | 0.58 (0.36-0.94) | 2.71E-02 |  |  |  |  |
|  |  |  |  | ARFIP1 | 0.67 (0.48-0.96) | 2.72E-02 |  |  |  |  |
|  |  |  |  | CD46 | 1.94 (1.08-3.49) | 2.72E-02 |  |  |  |  |
|  |  |  |  | IL12RB1 | 1.76 (1.07-2.9) | 2.73E-02 |  |  |  |  |
|  |  |  |  | ROBO2 | 2.41 (1.1-5.25) | 2.75E-02 |  |  |  |  |
|  |  |  |  | CD58 | 0.3 (0.1-0.88) | 2.76E-02 |  |  |  |  |
|  |  |  |  | RBPMS2 | 0.77 (0.61-0.97) | 2.76E-02 |  |  |  |  |
|  |  |  |  | ACTN4 | 1.63 (1.05-2.51) | 2.78E-02 |  |  |  |  |
|  |  |  |  | PSG1 | 1.24 (1.02-1.51) | 2.79E-02 |  |  |  |  |
|  |  |  |  | MFGE8 | 1.6 (1.05-2.44) | 2.80E-02 |  |  |  |  |
|  |  |  |  | CD244 | 0.44 (0.22-0.92) | 2.81E-02 |  |  |  |  |
|  |  |  |  | SNX18 | 0.47 (0.24-0.92) | 2.85E-02 |  |  |  |  |
|  |  |  |  | TLR4 | 0.38 (0.16-0.91) | 2.90E-02 |  |  |  |  |
|  |  |  |  | REPS1 | 0.46 (0.22-0.92) | 2.96E-02 |  |  |  |  |
|  |  |  |  | FAM172A | 0.64 (0.43-0.96) | 2.97E-02 |  |  |  |  |
|  |  |  |  | PPP1R9B | 0.79 (0.64-0.98) | 2.98E-02 |  |  |  |  |
|  |  |  |  | CALCB | 1.59 (1.05-2.41) | 2.98E-02 |  |  |  |  |
|  |  |  |  | RYR1 | 0.37 (0.15-0.91) | 2.98E-02 |  |  |  |  |
|  |  |  |  | PIBF1 | 0.71 (0.52-0.97) | 3.04E-02 |  |  |  |  |
|  |  |  |  | SELL | 0.36 (0.15-0.91) | 3.04E-02 |  |  |  |  |
|  |  |  |  | F3 | 2.09 (1.07-4.1) | 3.07E-02 |  |  |  |  |
|  |  |  |  | NAGK | 0.63 (0.41-0.96) | 3.08E-02 |  |  |  |  |
|  |  |  |  | NFKB1 | 0.7 (0.5-0.97) | 3.08E-02 |  |  |  |  |
|  |  |  |  | PTRHD1 | 0.65 (0.44-0.96) | 3.07E-02 |  |  |  |  |
|  |  |  |  | SIGLEC8 | 1.7 (1.05-2.75) | 3.10E-02 |  |  |  |  |
|  |  |  |  | AZU1 | 1.26 (1.02-1.56) | 3.13E-02 |  |  |  |  |
|  |  |  |  | SEL1L | 1.61 (1.04-2.47) | 3.13E-02 |  |  |  |  |
|  |  |  |  | TMEM132A | 0.66 (0.45-0.96) | 3.12E-02 |  |  |  |  |
|  |  |  |  | TTF2 | 0.56 (0.33-0.95) | 3.12E-02 |  |  |  |  |
|  |  |  |  | SLC39A14 | 1.64 (1.05-2.59) | 3.14E-02 |  |  |  |  |
|  |  |  |  | TMPRSS11B | 0.62 (0.41-0.96) | 3.16E-02 |  |  |  |  |
|  |  |  |  | IRAG2 | 0.75 (0.58-0.98) | 3.18E-02 |  |  |  |  |
|  |  |  |  | VEGFD | 1.8 (1.05-3.07) | 3.18E-02 |  |  |  |  |
|  |  |  |  | CCL23 | 1.64 (1.04-2.57) | 3.20E-02 |  |  |  |  |
|  |  |  |  | SMOC1 | 1.8 (1.05-3.07) | 3.21E-02 |  |  |  |  |
|  |  |  |  | CREB3 | 1.41 (1.03-1.93) | 3.22E-02 |  |  |  |  |
|  |  |  |  | SV2A | 0.61 (0.39-0.96) | 3.23E-02 |  |  |  |  |
|  |  |  |  | GKN1 | 2.05 (1.06-3.94) | 3.23E-02 |  |  |  |  |
|  |  |  |  | EEF1D | 0.75 (0.58-0.98) | 3.25E-02 |  |  |  |  |
|  |  |  |  | SNCG | 1.35 (1.03-1.78) | 3.26E-02 |  |  |  |  |
|  |  |  |  | DDAH1 | 1.53 (1.04-2.27) | 3.27E-02 |  |  |  |  |
|  |  |  |  | CABP2 | 0.61 (0.39-0.96) | 3.27E-02 |  |  |  |  |
|  |  |  |  | CHMP6 | 0.64 (0.42-0.96) | 3.28E-02 |  |  |  |  |
|  |  |  |  | DBN1 | 0.58 (0.35-0.96) | 3.29E-02 |  |  |  |  |
|  |  |  |  | VCPKMT | 0.65 (0.43-0.97) | 3.30E-02 |  |  |  |  |
|  |  |  |  | PTPRK | 2.14 (1.06-4.32) | 3.31E-02 |  |  |  |  |
|  |  |  |  | RNASEH2A | 0.51 (0.28-0.95) | 3.33E-02 |  |  |  |  |
|  |  |  |  | TIMM8A | 0.74 (0.56-0.98) | 3.33E-02 |  |  |  |  |
|  |  |  |  | LACRT | 0.5 (0.26-0.95) | 3.38E-02 |  |  |  |  |
|  |  |  |  | CALB2 | 1.5 (1.03-2.17) | 3.39E-02 |  |  |  |  |
|  |  |  |  | IL12A_IL12B | 0.76 (0.58-0.98) | 3.40E-02 |  |  |  |  |
|  |  |  |  | ALCAM | 2.7 (1.08-6.79) | 3.44E-02 |  |  |  |  |
|  |  |  |  | MYH4 | 1.45 (1.03-2.06) | 3.45E-02 |  |  |  |  |
|  |  |  |  | SKAP1 | 1.32 (1.02-1.71) | 3.45E-02 |  |  |  |  |
|  |  |  |  | PALM3 | 0.55 (0.32-0.96) | 3.47E-02 |  |  |  |  |
|  |  |  |  | CES2 | 1.41 (1.02-1.93) | 3.52E-02 |  |  |  |  |
|  |  |  |  | FAP | 0.47 (0.23-0.95) | 3.52E-02 |  |  |  |  |
|  |  |  |  | GTF2IRD1 | 1.28 (1.02-1.61) | 3.55E-02 |  |  |  |  |
|  |  |  |  | BLOC1S3 | 0.5 (0.26-0.95) | 3.55E-02 |  |  |  |  |
|  |  |  |  | SMS | 0.6 (0.37-0.97) | 3.58E-02 |  |  |  |  |
|  |  |  |  | EIF2S2 | 0.48 (0.24-0.95) | 3.60E-02 |  |  |  |  |
|  |  |  |  | PALM2 | 1.67 (1.03-2.7) | 3.61E-02 |  |  |  |  |
|  |  |  |  | LRPAP1 | 1.35 (1.02-1.79) | 3.62E-02 |  |  |  |  |
|  |  |  |  | TNFSF8 | 1.86 (1.04-3.31) | 3.64E-02 |  |  |  |  |
|  |  |  |  | ANKRD54 | 0.64 (0.42-0.97) | 3.68E-02 |  |  |  |  |
|  |  |  |  | VPS4B | 0.76 (0.59-0.98) | 3.70E-02 |  |  |  |  |
|  |  |  |  | CRIM1 | 2.53 (1.06-6.06) | 3.72E-02 |  |  |  |  |
|  |  |  |  | ANXA11 | 0.75 (0.57-0.98) | 3.80E-02 |  |  |  |  |
|  |  |  |  | ATXN2L | 0.54 (0.3-0.97) | 3.81E-02 |  |  |  |  |
|  |  |  |  | ADAMTS16 | 1.51 (1.02-2.23) | 3.83E-02 |  |  |  |  |
|  |  |  |  | FARSA | 0.65 (0.43-0.98) | 3.83E-02 |  |  |  |  |
|  |  |  |  | FAM3D | 1.33 (1.02-1.74) | 3.85E-02 |  |  |  |  |
|  |  |  |  | UBE2Z | 0.58 (0.35-0.97) | 3.86E-02 |  |  |  |  |
|  |  |  |  | TSHB | 0.76 (0.59-0.99) | 3.89E-02 |  |  |  |  |
|  |  |  |  | NIT1 | 0.63 (0.4-0.98) | 3.91E-02 |  |  |  |  |
|  |  |  |  | AKT2 | 0.83 (0.69-0.99) | 3.92E-02 |  |  |  |  |
|  |  |  |  | GGA1 | 0.66 (0.44-0.98) | 3.92E-02 |  |  |  |  |
|  |  |  |  | KLK8 | 1.73 (1.03-2.9) | 3.93E-02 |  |  |  |  |
|  |  |  |  | POLR2F | 1.53 (1.02-2.31) | 3.94E-02 |  |  |  |  |
|  |  |  |  | HSPB1 | 0.76 (0.58-0.99) | 3.99E-02 |  |  |  |  |
|  |  |  |  | RNASE10 | 1.49 (1.02-2.17) | 3.99E-02 |  |  |  |  |
|  |  |  |  | GLI2 | 0.39 (0.16-0.96) | 4.00E-02 |  |  |  |  |
|  |  |  |  | SMAD3 | 0.6 (0.37-0.98) | 4.01E-02 |  |  |  |  |
|  |  |  |  | PENK | 1.82 (1.03-3.22) | 4.02E-02 |  |  |  |  |
|  |  |  |  | CDH15 | 1.3 (1.01-1.67) | 4.03E-02 |  |  |  |  |
|  |  |  |  | DSG2 | 0.47 (0.23-0.97) | 4.03E-02 |  |  |  |  |
|  |  |  |  | LCN15 | 1.34 (1.01-1.78) | 4.04E-02 |  |  |  |  |
|  |  |  |  | CD276 | 1.78 (1.02-3.09) | 4.06E-02 |  |  |  |  |
|  |  |  |  | LMOD1 | 1.63 (1.02-2.61) | 4.07E-02 |  |  |  |  |
|  |  |  |  | IQGAP2 | 0.67 (0.46-0.98) | 4.08E-02 |  |  |  |  |
|  |  |  |  | NUBP1 | 0.74 (0.56-0.99) | 4.14E-02 |  |  |  |  |
|  |  |  |  | EIF2AK2 | 0.8 (0.64-0.99) | 4.16E-02 |  |  |  |  |
|  |  |  |  | DTD1 | 0.75 (0.57-0.99) | 4.17E-02 |  |  |  |  |
|  |  |  |  | RAB27B | 0.76 (0.59-0.99) | 4.23E-02 |  |  |  |  |
|  |  |  |  | DCTN1 | 0.73 (0.54-0.99) | 4.24E-02 |  |  |  |  |
|  |  |  |  | CRELD1 | 1.82 (1.02-3.25) | 4.29E-02 |  |  |  |  |
|  |  |  |  | LARP1 | 0.64 (0.41-0.99) | 4.28E-02 |  |  |  |  |
|  |  |  |  | LBP | 1.4 (1.01-1.94) | 4.28E-02 |  |  |  |  |
|  |  |  |  | NAA10 | 0.62 (0.39-0.98) | 4.29E-02 |  |  |  |  |
|  |  |  |  | ADA | 1.65 (1.02-2.68) | 4.33E-02 |  |  |  |  |
|  |  |  |  | KYNU | 1.5 (1.01-2.22) | 4.33E-02 |  |  |  |  |
|  |  |  |  | NEDD4L | 0.64 (0.42-0.99) | 4.32E-02 |  |  |  |  |
|  |  |  |  | SPINT3 | 1.09 (1-1.18) | 4.33E-02 |  |  |  |  |
|  |  |  |  | DBH | 0.84 (0.71-0.99) | 4.35E-02 |  |  |  |  |
|  |  |  |  | GDNF | 1.6 (1.01-2.54) | 4.35E-02 |  |  |  |  |
|  |  |  |  | DCC | 0.27 (0.07-0.97) | 4.40E-02 |  |  |  |  |
|  |  |  |  | NTproBNP | 1.19 (1-1.4) | 4.40E-02 |  |  |  |  |
|  |  |  |  | SPRED2 | 0.52 (0.28-0.98) | 4.40E-02 |  |  |  |  |
|  |  |  |  | SCARF1 | 1.58 (1.01-2.46) | 4.42E-02 |  |  |  |  |
|  |  |  |  | STXBP3 | 0.57 (0.33-0.99) | 4.43E-02 |  |  |  |  |
|  |  |  |  | HIP1R | 1.44 (1.01-2.05) | 4.44E-02 |  |  |  |  |
|  |  |  |  | LRRC25 | 1.55 (1.01-2.37) | 4.45E-02 |  |  |  |  |
|  |  |  |  | FUS | 1.57 (1.01-2.44) | 4.46E-02 |  |  |  |  |
|  |  |  |  | PKD1 | 2.29 (1.02-5.12) | 4.47E-02 |  |  |  |  |
|  |  |  |  | AMY2B | 1.52 (1.01-2.3) | 4.49E-02 |  |  |  |  |
|  |  |  |  | HS3ST3B1 | 1.7 (1.01-2.86) | 4.50E-02 |  |  |  |  |
|  |  |  |  | NCS1 | 1.9 (1.01-3.57) | 4.52E-02 |  |  |  |  |
|  |  |  |  | EDAR | 0.79 (0.63-1) | 4.53E-02 |  |  |  |  |
|  |  |  |  | MIF | 0.75 (0.56-0.99) | 4.52E-02 |  |  |  |  |
|  |  |  |  | SAMD9L | 0.75 (0.56-0.99) | 4.54E-02 |  |  |  |  |
|  |  |  |  | PDZD2 | 0.62 (0.39-0.99) | 4.55E-02 |  |  |  |  |
|  |  |  |  | NDUFS6 | 1.22 (1-1.48) | 4.56E-02 |  |  |  |  |
|  |  |  |  | IKZF2 | 0.72 (0.52-0.99) | 4.59E-02 |  |  |  |  |
|  |  |  |  | IGSF8 | 1.82 (1.01-3.27) | 4.64E-02 |  |  |  |  |
|  |  |  |  | RAB37 | 0.73 (0.54-1) | 4.65E-02 |  |  |  |  |
|  |  |  |  | XIAP | 0.64 (0.41-0.99) | 4.65E-02 |  |  |  |  |
|  |  |  |  | DCTD | 0.78 (0.61-1) | 4.67E-02 |  |  |  |  |
|  |  |  |  | MET | 0.38 (0.14-0.99) | 4.67E-02 |  |  |  |  |
|  |  |  |  | VPS37A | 0.77 (0.59-1) | 4.67E-02 |  |  |  |  |
|  |  |  |  | CLC | 1.3 (1-1.68) | 4.69E-02 |  |  |  |  |
|  |  |  |  | KDM3A | 0.65 (0.43-0.99) | 4.69E-02 |  |  |  |  |
|  |  |  |  | ITIH4 | 2.04 (1.01-4.13) | 4.70E-02 |  |  |  |  |
|  |  |  |  | CD70 | 1.57 (1-2.44) | 4.75E-02 |  |  |  |  |
|  |  |  |  | PFKFB2 | 0.77 (0.6-1) | 4.75E-02 |  |  |  |  |
|  |  |  |  | DARS1 | 0.76 (0.58-1) | 4.78E-02 |  |  |  |  |
|  |  |  |  | RICTOR | 0.48 (0.23-0.99) | 4.79E-02 |  |  |  |  |
|  |  |  |  | S100A11 | 1.38 (1-1.9) | 4.79E-02 |  |  |  |  |
|  |  |  |  | VSIG10L | 0.44 (0.2-0.99) | 4.80E-02 |  |  |  |  |
|  |  |  |  | IL13 | 0.53 (0.28-0.99) | 4.82E-02 |  |  |  |  |
|  |  |  |  | ADRA2A | 0.62 (0.39-1) | 4.85E-02 |  |  |  |  |
|  |  |  |  | VAV3 | 0.78 (0.61-1) | 4.85E-02 |  |  |  |  |
|  |  |  |  | MATN3 | 1.79 (1-3.19) | 4.92E-02 |  |  |  |  |
|  |  |  |  | SNX15 | 0.65 (0.42-1) | 4.95E-02 |  |  |  |  |
|  |  |  |  | C1QTNF1 | 1.52 (1-2.31) | 4.96E-02 |  |  |  |  |
|  |  |  |  | NT5C3A | 0.8 (0.64-1) | 4.96E-02 |  |  |  |  |
|  |  |  |  | GHRL | 1.26 (1-1.6) | 4.97E-02 |  |  |  |  |

| **Supplementary table 5. Multivariate logistic results for lung adenocarcinoma, squamous cell carcinoma, and small cell carcinoma.** | | | | | | | | | | |
| --- | --- | --- | --- | --- | --- | --- | --- | --- | --- | --- |
| **Protein** | **Adenocarcinoma** | |  | **Protein** | **Squamous Cell Carcinoma** | |  | **Protein** | **Small Cell Carcinoma** | |
|  | **OR (95%CI)** | ***P*** |  |  | **OR (95%CI)** | ***P*** |  |  | **OR (95%CI)** | ***P*** |
| CXCL17 | 2.11 (1.68-2.63) | 5.99E-11 |  | CEACAM6 | 2.27 (1.66-3.1) | 2.66E-07 |  | SERPINA3 | 9.15 (3.92-21.34) | 3.01E-07 |
| CEACAM5 | 1.62 (1.36-1.92) | 3.06E-08 |  | CXCL17 | 2.21 (1.61-3.04) | 1.00E-06 |  | CEACAM6 | 2.42 (1.71-3.43) | 6.78E-07 |
| WFDC2 | 2 (1.55-2.58) | 1.09E-07 |  | CDCP1 | 2.12 (1.56-2.9) | 1.89E-06 |  | SPINT1 | 4.55 (2.45-8.45) | 1.63E-06 |
| CEACAM6 | 1.84 (1.43-2.37) | 2.37E-06 |  | FUT3_FUT5 | 2.56 (1.71-3.82) | 4.33E-06 |  | SFTPD | 2.2 (1.54-3.14) | 1.39E-05 |
| ACHE | 0.34 (0.21-0.55) | 1.56E-05 |  | WFDC2 | 2.27 (1.6-3.22) | 4.61E-06 |  | JMJD1C | 1.81 (1.37-2.39) | 3.10E-05 |
| MMP9 | 1.61 (1.29-2) | 2.37E-05 |  | GDF15 | 1.99 (1.47-2.68) | 7.07E-06 |  | PRSS8 | 2.87 (1.73-4.74) | 4.01E-05 |
| LAMP3 | 1.64 (1.3-2.08) | 3.56E-05 |  | MMP12 | 1.89 (1.39-2.56) | 4.30E-05 |  | PSCA | 0.82 (0.74-0.9) | 8.19E-05 |
| CSPG4 | 0.4 (0.26-0.62) | 3.64E-05 |  | RAB6A | 0.42 (0.28-0.64) | 5.73E-05 |  | HEPH | 7.1 (2.67-18.85) | 8.33E-05 |
| SCGB3A1 | 3.05 (1.79-5.2) | 4.01E-05 |  | KRT19 | 1.61 (1.27-2.05) | 9.27E-05 |  | CEACAM5 | 1.73 (1.31-2.28) | 1.05E-04 |
| SLITRK2 | 0.5 (0.36-0.7) | 5.25E-05 |  | SUSD5 | 0.26 (0.13-0.52) | 1.02E-04 |  | NHLRC3 | 5.4 (2.3-12.69) | 1.11E-04 |
| PIGR | 1.91 (1.38-2.63) | 7.64E-05 |  | CEACAM5 | 1.62 (1.26-2.07) | 1.50E-04 |  | LAMP3 | 2.29 (1.49-3.54) | 1.71E-04 |
| SFTPA2 | 1.52 (1.23-1.87) | 8.14E-05 |  | CA9 | 1.91 (1.35-2.69) | 2.49E-04 |  | HLA.DRA | 2.96 (1.67-5.22) | 1.89E-04 |
| OSM | 1.44 (1.2-1.74) | 9.17E-05 |  | SPINT1 | 3 (1.64-5.51) | 3.87E-04 |  | PLAU | 2.3 (1.48-3.58) | 2.17E-04 |
| SFTPD | 1.46 (1.19-1.78) | 2.12E-04 |  | CLEC5A | 2.75 (1.57-4.81) | 3.99E-04 |  | LRG1 | 4.37 (1.99-9.6) | 2.38E-04 |
| TNR | 0.56 (0.41-0.76) | 2.34E-04 |  | PLAUR | 2.85 (1.59-5.09) | 4.20E-04 |  | CCL18 | 1.53 (1.22-1.93) | 2.38E-04 |
| DKK4 | 0.55 (0.4-0.77) | 3.39E-04 |  | MSLN | 1.64 (1.24-2.16) | 4.62E-04 |  | QSOX1 | 1.75 (1.3-2.35) | 2.44E-04 |
| IFNLR1 | 1.66 (1.26-2.2) | 3.80E-04 |  | IGFBP3 | 0.4 (0.24-0.67) | 5.13E-04 |  | CXCL17 | 2.05 (1.4-3.01) | 2.53E-04 |
| ITIH3 | 1.94 (1.34-2.8) | 4.38E-04 |  | TNFRSF6B | 1.65 (1.24-2.19) | 5.98E-04 |  | DPP10 | 2.33 (1.47-3.71) | 3.41E-04 |
| PROK1 | 1.46 (1.18-1.81) | 5.08E-04 |  | FCAR | 1.86 (1.31-2.66) | 6.11E-04 |  | SERPINA1 | 22.29 (3.95-125.75) | 4.38E-04 |
| RGMA | 0.47 (0.3-0.72) | 5.26E-04 |  | TNFSF13B | 2.64 (1.51-4.62) | 6.46E-04 |  | TNFSF13B | 2.85 (1.59-5.12) | 4.46E-04 |
| DDC | 0.61 (0.46-0.81) | 6.01E-04 |  | MZB1 | 1.78 (1.27-2.5) | 7.78E-04 |  | CCL22 | 1.93 (1.33-2.8) | 5.21E-04 |
| BGN | 0.8 (0.71-0.91) | 6.59E-04 |  | LRIG1 | 2.47 (1.45-4.19) | 8.11E-04 |  | BHMT2 | 0.18 (0.07-0.47) | 5.74E-04 |
| APLP1 | 0.63 (0.49-0.82) | 6.63E-04 |  | JCHAIN | 1.66 (1.23-2.23) | 8.69E-04 |  | CD80 | 3.34 (1.68-6.64) | 5.75E-04 |
| PLAUR | 2.02 (1.33-3.07) | 9.64E-04 |  | COL24A1 | 2.11 (1.36-3.28) | 8.92E-04 |  | LECT2 | 1.79 (1.28-2.51) | 6.97E-04 |
| CDA | 0.55 (0.39-0.79) | 1.00E-03 |  | UNC79 | 0.43 (0.26-0.71) | 9.42E-04 |  | IL18RAP | 1.23 (1.09-1.39) | 7.99E-04 |
| OLR1 | 1.38 (1.14-1.67) | 1.13E-03 |  | AREG | 1.83 (1.28-2.62) | 9.55E-04 |  | GRP | 2.11 (1.36-3.27) | 9.27E-04 |
| CAPG | 1.41 (1.14-1.74) | 1.65E-03 |  | PIGR | 2.19 (1.37-3.49) | 1.03E-03 |  | CPXM2 | 3.13 (1.59-6.18) | 1.01E-03 |
| ALPP | 1.18 (1.06-1.31) | 1.71E-03 |  | SFTPA2 | 1.62 (1.21-2.16) | 1.10E-03 |  | FASLG | 0.38 (0.21-0.68) | 1.09E-03 |
| DCN | 0.38 (0.2-0.7) | 1.91E-03 |  | PLAU | 2.01 (1.32-3.06) | 1.11E-03 |  | GDF15 | 1.86 (1.28-2.72) | 1.25E-03 |
| MYOM3 | 0.76 (0.64-0.91) | 2.49E-03 |  | LTA4H | 1.58 (1.2-2.09) | 1.23E-03 |  | WFDC2 | 2.08 (1.33-3.26) | 1.25E-03 |
| RSPO1 | 1.77 (1.22-2.58) | 2.54E-03 |  | EDA2R | 2.03 (1.32-3.12) | 1.27E-03 |  | CRELD1 | 2.9 (1.51-5.57) | 1.33E-03 |
| CEACAM8 | 1.44 (1.14-1.83) | 2.59E-03 |  | CD80 | 2.63 (1.45-4.77) | 1.42E-03 |  | FAM20A | 3.03 (1.54-5.96) | 1.37E-03 |
| ZBTB16 | 0.72 (0.59-0.89) | 2.64E-03 |  | TNFRSF10B | 1.46 (1.16-1.85) | 1.52E-03 |  | LTBP2 | 2.82 (1.49-5.32) | 1.39E-03 |
| FCN1 | 1.6 (1.18-2.17) | 2.73E-03 |  | APOC1 | 0.37 (0.2-0.69) | 1.54E-03 |  | LTA4H | 1.7 (1.23-2.36) | 1.40E-03 |
| IL22 | 1.33 (1.11-1.61) | 2.73E-03 |  | GPR15L | 1.72 (1.23-2.42) | 1.59E-03 |  | CDCP1 | 1.93 (1.29-2.89) | 1.48E-03 |
| MAN2B2 | 0.67 (0.52-0.87) | 2.76E-03 |  | SOX2 | 3.13 (1.54-6.36) | 1.64E-03 |  | ALPP | 1.33 (1.12-1.6) | 1.65E-03 |
| SUSD5 | 0.5 (0.32-0.79) | 2.92E-03 |  | GALNT5 | 2.64 (1.44-4.82) | 1.65E-03 |  | HSD11B1 | 0.4 (0.23-0.71) | 1.67E-03 |
| MYL3 | 0.71 (0.56-0.89) | 3.36E-03 |  | VWC2 | 2.13 (1.33-3.41) | 1.72E-03 |  | HEG1 | 4.14 (1.67-10.25) | 2.11E-03 |
| BLNK | 1.58 (1.16-2.15) | 3.41E-03 |  | IFI30 | 2.11 (1.32-3.36) | 1.77E-03 |  | PROCR | 0.19 (0.06-0.54) | 2.13E-03 |
| TGFA | 1.45 (1.13-1.86) | 3.56E-03 |  | TIGIT | 1.94 (1.28-2.95) | 1.85E-03 |  | TNFRSF10A | 2.33 (1.34-4.02) | 2.55E-03 |
| CDH1 | 0.55 (0.37-0.82) | 3.60E-03 |  | TRIM25 | 0.72 (0.58-0.88) | 1.86E-03 |  | LYSMD3 | 1.34 (1.11-1.62) | 2.71E-03 |
| CLEC4G | 1.79 (1.21-2.67) | 3.96E-03 |  | CXCL9 | 1.49 (1.16-1.92) | 1.94E-03 |  | PRAME | 0.19 (0.06-0.56) | 2.79E-03 |
| PTGR1 | 0.75 (0.62-0.91) | 4.13E-03 |  | APOA1 | 0.22 (0.09-0.58) | 2.06E-03 |  | IGSF3 | 2.13 (1.3-3.49) | 2.83E-03 |
| MMP8 | 1.28 (1.08-1.52) | 4.40E-03 |  | SIGLEC15 | 1.36 (1.12-1.65) | 2.07E-03 |  | KITLG | 0.41 (0.23-0.74) | 2.95E-03 |
| SDHB | 0.66 (0.49-0.88) | 4.58E-03 |  | CEACAM19 | 2.04 (1.29-3.23) | 2.15E-03 |  | C9 | 2.33 (1.33-4.08) | 2.98E-03 |
| CCL28 | 1.34 (1.09-1.65) | 5.07E-03 |  | VSIG2 | 1.54 (1.17-2.04) | 2.21E-03 |  | DEFB116 | 1.44 (1.13-1.83) | 3.12E-03 |
| NCAM1 | 0.53 (0.34-0.83) | 5.07E-03 |  | SUGP1 | 0.34 (0.17-0.68) | 2.30E-03 |  | ITGA5 | 3.79 (1.57-9.18) | 3.12E-03 |
| SYNGAP1 | 1.35 (1.09-1.66) | 5.08E-03 |  | CAPG | 1.6 (1.18-2.16) | 2.34E-03 |  | MANSC4 | 1.68 (1.19-2.38) | 3.36E-03 |
| SCGB1A1 | 0.75 (0.61-0.92) | 5.21E-03 |  | TRAF2 | 0.63 (0.47-0.85) | 2.36E-03 |  | SCGB3A1 | 3.98 (1.58-10.04) | 3.47E-03 |
| IL6 | 1.22 (1.06-1.4) | 5.39E-03 |  | TRIM21 | 0.71 (0.57-0.89) | 2.45E-03 |  | TREM2 | 1.83 (1.22-2.76) | 3.71E-03 |
| CSF2RB | 0.6 (0.42-0.87) | 6.17E-03 |  | CLEC4D | 1.58 (1.18-2.13) | 2.47E-03 |  | TUBB3 | 0.22 (0.08-0.62) | 3.88E-03 |
| NTRK3 | 0.42 (0.23-0.78) | 6.21E-03 |  | MAG | 0.57 (0.39-0.82) | 2.52E-03 |  | DUSP3 | 1.34 (1.1-1.63) | 4.26E-03 |
| MMP12 | 1.37 (1.09-1.71) | 6.26E-03 |  | PON1 | 0.37 (0.19-0.71) | 2.64E-03 |  | PSMA1 | 1.74 (1.19-2.54) | 4.31E-03 |
| SDK2 | 1.61 (1.14-2.26) | 6.33E-03 |  | SNAPIN | 0.52 (0.34-0.8) | 2.67E-03 |  | CD3G | 1.33 (1.09-1.61) | 4.37E-03 |
| CCL26 | 1.18 (1.05-1.32) | 6.34E-03 |  | TG | 1.32 (1.1-1.59) | 2.72E-03 |  | PIGR | 2.24 (1.28-3.9) | 4.52E-03 |
| S100A11 | 1.36 (1.09-1.69) | 6.51E-03 |  | IL10 | 1.32 (1.1-1.58) | 2.84E-03 |  | FGA | 3.14 (1.43-6.93) | 4.53E-03 |
| CFHR5 | 1.71 (1.16-2.51) | 6.60E-03 |  | PAGR1 | 0.38 (0.2-0.73) | 3.51E-03 |  | ORM1 | 5.68 (1.71-18.85) | 4.53E-03 |
| PGLYRP1 | 1.48 (1.11-1.96) | 6.76E-03 |  | NEXN | 0.58 (0.4-0.84) | 3.59E-03 |  | TNFRSF11B | 2.7 (1.36-5.35) | 4.55E-03 |
| ADGRE2 | 0.52 (0.32-0.83) | 6.81E-03 |  | CHIT1 | 1.38 (1.11-1.71) | 3.62E-03 |  | SUSD2 | 2.29 (1.29-4.07) | 4.68E-03 |
| SFTPA1 | 1.3 (1.07-1.57) | 6.87E-03 |  | RABEP1 | 0.63 (0.46-0.86) | 3.64E-03 |  | ANGPT2 | 2.18 (1.26-3.76) | 5.01E-03 |
| CDCP1 | 1.38 (1.09-1.75) | 6.93E-03 |  | SDCCAG8 | 0.74 (0.61-0.91) | 3.65E-03 |  | ADGRD1 | 2.38 (1.3-4.35) | 5.03E-03 |
| PTPRB | 1.94 (1.2-3.13) | 6.93E-03 |  | TNFRSF4 | 2.01 (1.25-3.21) | 3.69E-03 |  | GBP2 | 1.59 (1.15-2.19) | 5.05E-03 |
| SLMAP | 0.76 (0.62-0.93) | 7.00E-03 |  | PRR4 | 1.56 (1.15-2.11) | 3.77E-03 |  | GAD1 | 0.21 (0.07-0.63) | 5.38E-03 |
| CD22 | 0.64 (0.47-0.89) | 7.15E-03 |  | TAX1BP1 | 0.53 (0.35-0.82) | 3.79E-03 |  | C1RL | 8.04 (1.85-34.91) | 5.39E-03 |
| GDF15 | 1.41 (1.1-1.81) | 7.16E-03 |  | SCARB2 | 2.04 (1.26-3.31) | 3.81E-03 |  | PTPRB | 2.87 (1.36-6.05) | 5.48E-03 |
| PCDH9 | 0.58 (0.39-0.86) | 7.70E-03 |  | KLK4 | 1.46 (1.13-1.89) | 3.81E-03 |  | IL17RB | 0.51 (0.31-0.82) | 6.07E-03 |
| ENPEP | 1.39 (1.09-1.77) | 7.81E-03 |  | HADH | 1.35 (1.1-1.66) | 4.24E-03 |  | SLC34A3 | 1.68 (1.16-2.45) | 6.48E-03 |
| MSTN | 0.71 (0.55-0.91) | 7.87E-03 |  | ACAN | 0.36 (0.18-0.72) | 4.25E-03 |  | FLT3LG | 2.64 (1.31-5.32) | 6.53E-03 |
| LRG1 | 2.09 (1.21-3.6) | 7.92E-03 |  | C1QA | 3.3 (1.45-7.46) | 4.25E-03 |  | BMPER | 2.69 (1.32-5.51) | 6.64E-03 |
| CA5A | 0.8 (0.68-0.95) | 8.27E-03 |  | SMPD1 | 1.66 (1.17-2.35) | 4.53E-03 |  | VNN2 | 2.31 (1.26-4.24) | 6.65E-03 |
| HEPACAM2 | 0.61 (0.42-0.88) | 8.63E-03 |  | ANGPT2 | 1.93 (1.23-3.06) | 4.64E-03 |  | SPARCL1 | 0.29 (0.12-0.71) | 6.76E-03 |
| VAMP5 | 0.65 (0.47-0.9) | 8.72E-03 |  | TRIM58 | 0.68 (0.52-0.89) | 4.71E-03 |  | SMAD3 | 0.44 (0.24-0.8) | 6.88E-03 |
| ADGRE5 | 0.54 (0.34-0.86) | 8.86E-03 |  | GNPDA2 | 0.39 (0.2-0.75) | 4.84E-03 |  | CA6 | 0.58 (0.39-0.86) | 6.95E-03 |
| EIF2AK3 | 0.69 (0.53-0.91) | 9.14E-03 |  | AMY2B | 1.73 (1.18-2.54) | 4.85E-03 |  | MSR1 | 1.99 (1.21-3.28) | 7.00E-03 |
| CNTN2 | 0.66 (0.48-0.9) | 9.23E-03 |  | HLA.A | 2.83 (1.37-5.83) | 4.87E-03 |  | CX3CL1 | 0.34 (0.16-0.75) | 7.00E-03 |
| CCL7 | 1.3 (1.07-1.58) | 9.44E-03 |  | CALCOCO1 | 0.76 (0.62-0.92) | 4.92E-03 |  | NPC2 | 2.26 (1.24-4.11) | 7.48E-03 |
| DPP6 | 0.59 (0.4-0.88) | 9.74E-03 |  | APPL2 | 0.75 (0.62-0.92) | 4.99E-03 |  | CFB | 3.21 (1.36-7.59) | 7.80E-03 |
| TARM1 | 1.24 (1.05-1.46) | 1.01E-02 |  | LIPF | 1.6 (1.15-2.22) | 5.02E-03 |  | TNR | 0.47 (0.27-0.82) | 7.90E-03 |
| CD83 | 1.61 (1.12-2.31) | 1.02E-02 |  | ARAF | 0.55 (0.37-0.84) | 5.11E-03 |  | IGDCC4 | 0.33 (0.15-0.75) | 8.01E-03 |
| MEGF10 | 0.63 (0.44-0.9) | 1.02E-02 |  | ICAM5 | 1.85 (1.2-2.86) | 5.25E-03 |  | TGFBR3 | 0.42 (0.22-0.8) | 8.04E-03 |
| AGR3 | 0.72 (0.56-0.93) | 1.04E-02 |  | CRACR2A | 0.67 (0.51-0.89) | 5.33E-03 |  | CTSD | 1.77 (1.16-2.7) | 8.14E-03 |
| GNPDA1 | 1.49 (1.1-2.03) | 1.05E-02 |  | ALPP | 1.24 (1.07-1.44) | 5.34E-03 |  | SH3GL3 | 0.71 (0.56-0.92) | 8.20E-03 |
| EGFL7 | 1.49 (1.1-2.02) | 1.06E-02 |  | EVI5 | 0.68 (0.52-0.89) | 5.38E-03 |  | CACNA1H | 1.49 (1.11-2) | 8.36E-03 |
| ITGAV | 0.36 (0.16-0.79) | 1.08E-02 |  | DMP1 | 0.62 (0.45-0.87) | 5.82E-03 |  | CPTP | 0.27 (0.1-0.72) | 8.43E-03 |
| DNPEP | 0.73 (0.57-0.93) | 1.08E-02 |  | IL24 | 1.52 (1.13-2.05) | 5.84E-03 |  | LYPD8 | 1.78 (1.16-2.73) | 8.44E-03 |
| CD248 | 0.61 (0.42-0.89) | 1.08E-02 |  | ADD1 | 0.63 (0.46-0.88) | 5.90E-03 |  | TGFBI | 3.14 (1.33-7.41) | 9.03E-03 |
| SERPINA4 | 0.43 (0.23-0.82) | 1.08E-02 |  | TRIM26 | 0.46 (0.27-0.8) | 6.04E-03 |  | SPON1 | 2.23 (1.22-4.07) | 9.08E-03 |
| IFNGR2 | 1.38 (1.08-1.77) | 1.08E-02 |  | SUSD2 | 2.11 (1.24-3.61) | 6.13E-03 |  | NEB | 0.27 (0.1-0.72) | 9.13E-03 |
| MSLN | 1.29 (1.06-1.57) | 1.11E-02 |  | NTRK3 | 0.28 (0.11-0.69) | 6.19E-03 |  | IDS | 0.08 (0.01-0.53) | 9.15E-03 |
| CD3G | 1.19 (1.04-1.36) | 1.13E-02 |  | AZI2 | 0.52 (0.33-0.83) | 6.25E-03 |  | MMP15 | 0.23 (0.08-0.7) | 9.33E-03 |
| INSL4 | 1.33 (1.06-1.66) | 1.19E-02 |  | NPPC | 1.58 (1.14-2.2) | 6.39E-03 |  | UGDH | 1.31 (1.07-1.61) | 9.46E-03 |
| KEL | 0.62 (0.43-0.9) | 1.24E-02 |  | TBC1D5 | 0.67 (0.5-0.89) | 6.69E-03 |  | GALNT5 | 2.61 (1.26-5.38) | 9.51E-03 |
| BCAN | 0.62 (0.42-0.9) | 1.26E-02 |  | MAP3K5 | 0.72 (0.57-0.91) | 6.84E-03 |  | ICAM1 | 2.59 (1.26-5.32) | 9.68E-03 |
| CEACAM3 | 1.38 (1.07-1.77) | 1.27E-02 |  | BAG6 | 0.45 (0.25-0.8) | 6.85E-03 |  | IFNG | 0.79 (0.66-0.94) | 9.75E-03 |
| SORD | 0.78 (0.65-0.95) | 1.28E-02 |  | HMGCS1 | 0.42 (0.22-0.79) | 6.85E-03 |  | CBLN4 | 2.31 (1.22-4.37) | 1.01E-02 |
| NELL2 | 0.55 (0.34-0.88) | 1.31E-02 |  | RGCC | 0.66 (0.49-0.89) | 6.95E-03 |  | TCP11 | 1.47 (1.1-1.98) | 1.03E-02 |
| SLITRK1 | 0.55 (0.34-0.88) | 1.35E-02 |  | SFTPD | 1.5 (1.12-2.01) | 7.06E-03 |  | PGLYRP1 | 1.86 (1.16-2.98) | 1.03E-02 |
| DPP4 | 0.56 (0.35-0.89) | 1.36E-02 |  | DAPK2 | 0.48 (0.28-0.82) | 7.16E-03 |  | CCL17 | 1.39 (1.08-1.78) | 1.03E-02 |
| CLNS1A | 1.6 (1.1-2.32) | 1.39E-02 |  | BCAM | 2.47 (1.28-4.77) | 7.26E-03 |  | NME1 | 0.16 (0.04-0.65) | 1.04E-02 |
| SRPX | 0.6 (0.4-0.9) | 1.40E-02 |  | PRSS8 | 1.82 (1.17-2.81) | 7.38E-03 |  | TPM3 | 0.34 (0.15-0.77) | 1.04E-02 |
| IL1B | 1.29 (1.05-1.58) | 1.43E-02 |  | AMY2A | 1.71 (1.15-2.53) | 7.41E-03 |  | RCC1 | 1.59 (1.12-2.28) | 1.06E-02 |
| CCL21 | 1.39 (1.07-1.82) | 1.46E-02 |  | TNIP1 | 0.69 (0.52-0.9) | 7.41E-03 |  | ATF4 | 0.29 (0.11-0.75) | 1.08E-02 |
| GFAP | 0.69 (0.51-0.93) | 1.49E-02 |  | LAMP3 | 1.63 (1.14-2.33) | 7.54E-03 |  | FGL1 | 1.65 (1.12-2.42) | 1.11E-02 |
| IGDCC4 | 0.52 (0.31-0.88) | 1.52E-02 |  | CAMSAP1 | 0.74 (0.59-0.92) | 7.62E-03 |  | CFI | 5.71 (1.47-22.24) | 1.20E-02 |
| CASP1 | 1.23 (1.04-1.46) | 1.53E-02 |  | COMMD9 | 0.38 (0.19-0.77) | 7.72E-03 |  | MMP12 | 1.64 (1.11-2.42) | 1.24E-02 |
| MMP1 | 1.22 (1.04-1.43) | 1.53E-02 |  | ITPR1 | 1.42 (1.1-1.84) | 7.95E-03 |  | KLRB1 | 0.44 (0.23-0.85) | 1.39E-02 |
| FGFBP2 | 0.68 (0.5-0.93) | 1.54E-02 |  | EDIL3 | 1.91 (1.19-3.09) | 7.95E-03 |  | KIRREL2 | 0.45 (0.23-0.85) | 1.39E-02 |
| RNF41 | 0.77 (0.62-0.95) | 1.58E-02 |  | HEPH | 3.19 (1.35-7.52) | 8.02E-03 |  | BCAT2 | 0.38 (0.18-0.83) | 1.47E-02 |
| TACSTD2 | 0.53 (0.32-0.89) | 1.63E-02 |  | MAEA | 0.43 (0.23-0.8) | 8.06E-03 |  | NGFR | 0.25 (0.08-0.76) | 1.47E-02 |
| MDGA1 | 0.79 (0.65-0.96) | 1.69E-02 |  | LCN2 | 1.76 (1.16-2.69) | 8.37E-03 |  | ERBB2 | 3.25 (1.26-8.41) | 1.49E-02 |
| APOA4 | 0.55 (0.33-0.9) | 1.69E-02 |  | TAB2 | 0.74 (0.59-0.93) | 8.37E-03 |  | TNFRSF10B | 1.45 (1.07-1.95) | 1.51E-02 |
| PKD2 | 0.69 (0.51-0.94) | 1.70E-02 |  | ADGRG1 | 1.31 (1.07-1.6) | 8.41E-03 |  | ADAMTS16 | 1.71 (1.11-2.64) | 1.51E-02 |
| CD58 | 0.4 (0.19-0.85) | 1.71E-02 |  | RAB11FIP3 | 0.76 (0.61-0.93) | 8.48E-03 |  | HJV | 1.66 (1.1-2.5) | 1.57E-02 |
| CCL17 | 1.19 (1.03-1.37) | 1.73E-02 |  | SNX15 | 0.55 (0.35-0.86) | 8.82E-03 |  | HGF | 2.11 (1.15-3.88) | 1.58E-02 |
| HEG1 | 1.95 (1.12-3.38) | 1.74E-02 |  | CCL21 | 1.6 (1.13-2.28) | 8.91E-03 |  | SFTPA2 | 1.54 (1.08-2.18) | 1.63E-02 |
| BLMH | 0.64 (0.45-0.93) | 1.75E-02 |  | LELP1 | 1.36 (1.08-1.71) | 8.94E-03 |  | RAD51 | 1.46 (1.07-2) | 1.65E-02 |
| LAMA1 | 1.32 (1.05-1.65) | 1.75E-02 |  | CEP85 | 0.62 (0.44-0.89) | 8.98E-03 |  | IL12RB2 | 0.34 (0.14-0.82) | 1.67E-02 |
| CD80 | 1.68 (1.09-2.58) | 1.77E-02 |  | CD83 | 1.98 (1.18-3.32) | 9.18E-03 |  | CD5L | 2.03 (1.13-3.61) | 1.70E-02 |
| PPP1R9B | 0.84 (0.73-0.97) | 1.84E-02 |  | EDN1 | 2.47 (1.25-4.88) | 9.26E-03 |  | CD2 | 0.29 (0.11-0.8) | 1.71E-02 |
| GPC1 | 0.58 (0.37-0.91) | 1.85E-02 |  | IGHMBP2 | 0.48 (0.28-0.83) | 9.27E-03 |  | IL13RA1 | 3.66 (1.26-10.66) | 1.72E-02 |
| ACAN | 0.56 (0.35-0.91) | 1.85E-02 |  | MARS1 | 0.69 (0.52-0.91) | 9.40E-03 |  | INHBB | 1.72 (1.1-2.7) | 1.72E-02 |
| ENAH | 0.69 (0.51-0.94) | 1.86E-02 |  | TEX33 | 1.51 (1.11-2.06) | 9.51E-03 |  | CCAR2 | 0.32 (0.13-0.82) | 1.80E-02 |
| COMP | 0.62 (0.41-0.92) | 1.89E-02 |  | LRRFIP1 | 0.48 (0.28-0.84) | 9.68E-03 |  | DIPK2B | 2.66 (1.18-6) | 1.81E-02 |
| TREM2 | 1.33 (1.05-1.7) | 1.90E-02 |  | USP47 | 0.61 (0.42-0.89) | 1.00E-02 |  | IGSF21 | 0.37 (0.16-0.85) | 1.89E-02 |
| CLEC3B | 0.41 (0.2-0.87) | 1.93E-02 |  | DCTN2 | 0.74 (0.58-0.93) | 1.03E-02 |  | TNFSF11 | 0.62 (0.41-0.92) | 1.91E-02 |
| NPL | 1.46 (1.06-2) | 1.96E-02 |  | EFNA4 | 2.01 (1.18-3.42) | 1.04E-02 |  | TNFRSF10C | 1.88 (1.11-3.19) | 1.91E-02 |
| EDNRB | 1.38 (1.05-1.8) | 1.98E-02 |  | RNF41 | 0.64 (0.46-0.9) | 1.04E-02 |  | MSLN | 1.49 (1.07-2.07) | 1.92E-02 |
| STOML2 | 0.78 (0.63-0.96) | 2.00E-02 |  | CHAD | 0.56 (0.36-0.87) | 1.06E-02 |  | IL13RA2 | 0.32 (0.12-0.83) | 1.93E-02 |
| ADH1B | 0.76 (0.6-0.96) | 2.02E-02 |  | OPHN1 | 0.76 (0.62-0.94) | 1.06E-02 |  | IL9 | 1.47 (1.06-2.03) | 1.95E-02 |
| HGF | 1.52 (1.07-2.16) | 2.03E-02 |  | MUC16 | 0.88 (0.8-0.97) | 1.09E-02 |  | BCAT1 | 2.22 (1.14-4.34) | 1.97E-02 |
| SEZ6 | 0.53 (0.3-0.91) | 2.04E-02 |  | PTP4A3 | 1.55 (1.11-2.18) | 1.09E-02 |  | ANKRA2 | 1.61 (1.08-2.39) | 2.01E-02 |
| S100A12 | 1.23 (1.03-1.46) | 2.09E-02 |  | NARS1 | 0.55 (0.35-0.87) | 1.10E-02 |  | PTH | 0.64 (0.44-0.93) | 2.04E-02 |
| DLL4 | 1.15 (1.02-1.29) | 2.22E-02 |  | NFX1 | 0.61 (0.41-0.89) | 1.12E-02 |  | CDC123 | 1.4 (1.05-1.86) | 2.09E-02 |
| HRC | 0.54 (0.32-0.92) | 2.23E-02 |  | HCG22 | 1.38 (1.08-1.78) | 1.12E-02 |  | CTAG1A_CTAG1B | 1.59 (1.07-2.36) | 2.10E-02 |
| ITGB7 | 0.7 (0.51-0.95) | 2.31E-02 |  | DDX1 | 0.43 (0.23-0.83) | 1.13E-02 |  | GSN | 0.22 (0.06-0.8) | 2.18E-02 |
| ERI1 | 1.58 (1.06-2.35) | 2.31E-02 |  | MYH4 | 1.62 (1.11-2.36) | 1.15E-02 |  | JCHAIN | 1.55 (1.07-2.25) | 2.19E-02 |
| SV2A | 0.71 (0.54-0.96) | 2.32E-02 |  | TGFA | 1.58 (1.11-2.25) | 1.15E-02 |  | SSBP1 | 0.3 (0.11-0.84) | 2.23E-02 |
| CD72 | 0.65 (0.45-0.94) | 2.37E-02 |  | NFAT5 | 0.66 (0.47-0.91) | 1.15E-02 |  | TMOD4 | 0.41 (0.19-0.88) | 2.23E-02 |
| SEL1L | 1.49 (1.05-2.11) | 2.42E-02 |  | TIMD4 | 1.77 (1.13-2.75) | 1.19E-02 |  | CA14 | 0.45 (0.23-0.89) | 2.24E-02 |
| OMG | 0.79 (0.65-0.97) | 2.49E-02 |  | ANXA5 | 2.21 (1.19-4.12) | 1.22E-02 |  | DCDC2C | 1.47 (1.05-2.04) | 2.28E-02 |
| C2 | 0.64 (0.43-0.95) | 2.55E-02 |  | INPP5D | 0.57 (0.37-0.89) | 1.24E-02 |  | POMC | 0.63 (0.42-0.94) | 2.28E-02 |
| ASGR2 | 1.85 (1.08-3.18) | 2.55E-02 |  | IKBKG | 0.75 (0.6-0.94) | 1.24E-02 |  | PROS1 | 3.03 (1.17-7.89) | 2.29E-02 |
| LAMA4 | 1.54 (1.05-2.26) | 2.78E-02 |  | ANKRD54 | 0.58 (0.37-0.89) | 1.24E-02 |  | ACTN2 | 0.49 (0.26-0.91) | 2.32E-02 |
| EDIL3 | 1.46 (1.04-2.05) | 2.86E-02 |  | GIT1 | 0.73 (0.57-0.93) | 1.25E-02 |  | LAMTOR5 | 1.76 (1.08-2.87) | 2.42E-02 |
| AGRN | 1.48 (1.04-2.11) | 2.90E-02 |  | DLG4 | 0.63 (0.44-0.91) | 1.25E-02 |  | CD300LG | 0.47 (0.24-0.91) | 2.44E-02 |
| BSND | 0.53 (0.3-0.94) | 2.92E-02 |  | DNAJB6 | 0.69 (0.52-0.92) | 1.25E-02 |  | CALB1 | 0.62 (0.41-0.94) | 2.44E-02 |
| MCAM | 0.63 (0.42-0.96) | 2.95E-02 |  | GHR | 0.37 (0.17-0.81) | 1.26E-02 |  | SLC4A1 | 0.49 (0.26-0.91) | 2.45E-02 |
| FLT3LG | 0.65 (0.44-0.96) | 2.96E-02 |  | EEF1D | 0.71 (0.54-0.93) | 1.27E-02 |  | ROBO2 | 3.05 (1.15-8.09) | 2.46E-02 |
| PTH1R | 1.35 (1.03-1.77) | 2.96E-02 |  | IGFBP2 | 1.52 (1.09-2.12) | 1.29E-02 |  | KLK3 | 0.7 (0.51-0.96) | 2.48E-02 |
| CDC42BPB | 0.84 (0.72-0.98) | 3.00E-02 |  | NECTIN4 | 1.91 (1.15-3.19) | 1.29E-02 |  | KIAA1549 | 1.47 (1.05-2.05) | 2.52E-02 |
| CORO1A | 0.84 (0.71-0.98) | 3.02E-02 |  | IQGAP2 | 0.59 (0.39-0.9) | 1.30E-02 |  | NPR1 | 0.3 (0.1-0.86) | 2.55E-02 |
| LCN2 | 1.41 (1.03-1.93) | 3.04E-02 |  | TPP1 | 1.55 (1.1-2.19) | 1.32E-02 |  | ERBB4 | 2.96 (1.14-7.69) | 2.62E-02 |
| CFB | 1.7 (1.05-2.75) | 3.05E-02 |  | PRND | 1.64 (1.11-2.43) | 1.32E-02 |  | C3 | 1.68 (1.06-2.66) | 2.65E-02 |
| SEPTIN3 | 1.24 (1.02-1.51) | 3.06E-02 |  | CHGB | 1.74 (1.12-2.7) | 1.32E-02 |  | TNFSF10 | 0.37 (0.15-0.89) | 2.65E-02 |
| VCAN | 0.61 (0.39-0.96) | 3.09E-02 |  | PDCD1 | 1.71 (1.12-2.61) | 1.35E-02 |  | ART5 | 0.41 (0.19-0.9) | 2.71E-02 |
| TREH | 0.8 (0.65-0.98) | 3.11E-02 |  | DDHD2 | 0.64 (0.45-0.91) | 1.36E-02 |  | APCS | 2.6 (1.11-6.09) | 2.75E-02 |
| PRSS8 | 1.39 (1.03-1.89) | 3.12E-02 |  | NFE2 | 0.66 (0.47-0.92) | 1.38E-02 |  | BPIFB2 | 1.47 (1.04-2.08) | 2.87E-02 |
| TRIM5 | 0.84 (0.72-0.98) | 3.17E-02 |  | ANKMY2 | 0.68 (0.5-0.93) | 1.42E-02 |  | IL1RN | 1.46 (1.04-2.04) | 2.88E-02 |
| HMOX2 | 0.68 (0.48-0.97) | 3.18E-02 |  | ST13 | 0.59 (0.39-0.9) | 1.44E-02 |  | GKN1 | 2.24 (1.09-4.61) | 2.92E-02 |
| CLC | 1.22 (1.02-1.47) | 3.26E-02 |  | PCDH9 | 0.51 (0.3-0.87) | 1.44E-02 |  | SLK | 0.48 (0.25-0.93) | 2.94E-02 |
| GIGYF2 | 0.79 (0.64-0.98) | 3.28E-02 |  | CTLA4 | 0.37 (0.17-0.82) | 1.44E-02 |  | C1R | 2.58 (1.1-6.04) | 2.95E-02 |
| FOSB | 1.33 (1.02-1.74) | 3.44E-02 |  | IL15 | 2.02 (1.15-3.54) | 1.44E-02 |  | M6PR | 1.76 (1.06-2.93) | 2.97E-02 |
| COL4A1 | 1.43 (1.03-1.98) | 3.45E-02 |  | BPIFB1 | 1.5 (1.08-2.08) | 1.46E-02 |  | OCLN | 1.8 (1.06-3.05) | 2.99E-02 |
| KRT17 | 1.49 (1.03-2.17) | 3.49E-02 |  | GSN | 0.25 (0.08-0.76) | 1.48E-02 |  | CLTA | 1.56 (1.04-2.33) | 2.99E-02 |
| ADGRG2 | 0.59 (0.36-0.96) | 3.49E-02 |  | PPP1CC | 0.69 (0.52-0.93) | 1.48E-02 |  | C8B | 2.87 (1.11-7.44) | 3.00E-02 |
| SPTLC1 | 0.57 (0.33-0.96) | 3.59E-02 |  | IL19 | 1.36 (1.06-1.73) | 1.50E-02 |  | HSD17B3 | 0.45 (0.22-0.93) | 3.06E-02 |
| MICALL2 | 0.75 (0.58-0.98) | 3.61E-02 |  | NFKB1 | 0.66 (0.47-0.92) | 1.51E-02 |  | SNX18 | 0.39 (0.17-0.92) | 3.17E-02 |
| CBS | 0.68 (0.48-0.98) | 3.66E-02 |  | REN | 1.3 (1.05-1.6) | 1.51E-02 |  | IL36G | 1.37 (1.02-1.85) | 3.43E-02 |
| CD5L | 1.41 (1.02-1.96) | 3.68E-02 |  | ATP5PO | 1.65 (1.1-2.48) | 1.54E-02 |  | IL18R1 | 2.11 (1.05-4.24) | 3.59E-02 |
| NDUFB7 | 0.83 (0.69-0.99) | 3.73E-02 |  | CDC42BPB | 0.74 (0.59-0.95) | 1.56E-02 |  | ACRV1 | 0.62 (0.4-0.97) | 3.63E-02 |
| HSBP1 | 1.19 (1.01-1.41) | 3.74E-02 |  | CRH | 0.73 (0.56-0.94) | 1.56E-02 |  | SPESP1 | 0.35 (0.13-0.94) | 3.71E-02 |
| BHLHE40 | 1.23 (1.01-1.49) | 3.75E-02 |  | GTPBP2 | 0.69 (0.51-0.93) | 1.56E-02 |  | FBP1 | 1.37 (1.02-1.85) | 3.73E-02 |
| ESYT2 | 0.81 (0.67-0.99) | 3.83E-02 |  | PPCDC | 0.58 (0.37-0.9) | 1.60E-02 |  | PTH1R | 0.37 (0.15-0.94) | 3.73E-02 |
| CNTN1 | 0.6 (0.37-0.97) | 3.87E-02 |  | IGLC2 | 1.96 (1.13-3.38) | 1.61E-02 |  | MMP9 | 1.53 (1.02-2.28) | 3.83E-02 |
| NAMPT | 0.72 (0.53-0.98) | 3.90E-02 |  | CNDP1 | 0.63 (0.43-0.92) | 1.62E-02 |  | SDK2 | 1.8 (1.03-3.14) | 3.84E-02 |
| SLC13A1 | 1.23 (1.01-1.5) | 3.95E-02 |  | COQ7 | 0.41 (0.2-0.85) | 1.62E-02 |  | SHD | 0.55 (0.31-0.97) | 3.88E-02 |
| CTSZ | 0.64 (0.42-0.98) | 3.95E-02 |  | DPP4 | 0.44 (0.22-0.86) | 1.62E-02 |  | LEPR | 0.42 (0.18-0.96) | 3.89E-02 |
| DHODH | 0.65 (0.43-0.98) | 3.96E-02 |  | LAIR1 | 1.78 (1.11-2.84) | 1.62E-02 |  | MCTS1 | 0.24 (0.06-0.93) | 3.94E-02 |
| DCXR | 0.76 (0.58-0.99) | 4.00E-02 |  | RANBP1 | 0.56 (0.35-0.9) | 1.64E-02 |  | PROK1 | 1.48 (1.02-2.16) | 4.00E-02 |
| SEZ6L | 0.61 (0.38-0.98) | 4.07E-02 |  | MTSS1 | 0.78 (0.63-0.96) | 1.65E-02 |  | CDH2 | 0.49 (0.25-0.97) | 4.03E-02 |
| KLRB1 | 0.7 (0.49-0.98) | 4.08E-02 |  | TSC22D1 | 0.62 (0.42-0.92) | 1.67E-02 |  | MAN1A2 | 3.14 (1.05-9.39) | 4.06E-02 |
| ITGB2 | 0.6 (0.37-0.98) | 4.17E-02 |  | TNFRSF9 | 1.68 (1.1-2.57) | 1.69E-02 |  | KLB | 0.7 (0.5-0.99) | 4.09E-02 |
| IL2RB | 0.64 (0.42-0.98) | 4.19E-02 |  | TNR | 0.56 (0.35-0.9) | 1.70E-02 |  | SIL1 | 1.79 (1.02-3.15) | 4.17E-02 |
| MZB1 | 1.28 (1.01-1.63) | 4.21E-02 |  | ENTPD2 | 0.58 (0.37-0.91) | 1.71E-02 |  | CREBZF | 0.28 (0.08-0.95) | 4.18E-02 |
| ARAF | 0.76 (0.59-0.99) | 4.23E-02 |  | PIK3IP1 | 2.02 (1.13-3.6) | 1.73E-02 |  | COX6B1 | 0.38 (0.15-0.97) | 4.19E-02 |
| GIP | 0.74 (0.55-0.99) | 4.24E-02 |  | WWP2 | 0.62 (0.41-0.92) | 1.76E-02 |  | ACADSB | 0.71 (0.51-0.99) | 4.20E-02 |
| ABO | 1.12 (1-1.25) | 4.29E-02 |  | S100A4 | 0.66 (0.47-0.93) | 1.77E-02 |  | ZNRF4 | 0.55 (0.31-0.98) | 4.28E-02 |
| PRKAR2A | 0.82 (0.68-0.99) | 4.29E-02 |  | PI16 | 0.32 (0.12-0.82) | 1.81E-02 |  | NCR1 | 0.5 (0.26-0.98) | 4.28E-02 |
| FAM3B | 0.68 (0.47-0.99) | 4.30E-02 |  | WASF1 | 0.76 (0.6-0.95) | 1.81E-02 |  | SCGB3A2 | 1.24 (1.01-1.54) | 4.29E-02 |
| GORASP2 | 0.7 (0.49-0.99) | 4.30E-02 |  | ZNF75D | 1.38 (1.06-1.81) | 1.81E-02 |  | RAPGEF2 | 0.53 (0.29-0.98) | 4.30E-02 |
| CA11 | 0.53 (0.29-0.98) | 4.38E-02 |  | THOP1 | 0.52 (0.31-0.9) | 1.82E-02 |  | TNFRSF6B | 1.46 (1.01-2.1) | 4.31E-02 |
| VAMP8 | 0.83 (0.69-0.99) | 4.39E-02 |  | SPINK6 | 1.63 (1.09-2.44) | 1.82E-02 |  | SELE | 1.64 (1.01-2.65) | 4.37E-02 |
| SIRT5 | 1.25 (1.01-1.54) | 4.39E-02 |  | HGF | 1.83 (1.11-3.03) | 1.85E-02 |  | C1GALT1C1 | 0.35 (0.13-0.97) | 4.40E-02 |
| TBC1D5 | 0.83 (0.69-1) | 4.41E-02 |  | RAD23B | 0.56 (0.34-0.91) | 1.87E-02 |  | DCTN2 | 1.29 (1.01-1.65) | 4.42E-02 |
| OMD | 0.74 (0.55-0.99) | 4.42E-02 |  | DPP10 | 1.7 (1.09-2.64) | 1.88E-02 |  | NRP1 | 2.39 (1.02-5.59) | 4.43E-02 |
| NPHS2 | 1.15 (1-1.31) | 4.43E-02 |  | ELOB | 1.72 (1.09-2.69) | 1.88E-02 |  | CEP152 | 1.62 (1.01-2.6) | 4.46E-02 |
| RTN4R | 0.67 (0.45-0.99) | 4.44E-02 |  | BSG | 2.78 (1.18-6.56) | 1.91E-02 |  | LEFTY2 | 0.62 (0.38-0.99) | 4.48E-02 |
| PALM | 0.63 (0.4-0.99) | 4.45E-02 |  | TLR2 | 3.11 (1.2-8.02) | 1.91E-02 |  | APOB | 0.33 (0.11-0.98) | 4.53E-02 |
| ARHGAP25 | 0.77 (0.6-0.99) | 4.45E-02 |  | CLEC6A | 1.64 (1.08-2.49) | 1.93E-02 |  | OTOA | 0.45 (0.2-0.99) | 4.61E-02 |
| USP28 | 1.42 (1.01-2.01) | 4.51E-02 |  | NFKBIE | 0.57 (0.35-0.91) | 1.94E-02 |  | IL6 | 1.26 (1-1.58) | 4.65E-02 |
| CR2 | 0.75 (0.57-0.99) | 4.52E-02 |  | SPTLC1 | 0.37 (0.16-0.85) | 1.95E-02 |  | IFI30 | 1.81 (1.01-3.27) | 4.79E-02 |
| BST2 | 0.74 (0.55-1) | 4.66E-02 |  | ENO3 | 0.58 (0.37-0.92) | 1.98E-02 |  | SIGLEC7 | 2.22 (1.01-4.92) | 4.84E-02 |
| ACTN2 | 0.73 (0.54-1) | 4.66E-02 |  | SPART | 0.68 (0.5-0.94) | 1.99E-02 |  | DSG2 | 0.39 (0.15-1) | 4.90E-02 |
| SMAD3 | 0.73 (0.53-1) | 4.73E-02 |  | TMPRSS15 | 1.3 (1.04-1.62) | 2.01E-02 |  | FMR1 | 1.93 (1-3.72) | 4.93E-02 |
| A1BG | 2.57 (1.01-6.52) | 4.73E-02 |  | CRNN | 0.71 (0.53-0.95) | 2.01E-02 |  | PON3 | 0.52 (0.27-1) | 4.95E-02 |
| PTGES2 | 0.73 (0.54-1) | 4.76E-02 |  | CKB | 1.55 (1.07-2.24) | 2.03E-02 |  | BMP4 | 1.61 (1-2.59) | 4.95E-02 |
| HAVCR1 | 1.22 (1-1.48) | 4.79E-02 |  | GSAP | 0.64 (0.44-0.93) | 2.05E-02 |  | FCRL2 | 1.55 (1-2.4) | 4.95E-02 |
| DBN1 | 0.71 (0.5-1) | 4.80E-02 |  | TGM2 | 0.74 (0.57-0.95) | 2.07E-02 |  | NLGN1 | 1.35 (1-1.82) | 4.99E-02 |
| TFF3 | 1.3 (1-1.69) | 4.82E-02 |  | CD302 | 1.94 (1.11-3.41) | 2.09E-02 |  |  |  |  |
| FRMD4B | 0.78 (0.61-1) | 4.83E-02 |  | EIF4G3 | 0.63 (0.42-0.93) | 2.09E-02 |  |  |  |  |
| GGT1 | 0.75 (0.56-1) | 4.84E-02 |  | CEACAM8 | 1.51 (1.06-2.15) | 2.12E-02 |  |  |  |  |
| STX16 | 0.76 (0.57-1) | 4.84E-02 |  | BIRC2 | 0.67 (0.47-0.94) | 2.14E-02 |  |  |  |  |
| TRIM21 | 0.85 (0.72-1) | 4.85E-02 |  | OGT | 0.53 (0.31-0.91) | 2.14E-02 |  |  |  |  |
| CD300LG | 0.7 (0.49-1) | 4.87E-02 |  | GTF2IRD1 | 1.33 (1.04-1.69) | 2.15E-02 |  |  |  |  |
| IGFBP4 | 1.31 (1-1.71) | 4.91E-02 |  | FMNL1 | 0.61 (0.4-0.93) | 2.17E-02 |  |  |  |  |
| MSLNL | 0.63 (0.39-1) | 4.93E-02 |  | AKT3 | 0.65 (0.44-0.94) | 2.22E-02 |  |  |  |  |
| ANGPTL7 | 0.69 (0.48-1) | 4.93E-02 |  | UNC5D | 0.32 (0.12-0.85) | 2.23E-02 |  |  |  |  |
| CSRP3 | 0.83 (0.69-1) | 4.97E-02 |  | VTI1A | 0.58 (0.36-0.93) | 2.24E-02 |  |  |  |  |
|  |  |  |  | LPP | 0.65 (0.45-0.94) | 2.27E-02 |  |  |  |  |
|  |  |  |  | NPC2 | 1.88 (1.09-3.23) | 2.27E-02 |  |  |  |  |
|  |  |  |  | MYO9B | 0.66 (0.46-0.94) | 2.28E-02 |  |  |  |  |
|  |  |  |  | CLIP2 | 0.82 (0.69-0.97) | 2.28E-02 |  |  |  |  |
|  |  |  |  | KAZN | 0.78 (0.63-0.97) | 2.29E-02 |  |  |  |  |
|  |  |  |  | CCL28 | 1.42 (1.05-1.92) | 2.30E-02 |  |  |  |  |
|  |  |  |  | CHCHD10 | 1.7 (1.08-2.69) | 2.31E-02 |  |  |  |  |
|  |  |  |  | ADM | 2.34 (1.12-4.87) | 2.32E-02 |  |  |  |  |
|  |  |  |  | PDLIM5 | 0.81 (0.67-0.97) | 2.36E-02 |  |  |  |  |
|  |  |  |  | PDZD2 | 0.56 (0.34-0.93) | 2.37E-02 |  |  |  |  |
|  |  |  |  | PFKFB2 | 0.74 (0.57-0.96) | 2.41E-02 |  |  |  |  |
|  |  |  |  | UGDH | 0.61 (0.4-0.94) | 2.46E-02 |  |  |  |  |
|  |  |  |  | BLMH | 0.56 (0.34-0.93) | 2.48E-02 |  |  |  |  |
|  |  |  |  | FOLR1 | 2.04 (1.09-3.8) | 2.49E-02 |  |  |  |  |
|  |  |  |  | RSPO3 | 2.04 (1.09-3.81) | 2.51E-02 |  |  |  |  |
|  |  |  |  | C1RL | 4.13 (1.19-14.32) | 2.53E-02 |  |  |  |  |
|  |  |  |  | ARHGAP45 | 0.72 (0.54-0.96) | 2.53E-02 |  |  |  |  |
|  |  |  |  | NFATC1 | 0.79 (0.64-0.97) | 2.54E-02 |  |  |  |  |
|  |  |  |  | ZBTB16 | 0.7 (0.51-0.96) | 2.55E-02 |  |  |  |  |
|  |  |  |  | PTPRN2 | 1.86 (1.08-3.19) | 2.55E-02 |  |  |  |  |
|  |  |  |  | ISLR2 | 1.77 (1.07-2.92) | 2.56E-02 |  |  |  |  |
|  |  |  |  | RELT | 1.96 (1.08-3.54) | 2.57E-02 |  |  |  |  |
|  |  |  |  | CALCA | 1.34 (1.04-1.73) | 2.61E-02 |  |  |  |  |
|  |  |  |  | DNAJC6 | 0.72 (0.54-0.96) | 2.67E-02 |  |  |  |  |
|  |  |  |  | RPS10 | 0.43 (0.2-0.91) | 2.69E-02 |  |  |  |  |
|  |  |  |  | LAYN | 1.73 (1.06-2.8) | 2.69E-02 |  |  |  |  |
|  |  |  |  | RNF168 | 0.42 (0.19-0.91) | 2.71E-02 |  |  |  |  |
|  |  |  |  | DNAJB2 | 0.59 (0.37-0.94) | 2.71E-02 |  |  |  |  |
|  |  |  |  | WAS | 0.68 (0.48-0.96) | 2.71E-02 |  |  |  |  |
|  |  |  |  | REPS1 | 0.44 (0.21-0.91) | 2.71E-02 |  |  |  |  |
|  |  |  |  | RBFOX3 | 1.67 (1.06-2.63) | 2.73E-02 |  |  |  |  |
|  |  |  |  | SPINK2 | 1.89 (1.07-3.33) | 2.73E-02 |  |  |  |  |
|  |  |  |  | PRKG1 | 0.81 (0.68-0.98) | 2.79E-02 |  |  |  |  |
|  |  |  |  | DSC2 | 1.76 (1.06-2.92) | 2.80E-02 |  |  |  |  |
|  |  |  |  | MMP7 | 1.61 (1.05-2.46) | 2.80E-02 |  |  |  |  |
|  |  |  |  | MCAM | 0.5 (0.27-0.93) | 2.81E-02 |  |  |  |  |
|  |  |  |  | GP1BA | 0.5 (0.27-0.93) | 2.81E-02 |  |  |  |  |
|  |  |  |  | OGA | 0.69 (0.49-0.96) | 2.86E-02 |  |  |  |  |
|  |  |  |  | AMOT | 0.51 (0.28-0.93) | 2.86E-02 |  |  |  |  |
|  |  |  |  | VPS37A | 0.75 (0.58-0.97) | 2.86E-02 |  |  |  |  |
|  |  |  |  | SV2A | 0.61 (0.39-0.95) | 2.88E-02 |  |  |  |  |
|  |  |  |  | EIF2AK2 | 0.78 (0.62-0.97) | 2.90E-02 |  |  |  |  |
|  |  |  |  | CHMP6 | 0.63 (0.42-0.95) | 2.90E-02 |  |  |  |  |
|  |  |  |  | CCT5 | 0.56 (0.33-0.94) | 2.92E-02 |  |  |  |  |
|  |  |  |  | ACVRL1 | 2.07 (1.08-3.96) | 2.92E-02 |  |  |  |  |
|  |  |  |  | GYS1 | 0.78 (0.62-0.97) | 2.95E-02 |  |  |  |  |
|  |  |  |  | PIBF1 | 0.71 (0.52-0.97) | 2.95E-02 |  |  |  |  |
|  |  |  |  | PIKFYVE | 0.55 (0.32-0.94) | 2.99E-02 |  |  |  |  |
|  |  |  |  | BRAP | 0.8 (0.65-0.98) | 3.05E-02 |  |  |  |  |
|  |  |  |  | DHODH | 0.49 (0.25-0.94) | 3.08E-02 |  |  |  |  |
|  |  |  |  | SIGLEC5 | 1.29 (1.02-1.62) | 3.08E-02 |  |  |  |  |
|  |  |  |  | PTGR1 | 1.33 (1.03-1.72) | 3.10E-02 |  |  |  |  |
|  |  |  |  | CRTAP | 0.45 (0.22-0.93) | 3.10E-02 |  |  |  |  |
|  |  |  |  | YJU2 | 0.42 (0.19-0.92) | 3.13E-02 |  |  |  |  |
|  |  |  |  | CC2D1A | 0.72 (0.54-0.97) | 3.14E-02 |  |  |  |  |
|  |  |  |  | VAT1 | 0.31 (0.11-0.9) | 3.20E-02 |  |  |  |  |
|  |  |  |  | CFHR5 | 1.87 (1.06-3.3) | 3.20E-02 |  |  |  |  |
|  |  |  |  | SEZ6L2 | 2.23 (1.07-4.65) | 3.21E-02 |  |  |  |  |
|  |  |  |  | CSDE1 | 0.74 (0.56-0.97) | 3.21E-02 |  |  |  |  |
|  |  |  |  | NAGK | 0.62 (0.4-0.96) | 3.21E-02 |  |  |  |  |
|  |  |  |  | RRM2B | 0.71 (0.52-0.97) | 3.22E-02 |  |  |  |  |
|  |  |  |  | SAMD9L | 0.72 (0.54-0.97) | 3.24E-02 |  |  |  |  |
|  |  |  |  | ATP6V1G1 | 0.69 (0.49-0.97) | 3.26E-02 |  |  |  |  |
|  |  |  |  | HPGDS | 0.55 (0.32-0.95) | 3.32E-02 |  |  |  |  |
|  |  |  |  | RAPGEF2 | 0.58 (0.35-0.96) | 3.34E-02 |  |  |  |  |
|  |  |  |  | GFRA2 | 0.37 (0.15-0.93) | 3.38E-02 |  |  |  |  |
|  |  |  |  | YWHAQ | 0.7 (0.5-0.97) | 3.39E-02 |  |  |  |  |
|  |  |  |  | IL18R1 | 1.89 (1.05-3.4) | 3.43E-02 |  |  |  |  |
|  |  |  |  | IFNAR1 | 2.15 (1.06-4.36) | 3.43E-02 |  |  |  |  |
|  |  |  |  | ADAM22 | 0.55 (0.32-0.96) | 3.44E-02 |  |  |  |  |
|  |  |  |  | PTRHD1 | 0.65 (0.44-0.97) | 3.47E-02 |  |  |  |  |
|  |  |  |  | C7 | 2.02 (1.05-3.9) | 3.52E-02 |  |  |  |  |
|  |  |  |  | LARP1 | 0.62 (0.4-0.97) | 3.52E-02 |  |  |  |  |
|  |  |  |  | AGER | 0.6 (0.37-0.97) | 3.53E-02 |  |  |  |  |
|  |  |  |  | LZTFL1 | 0.69 (0.49-0.97) | 3.53E-02 |  |  |  |  |
|  |  |  |  | SNU13 | 0.53 (0.29-0.96) | 3.55E-02 |  |  |  |  |
|  |  |  |  | AP2B1 | 0.54 (0.3-0.96) | 3.55E-02 |  |  |  |  |
|  |  |  |  | PTK7 | 1.72 (1.04-2.86) | 3.56E-02 |  |  |  |  |
|  |  |  |  | DDX53 | 1.79 (1.04-3.09) | 3.56E-02 |  |  |  |  |
|  |  |  |  | PILRA | 1.66 (1.03-2.66) | 3.57E-02 |  |  |  |  |
|  |  |  |  | TPPP3 | 0.49 (0.25-0.95) | 3.57E-02 |  |  |  |  |
|  |  |  |  | DCTN1 | 0.71 (0.52-0.98) | 3.58E-02 |  |  |  |  |
|  |  |  |  | TREM2 | 1.45 (1.02-2.06) | 3.61E-02 |  |  |  |  |
|  |  |  |  | APOE | 0.6 (0.37-0.97) | 3.64E-02 |  |  |  |  |
|  |  |  |  | MPO | 1.48 (1.03-2.14) | 3.64E-02 |  |  |  |  |
|  |  |  |  | RNASEH2A | 0.5 (0.26-0.96) | 3.65E-02 |  |  |  |  |
|  |  |  |  | SERPINA12 | 1.19 (1.01-1.41) | 3.65E-02 |  |  |  |  |
|  |  |  |  | HAVCR2 | 1.74 (1.03-2.94) | 3.69E-02 |  |  |  |  |
|  |  |  |  | ULBP2 | 1.67 (1.03-2.72) | 3.71E-02 |  |  |  |  |
|  |  |  |  | PRKAR2A | 0.73 (0.55-0.98) | 3.73E-02 |  |  |  |  |
|  |  |  |  | IRAG2 | 0.75 (0.58-0.98) | 3.76E-02 |  |  |  |  |
|  |  |  |  | PPP1R9B | 0.8 (0.64-0.99) | 3.76E-02 |  |  |  |  |
|  |  |  |  | NTF4 | 1.47 (1.02-2.12) | 3.77E-02 |  |  |  |  |
|  |  |  |  | NAA10 | 0.61 (0.39-0.97) | 3.78E-02 |  |  |  |  |
|  |  |  |  | PARD3 | 0.67 (0.46-0.98) | 3.79E-02 |  |  |  |  |
|  |  |  |  | MNAT1 | 0.49 (0.25-0.96) | 3.80E-02 |  |  |  |  |
|  |  |  |  | USP25 | 0.7 (0.5-0.98) | 3.83E-02 |  |  |  |  |
|  |  |  |  | HEXIM1 | 0.76 (0.59-0.99) | 3.84E-02 |  |  |  |  |
|  |  |  |  | WNT9A | 1.85 (1.03-3.32) | 3.93E-02 |  |  |  |  |
|  |  |  |  | EGFL7 | 1.63 (1.02-2.6) | 3.97E-02 |  |  |  |  |
|  |  |  |  | TNFRSF1A | 1.78 (1.03-3.09) | 3.97E-02 |  |  |  |  |
|  |  |  |  | APCS | 2.12 (1.03-4.36) | 4.00E-02 |  |  |  |  |
|  |  |  |  | RBPMS2 | 0.78 (0.62-0.99) | 4.03E-02 |  |  |  |  |
|  |  |  |  | RSPO1 | 1.8 (1.03-3.16) | 4.06E-02 |  |  |  |  |
|  |  |  |  | BMP10 | 0.46 (0.22-0.97) | 4.08E-02 |  |  |  |  |
|  |  |  |  | EIF2AK3 | 0.64 (0.42-0.98) | 4.10E-02 |  |  |  |  |
|  |  |  |  | GASK1A | 0.56 (0.32-0.98) | 4.11E-02 |  |  |  |  |
|  |  |  |  | FARSA | 0.65 (0.42-0.98) | 4.12E-02 |  |  |  |  |
|  |  |  |  | KLK13 | 1.5 (1.02-2.2) | 4.14E-02 |  |  |  |  |
|  |  |  |  | ICOSLG | 0.33 (0.11-0.96) | 4.18E-02 |  |  |  |  |
|  |  |  |  | MSLNL | 0.47 (0.23-0.97) | 4.18E-02 |  |  |  |  |
|  |  |  |  | ARFIP1 | 0.7 (0.49-0.99) | 4.19E-02 |  |  |  |  |
|  |  |  |  | RAB27B | 0.76 (0.59-0.99) | 4.20E-02 |  |  |  |  |
|  |  |  |  | TSC1 | 0.57 (0.33-0.98) | 4.23E-02 |  |  |  |  |
|  |  |  |  | SCP2 | 0.67 (0.46-0.99) | 4.25E-02 |  |  |  |  |
|  |  |  |  | ANXA11 | 0.75 (0.57-0.99) | 4.27E-02 |  |  |  |  |
|  |  |  |  | CNTN3 | 0.52 (0.28-0.98) | 4.28E-02 |  |  |  |  |
|  |  |  |  | BACH1 | 0.74 (0.56-0.99) | 4.29E-02 |  |  |  |  |
|  |  |  |  | FGF5 | 1.85 (1.02-3.34) | 4.31E-02 |  |  |  |  |
|  |  |  |  | BLOC1S3 | 0.51 (0.27-0.98) | 4.31E-02 |  |  |  |  |
|  |  |  |  | ATXN2L | 0.54 (0.3-0.98) | 4.32E-02 |  |  |  |  |
|  |  |  |  | BPIFB2 | 0.67 (0.45-0.99) | 4.33E-02 |  |  |  |  |
|  |  |  |  | FGA | 2.08 (1.02-4.21) | 4.33E-02 |  |  |  |  |
|  |  |  |  | TMPRSS11B | 0.65 (0.42-0.99) | 4.36E-02 |  |  |  |  |
|  |  |  |  | CD2AP | 0.72 (0.53-0.99) | 4.37E-02 |  |  |  |  |
|  |  |  |  | ARHGAP25 | 0.67 (0.46-0.99) | 4.38E-02 |  |  |  |  |
|  |  |  |  | FOXJ3 | 0.51 (0.27-0.98) | 4.38E-02 |  |  |  |  |
|  |  |  |  | ZP3 | 0.92 (0.84-1) | 4.41E-02 |  |  |  |  |
|  |  |  |  | ACP6 | 1.42 (1.01-1.99) | 4.44E-02 |  |  |  |  |
|  |  |  |  | BRSK2 | 0.46 (0.22-0.98) | 4.45E-02 |  |  |  |  |
|  |  |  |  | EHBP1 | 0.72 (0.52-0.99) | 4.46E-02 |  |  |  |  |
|  |  |  |  | CREB3 | 1.38 (1.01-1.88) | 4.48E-02 |  |  |  |  |
|  |  |  |  | VSIG10L | 0.44 (0.19-0.98) | 4.49E-02 |  |  |  |  |
|  |  |  |  | BIN2 | 0.79 (0.63-0.99) | 4.51E-02 |  |  |  |  |
|  |  |  |  | SOD3 | 0.48 (0.23-0.98) | 4.51E-02 |  |  |  |  |
|  |  |  |  | TMEM132A | 0.67 (0.45-0.99) | 4.52E-02 |  |  |  |  |
|  |  |  |  | HEG1 | 2.23 (1.02-4.88) | 4.55E-02 |  |  |  |  |
|  |  |  |  | CALCOCO2 | 0.72 (0.52-0.99) | 4.56E-02 |  |  |  |  |
|  |  |  |  | SCGB1A1 | 0.74 (0.55-0.99) | 4.61E-02 |  |  |  |  |
|  |  |  |  | APRT | 0.66 (0.44-0.99) | 4.62E-02 |  |  |  |  |
|  |  |  |  | VEGFA | 1.39 (1.01-1.92) | 4.63E-02 |  |  |  |  |
|  |  |  |  | RYR1 | 0.4 (0.16-0.99) | 4.70E-02 |  |  |  |  |
|  |  |  |  | EPHA1 | 0.55 (0.31-0.99) | 4.70E-02 |  |  |  |  |
|  |  |  |  | CLEC3B | 0.33 (0.11-0.99) | 4.71E-02 |  |  |  |  |
|  |  |  |  | VCPKMT | 0.67 (0.45-1) | 4.74E-02 |  |  |  |  |
|  |  |  |  | GZMH | 0.74 (0.55-1) | 4.74E-02 |  |  |  |  |
|  |  |  |  | FDX1 | 1.37 (1-1.86) | 4.75E-02 |  |  |  |  |
|  |  |  |  | CORO1A | 0.78 (0.6-1) | 4.77E-02 |  |  |  |  |
|  |  |  |  | TSPAN7 | 0.67 (0.45-1) | 4.78E-02 |  |  |  |  |
|  |  |  |  | NUMB | 0.75 (0.57-1) | 4.81E-02 |  |  |  |  |
|  |  |  |  | ATG16L1 | 0.76 (0.58-1) | 4.85E-02 |  |  |  |  |
|  |  |  |  | MAP2K6 | 0.82 (0.67-1) | 4.86E-02 |  |  |  |  |
|  |  |  |  | COL18A1 | 2.23 (1-4.95) | 4.89E-02 |  |  |  |  |
|  |  |  |  | ANPEP | 0.47 (0.22-1) | 4.90E-02 |  |  |  |  |
|  |  |  |  | GALNT2 | 0.47 (0.22-1) | 4.93E-02 |  |  |  |  |
|  |  |  |  | NFASC | 1.87 (1-3.51) | 4.98E-02 |  |  |  |  |

| **Supplementary table 6. Two-sample Mendelian randomization results for lung adenocarcinoma.** | | | | | | |
| --- | --- | --- | --- | --- | --- | --- |
| **Protein** | **Method** | **Nsnp** | **Beta** | **SE** | ***P*** | **OR (95%CI)** |
| TMCO5A | Wald ratio | 1 | 1.31 | 0.29 | 6.54E-06 | 3.72 (2.1-6.59) |
| RNASET2 | Inverse variance weighted | 17 | 0.18 | 0.04 | 4.14E-05 | 1.2 (1.1-1.3) |
| EPHA4 | Inverse variance weighted | 11 | 0.15 | 0.04 | 9.05E-05 | 1.16 (1.08-1.25) |
| PLXDC2 | Inverse variance weighted | 14 | 0.20 | 0.05 | 3.65E-04 | 1.22 (1.09-1.35) |
| CXCL10 | Inverse variance weighted | 11 | -0.37 | 0.10 | 4.20E-04 | 0.69 (0.56-0.85) |
| INPP5J | Inverse variance weighted | 3 | -0.78 | 0.22 | 4.86E-04 | 0.46 (0.3-0.71) |
| SCT | Inverse variance weighted | 5 | 0.52 | 0.15 | 6.10E-04 | 1.69 (1.25-2.28) |
| JCHAIN | Inverse variance weighted | 18 | 0.26 | 0.08 | 6.95E-04 | 1.3 (1.12-1.52) |
| SEMA4C | Inverse variance weighted | 7 | 0.22 | 0.07 | 8.08E-04 | 1.25 (1.1-1.42) |
| IL18 | Inverse variance weighted | 17 | -0.17 | 0.05 | 9.26E-04 | 0.84 (0.76-0.93) |
| DDR1 | Inverse variance weighted | 16 | 0.24 | 0.08 | 1.71E-03 | 1.28 (1.1-1.49) |
| NECTIN1 | Inverse variance weighted | 2 | 0.24 | 0.08 | 1.73E-03 | 1.27 (1.09-1.48) |
| SCG2 | Inverse variance weighted | 8 | 0.49 | 0.16 | 1.80E-03 | 1.63 (1.2-2.21) |
| ROBO4 | Inverse variance weighted | 5 | 0.11 | 0.03 | 1.83E-03 | 1.11 (1.04-1.19) |
| GOLM2 | Inverse variance weighted | 14 | -0.15 | 0.05 | 2.01E-03 | 0.86 (0.78-0.95) |
| CD84 | Inverse variance weighted | 19 | -0.15 | 0.05 | 2.10E-03 | 0.86 (0.78-0.95) |
| CD5 | Inverse variance weighted | 13 | -0.19 | 0.06 | 2.35E-03 | 0.83 (0.73-0.93) |
| IL19 | Inverse variance weighted | 9 | 0.13 | 0.04 | 2.44E-03 | 1.14 (1.05-1.25) |
| TYMP | Inverse variance weighted | 5 | -0.27 | 0.09 | 2.53E-03 | 0.77 (0.64-0.91) |
| IFNGR1 | Inverse variance weighted | 18 | 0.16 | 0.05 | 2.75E-03 | 1.17 (1.06-1.31) |
| TINAGL1 | Inverse variance weighted | 13 | -0.26 | 0.09 | 3.14E-03 | 0.77 (0.65-0.92) |
| ISLR2 | Inverse variance weighted | 13 | 0.12 | 0.04 | 3.95E-03 | 1.12 (1.04-1.21) |
| NPTX1 | Inverse variance weighted | 22 | 0.16 | 0.06 | 4.04E-03 | 1.18 (1.05-1.32) |
| CD5L | Inverse variance weighted | 42 | 0.14 | 0.05 | 4.05E-03 | 1.15 (1.04-1.26) |
| GYS1 | Inverse variance weighted | 9 | -0.17 | 0.06 | 4.15E-03 | 0.84 (0.75-0.95) |
| JAM2 | Inverse variance weighted | 12 | 0.19 | 0.07 | 4.21E-03 | 1.21 (1.06-1.37) |
| TCN1 | Inverse variance weighted | 32 | 0.08 | 0.03 | 4.29E-03 | 1.09 (1.03-1.15) |
| FCAMR | Inverse variance weighted | 21 | -0.15 | 0.05 | 4.43E-03 | 0.86 (0.78-0.96) |
| LMNB2 | Inverse variance weighted | 9 | -0.40 | 0.14 | 4.54E-03 | 0.67 (0.51-0.88) |
| EPHB4 | Inverse variance weighted | 17 | 0.13 | 0.04 | 4.74E-03 | 1.13 (1.04-1.24) |
| ITGAL | Inverse variance weighted | 5 | -0.33 | 0.12 | 4.86E-03 | 0.72 (0.57-0.9) |
| ARG2 | Inverse variance weighted | 4 | 0.45 | 0.16 | 4.91E-03 | 1.56 (1.14-2.13) |
| CRX | Wald ratio | 1 | 0.98 | 0.35 | 4.99E-03 | 2.68 (1.35-5.32) |
| SCAMP3 | Inverse variance weighted | 8 | -0.29 | 0.10 | 5.14E-03 | 0.75 (0.61-0.92) |
| GCHFR | Inverse variance weighted | 4 | -0.38 | 0.14 | 5.62E-03 | 0.69 (0.53-0.9) |
| ANXA10 | Inverse variance weighted | 2 | -0.66 | 0.24 | 5.62E-03 | 0.52 (0.33-0.83) |
| WNT9A | Inverse variance weighted | 3 | 0.34 | 0.12 | 5.63E-03 | 1.4 (1.1-1.77) |
| PCSK7 | Wald ratio | 1 | 0.56 | 0.20 | 5.83E-03 | 1.75 (1.18-2.61) |
| CEP152 | Wald ratio | 1 | -1.01 | 0.37 | 5.97E-03 | 0.37 (0.18-0.75) |
| ROR1 | Inverse variance weighted | 21 | 0.12 | 0.04 | 6.18E-03 | 1.13 (1.04-1.23) |
| CX3CL1 | Inverse variance weighted | 22 | 0.27 | 0.10 | 6.71E-03 | 1.31 (1.08-1.58) |
| PROK1 | Inverse variance weighted | 7 | -0.21 | 0.08 | 6.94E-03 | 0.81 (0.7-0.94) |
| TGFBR2 | Inverse variance weighted | 21 | 0.18 | 0.07 | 7.02E-03 | 1.2 (1.05-1.37) |
| CD164 | Inverse variance weighted | 12 | -0.20 | 0.07 | 7.10E-03 | 0.82 (0.71-0.95) |
| IL7R | Inverse variance weighted | 32 | 0.09 | 0.03 | 7.47E-03 | 1.09 (1.02-1.16) |
| GP5 | Inverse variance weighted | 10 | -0.23 | 0.09 | 7.73E-03 | 0.79 (0.67-0.94) |
| PRSS8 | Inverse variance weighted | 12 | -0.24 | 0.09 | 7.98E-03 | 0.79 (0.66-0.94) |
| TIMP3 | Inverse variance weighted | 33 | -0.10 | 0.04 | 8.07E-03 | 0.91 (0.85-0.98) |
| NPDC1 | Inverse variance weighted | 10 | 0.31 | 0.12 | 8.95E-03 | 1.36 (1.08-1.72) |
| ECI2 | Inverse variance weighted | 3 | -0.16 | 0.06 | 9.18E-03 | 0.85 (0.75-0.96) |
| ITGBL1 | Inverse variance weighted | 9 | 0.12 | 0.05 | 9.77E-03 | 1.13 (1.03-1.23) |
| SFRP1 | Inverse variance weighted | 11 | 0.15 | 0.06 | 1.02E-02 | 1.17 (1.04-1.31) |
| MGMT | Inverse variance weighted | 6 | 0.15 | 0.06 | 1.03E-02 | 1.16 (1.04-1.31) |
| ALCAM | Inverse variance weighted | 18 | 0.20 | 0.08 | 1.07E-02 | 1.22 (1.05-1.41) |
| CD40 | Inverse variance weighted | 13 | -0.13 | 0.05 | 1.07E-02 | 0.87 (0.79-0.97) |
| MMP1 | Inverse variance weighted | 28 | -0.09 | 0.04 | 1.08E-02 | 0.91 (0.85-0.98) |
| CLEC14A | Inverse variance weighted | 12 | 0.12 | 0.05 | 1.11E-02 | 1.12 (1.03-1.23) |
| CENPF | Wald ratio | 1 | -0.74 | 0.29 | 1.12E-02 | 0.48 (0.27-0.85) |
| LIFR | Inverse variance weighted | 21 | 0.09 | 0.04 | 1.14E-02 | 1.1 (1.02-1.18) |
| PRTN3 | Inverse variance weighted | 26 | 0.10 | 0.04 | 1.16E-02 | 1.11 (1.02-1.2) |
| KIRREL2 | Inverse variance weighted | 20 | 0.13 | 0.05 | 1.18E-02 | 1.14 (1.03-1.25) |
| THSD1 | Inverse variance weighted | 6 | 0.20 | 0.08 | 1.22E-02 | 1.22 (1.04-1.43) |
| DAG1 | Inverse variance weighted | 10 | -0.27 | 0.11 | 1.22E-02 | 0.76 (0.62-0.94) |
| NCR3LG1 | Inverse variance weighted | 26 | 0.10 | 0.04 | 1.26E-02 | 1.1 (1.02-1.19) |
| FOLR1 | Inverse variance weighted | 11 | 0.27 | 0.11 | 1.28E-02 | 1.31 (1.06-1.63) |
| GCNT1 | Inverse variance weighted | 14 | -0.15 | 0.06 | 1.29E-02 | 0.86 (0.76-0.97) |
| DCC | Inverse variance weighted | 3 | 0.57 | 0.23 | 1.35E-02 | 1.76 (1.12-2.76) |
| ITGB7 | Inverse variance weighted | 31 | -0.09 | 0.04 | 1.40E-02 | 0.91 (0.85-0.98) |
| BTN1A1 | Inverse variance weighted | 2 | -0.22 | 0.09 | 1.42E-02 | 0.8 (0.67-0.96) |
| EVI5 | Inverse variance weighted | 6 | -0.25 | 0.10 | 1.44E-02 | 0.78 (0.64-0.95) |
| IFNL1 | Inverse variance weighted | 10 | -0.33 | 0.14 | 1.64E-02 | 0.72 (0.54-0.94) |
| TNFRSF21 | Inverse variance weighted | 20 | 0.16 | 0.07 | 1.69E-02 | 1.17 (1.03-1.33) |
| APOC1 | Inverse variance weighted | 13 | 0.12 | 0.05 | 1.71E-02 | 1.13 (1.02-1.25) |
| SDC1 | Inverse variance weighted | 18 | 0.16 | 0.07 | 1.71E-02 | 1.18 (1.03-1.34) |
| CFP | Inverse variance weighted | 10 | -0.18 | 0.08 | 1.77E-02 | 0.84 (0.72-0.97) |
| INSL3 | Inverse variance weighted | 2 | -0.66 | 0.28 | 1.82E-02 | 0.52 (0.3-0.89) |
| WFIKKN1 | Inverse variance weighted | 27 | 0.11 | 0.05 | 1.82E-02 | 1.12 (1.02-1.23) |
| ENTPD2 | Inverse variance weighted | 8 | 0.16 | 0.07 | 1.83E-02 | 1.17 (1.03-1.33) |
| ADAMTS8 | Inverse variance weighted | 14 | -0.10 | 0.04 | 1.91E-02 | 0.9 (0.83-0.98) |
| APOE | Inverse variance weighted | 13 | 0.10 | 0.04 | 1.97E-02 | 1.1 (1.02-1.2) |
| CRYM | Inverse variance weighted | 5 | -0.20 | 0.09 | 2.01E-02 | 0.82 (0.69-0.97) |
| NRCAM | Inverse variance weighted | 15 | 0.11 | 0.05 | 2.02E-02 | 1.12 (1.02-1.23) |
| GGA1 | Wald ratio | 1 | -0.95 | 0.41 | 2.04E-02 | 0.39 (0.17-0.86) |
| BST2 | Inverse variance weighted | 27 | -0.17 | 0.07 | 2.09E-02 | 0.85 (0.73-0.98) |
| GNPDA1 | Inverse variance weighted | 3 | 0.30 | 0.13 | 2.11E-02 | 1.35 (1.05-1.75) |
| PKD1 | Inverse variance weighted | 17 | 0.13 | 0.06 | 2.12E-02 | 1.14 (1.02-1.27) |
| TXNDC15 | Inverse variance weighted | 19 | -0.09 | 0.04 | 2.13E-02 | 0.91 (0.84-0.99) |
| CTHRC1 | Inverse variance weighted | 9 | 0.34 | 0.15 | 2.14E-02 | 1.4 (1.05-1.87) |
| SLC9A3R2 | Inverse variance weighted | 6 | -0.21 | 0.09 | 2.19E-02 | 0.81 (0.67-0.97) |
| KLK13 | Inverse variance weighted | 17 | -0.11 | 0.05 | 2.22E-02 | 0.9 (0.82-0.98) |
| CD1C | Inverse variance weighted | 31 | -0.10 | 0.05 | 2.23E-02 | 0.9 (0.82-0.99) |
| GABARAP | Inverse variance weighted | 3 | 0.43 | 0.19 | 2.24E-02 | 1.54 (1.06-2.24) |
| LAMP1 | Inverse variance weighted | 8 | 0.32 | 0.14 | 2.26E-02 | 1.38 (1.05-1.83) |
| ANP32C | Wald ratio | 1 | -0.94 | 0.41 | 2.29E-02 | 0.39 (0.17-0.88) |
| FLT3LG | Inverse variance weighted | 41 | -0.20 | 0.09 | 2.30E-02 | 0.82 (0.7-0.97) |
| TSPAN15 | Wald ratio | 1 | -0.66 | 0.29 | 2.31E-02 | 0.52 (0.29-0.91) |
| BTN2A1 | Inverse variance weighted | 18 | 0.14 | 0.06 | 2.31E-02 | 1.15 (1.02-1.29) |
| TPMT | Inverse variance weighted | 5 | 0.16 | 0.07 | 2.33E-02 | 1.18 (1.02-1.36) |
| SERPINH1 | Inverse variance weighted | 8 | 0.23 | 0.10 | 2.42E-02 | 1.26 (1.03-1.53) |
| SLC51B | Inverse variance weighted | 17 | -0.13 | 0.06 | 2.45E-02 | 0.88 (0.79-0.98) |
| CD93 | Inverse variance weighted | 12 | 0.20 | 0.09 | 2.48E-02 | 1.22 (1.03-1.46) |
| HGFAC | Inverse variance weighted | 28 | -0.07 | 0.03 | 2.61E-02 | 0.93 (0.87-0.99) |
| NPTXR | Inverse variance weighted | 11 | 0.27 | 0.12 | 2.62E-02 | 1.31 (1.03-1.67) |
| LY9 | Inverse variance weighted | 23 | -0.08 | 0.04 | 2.64E-02 | 0.92 (0.85-0.99) |
| WFIKKN2 | Inverse variance weighted | 12 | -0.08 | 0.03 | 2.64E-02 | 0.93 (0.87-0.99) |
| HNMT | Inverse variance weighted | 9 | -0.11 | 0.05 | 2.77E-02 | 0.9 (0.82-0.99) |
| TFPI | Inverse variance weighted | 10 | 0.10 | 0.05 | 2.82E-02 | 1.11 (1.01-1.21) |
| MCAM | Inverse variance weighted | 18 | 0.15 | 0.07 | 2.83E-02 | 1.16 (1.02-1.33) |
| ADAMTS13 | Inverse variance weighted | 26 | 0.09 | 0.04 | 2.85E-02 | 1.09 (1.01-1.18) |
| ITPRIP | Wald ratio | 1 | 0.88 | 0.40 | 2.86E-02 | 2.4 (1.1-5.26) |
| GFRA2 | Inverse variance weighted | 10 | -0.14 | 0.06 | 2.86E-02 | 0.87 (0.77-0.99) |
| ITGB2 | Inverse variance weighted | 22 | -0.15 | 0.07 | 2.87E-02 | 0.86 (0.75-0.98) |
| ANK2 | Inverse variance weighted | 2 | 0.47 | 0.22 | 2.89E-02 | 1.61 (1.05-2.45) |
| BCHE | Inverse variance weighted | 21 | -0.07 | 0.03 | 2.92E-02 | 0.93 (0.87-0.99) |
| SPINK5 | Inverse variance weighted | 15 | 0.13 | 0.06 | 2.94E-02 | 1.14 (1.01-1.28) |
| COL2A1 | Inverse variance weighted | 36 | -0.07 | 0.03 | 2.96E-02 | 0.93 (0.87-0.99) |
| C5 | Inverse variance weighted | 2 | -0.23 | 0.10 | 2.98E-02 | 0.8 (0.65-0.98) |
| CRH | Inverse variance weighted | 46 | -0.08 | 0.04 | 3.02E-02 | 0.92 (0.85-0.99) |
| SERPING1 | Inverse variance weighted | 15 | -0.13 | 0.06 | 3.06E-02 | 0.88 (0.79-0.99) |
| ERBB4 | Inverse variance weighted | 27 | 0.11 | 0.05 | 3.07E-02 | 1.11 (1.01-1.23) |
| SEPTIN3 | Inverse variance weighted | 16 | 0.11 | 0.05 | 3.09E-02 | 1.12 (1.01-1.24) |
| SCARF2 | Inverse variance weighted | 19 | 0.12 | 0.06 | 3.33E-02 | 1.13 (1.01-1.26) |
| AGRN | Inverse variance weighted | 13 | 0.12 | 0.06 | 3.39E-02 | 1.12 (1.01-1.25) |
| IFIT3 | Inverse variance weighted | 5 | -0.15 | 0.07 | 3.42E-02 | 0.86 (0.75-0.99) |
| ALPI | Inverse variance weighted | 9 | 0.12 | 0.06 | 3.50E-02 | 1.12 (1.01-1.25) |
| FGF2 | Inverse variance weighted | 14 | -0.08 | 0.04 | 3.59E-02 | 0.93 (0.86-0.99) |
| TYRO3 | Inverse variance weighted | 26 | 0.09 | 0.04 | 3.64E-02 | 1.09 (1.01-1.18) |
| NECTIN4 | Inverse variance weighted | 12 | 0.13 | 0.06 | 3.67E-02 | 1.13 (1.01-1.28) |
| THY1 | Inverse variance weighted | 9 | -0.11 | 0.05 | 3.70E-02 | 0.9 (0.81-0.99) |
| GALNT3 | Inverse variance weighted | 17 | -0.12 | 0.06 | 3.74E-02 | 0.89 (0.8-0.99) |
| THBD | Inverse variance weighted | 23 | 0.12 | 0.06 | 3.75E-02 | 1.13 (1.01-1.27) |
| NOMO1 | Inverse variance weighted | 11 | -0.12 | 0.06 | 3.76E-02 | 0.89 (0.8-0.99) |
| CD34 | Inverse variance weighted | 10 | 0.10 | 0.05 | 3.80E-02 | 1.11 (1.01-1.22) |
| CSRP3 | Inverse variance weighted | 3 | 0.43 | 0.21 | 3.82E-02 | 1.53 (1.02-2.29) |
| ICAM4 | Inverse variance weighted | 32 | 0.07 | 0.03 | 3.83E-02 | 1.07 (1-1.15) |
| SIT1 | Inverse variance weighted | 9 | -0.19 | 0.09 | 3.86E-02 | 0.83 (0.69-0.99) |
| APOH | Inverse variance weighted | 6 | 0.08 | 0.04 | 3.95E-02 | 1.09 (1-1.18) |
| DTNB | Inverse variance weighted | 4 | 0.43 | 0.21 | 3.98E-02 | 1.54 (1.02-2.34) |
| NACC1 | Wald ratio | 1 | -0.80 | 0.39 | 4.14E-02 | 0.45 (0.21-0.97) |
| IL3RA | Inverse variance weighted | 7 | 0.13 | 0.06 | 4.20E-02 | 1.14 (1-1.3) |
| IGF2R | Inverse variance weighted | 22 | 0.09 | 0.04 | 4.22E-02 | 1.09 (1-1.19) |
| ENG | Inverse variance weighted | 13 | 0.07 | 0.03 | 4.27E-02 | 1.07 (1-1.15) |
| PDGFB | Inverse variance weighted | 28 | -0.09 | 0.05 | 4.29E-02 | 0.91 (0.83-1) |
| GAS2 | Inverse variance weighted | 3 | 0.28 | 0.14 | 4.36E-02 | 1.32 (1.01-1.74) |
| ICA1 | Inverse variance weighted | 5 | 0.14 | 0.07 | 4.45E-02 | 1.16 (1-1.33) |
| ECE1 | Inverse variance weighted | 9 | 0.13 | 0.06 | 4.57E-02 | 1.14 (1-1.29) |
| CSPG5 | Wald ratio | 1 | -0.66 | 0.33 | 4.62E-02 | 0.52 (0.27-0.99) |
| XRCC4 | Wald ratio | 1 | 0.48 | 0.24 | 4.65E-02 | 1.62 (1.01-2.6) |
| GNPDA2 | Inverse variance weighted | 8 | -0.10 | 0.05 | 4.66E-02 | 0.9 (0.82-1) |
| ADAM23 | Inverse variance weighted | 18 | 0.07 | 0.04 | 4.67E-02 | 1.07 (1-1.15) |
| WWP2 | Inverse variance weighted | 6 | -0.16 | 0.08 | 4.70E-02 | 0.85 (0.73-1) |
| CLEC1A | Inverse variance weighted | 15 | 0.09 | 0.04 | 4.76E-02 | 1.09 (1-1.19) |
| PIKFYVE | Inverse variance weighted | 5 | 0.60 | 0.30 | 4.86E-02 | 1.82 (1-3.29) |
| LYAR | Wald ratio | 1 | 0.66 | 0.34 | 4.87E-02 | 1.94 (1-3.74) |
| TPSD1 | Inverse variance weighted | 7 | 0.14 | 0.07 | 4.91E-02 | 1.15 (1-1.33) |
| MMP9 | Inverse variance weighted | 14 | -0.15 | 0.07 | 4.91E-02 | 0.86 (0.75-1) |

| **Supplementary table 7. Two-sample Mendelian randomization results for lung squamous cell carcinoma.** | | | | | | |
| --- | --- | --- | --- | --- | --- | --- |
| **Protein** | **Method** | **Nsnp** | **Beta** | **SE** | ***P*** | **OR (95%CI)** |
| ATP6V1G2 | Wald ratio | 1 | -2.21 | 0.42 | 1.02E-07 | 0.11 (0.05-0.25) |
| IFNL1 | Inverse variance weighted | 10 | -0.59 | 0.14 | 1.58E-05 | 0.55 (0.42-0.72) |
| SIGLEC1 | Inverse variance weighted | 21 | -0.27 | 0.07 | 1.10E-04 | 0.77 (0.67-0.88) |
| LRP1 | Inverse variance weighted | 14 | 0.09 | 0.03 | 2.63E-04 | 1.1 (1.04-1.15) |
| NOTCH2 | Inverse variance weighted | 6 | -0.45 | 0.13 | 3.83E-04 | 0.64 (0.5-0.82) |
| USP28 | Inverse variance weighted | 2 | 0.86 | 0.25 | 5.86E-04 | 2.35 (1.44-3.83) |
| CTHRC1 | Inverse variance weighted | 10 | 0.46 | 0.14 | 7.38E-04 | 1.58 (1.21-2.07) |
| NECTIN2 | Inverse variance weighted | 9 | 0.35 | 0.11 | 9.33E-04 | 1.42 (1.15-1.74) |
| SPINK2 | Inverse variance weighted | 17 | -0.15 | 0.04 | 1.02E-03 | 0.86 (0.79-0.94) |
| LRPAP1 | Inverse variance weighted | 20 | 0.09 | 0.03 | 1.02E-03 | 1.09 (1.04-1.16) |
| PGF | Inverse variance weighted | 3 | -0.61 | 0.19 | 1.07E-03 | 0.54 (0.38-0.78) |
| DAAM1 | Inverse variance weighted | 10 | -0.36 | 0.11 | 1.09E-03 | 0.7 (0.56-0.87) |
| MICB_MICA | Inverse variance weighted | 17 | 0.33 | 0.10 | 1.43E-03 | 1.39 (1.13-1.7) |
| PLA2G7 | Inverse variance weighted | 18 | -0.19 | 0.06 | 1.79E-03 | 0.83 (0.74-0.93) |
| CD300C | Inverse variance weighted | 24 | -0.11 | 0.03 | 1.80E-03 | 0.9 (0.84-0.96) |
| SIGLEC8 | Inverse variance weighted | 18 | -0.14 | 0.05 | 2.20E-03 | 0.87 (0.79-0.95) |
| CARHSP1 | Inverse variance weighted | 2 | 0.64 | 0.21 | 2.46E-03 | 1.9 (1.25-2.87) |
| GSTT2B | Inverse variance weighted | 7 | 0.08 | 0.03 | 3.33E-03 | 1.09 (1.03-1.15) |
| CETN3 | Inverse variance weighted | 15 | 0.20 | 0.07 | 3.47E-03 | 1.22 (1.07-1.39) |
| ADAMTSL4 | Inverse variance weighted | 8 | -0.23 | 0.08 | 3.59E-03 | 0.8 (0.68-0.93) |
| SGSH | Inverse variance weighted | 19 | 0.08 | 0.03 | 3.71E-03 | 1.09 (1.03-1.15) |
| RPL14 | Inverse variance weighted | 3 | 0.64 | 0.22 | 3.81E-03 | 1.9 (1.23-2.94) |
| ROBO4 | Inverse variance weighted | 5 | 0.12 | 0.04 | 4.02E-03 | 1.12 (1.04-1.21) |
| IL1RN | Inverse variance weighted | 9 | -0.19 | 0.07 | 4.41E-03 | 0.83 (0.72-0.94) |
| BTN2A1 | Inverse variance weighted | 18 | 0.32 | 0.11 | 4.88E-03 | 1.37 (1.1-1.71) |
| CLEC3B | Inverse variance weighted | 6 | 0.22 | 0.08 | 5.03E-03 | 1.24 (1.07-1.44) |
| CD160 | Inverse variance weighted | 24 | -0.13 | 0.04 | 5.08E-03 | 0.88 (0.81-0.96) |
| CDSN | Inverse variance weighted | 29 | 0.23 | 0.08 | 5.11E-03 | 1.26 (1.07-1.48) |
| RCC1 | Wald ratio | 1 | 1.18 | 0.42 | 5.24E-03 | 3.24 (1.42-7.41) |
| SNAP25 | Inverse variance weighted | 14 | -0.15 | 0.05 | 5.86E-03 | 0.86 (0.78-0.96) |
| KRT18 | Inverse variance weighted | 13 | -0.25 | 0.09 | 5.94E-03 | 0.78 (0.65-0.93) |
| PBXIP1 | Inverse variance weighted | 2 | 0.79 | 0.29 | 6.37E-03 | 2.2 (1.25-3.89) |
| BST2 | Inverse variance weighted | 24 | -0.19 | 0.07 | 6.79E-03 | 0.83 (0.72-0.95) |
| LY9 | Inverse variance weighted | 21 | -0.12 | 0.04 | 7.16E-03 | 0.89 (0.81-0.97) |
| F2 | Inverse variance weighted | 4 | 0.39 | 0.15 | 7.66E-03 | 1.47 (1.11-1.96) |
| CKMT1A_CKMT1B | Inverse variance weighted | 8 | 0.24 | 0.09 | 8.08E-03 | 1.28 (1.07-1.53) |
| VSIR | Inverse variance weighted | 14 | -0.15 | 0.06 | 8.10E-03 | 0.86 (0.77-0.96) |
| COL9A2 | Wald ratio | 1 | -1.18 | 0.45 | 8.40E-03 | 0.31 (0.13-0.74) |
| CEND1 | Wald ratio | 1 | -0.96 | 0.37 | 8.69E-03 | 0.38 (0.19-0.78) |
| AIF1L | Inverse variance weighted | 3 | -0.19 | 0.07 | 8.79E-03 | 0.83 (0.72-0.95) |
| MZT1 | Inverse variance weighted | 2 | 0.64 | 0.25 | 9.93E-03 | 1.9 (1.17-3.1) |
| INSL4 | Inverse variance weighted | 7 | 0.24 | 0.09 | 9.99E-03 | 1.27 (1.06-1.51) |
| IL2RB | Inverse variance weighted | 2 | -0.82 | 0.32 | 1.00E-02 | 0.44 (0.24-0.82) |
| CDKN1A | Inverse variance weighted | 10 | -0.23 | 0.09 | 1.04E-02 | 0.79 (0.66-0.95) |
| CD84 | Inverse variance weighted | 19 | -0.15 | 0.06 | 1.05E-02 | 0.86 (0.77-0.97) |
| FNTA | Inverse variance weighted | 2 | 0.73 | 0.29 | 1.06E-02 | 2.08 (1.19-3.65) |
| GP5 | Inverse variance weighted | 12 | -0.24 | 0.10 | 1.10E-02 | 0.78 (0.65-0.95) |
| RNASET2 | Inverse variance weighted | 19 | 0.21 | 0.08 | 1.12E-02 | 1.23 (1.05-1.44) |
| GALNT10 | Inverse variance weighted | 12 | -0.12 | 0.05 | 1.12E-02 | 0.89 (0.81-0.97) |
| BTN3A2 | Inverse variance weighted | 15 | -0.17 | 0.07 | 1.12E-02 | 0.85 (0.75-0.96) |
| C5 | Inverse variance weighted | 2 | -0.31 | 0.12 | 1.15E-02 | 0.74 (0.58-0.93) |
| PLAT | Inverse variance weighted | 5 | -0.37 | 0.15 | 1.16E-02 | 0.69 (0.52-0.92) |
| PLXNB3 | Inverse variance weighted | 12 | -0.27 | 0.11 | 1.20E-02 | 0.77 (0.62-0.94) |
| PILRA | Inverse variance weighted | 27 | -0.09 | 0.03 | 1.24E-02 | 0.92 (0.86-0.98) |
| ROBO2 | Inverse variance weighted | 14 | 0.25 | 0.10 | 1.24E-02 | 1.28 (1.06-1.56) |
| CD14 | Inverse variance weighted | 11 | 0.16 | 0.07 | 1.26E-02 | 1.18 (1.04-1.34) |
| MDM1 | Wald ratio | 1 | 0.25 | 0.10 | 1.34E-02 | 1.29 (1.05-1.58) |
| BDNF | Inverse variance weighted | 29 | -0.14 | 0.06 | 1.43E-02 | 0.87 (0.78-0.97) |
| PKLR | Inverse variance weighted | 8 | -0.21 | 0.09 | 1.45E-02 | 0.81 (0.68-0.96) |
| PTGR1 | Inverse variance weighted | 14 | -0.10 | 0.04 | 1.49E-02 | 0.9 (0.83-0.98) |
| CGREF1 | Inverse variance weighted | 17 | 0.09 | 0.04 | 1.51E-02 | 1.1 (1.02-1.18) |
| CLMP | Inverse variance weighted | 8 | 0.17 | 0.07 | 1.55E-02 | 1.18 (1.03-1.35) |
| ARF6 | Inverse variance weighted | 5 | 0.48 | 0.20 | 1.58E-02 | 1.61 (1.09-2.37) |
| UBE2L6 | Inverse variance weighted | 8 | 0.17 | 0.07 | 1.58E-02 | 1.19 (1.03-1.37) |
| CXCL3 | Inverse variance weighted | 6 | -0.38 | 0.16 | 1.60E-02 | 0.69 (0.51-0.93) |
| DMP1 | Inverse variance weighted | 20 | -0.18 | 0.08 | 1.64E-02 | 0.83 (0.72-0.97) |
| CDON | Inverse variance weighted | 8 | -0.25 | 0.10 | 1.67E-02 | 0.78 (0.64-0.96) |
| SYT1 | Wald ratio | 1 | -1.12 | 0.47 | 1.70E-02 | 0.33 (0.13-0.82) |
| PTPRC | Inverse variance weighted | 16 | -0.18 | 0.07 | 1.71E-02 | 0.84 (0.72-0.97) |
| CD80 | Inverse variance weighted | 19 | -0.10 | 0.04 | 1.76E-02 | 0.91 (0.84-0.98) |
| FGF21 | Inverse variance weighted | 8 | -0.25 | 0.11 | 1.78E-02 | 0.78 (0.63-0.96) |
| VWC2L | Inverse variance weighted | 2 | 0.52 | 0.22 | 1.78E-02 | 1.69 (1.09-2.61) |
| TRAF2 | Inverse variance weighted | 3 | 0.43 | 0.18 | 1.79E-02 | 1.54 (1.08-2.21) |
| PBK | Wald ratio | 1 | 1.16 | 0.49 | 1.80E-02 | 3.19 (1.22-8.36) |
| MPRIP | Wald ratio | 1 | 0.74 | 0.31 | 1.82E-02 | 2.1 (1.13-3.87) |
| SIAE | Inverse variance weighted | 17 | -0.18 | 0.08 | 1.82E-02 | 0.84 (0.72-0.97) |
| PF4 | Inverse variance weighted | 20 | -0.15 | 0.06 | 1.83E-02 | 0.86 (0.76-0.97) |
| CD72 | Inverse variance weighted | 18 | -0.18 | 0.08 | 1.89E-02 | 0.84 (0.72-0.97) |
| TIMP1 | Inverse variance weighted | 12 | -0.22 | 0.09 | 1.96E-02 | 0.8 (0.67-0.97) |
| MEP1A | Inverse variance weighted | 29 | 0.09 | 0.04 | 2.03E-02 | 1.09 (1.01-1.17) |
| PDGFB | Inverse variance weighted | 29 | -0.12 | 0.05 | 2.14E-02 | 0.88 (0.8-0.98) |
| FARSA | Inverse variance weighted | 4 | 0.49 | 0.21 | 2.15E-02 | 1.64 (1.08-2.5) |
| LGALS1 | Inverse variance weighted | 5 | 0.21 | 0.09 | 2.18E-02 | 1.23 (1.03-1.47) |
| RNF43 | Inverse variance weighted | 4 | -0.48 | 0.21 | 2.18E-02 | 0.62 (0.41-0.93) |
| SCGN | Inverse variance weighted | 12 | 0.18 | 0.08 | 2.19E-02 | 1.2 (1.03-1.4) |
| CCL18 | Inverse variance weighted | 10 | -0.15 | 0.07 | 2.22E-02 | 0.86 (0.76-0.98) |
| DAG1 | Inverse variance weighted | 11 | -0.27 | 0.12 | 2.22E-02 | 0.76 (0.61-0.96) |
| EPHB4 | Inverse variance weighted | 18 | 0.11 | 0.05 | 2.25E-02 | 1.11 (1.01-1.22) |
| NEO1 | Wald ratio | 1 | -0.63 | 0.28 | 2.30E-02 | 0.53 (0.31-0.92) |
| RNF41 | Inverse variance weighted | 2 | 0.63 | 0.28 | 2.30E-02 | 1.89 (1.09-3.26) |
| CPA4 | Inverse variance weighted | 15 | 0.14 | 0.06 | 2.40E-02 | 1.15 (1.02-1.3) |
| **EDA2R** | Inverse variance weighted | 12 | 0.46 | 0.21 | 2.43E-02 | 1.59 (1.06-2.38) |
| ECHDC3 | Inverse variance weighted | 13 | -0.11 | 0.05 | 2.51E-02 | 0.9 (0.82-0.99) |
| CDC27 | Inverse variance weighted | 2 | 0.65 | 0.29 | 2.53E-02 | 1.91 (1.08-3.35) |
| CDH1 | Inverse variance weighted | 16 | 0.09 | 0.04 | 2.54E-02 | 1.1 (1.01-1.19) |
| AFM | Inverse variance weighted | 10 | 0.23 | 0.10 | 2.56E-02 | 1.25 (1.03-1.53) |
| COL2A1 | Inverse variance weighted | 38 | -0.08 | 0.03 | 2.57E-02 | 0.93 (0.86-0.99) |
| AKT1S1 | Inverse variance weighted | 2 | 0.49 | 0.22 | 2.61E-02 | 1.64 (1.06-2.53) |
| CNTN2 | Inverse variance weighted | 17 | -0.08 | 0.04 | 2.64E-02 | 0.92 (0.85-0.99) |
| ADAM23 | Inverse variance weighted | 16 | 0.09 | 0.04 | 2.70E-02 | 1.09 (1.01-1.18) |
| CXCL10 | Inverse variance weighted | 11 | -0.32 | 0.15 | 2.73E-02 | 0.72 (0.54-0.96) |
| BCHE | Inverse variance weighted | 23 | -0.14 | 0.06 | 2.74E-02 | 0.87 (0.77-0.98) |
| CPE | Inverse variance weighted | 12 | 0.15 | 0.07 | 2.75E-02 | 1.16 (1.02-1.33) |
| ISM2 | Wald ratio | 1 | 1.00 | 0.45 | 2.75E-02 | 2.72 (1.12-6.64) |
| ITGAL | Inverse variance weighted | 4 | -0.32 | 0.15 | 2.78E-02 | 0.73 (0.55-0.97) |
| FCRL2 | Inverse variance weighted | 31 | -0.10 | 0.05 | 2.82E-02 | 0.9 (0.83-0.99) |
| ZNRF4 | Inverse variance weighted | 5 | -0.22 | 0.10 | 2.86E-02 | 0.8 (0.66-0.98) |
| ITGB2 | Inverse variance weighted | 23 | -0.14 | 0.06 | 2.89E-02 | 0.87 (0.77-0.99) |
| CEACAM20 | Inverse variance weighted | 6 | 0.16 | 0.07 | 2.91E-02 | 1.17 (1.02-1.35) |
| TNFRSF10A | Inverse variance weighted | 11 | 0.19 | 0.09 | 3.09E-02 | 1.21 (1.02-1.43) |
| BACH1 | Inverse variance weighted | 5 | -0.40 | 0.18 | 3.17E-02 | 0.67 (0.47-0.97) |
| GHR | Inverse variance weighted | 13 | 0.17 | 0.08 | 3.18E-02 | 1.19 (1.02-1.39) |
| MLN | Inverse variance weighted | 33 | 0.11 | 0.05 | 3.20E-02 | 1.11 (1.01-1.23) |
| HEPH | Inverse variance weighted | 8 | 0.15 | 0.07 | 3.21E-02 | 1.17 (1.01-1.34) |
| OGFR | Inverse variance weighted | 5 | -0.35 | 0.16 | 3.23E-02 | 0.71 (0.51-0.97) |
| CSNK2A1 | Inverse variance weighted | 11 | -0.18 | 0.08 | 3.28E-02 | 0.84 (0.71-0.99) |
| FCRLB | Inverse variance weighted | 17 | -0.09 | 0.04 | 3.40E-02 | 0.91 (0.84-0.99) |
| OBP2B | Inverse variance weighted | 17 | 0.13 | 0.06 | 3.52E-02 | 1.14 (1.01-1.28) |
| DPP10 | Inverse variance weighted | 9 | 0.13 | 0.06 | 3.58E-02 | 1.14 (1.01-1.29) |
| LGALS9 | Inverse variance weighted | 12 | -0.18 | 0.08 | 3.61E-02 | 0.84 (0.71-0.99) |
| ADAM22 | Inverse variance weighted | 16 | 0.09 | 0.04 | 3.63E-02 | 1.1 (1.01-1.19) |
| GP1BA | Inverse variance weighted | 31 | -0.11 | 0.05 | 3.63E-02 | 0.89 (0.81-0.99) |
| CEP112 | Wald ratio | 1 | -0.61 | 0.29 | 3.65E-02 | 0.54 (0.31-0.96) |
| MYBPC2 | Inverse variance weighted | 3 | -0.54 | 0.26 | 3.67E-02 | 0.58 (0.35-0.97) |
| CPXM1 | Inverse variance weighted | 35 | -0.09 | 0.04 | 3.71E-02 | 0.92 (0.85-0.99) |
| GFRA2 | Inverse variance weighted | 11 | -0.13 | 0.06 | 3.72E-02 | 0.88 (0.77-0.99) |
| C7 | Inverse variance weighted | 14 | 0.11 | 0.05 | 3.74E-02 | 1.11 (1.01-1.23) |
| SKAP1 | Inverse variance weighted | 12 | -0.23 | 0.11 | 3.79E-02 | 0.8 (0.64-0.99) |
| ACAN | Inverse variance weighted | 24 | -0.13 | 0.06 | 3.82E-02 | 0.88 (0.78-0.99) |
| BTC | Inverse variance weighted | 11 | 0.17 | 0.08 | 3.83E-02 | 1.18 (1.01-1.39) |
| SIRPB1 | Inverse variance weighted | 22 | -0.08 | 0.04 | 3.84E-02 | 0.92 (0.85-1) |
| AHSG | Inverse variance weighted | 10 | -0.08 | 0.04 | 3.86E-02 | 0.92 (0.85-1) |
| TREML2 | Inverse variance weighted | 20 | -0.12 | 0.06 | 3.88E-02 | 0.88 (0.79-0.99) |
| INPP5J | Inverse variance weighted | 4 | -0.53 | 0.26 | 3.90E-02 | 0.59 (0.35-0.97) |
| ECHS1 | Inverse variance weighted | 8 | -0.17 | 0.08 | 3.91E-02 | 0.84 (0.72-0.99) |
| BAMBI | Inverse variance weighted | 3 | -0.46 | 0.22 | 3.93E-02 | 0.63 (0.4-0.98) |
| IL1R1 | Inverse variance weighted | 27 | 0.10 | 0.05 | 3.96E-02 | 1.11 (1-1.22) |
| CEP20 | Inverse variance weighted | 7 | 0.24 | 0.12 | 4.01E-02 | 1.27 (1.01-1.6) |
| LRCH4 | Inverse variance weighted | 7 | -0.34 | 0.17 | 4.02E-02 | 0.71 (0.51-0.98) |
| PPCDC | Inverse variance weighted | 4 | 0.15 | 0.07 | 4.25E-02 | 1.16 (1.01-1.35) |
| ANGPT1 | Inverse variance weighted | 35 | -0.11 | 0.05 | 4.25E-02 | 0.9 (0.81-1) |
| SEPTIN3 | Inverse variance weighted | 12 | 0.20 | 0.10 | 4.26E-02 | 1.23 (1.01-1.49) |
| SORD | Inverse variance weighted | 6 | -0.23 | 0.12 | 4.28E-02 | 0.79 (0.63-0.99) |
| USP8 | Inverse variance weighted | 8 | 0.21 | 0.11 | 4.28E-02 | 1.24 (1.01-1.53) |
| SULT1A1 | Inverse variance weighted | 11 | -0.21 | 0.10 | 4.31E-02 | 0.81 (0.66-0.99) |
| CXCL16 | Inverse variance weighted | 21 | 0.12 | 0.06 | 4.37E-02 | 1.12 (1-1.26) |
| XRCC4 | Wald ratio | 1 | 0.56 | 0.28 | 4.52E-02 | 1.74 (1.01-3) |
| IFNLR1 | Inverse variance weighted | 5 | 0.13 | 0.06 | 4.55E-02 | 1.14 (1-1.29) |
| F11R | Inverse variance weighted | 13 | -0.19 | 0.09 | 4.56E-02 | 0.83 (0.69-1) |
| BNIP3L | Inverse variance weighted | 6 | -0.32 | 0.16 | 4.60E-02 | 0.73 (0.53-0.99) |
| SERPINI2 | Inverse variance weighted | 35 | -0.08 | 0.04 | 4.66E-02 | 0.92 (0.85-1) |
| CA14 | Inverse variance weighted | 12 | 0.29 | 0.14 | 4.68E-02 | 1.33 (1-1.76) |
| GPD1 | Wald ratio | 1 | -0.73 | 0.37 | 4.70E-02 | 0.48 (0.23-0.99) |
| FKBP7 | Wald ratio | 1 | -0.58 | 0.29 | 4.77E-02 | 0.56 (0.32-0.99) |
| PDGFA | Inverse variance weighted | 29 | -0.10 | 0.05 | 4.81E-02 | 0.9 (0.82-1) |
| CKAP4 | Inverse variance weighted | 16 | 0.13 | 0.07 | 4.86E-02 | 1.14 (1-1.3) |
| YJU2 | Inverse variance weighted | 3 | -0.48 | 0.24 | 4.88E-02 | 0.62 (0.38-1) |
| CRISP2 | Inverse variance weighted | 27 | -0.09 | 0.04 | 4.96E-02 | 0.92 (0.84-1) |

| **Supplementary table 8. Two-sample Mendelian randomization results for lung small cell carcinoma.** | | | | | | |
| --- | --- | --- | --- | --- | --- | --- |
| **Protein** | **Method** | **Nsnp** | **Beta** | **SE** | ***P*** | **OR (95%CI)** |
| BTN3A2 | Inverse variance weighted | 13 | -0.44 | 0.09 | 1.99E-06 | 0.64 (0.54-0.77) |
| BTN1A1 | Wald ratio | 1 | -0.79 | 0.17 | 3.14E-06 | 0.45 (0.33-0.63) |
| CRX | Wald ratio | 1 | 2.85 | 0.65 | 9.91E-06 | 17.31 (4.89-61.3) |
| SF3B4 | Inverse variance weighted | 6 | 0.80 | 0.24 | 7.09E-04 | 2.22 (1.4-3.53) |
| IFNL1 | Inverse variance weighted | 9 | -0.60 | 0.20 | 2.32E-03 | 0.55 (0.37-0.81) |
| DDI2 | Wald ratio | 1 | -1.99 | 0.66 | 2.52E-03 | 0.14 (0.04-0.5) |
| PRDX2 | Wald ratio | 1 | -1.56 | 0.52 | 2.52E-03 | 0.21 (0.08-0.58) |
| CTSV | Inverse variance weighted | 30 | 0.23 | 0.08 | 2.79E-03 | 1.26 (1.08-1.46) |
| MAMDC4 | Inverse variance weighted | 6 | -0.32 | 0.11 | 2.82E-03 | 0.73 (0.59-0.9) |
| GALNT3 | Inverse variance weighted | 16 | -0.18 | 0.06 | 3.88E-03 | 0.84 (0.74-0.94) |
| ECM1 | Inverse variance weighted | 6 | -0.23 | 0.08 | 3.90E-03 | 0.79 (0.68-0.93) |
| CGB3_CGB5_CGB8 | Inverse variance weighted | 3 | -0.92 | 0.32 | 4.74E-03 | 0.4 (0.21-0.76) |
| CEACAM6 | Inverse variance weighted | 32 | 0.24 | 0.08 | 4.89E-03 | 1.27 (1.07-1.49) |
| LPP | Inverse variance weighted | 4 | -0.83 | 0.30 | 5.00E-03 | 0.44 (0.24-0.78) |
| CR2 | Inverse variance weighted | 38 | 0.22 | 0.08 | 6.50E-03 | 1.24 (1.06-1.46) |
| CEND1 | Wald ratio | 1 | -1.59 | 0.58 | 6.65E-03 | 0.2 (0.07-0.64) |
| RNASE10 | Inverse variance weighted | 6 | 0.16 | 0.06 | 7.09E-03 | 1.18 (1.05-1.33) |
| EPO | Inverse variance weighted | 13 | 0.33 | 0.12 | 7.22E-03 | 1.4 (1.09-1.78) |
| MEGF9 | Inverse variance weighted | 17 | 0.23 | 0.09 | 7.38E-03 | 1.26 (1.06-1.5) |
| PXN | Inverse variance weighted | 8 | -0.20 | 0.08 | 7.40E-03 | 0.82 (0.7-0.95) |
| MMP10 | Inverse variance weighted | 6 | 0.26 | 0.10 | 8.06E-03 | 1.3 (1.07-1.57) |
| STC2 | Inverse variance weighted | 15 | -0.41 | 0.16 | 8.46E-03 | 0.66 (0.49-0.9) |
| FAM171B | Inverse variance weighted | 5 | 0.27 | 0.10 | 8.49E-03 | 1.31 (1.07-1.6) |
| CCL23 | Inverse variance weighted | 9 | -0.26 | 0.10 | 8.57E-03 | 0.77 (0.64-0.94) |
| ITGBL1 | Inverse variance weighted | 8 | -0.23 | 0.09 | 8.62E-03 | 0.8 (0.67-0.94) |
| RP2 | Inverse variance weighted | 4 | -0.63 | 0.24 | 9.01E-03 | 0.54 (0.33-0.86) |
| QSOX1 | Inverse variance weighted | 12 | -0.20 | 0.08 | 9.05E-03 | 0.82 (0.71-0.95) |
| LCP1 | Inverse variance weighted | 7 | 0.16 | 0.06 | 9.11E-03 | 1.18 (1.04-1.33) |
| DIPK1C | Wald ratio | 1 | 1.76 | 0.68 | 9.52E-03 | 5.83 (1.54-22.09) |
| PDZD2 | Inverse variance weighted | 3 | 0.35 | 0.13 | 1.01E-02 | 1.41 (1.09-1.84) |
| TNFRSF10B | Inverse variance weighted | 8 | -0.28 | 0.11 | 1.05E-02 | 0.75 (0.61-0.94) |
| PRRT3 | Inverse variance weighted | 12 | 0.31 | 0.12 | 1.06E-02 | 1.37 (1.08-1.74) |
| IL18 | Inverse variance weighted | 16 | -0.21 | 0.08 | 1.07E-02 | 0.81 (0.68-0.95) |
| CA1 | Inverse variance weighted | 5 | -0.52 | 0.20 | 1.12E-02 | 0.6 (0.4-0.89) |
| CXCL10 | Inverse variance weighted | 8 | -0.47 | 0.19 | 1.28E-02 | 0.62 (0.43-0.9) |
| OSMR | Inverse variance weighted | 14 | -0.20 | 0.08 | 1.28E-02 | 0.82 (0.7-0.96) |
| SBSN | Inverse variance weighted | 11 | 0.32 | 0.13 | 1.33E-02 | 1.38 (1.07-1.78) |
| MEGF10 | Inverse variance weighted | 30 | 0.15 | 0.06 | 1.36E-02 | 1.16 (1.03-1.31) |
| C7orf50 | Inverse variance weighted | 2 | 0.41 | 0.17 | 1.36E-02 | 1.51 (1.09-2.1) |
| EPHB6 | Inverse variance weighted | 7 | 0.24 | 0.10 | 1.38E-02 | 1.27 (1.05-1.53) |
| CTSB | Inverse variance weighted | 12 | -0.19 | 0.08 | 1.44E-02 | 0.83 (0.71-0.96) |
| FOLH1 | Inverse variance weighted | 6 | -0.68 | 0.28 | 1.45E-02 | 0.51 (0.29-0.87) |
| PBLD | Inverse variance weighted | 3 | 0.38 | 0.16 | 1.50E-02 | 1.46 (1.08-1.99) |
| TXNDC15 | Inverse variance weighted | 19 | -0.17 | 0.07 | 1.55E-02 | 0.84 (0.73-0.97) |
| IL12RB2 | Inverse variance weighted | 2 | -1.72 | 0.71 | 1.56E-02 | 0.18 (0.04-0.72) |
| PM20D1 | Inverse variance weighted | 15 | 0.14 | 0.06 | 1.70E-02 | 1.15 (1.02-1.28) |
| NCAN | Inverse variance weighted | 20 | -0.25 | 0.10 | 1.71E-02 | 0.78 (0.64-0.96) |
| PGLYRP4 | Inverse variance weighted | 8 | -0.23 | 0.10 | 1.90E-02 | 0.79 (0.65-0.96) |
| VSIG10L | Inverse variance weighted | 3 | -0.32 | 0.14 | 1.95E-02 | 0.73 (0.55-0.95) |
| AP1G2 | Inverse variance weighted | 7 | -0.48 | 0.20 | 1.98E-02 | 0.62 (0.42-0.93) |
| CXCL16 | Inverse variance weighted | 20 | 0.22 | 0.10 | 1.98E-02 | 1.25 (1.04-1.51) |
| A1BG | Inverse variance weighted | 10 | -0.32 | 0.14 | 2.00E-02 | 0.73 (0.55-0.95) |
| FOLR1 | Inverse variance weighted | 10 | 0.32 | 0.14 | 2.03E-02 | 1.38 (1.05-1.82) |
| ADAMTSL4 | Inverse variance weighted | 7 | -0.29 | 0.13 | 2.06E-02 | 0.74 (0.58-0.96) |
| YES1 | Inverse variance weighted | 6 | -0.53 | 0.23 | 2.06E-02 | 0.59 (0.37-0.92) |
| MENT | Inverse variance weighted | 19 | -0.17 | 0.07 | 2.12E-02 | 0.85 (0.74-0.98) |
| VAT1 | Inverse variance weighted | 15 | 0.25 | 0.11 | 2.14E-02 | 1.29 (1.04-1.6) |
| CPXM2 | Inverse variance weighted | 6 | 0.27 | 0.12 | 2.16E-02 | 1.31 (1.04-1.66) |
| SDC1 | Inverse variance weighted | 22 | 0.23 | 0.10 | 2.18E-02 | 1.26 (1.03-1.55) |
| CHGA | Inverse variance weighted | 9 | -0.28 | 0.12 | 2.24E-02 | 0.76 (0.6-0.96) |
| MEGF11 | Inverse variance weighted | 3 | 0.38 | 0.17 | 2.34E-02 | 1.46 (1.05-2.02) |
| SNAP25 | Inverse variance weighted | 12 | -0.18 | 0.08 | 2.36E-02 | 0.83 (0.71-0.98) |
| ATXN3 | Inverse variance weighted | 4 | -0.27 | 0.12 | 2.38E-02 | 0.77 (0.61-0.97) |
| HNMT | Inverse variance weighted | 7 | -0.25 | 0.11 | 2.49E-02 | 0.78 (0.63-0.97) |
| GDNF | Inverse variance weighted | 6 | -0.32 | 0.14 | 2.52E-02 | 0.72 (0.55-0.96) |
| MED18 | Inverse variance weighted | 4 | -0.65 | 0.29 | 2.56E-02 | 0.52 (0.3-0.92) |
| GSR | Inverse variance weighted | 3 | 0.49 | 0.22 | 2.63E-02 | 1.64 (1.06-2.54) |
| GKN1 | Inverse variance weighted | 3 | -0.59 | 0.27 | 2.74E-02 | 0.56 (0.33-0.94) |
| ACADSB | Inverse variance weighted | 9 | -0.23 | 0.11 | 2.75E-02 | 0.79 (0.64-0.97) |
| DSG3 | Inverse variance weighted | 24 | 0.17 | 0.08 | 2.81E-02 | 1.19 (1.02-1.39) |
| APOF | Inverse variance weighted | 13 | -0.28 | 0.13 | 2.82E-02 | 0.76 (0.59-0.97) |
| PSMD9 | Wald ratio | 1 | 0.37 | 0.17 | 2.83E-02 | 1.44 (1.04-2) |
| REG3G | Inverse variance weighted | 5 | -0.24 | 0.11 | 2.88E-02 | 0.79 (0.63-0.98) |
| BPIFB1 | Inverse variance weighted | 30 | 0.16 | 0.07 | 3.01E-02 | 1.17 (1.02-1.35) |
| PLG | Inverse variance weighted | 16 | -0.23 | 0.11 | 3.12E-02 | 0.8 (0.65-0.98) |
| ACTA2 | Wald ratio | 1 | 1.06 | 0.49 | 3.24E-02 | 2.87 (1.09-7.55) |
| DUSP13 | Inverse variance weighted | 2 | -0.94 | 0.44 | 3.38E-02 | 0.39 (0.17-0.93) |
| CRTAM | Inverse variance weighted | 24 | 0.21 | 0.10 | 3.38E-02 | 1.24 (1.02-1.5) |
| GALNT7 | Inverse variance weighted | 10 | -0.19 | 0.09 | 3.38E-02 | 0.83 (0.7-0.99) |
| LY9 | Inverse variance weighted | 19 | -0.14 | 0.07 | 3.39E-02 | 0.87 (0.76-0.99) |
| CD46 | Inverse variance weighted | 7 | 0.38 | 0.18 | 3.46E-02 | 1.47 (1.03-2.09) |
| HDGF | Inverse variance weighted | 7 | -0.27 | 0.13 | 3.53E-02 | 0.76 (0.6-0.98) |
| GSTA1 | Inverse variance weighted | 10 | -0.20 | 0.10 | 3.67E-02 | 0.82 (0.67-0.99) |
| ENTR1 | Inverse variance weighted | 4 | -0.24 | 0.12 | 3.69E-02 | 0.79 (0.63-0.99) |
| TSPAN15 | Wald ratio | 1 | -1.10 | 0.53 | 3.75E-02 | 0.33 (0.12-0.94) |
| GPRC5C | Inverse variance weighted | 11 | -0.33 | 0.16 | 3.76E-02 | 0.72 (0.53-0.98) |
| MBL2 | Inverse variance weighted | 14 | 0.13 | 0.06 | 3.78E-02 | 1.14 (1.01-1.29) |
| CFC1 | Inverse variance weighted | 10 | 0.39 | 0.19 | 3.78E-02 | 1.48 (1.02-2.15) |
| POMC | Inverse variance weighted | 17 | 0.20 | 0.10 | 3.92E-02 | 1.22 (1.01-1.47) |
| FKBP5 | Inverse variance weighted | 7 | -0.27 | 0.13 | 3.92E-02 | 0.76 (0.59-0.99) |
| ADGRG1 | Inverse variance weighted | 16 | -0.28 | 0.13 | 3.96E-02 | 0.76 (0.58-0.99) |
| UNG | Wald ratio | 1 | 1.50 | 0.73 | 3.98E-02 | 4.48 (1.07-18.69) |
| GGT5 | Inverse variance weighted | 6 | -0.22 | 0.11 | 4.03E-02 | 0.81 (0.66-0.99) |
| CPE | Inverse variance weighted | 12 | 0.26 | 0.13 | 4.04E-02 | 1.3 (1.01-1.67) |
| DUSP3 | Inverse variance weighted | 2 | -0.74 | 0.36 | 4.08E-02 | 0.47 (0.23-0.97) |
| MNDA | Inverse variance weighted | 2 | -0.96 | 0.47 | 4.11E-02 | 0.38 (0.15-0.96) |
| EPCAM | Inverse variance weighted | 26 | -0.17 | 0.08 | 4.11E-02 | 0.85 (0.72-0.99) |
| MICB_MICA | Inverse variance weighted | 18 | 0.25 | 0.12 | 4.12E-02 | 1.28 (1.01-1.62) |
| SPAG1 | Inverse variance weighted | 5 | 0.19 | 0.10 | 4.15E-02 | 1.21 (1.01-1.46) |
| LTA4H | Wald ratio | 1 | 0.62 | 0.31 | 4.21E-02 | 1.86 (1.02-3.4) |
| TMEM106A | Inverse variance weighted | 19 | -0.20 | 0.10 | 4.22E-02 | 0.82 (0.68-0.99) |
| SYT1 | Wald ratio | 1 | -1.51 | 0.75 | 4.27E-02 | 0.22 (0.05-0.95) |
| NFATC1 | Inverse variance weighted | 5 | -0.44 | 0.22 | 4.29E-02 | 0.65 (0.42-0.99) |
| NGRN | Inverse variance weighted | 5 | 0.54 | 0.27 | 4.31E-02 | 1.72 (1.02-2.91) |
| CA5A | Inverse variance weighted | 7 | -0.55 | 0.27 | 4.34E-02 | 0.58 (0.34-0.98) |
| CDK1 | Wald ratio | 1 | 0.74 | 0.37 | 4.35E-02 | 2.09 (1.02-4.28) |
| COX6B1 | Inverse variance weighted | 2 | -0.85 | 0.42 | 4.39E-02 | 0.43 (0.19-0.98) |
| STAU1 | Inverse variance weighted | 2 | -0.65 | 0.32 | 4.52E-02 | 0.52 (0.28-0.99) |
| SLC4A1 | Inverse variance weighted | 2 | 0.80 | 0.40 | 4.56E-02 | 2.23 (1.02-4.88) |
| NID2 | Inverse variance weighted | 26 | 0.13 | 0.07 | 4.57E-02 | 1.14 (1-1.3) |
| CELSR2 | Inverse variance weighted | 7 | 0.17 | 0.08 | 4.59E-02 | 1.18 (1-1.4) |
| GPA33 | Inverse variance weighted | 30 | -0.13 | 0.06 | 4.60E-02 | 0.88 (0.78-1) |
| LRRC25 | Inverse variance weighted | 27 | 0.11 | 0.06 | 4.60E-02 | 1.12 (1-1.24) |
| MAD1L1 | Inverse variance weighted | 4 | -0.44 | 0.22 | 4.62E-02 | 0.64 (0.42-0.99) |
| ANXA4 | Inverse variance weighted | 4 | -0.55 | 0.28 | 4.72E-02 | 0.58 (0.33-0.99) |
| SMAD2 | Inverse variance weighted | 7 | -0.57 | 0.29 | 4.73E-02 | 0.56 (0.32-0.99) |
| MELTF | Inverse variance weighted | 13 | 0.16 | 0.08 | 4.75E-02 | 1.17 (1-1.37) |
| KITLG | Inverse variance weighted | 25 | 0.14 | 0.07 | 4.76E-02 | 1.15 (1-1.33) |
| BTN2A1 | Inverse variance weighted | 15 | 0.25 | 0.13 | 4.82E-02 | 1.29 (1-1.65) |
| GSTA3 | Inverse variance weighted | 9 | -0.20 | 0.10 | 4.87E-02 | 0.82 (0.67-1) |
| CPB1 | Inverse variance weighted | 22 | 0.16 | 0.08 | 4.93E-02 | 1.18 (1-1.38) |
| NT5C1A | Inverse variance weighted | 3 | 0.42 | 0.22 | 4.93E-02 | 1.53 (1-2.33) |
| TOR1AIP1 | Inverse variance weighted | 12 | -0.16 | 0.08 | 4.96E-02 | 0.85 (0.73-1) |

| **Supplemental table 9. The results of horizontal pleiotropic and heterogeneity tests for identified proteins on lung adenocarcinoma, squamous cell carcinoma and small cell carcinoma.** | | | | | | | |
| --- | --- | --- | --- | --- | --- | --- | --- |
| **Protein** | **Outcome** | **Q** | **Q_df** | **Q_pval** | **egger_intercept** | **se** | **pval** |
| CD5L | Lung adenocarcinoma | 53.43 | 41 | 0.092 | 0.00 | 0.01 | 0.565 |
| GNPDA1 | Lung adenocarcinoma | 0.40 | 2 | 0.817 | -0.02 | 0.04 | 0.645 |
| ITGB2 | Lung adenocarcinoma | 24.56 | 21 | 0.267 | 0.02 | 0.01 | 0.156 |
| SEPTIN3 | Lung adenocarcinoma | 11.95 | 15 | 0.683 | 0.00 | 0.01 | 0.752 |
| AGRN | Lung adenocarcinoma | 6.08 | 12 | 0.912 | 0.00 | 0.01 | 0.890 |
| DMP1 | Squamous Cell Lung Cancer | 14.62 | 19 | 0.746 | -0.04 | 0.02 | 0.054 |
| **EDA2R** | **Squamous Cell Lung Cancer** | **21.83** | **11** | **0.026** | **-0.04** | **0.02** | **0.145** |
| **EDA2R**^*^ | **Squamous Cell Lung Cancer** | **2.53** | **8** | **0.960** | **0.03** | **0.04** | **0.519** |
| HEPH | Squamous Cell Lung Cancer | 10.94 | 7 | 0.141 | -0.02 | 0.02 | 0.239 |
| DPP10 | Squamous Cell Lung Cancer | 4.50 | 8 | 0.809 | 0.01 | 0.01 | 0.660 |
| GP1BA | Squamous Cell Lung Cancer | 24.41 | 30 | 0.753 | 0.00 | 0.01 | 0.983 |
| C7 | Squamous Cell Lung Cancer | 14.00 | 13 | 0.374 | -0.01 | 0.02 | 0.561 |
| ACAN | Squamous Cell Lung Cancer | 27.26 | 23 | 0.245 | 0.01 | 0.01 | 0.366 |
| CEACAM6 | Small cell lung cancer | 32.98 | 31 | 0.370 | -0.01 | 0.01 | 0.534 |
| IL12RB2 | Small cell lung cancer | 2.15 | 1 | 0.142 | - | - | - |
| CPXM2 | Small cell lung cancer | 4.79 | 5 | 0.442 | -0.03 | 0.03 | 0.312 |
| ACADSB | Small cell lung cancer | 6.67 | 8 | 0.573 | 0.00 | 0.02 | 0.814 |
| COX6B1 | Small cell lung cancer | 0.01 | 1 | 0.931 | - | - | - |
| *The results uesd radial MR to remove outliers. | |  |  |  |  |  |  |

| **Supplemental table 10. Results of Gene Ontology (GO) enrichment of five identified lung adenocarcinoma related proteins.** | | | | | | | | | |
| --- | --- | --- | --- | --- | --- | --- | --- | --- | --- |
| **Ontology** | **ID** | **Description** | **GeneRatio** | **BgRatio** | ***P*** | **p.adjust** | **qvalue** | **geneID** | **Count** |
| BP | GO:0043113 | receptor clustering | 2/5 | 57/18903 | 8.88E-05 | 1.61E-02 | 6.45E-03 | ITGB2/AGRN | 2 |
| BP | GO:0006054 | N-acetylneuraminate metabolic process | 1/5 | 11/18903 | 2.91E-03 | 4.58E-02 | 1.84E-02 | GNPDA1 | 1 |
| BP | GO:0046349 | amino sugar biosynthetic process | 1/5 | 11/18903 | 2.91E-03 | 4.58E-02 | 1.84E-02 | GNPDA1 | 1 |
| BP | GO:0097278 | complement-dependent cytotoxicity | 1/5 | 11/18903 | 2.91E-03 | 4.58E-02 | 1.84E-02 | CD5L | 1 |
| BP | GO:0043312 | neutrophil degranulation | 1/5 | 12/18903 | 3.17E-03 | 4.58E-02 | 1.84E-02 | ITGB2 | 1 |
| BP | GO:0045161 | neuronal ion channel clustering | 1/5 | 12/18903 | 3.17E-03 | 4.58E-02 | 1.84E-02 | AGRN | 1 |
| BP | GO:0006047 | UDP-N-acetylglucosamine metabolic process | 1/5 | 14/18903 | 3.70E-03 | 4.58E-02 | 1.84E-02 | GNPDA1 | 1 |
| BP | GO:0046348 | amino sugar catabolic process | 1/5 | 14/18903 | 3.70E-03 | 4.58E-02 | 1.84E-02 | GNPDA1 | 1 |
| BP | GO:0002697 | regulation of immune effector process | 2/5 | 379/18903 | 3.85E-03 | 4.58E-02 | 1.84E-02 | CD5L/ITGB2 | 2 |
| BP | GO:1902563 | regulation of neutrophil activation | 1/5 | 15/18903 | 3.96E-03 | 4.58E-02 | 1.84E-02 | ITGB2 | 1 |
| BP | GO:0006044 | N-acetylglucosamine metabolic process | 1/5 | 18/18903 | 4.75E-03 | 4.58E-02 | 1.84E-02 | GNPDA1 | 1 |
| BP | GO:0002283 | neutrophil activation involved in immune response | 1/5 | 19/18903 | 5.02E-03 | 4.58E-02 | 1.84E-02 | ITGB2 | 1 |
| BP | GO:0007213 | G protein-coupled acetylcholine receptor signaling pathway | 1/5 | 19/18903 | 5.02E-03 | 4.58E-02 | 1.84E-02 | AGRN | 1 |
| BP | GO:0009226 | nucleotide-sugar biosynthetic process | 1/5 | 19/18903 | 5.02E-03 | 4.58E-02 | 1.84E-02 | GNPDA1 | 1 |
| BP | GO:0032930 | positive regulation of superoxide anion generation | 1/5 | 19/18903 | 5.02E-03 | 4.58E-02 | 1.84E-02 | ITGB2 | 1 |
| BP | GO:0002888 | positive regulation of myeloid leukocyte mediated immunity | 1/5 | 20/18903 | 5.28E-03 | 4.58E-02 | 1.84E-02 | ITGB2 | 1 |
| BP | GO:0042053 | regulation of dopamine metabolic process | 1/5 | 20/18903 | 5.28E-03 | 4.58E-02 | 1.84E-02 | ITGB2 | 1 |
| BP | GO:0042069 | regulation of catecholamine metabolic process | 1/5 | 20/18903 | 5.28E-03 | 4.58E-02 | 1.84E-02 | ITGB2 | 1 |
| BP | GO:0030449 | regulation of complement activation | 1/5 | 22/18903 | 5.81E-03 | 4.58E-02 | 1.84E-02 | CD5L | 1 |
| BP | GO:0032928 | regulation of superoxide anion generation | 1/5 | 22/18903 | 5.81E-03 | 4.58E-02 | 1.84E-02 | ITGB2 | 1 |
| BP | GO:0071404 | cellular response to low-density lipoprotein particle stimulus | 1/5 | 22/18903 | 5.81E-03 | 4.58E-02 | 1.84E-02 | ITGB2 | 1 |
| BP | GO:0043302 | positive regulation of leukocyte degranulation | 1/5 | 25/18903 | 6.60E-03 | 4.58E-02 | 1.84E-02 | ITGB2 | 1 |
| BP | GO:1901071 | glucosamine-containing compound metabolic process | 1/5 | 25/18903 | 6.60E-03 | 4.58E-02 | 1.84E-02 | GNPDA1 | 1 |
| BP | GO:1904996 | positive regulation of leukocyte adhesion to vascular endothelial cell | 1/5 | 25/18903 | 6.60E-03 | 4.58E-02 | 1.84E-02 | ITGB2 | 1 |
| BP | GO:0090314 | positive regulation of protein targeting to membrane | 1/5 | 28/18903 | 7.39E-03 | 4.58E-02 | 1.84E-02 | ITGB2 | 1 |
| BP | GO:0095500 | acetylcholine receptor signaling pathway | 1/5 | 29/18903 | 7.65E-03 | 4.58E-02 | 1.84E-02 | AGRN | 1 |
| BP | GO:0051491 | positive regulation of filopodium assembly | 1/5 | 31/18903 | 8.17E-03 | 4.58E-02 | 1.84E-02 | AGRN | 1 |
| BP | GO:1905145 | cellular response to acetylcholine | 1/5 | 31/18903 | 8.17E-03 | 4.58E-02 | 1.84E-02 | AGRN | 1 |
| BP | GO:0033238 | regulation of cellular amine metabolic process | 1/5 | 34/18903 | 8.96E-03 | 4.58E-02 | 1.84E-02 | ITGB2 | 1 |
| BP | GO:0055094 | response to lipoprotein particle | 1/5 | 34/18903 | 8.96E-03 | 4.58E-02 | 1.84E-02 | ITGB2 | 1 |
| BP | GO:0090313 | regulation of protein targeting to membrane | 1/5 | 34/18903 | 8.96E-03 | 4.58E-02 | 1.84E-02 | ITGB2 | 1 |
| BP | GO:0090322 | regulation of superoxide metabolic process | 1/5 | 34/18903 | 8.96E-03 | 4.58E-02 | 1.84E-02 | ITGB2 | 1 |
| BP | GO:1905144 | response to acetylcholine | 1/5 | 34/18903 | 8.96E-03 | 4.58E-02 | 1.84E-02 | AGRN | 1 |
| BP | GO:0002446 | neutrophil mediated immunity | 1/5 | 35/18903 | 9.22E-03 | 4.58E-02 | 1.84E-02 | ITGB2 | 1 |
| BP | GO:0009225 | nucleotide-sugar metabolic process | 1/5 | 37/18903 | 9.75E-03 | 4.58E-02 | 1.84E-02 | GNPDA1 | 1 |
| BP | GO:0071402 | cellular response to lipoprotein particle stimulus | 1/5 | 37/18903 | 9.75E-03 | 4.58E-02 | 1.84E-02 | ITGB2 | 1 |
| BP | GO:0098926 | postsynaptic signal transduction | 1/5 | 38/18903 | 1.00E-02 | 4.58E-02 | 1.84E-02 | AGRN | 1 |
| BP | GO:0097242 | amyloid-beta clearance | 1/5 | 39/18903 | 1.03E-02 | 4.58E-02 | 1.84E-02 | ITGB2 | 1 |
| BP | GO:1904994 | regulation of leukocyte adhesion to vascular endothelial cell | 1/5 | 40/18903 | 1.05E-02 | 4.58E-02 | 1.84E-02 | ITGB2 | 1 |
| BP | GO:0006040 | amino sugar metabolic process | 1/5 | 41/18903 | 1.08E-02 | 4.58E-02 | 1.84E-02 | GNPDA1 | 1 |
| BP | GO:0042119 | neutrophil activation | 1/5 | 41/18903 | 1.08E-02 | 4.58E-02 | 1.84E-02 | ITGB2 | 1 |
| BP | GO:0042417 | dopamine metabolic process | 1/5 | 41/18903 | 1.08E-02 | 4.58E-02 | 1.84E-02 | ITGB2 | 1 |
| BP | GO:0042554 | superoxide anion generation | 1/5 | 43/18903 | 1.13E-02 | 4.58E-02 | 1.84E-02 | ITGB2 | 1 |
| BP | GO:0042551 | neuron maturation | 1/5 | 45/18903 | 1.18E-02 | 4.58E-02 | 1.84E-02 | AGRN | 1 |
| BP | GO:0002920 | regulation of humoral immune response | 1/5 | 46/18903 | 1.21E-02 | 4.58E-02 | 1.84E-02 | CD5L | 1 |
| BP | GO:0001774 | microglial cell activation | 1/5 | 47/18903 | 1.24E-02 | 4.58E-02 | 1.84E-02 | ITGB2 | 1 |
| BP | GO:0036230 | granulocyte activation | 1/5 | 47/18903 | 1.24E-02 | 4.58E-02 | 1.84E-02 | ITGB2 | 1 |
| BP | GO:0007528 | neuromuscular junction development | 1/5 | 48/18903 | 1.26E-02 | 4.58E-02 | 1.84E-02 | AGRN | 1 |
| BP | GO:0035987 | endodermal cell differentiation | 1/5 | 49/18903 | 1.29E-02 | 4.58E-02 | 1.84E-02 | ITGB2 | 1 |
| BP | GO:0043300 | regulation of leukocyte degranulation | 1/5 | 49/18903 | 1.29E-02 | 4.58E-02 | 1.84E-02 | ITGB2 | 1 |
| BP | GO:0002269 | leukocyte activation involved in inflammatory response | 1/5 | 50/18903 | 1.32E-02 | 4.58E-02 | 1.84E-02 | ITGB2 | 1 |
| BP | GO:0051489 | regulation of filopodium assembly | 1/5 | 51/18903 | 1.34E-02 | 4.58E-02 | 1.84E-02 | AGRN | 1 |
| BP | GO:1903307 | positive regulation of regulated secretory pathway | 1/5 | 51/18903 | 1.34E-02 | 4.58E-02 | 1.84E-02 | ITGB2 | 1 |
| BP | GO:0006968 | cellular defense response | 1/5 | 52/18903 | 1.37E-02 | 4.59E-02 | 1.84E-02 | CD5L | 1 |
| BP | GO:0061900 | glial cell activation | 1/5 | 54/18903 | 1.42E-02 | 4.59E-02 | 1.84E-02 | ITGB2 | 1 |
| BP | GO:0006584 | catecholamine metabolic process | 1/5 | 55/18903 | 1.45E-02 | 4.59E-02 | 1.84E-02 | ITGB2 | 1 |
| BP | GO:0009712 | catechol-containing compound metabolic process | 1/5 | 55/18903 | 1.45E-02 | 4.59E-02 | 1.84E-02 | ITGB2 | 1 |
| BP | GO:0061756 | leukocyte adhesion to vascular endothelial cell | 1/5 | 57/18903 | 1.50E-02 | 4.68E-02 | 1.88E-02 | ITGB2 | 1 |
| BP | GO:0001706 | endoderm formation | 1/5 | 58/18903 | 1.52E-02 | 4.68E-02 | 1.88E-02 | ITGB2 | 1 |
| BP | GO:0002886 | regulation of myeloid leukocyte mediated immunity | 1/5 | 60/18903 | 1.58E-02 | 4.76E-02 | 1.91E-02 | ITGB2 | 1 |
| BP | GO:0034113 | heterotypic cell-cell adhesion | 1/5 | 61/18903 | 1.60E-02 | 4.76E-02 | 1.91E-02 | ITGB2 | 1 |
| BP | GO:0051965 | positive regulation of synapse assembly | 1/5 | 63/18903 | 1.66E-02 | 4.83E-02 | 1.94E-02 | AGRN | 1 |
| BP | GO:0031343 | positive regulation of cell killing | 1/5 | 66/18903 | 1.73E-02 | 4.98E-02 | 2.00E-02 | CD5L | 1 |
| BP | GO:0031638 | zymogen activation | 1/5 | 67/18903 | 1.76E-02 | 4.98E-02 | 2.00E-02 | CD5L | 1 |
| CC | GO:0099569 | presynaptic cytoskeleton | 1/5 | 10/19869 | 2.51E-03 | 2.81E-02 | 9.52E-03 | SEPTIN3 | 1 |
| CC | GO:0005940 | septin ring | 1/5 | 14/19869 | 3.52E-03 | 2.81E-02 | 9.52E-03 | SEPTIN3 | 1 |
| CC | GO:0031105 | septin complex | 1/5 | 14/19869 | 3.52E-03 | 2.81E-02 | 9.52E-03 | SEPTIN3 | 1 |
| CC | GO:0032156 | septin cytoskeleton | 1/5 | 16/19869 | 4.02E-03 | 2.81E-02 | 9.52E-03 | SEPTIN3 | 1 |
| CC | GO:0008305 | integrin complex | 1/5 | 31/19869 | 7.78E-03 | 4.36E-02 | 1.47E-02 | ITGB2 | 1 |
| MF | GO:0038024 | cargo receptor activity | 2/5 | 82/18432 | 1.94E-04 | 6.59E-03 | 2.45E-03 | CD5L/ITGB2 | 2 |
| MF | GO:0035374 | chondroitin sulfate binding | 1/5 | 10/18432 | 2.71E-03 | 2.53E-02 | 9.41E-03 | AGRN | 1 |
| MF | GO:0001851 | complement component C3b binding | 1/5 | 11/18432 | 2.98E-03 | 2.53E-02 | 9.41E-03 | ITGB2 | 1 |
| MF | GO:0002162 | dystroglycan binding | 1/5 | 11/18432 | 2.98E-03 | 2.53E-02 | 9.41E-03 | AGRN | 1 |
| MF | GO:0043395 | heparan sulfate proteoglycan binding | 1/5 | 18/18432 | 4.87E-03 | 2.86E-02 | 1.06E-02 | AGRN | 1 |
| MF | GO:0001846 | opsonin binding | 1/5 | 21/18432 | 5.68E-03 | 2.86E-02 | 1.06E-02 | ITGB2 | 1 |
| MF | GO:0033691 | sialic acid binding | 1/5 | 22/18432 | 5.95E-03 | 2.86E-02 | 1.06E-02 | AGRN | 1 |
| MF | GO:0001848 | complement binding | 1/5 | 26/18432 | 7.03E-03 | 2.86E-02 | 1.06E-02 | ITGB2 | 1 |
| MF | GO:0043236 | laminin binding | 1/5 | 28/18432 | 7.57E-03 | 2.86E-02 | 1.06E-02 | AGRN | 1 |
| MF | GO:0019239 | deaminase activity | 1/5 | 33/18432 | 8.92E-03 | 3.01E-02 | 1.12E-02 | GNPDA1 | 1 |
| MF | GO:0043394 | proteoglycan binding | 1/5 | 36/18432 | 9.73E-03 | 3.01E-02 | 1.12E-02 | AGRN | 1 |
| MF | GO:0005044 | scavenger receptor activity | 1/5 | 47/18432 | 1.27E-02 | 3.59E-02 | 1.34E-02 | CD5L | 1 |
| MF | GO:0016860 | intramolecular oxidoreductase activity | 1/5 | 51/18432 | 1.38E-02 | 3.60E-02 | 1.34E-02 | GNPDA1 | 1 |
| MF | GO:0050840 | extracellular matrix binding | 1/5 | 56/18432 | 1.51E-02 | 3.67E-02 | 1.36E-02 | AGRN | 1 |

| **Supplemental table 11. Results of Gene Ontology (GO) enrichment of six identified lung squamous cell carcinoma related proteins.** | | | | | | | | | |
| --- | --- | --- | --- | --- | --- | --- | --- | --- | --- |
| **Ontology** | **ID** | **Description** | **GeneRatio** | **BgRatio** | ***P*** | **p.adjust** | **qvalue** | **geneID** | **Count** |
| CC | GO:0031362 | anchored component of external side of plasma membrane | 1/6 | 20/19869 | 6.03E-03 | 4.30E-02 | 2.47E-02 | GP1BA | 1 |
| CC | GO:0090665 | glycoprotein complex | 1/6 | 22/19869 | 6.63E-03 | 4.30E-02 | 2.47E-02 | GP1BA | 1 |
| CC | GO:0031233 | intrinsic component of external side of plasma membrane | 1/6 | 24/19869 | 7.23E-03 | 4.30E-02 | 2.47E-02 | GP1BA | 1 |
| CC | GO:0046930 | pore complex | 1/6 | 26/19869 | 7.83E-03 | 4.30E-02 | 2.47E-02 | C7 | 1 |
| MF | GO:0016722 | oxidoreductase activity, acting on metal ions | 1/5 | 16/18432 | 4.33E-03 | 3.47E-02 | 7.69E-03 | HEPH | 1 |
| MF | GO:0030021 | extracellular matrix structural constituent conferring compression resistance | 1/5 | 22/18432 | 5.95E-03 | 3.47E-02 | 7.69E-03 | ACAN | 1 |
| MF | GO:0005540 | hyaluronic acid binding | 1/5 | 24/18432 | 6.49E-03 | 3.47E-02 | 7.69E-03 | ACAN | 1 |
| MF | GO:0008198 | ferrous iron binding | 1/5 | 27/18432 | 7.30E-03 | 3.47E-02 | 7.69E-03 | HEPH | 1 |
| MF | GO:0015459 | potassium channel regulator activity | 1/5 | 54/18432 | 1.46E-02 | 4.46E-02 | 9.89E-03 | DPP10 | 1 |
| MF | GO:0050840 | extracellular matrix binding | 1/5 | 56/18432 | 1.51E-02 | 4.46E-02 | 9.89E-03 | DMP1 | 1 |
| MF | GO:0005507 | copper ion binding | 1/5 | 61/18432 | 1.64E-02 | 4.46E-02 | 9.89E-03 | HEPH | 1 |

| **Supplemental table 12. Results of Gene Ontology (GO) enrichment of five identified lung small cell carcinoma related proteins.** | | | | | | | | | |
| --- | --- | --- | --- | --- | --- | --- | --- | --- | --- |
| **Ontology** | **ID** | **Description** | **GeneRatio** | **BgRatio** | ***P*** | **p.adjust** | **qvalue** | **geneID** | **Count** |
| MF | GO:0003995 | acyl-CoA dehydrogenase activity | 1/5 | 12/18432 | 3.25E-03 | 2.84E-02 | 4.27E-03 | ACADSB | 1 |
| MF | GO:0052890 | oxidoreductase activity, acting on the CH-CH group of donors, with a flavin as acceptor | 1/5 | 12/18432 | 3.25E-03 | 2.84E-02 | 4.27E-03 | ACADSB | 1 |
| MF | GO:0004129 | cytochrome-c oxidase activity | 1/5 | 19/18432 | 5.14E-03 | 2.84E-02 | 4.27E-03 | COX6B1 | 1 |
| MF | GO:0016675 | oxidoreductase activity, acting on a heme group of donors | 1/5 | 20/18432 | 5.41E-03 | 2.84E-02 | 4.27E-03 | COX6B1 | 1 |
| MF | GO:0004181 | metallocarboxypeptidase activity | 1/5 | 29/18432 | 7.84E-03 | 3.29E-02 | 4.95E-03 | CPXM2 | 1 |
| MF | GO:0004180 | carboxypeptidase activity | 1/5 | 44/18432 | 1.19E-02 | 4.16E-02 | 6.25E-03 | CPXM2 | 1 |
| MF | GO:0016627 | oxidoreductase activity, acting on the CH-CH group of donors | 1/5 | 60/18432 | 1.62E-02 | 4.46E-02 | 6.71E-03 | ACADSB | 1 |
| MF | GO:0008235 | metalloexopeptidase activity | 1/5 | 66/18432 | 1.78E-02 | 4.46E-02 | 6.71E-03 | CPXM2 | 1 |
| MF | GO:0015453 | oxidoreduction-driven active transmembrane transporter activity | 1/5 | 71/18432 | 1.91E-02 | 4.46E-02 | 6.71E-03 | COX6B1 | 1 |
| MF | GO:0050660 | flavin adenine dinucleotide binding | 1/5 | 87/18432 | 2.34E-02 | 4.60E-02 | 6.92E-03 | ACADSB | 1 |
| MF | GO:0004896 | cytokine receptor activity | 1/5 | 97/18432 | 2.60E-02 | 4.60E-02 | 6.92E-03 | IL12RB2 | 1 |
| MF | GO:0008238 | exopeptidase activity | 1/5 | 98/18432 | 2.63E-02 | 4.60E-02 | 6.92E-03 | CPXM2 | 1 |

| **Supplementary table 13a. Biological functions of proteins related to lung adenocarcinoma supported by GO analysis.** | | | | | | | |
| --- | --- | --- | --- | --- | --- | --- | --- |
| **ID** | **Description** | ***P*** | **geneID** | **Definition** | **biological interpretation** | **References** |  |
| GO:0038024 | cargo receptor activity | 1.94E-04 | CD5L/ITGB2 | Binding specifically to a substance (cargo) to deliver it to a transport vesicle. Cargo receptors span membranes (for instance the plasma membrane or the endoplasmic reticulum membrane), binding simultaneously to cargo molecules and coat adaptors, to efficiently recruit the cargo molecules to nascent vesicles. | Importins and exportins are overexpressed in lung cancer. Furthermore, some of the karyopherin-β proteins such as exportin-1 have been implicated in drug resistance in cancer. Importin and exportin inhibitors are being considered as therapeutic targets against cancer and have shown preclinical anticancer activity [1]. | [1] Mahipal A, Malafa M. Importins and exportins as therapeutic targets in cancer. Pharmacol Ther. Aug 2016;164:135-43. |  |
|  |  |  |  |  |  |  |  |
| **Supplementary table 13b. Biological functions of proteins related to lung squamous cell carcinoma supported by GO analysis.** | | | | | | | |
| **ID** | **Description** | ***P*** | **geneID** | **Definition** | **biological interpretation** | **References** |  |
| GO:0016722 | oxidoreductase activity, acting on metal ions | 4.33E-03 | HEPH | Catalysis of an oxidation-reduction in which the oxidation state of metal ion is altered. | Hypoxia is a common feature of lung squamous cell carcinoma [1]. In lung cancer, alterations in redox balance are extensively observed and are a consequence of disease as well as co-occurrent with smoking. Parameters related to lipid alterations are associated with oxidative stress in lung cancer patients [2]. | [1] Han P, Zhang B, Li Y, et al. MiR-183-5p inhibits lung squamous cell carcinoma survival through disrupting hypoxia adaptation mediated by HIF-1α/NDUFA4L2 axis. Oncogene. Sep 2024;43(38):2821-2834.  [2] Zabłocka-Słowińska K, Płaczkowska S, Skórska K, et al. Oxidative stress in lung cancer patients is associated with altered serum markers of lipid metabolism. PLoS One. 2019;14(4):e0215246. |  |
| GO:0030021 | extracellular matrix structural constituent conferring compression resistance | 5.95E-03 | GP1BA | A constituent of the extracellular matrix that enables the matrix to resist compressive forces; often a proteoglycan. | Glypican-3 expression in squamous cell carcinoma as an oncofetal protein renders it a potential candidate marker for early detection of lung squamous cell carcinoma [1]. The study demonstrates that lung cancer growth is suppressed through the downregulation of glypican-3/Wnt/β-catenin signaling, which is associated with the activation of autophagy. These findings suggest that glypican-3 may serve as a promising therapeutic target of rapamycin in the treatment of lung squamous cell carcinoma [2]. Previous study conclude that syndecan-1 expression decreases in parallel with histological dedifferentiation in squamous cell carcinoma of the lung, and that low syndecan-1 expression is associated with unfavourable outcome [3]. | [1] Aviel-Ronen S, Lau SK, Pintilie M, et al. Glypican-3 is overexpressed in lung squamous cell carcinoma, but not in adenocarcinoma. Mod Pathol. Jul 2008;21(7):817-25.  [2] Bi Y, Jiang Y, Li X, Hou G, Li K. Rapamycin inhibits lung squamous cell carcinoma growth by downregulating glypican-3/Wnt/β-catenin signaling and autophagy. J Cancer Res Clin Oncol. Feb 2021;147(2):499-505. [3] Anttonen A, Heikkilä P, Kajanti M, Jalkanen M, Joensuu H. High syndecan-1 expression is associated with favourable outcome in squamous cell lung carcinoma treated with radical surgery. Lung Cancer. Jun 2001;32(3):297-305. |  |
|  |  |  |  |  |  |  |  |
| **Supplementary table 13c. Biological functions of proteins related to small cell lung carcinoma supported by GO analysis.** | | | | | | | |
| **ID** | **Description** | ***P*** | **geneID** | **Definition** | **biological interpretation** | **References** |  |
| GO:0003995 | acyl-CoA dehydrogenase activity | ACADSB | 3.25E-03 | Catalysis of the reaction: a 2,3-saturated acyl-CoA + H+ oxidized [electron-transfer flavoprotein] = a (2E)-enoyl-CoA + reduced [electron-transfer flavoprotein]. | Acyl-CoA dehydrogenase activity catalyzes the first step of fatty acid β-oxidation in fatty acid metabolism and serves as a key enzymatic function in the energy metabolism of fatty acids. A recent study identified a phenotypic transition from non-small cell lung cancer to small cell lung cancer, accompanied by a metabolic shift from glucose metabolism to fatty acid metabolism [1]. Furthermore, among small cell lung cancer patients treated with chemotherapy, those harboring more mutations in the fatty acid metabolism pathway showed better prognosis [2]. | [1] Zhao QY, Liu WJ, Wang JG, et al. Increasing cisplatin exposure promotes small-cell lung cancer transformation after a shift from glucose metabolism to fatty acid metabolism. J Cancer Res Clin Oncol. Mar 28 2025;151(3):126.  [2] Lyu Q, Zhu W, Wei T, et al. High mutations in fatty acid metabolism contribute to a better prognosis of small-cell lung cancer patients treated with chemotherapy. Cancer Med. Nov 2021;10(21):7863-7876. |  |
| GO:0052890 | oxidoreductase activity, acting on the CH-CH group of donors, with a flavin as acceptor | ACADSB | 3.25E-03 | Catalysis of an oxidation-reduction (redox) reaction in which a CH-CH group acts as a hydrogen or electron donor and reduces a flavin. | Elevated expression of mitochondrial-cytochrome c oxidase II (MT-CO2) increases flavin adenosine dinucleotide (FAD) levels in activating lysine-specific demethylase 1 (LSD1) to epigenetically upregulate JUN transcription, consequently promoting glutaminase-1 (GLS1) and glutaminolysis for tumor cell survival [1]. | [1] Yi Y, Wang G, Zhang W, et al. Mitochondrial-cytochrome c oxidase II promotes glutaminolysis to sustain tumor cell survival upon glucose deprivation. Nat Commun. Jan 2 2025;16(1):212. |  |
| GO:0004129 | cytochrome-c oxidase activity | COX6B1 | 5.14E-03 | Catalysis of the reaction: 4 Fe(II)-[cytochrome c] + O2 + 8 H+(in) = 4 Fe(III)-[cytochrome c] + 2 H2O + 4 H+(out). | Elevated expression of mitochondrial-cytochrome c oxidase II (MT-CO2) is associated with poor prognosis in lung cancer patients. The study shows that upregulation of the mitochondrial genome-encoded complex IV protein MT-CO2 is induced upon glucose deprivation to promote glutaminolysis through epigeneticmediated mechanisms [1]. | [1] Yi Y, Wang G, Zhang W, et al. Mitochondrial-cytochrome c oxidase II promotes glutaminolysis to sustain tumor cell survival upon glucose deprivation. Nat Commun. Jan 2 2025;16(1):212. |  |

**Supplementary table 14. Characteristics of studies about drugs targeting identified proteins on lung cancer subtypes.**

| **Protein** | **Drug or Component name** | **References** | **Year** | **Conclusion** |
| --- | --- | --- | --- | --- |
| ITGB2 | Simvastatin | Simvastatin Overcomes Resistance to Tyrosine Kinase Inhibitors in Patient-derived, Oncogene-driven Lung Adenocarcinoma Models | 2024 | The addition of simvastatin is a safe approach to overcome acquired resistance to TKIs in several oncogene-driven LUAD models, which deserve further investigation. |
|  |  | Simvastatin Enhanced Anti-tumor Effects of Bevacizumab against Lung Adenocarcinoma A549 Cells via Abating HIF-1α-Wnt/β-Catenin Signaling Pathway | 2023 | Based on our findings, simvastatin may affect the biological responses of bevacizumab on A549 cells by restraining the HIF-1α-Wnt/β-catenin signaling pathway, thus representing a novel and effective combination therapy that can be potentially applied in a clinical therapy for lung adenocarcinoma. |
|  |  | Therapeutic effects of statins against lung adenocarcinoma via p53 mutant-mediated apoptosis | 2019 | Significantly higher levels of cellular apoptosis, inhibited cell growth, and regulated lipid raft content were observed in mutant p53 lung cancer cells treated with simvastatin. Further, simvastatin increased the caspase-dependent apoptotic pathway, promotes mutant p53 protein degradation, and decreased motile activity in lung cancer cells with p53 missense mutations. |
|  |  | Statins may have double-edged effects in patients with lung adenocarcinoma after lung resection | 2019 | Statins suppress EMT and change the prognosis of patients with lung adenocarcinoma in a p53 mutation-dependent manner. |
|  |  | Statins associate with improved mortality among patients with certain histological subtypes of lung cancer | 2018 | There is consistent evidence indicating that baseline or post-diagnostic exposure to simvastatin and atorvastatin is associated with extended survival in non-small-cell lung cancer subtypes. |
|  |  | Simvastatin prevents proliferation and bone metastases of lung adenocarcinoma in vitro and in vivo | 2013 | The HMGR inhibitor simvastatin prevents proliferation and osteolytic bone metastases of lung adenocarcinoma cells in vitro and vivo. |
|  |  | A randomized phase II study of gefitinib plus simvastatin versus gefitinib alone in previously treated patients with advanced non-small cell lung cancer | 2011 | Simvastatin may improve the efficacy of gefitinib in that subgroup of gefitinib-resistant NSCLC patients. |
|  | BMS-587101 (Lifitegrast) | - | - | - |
| GP1BA | Ibuprofen | Preclinical Evidence for Combined Use of Aromatase Inhibitors and NSAIDs as Preventive Agents of Tobacco-Induced Lung Cancer | 2018 | Anastrozole combined with ibuprofen showed enhanced antitumor effects. |
|  |  | Ibuprofen and fatal lung cancer: A brief report of the prospective results from the Third National Health and Nutrition Examination Survey (NHANES III) | 2017 | Multivariate regression models revealed that regular use of ibuprofen resulted in a 48% reduced risk of lung cancer mortality (HR=0.52, 95% CI: 0.33-0.82, P<0.01). |
|  |  | Ibuprofen enhances the anticancer activity of cisplatin in lung cancer cells by inhibiting the heat shock protein 70 | 2014 | Our observations indicate that the suppression of Hsp70 by ibuprofen mediates the sensitivity to cisplatin by enhancing apoptosis at several stages of the mitochondrial cascade. |
|  |  | Reduction in cancer risk by selective and nonselective cyclooxygenase-2 (COX-2) inhibitors | 2012 | Ibuprofen use reduced the lung cancer development by 62%. |
|  |  | Reduced risk of human lung cancer by selective cyclooxygenase 2 (COX-2) blockade: results of a case control study | 2017 | Intake of ibuprofen produced significant risk reductions (OR=0.40, 95% CI=0.23-0.73). |
|  |  | Chemoprevention of lung cancer by non-steroidal anti-inflammatory drugs among cigarette smokers | 2002 | These results combined with the current molecular evidence suggest that ibuprofen intake may prevent tobacco carcinogenesis through COX-2 blockade. |
|  | Dexibuprofen | - | - | - |
| ACADSB | Valproic acid | Valproic acid increased the efficacy of EGFR TKIs on EGFR/TP53 co-mutated lung cancers and downregulated mutant-p53 levels | 2023 | This study suggested that valproic acid (VPA) combination treatment could have beneficial effects on EGFR-mutant lung cancers with concurrent p53 mutation in both early and late stages, expanding the potential clinical applications for VPA. |
|  |  | Anticancer Effects of Valproic Acid via Regulation of Epigenetic Mechanisms in Non-small-cell Lung Cancer A549 Cell Line | 2021 | The use of valproic acid can be effective in the suppression of metastases and the treatment of these tumors. |
|  |  | Combination of Arsenic Trioxide and Valproic Acid Efficiently Inhibits Growth of Lung Cancer Cells via G2/M-Phase Arrest and Apoptotic Cell Death | 2020 | The combination of Arsenic trioxide (ATO) / Valproic acid (VPA) effectively inhibited the growth of lung cancer cells through G2/M-phase arrest and apoptotic cell death, and had a synergistic antitumor effect in vivo. |
|  |  | Valproic acid (VPA) enhances cisplatin sensitivity of non-small cell lung cancer cells via HDAC2 mediated down regulation of ABCA1 | 2017 | It suggested that combination of Valproic acid (VPA) and anticancer drugs such as cisplatin (DDP) might be great helpful for treatment of NSCLC patients. |
|  |  | Valproic acid improves second-line regimen of small cell lung carcinoma in preclinical models | 2015 | Transcriptomic profiling integrating microRNA and mRNA data identifies key signalling pathways in the response of small cell lung carcinoma cells to valproic acid, opening new prospects for improved therapies. |
|  |  | Valproic acid, an inhibitor of class I histone deacetylases, reverses acquired Erlotinib-resistance of lung adenocarcinoma cells: a Connectivity Mapping analysis and an experimental study | 2015 | Valproic acid (VPA) might markedly increase the sensitivity of TKI-resistant lung adenocarcinoma cells to Erlotinib, thus reversing the acquired TKI-resistance of cancer cells and raising VPA as a potential agent for TKI-resistant lung cancer therapy. |
|  |  | Enhanced suppression of proliferation and migration in highly-metastatic lung cancer cells by combination of valproic acid and coumarin-3-carboxylic acid and its molecular mechanisms of action | 2013 | Our results suggest that the combination of Valproic acid (VPA) with coumarin-3-carboxylic acid suppresses the proliferation and migration of lung cancer cells via EGFR/VEGFR2/c-Met-Akt-NF-κB signaling pathways; this combination may have a wide therapeutic and/or adjuvant therapeutic application in the treatment of lung cancer. |
|  |  | Valproic acid induces Notch1 signaling in small cell lung cancer cells | 2008 | These results suggest that Valproic acid has potential as a novel therapeutic agent for small cell lung cancer. |
| COX6B1 | Cholic Acid | Comprehensive metabolomic analysis identifies key biomarkers and modulators of immunotherapy response in NSCLC patients | 2024 | Specific bile acids, glycochenodeoxycholic acid (GCDCA) and taurolithocholic acid (TLCA), are associated with better survival and therapeutic response |
|  |  | Application of a nomogram from coagulation-related biomarkers and C1q and total bile acids in distinguishing advanced and early-stage lung cancer | 2024 | Multiple regression analysis based on subgroup analysis of clinical stage showed that compared with early-stage lung cancer, total bile acids (P = 0.011) were negatively related to advanced lung cancer. |
|  |  | Chenodeoxycholic acid inhibits lung adenocarcinoma progression via the integrin α5β1/FAK/p53 signaling pathway | 2022 | Chenodeoxycholic acid (CDCA) inhibited LUAD cell proliferation, migration, and invasion. Furthermore, it promoted apoptosis in LUAD cells. Mechanistically, CDCA inhibited the integrin α5β |
|  |  | Evolutionary metabolic landscape from preneoplasia to invasive lung adenocarcinoma | 2021 | We identify correlation between aberrant bile acid metabolism in subtype III with poor clinical features and demonstrate dysregulated bile acid metabolism promotes migration of lung adenocarcinoma, which could be exploited as potential targetable vulnerability and for stratifying patients. |

| **Supplemental table 15. Detailed information of the differentially expressed genes between lung adenocarcinoma and normal samples in TCGA dataset.** | | | | | | |
| --- | --- | --- | --- | --- | --- | --- |
| **Gene** | **baseMean** | **log_2_FoldChange** | **lfcSE** | **stat** | ***P*** | ***P*_FDR_** |
| AGRN | 17984.97 | 0.56 | 0.12 | 4.74 | 2.14E-06 | 5.41E-06 |
| CD5L | 22.25 | -4.10 | 0.26 | -15.94 | 3.38E-57 | 2.11E-55 |
| ITGB2 | 10139.18 | -0.62 | 0.14 | -4.32 | 1.54E-05 | 3.53E-05 |
| SEPTIN3 | 349.90 | 1.54 | 0.20 | 7.56 | 3.97E-14 | 2.03E-13 |

| **Supplemental table 16. Detailed information of the differentially expressed genes between lung squamous cell carcinoma and normal samples in TCGA dataset.** | | | | | | |
| --- | --- | --- | --- | --- | --- | --- |
| **Gene** | **baseMean** | **log_2_FoldChange** | **lfcSE** | **stat** | ***P*** | ***P*_FDR_** |
| ACAN | 164.08 | 0.64 | 0.22 | 2.92 | 3.54E-03 | 5.21E-03 |
| C7 | 4379.75 | -3.38 | 0.27 | -12.32 | 7.14E-35 | 7.11E-34 |
| DMP1 | 11.57 | 3.22 | 0.27 | 12.11 | 9.79E-34 | 9.31E-33 |
| DPP10 | 88.61 | 1.08 | 0.38 | 2.82 | 4.84E-03 | 7.02E-03 |
| GP1BA | 65.13 | -1.21 | 0.17 | -6.94 | 4.01E-12 | 1.24E-11 |
| HEPH | 991.31 | -0.75 | 0.17 | -4.37 | 1.25E-05 | 2.38E-05 |

| **Supplemental table 17. Detailed information of the differentially expressed genes between lung small cell carcinoma and normal samples in GEO dataset.** | | | | | | |
| --- | --- | --- | --- | --- | --- | --- |
| **Gene** | **Log_2_FC** | **AveExpr** | **t** | ***P*** | ***P*_FDR_** | **B** |
| ACADSB | -0.64 | 5.04 | -2.89 | 6.61E-03 | 1.68E-02 | -3.42 |
| CEACAM6 | -4.75 | 8.32 | -8.08 | 1.60E-09 | 2.80E-08 | 11.55 |
| COX6B1 | 0.65 | 10.80 | 5.61 | 2.48E-06 | 1.71E-05 | 4.23 |
| CPXM2 | 0.19 | 4.52 | 2.75 | 9.34E-03 | 2.26E-02 | -3.75 |
| IL12RB2 | 0.64 | 4.12 | 2.09 | 4.41E-02 | 8.40E-02 | -5.15 |

| **Supplementary table 18a.** **Sensitivity analysis of logistic results for lung adenocarcinoma.** | | | | |  |
| --- | --- | --- | --- | --- | --- |
| **Protein** | **Univariate logistic regression** | |  | **Multivariable logistic regression** | |
|  | **OR (95%CI)** | ***P*** |  | **OR (95%CI)** | ***P*** |
| AGRN | 2.20 (1.59-3.03) | 1.91E-06 |  | 1.42 (0.97-2.07) | 6.84E-02 |
| CD5L | 1.28 (0.91-1.80) | 1.63E-01 |  | 1.28 (0.90-1.80) | 1.67E-01 |
| GNPDA1 | 1.63 (1.19-2.23) | 2.52E-03 |  | 1.55 (1.13-2.13) | 6.88E-03 |
| ITGB2 | 0.42 (0.26-0.69) | 6.03E-04 |  | 0.61 (0.36-1.02) | 5.79E-02 |
| SEPTIN3 | 1.38 (1.16-1.65) | 3.35E-04 |  | 1.23 (0.99-1.51) | 5.60E-02 |
|  |  |  |  |  |  |
| **Supplementary table 18b. Sensitivity analysis of logistic results for lung squamous cell carcinoma.** | | | | | |
| **Protein** | **Univariate logistic regression** | |  | **Multivariable logistic regression** | |
|  | **OR (95%CI)** | ***P*** |  | **OR (95%CI)** | ***P*** |
| ACAN | 0.50 (0.25-0.997) | 4.90E-02 |  | 0.51 (0.24-1.09) | 8.16E-02 |
| C7 | 3.15 (1.60-6.19) | 8.98E-04 |  | 1.70 (0.85-3.39) | 1.33E-01 |
| DMP1 | 0.54 (0.42-0.71) | 8.85E-06 |  | 0.63 (0.44-0.91) | 1.25E-02 |
| DPP10 | 2.35 (1.54-3.59) | 8.28E-05 |  | 1.55 (0.97-2.48) | 6.67E-02 |
| GP1BA | 0.38 (0.19-0.74) | 4.24E-03 |  | 0.42 (0.22-0.82) | 1.02E-02 |
| HEPH | 13.38 (6.17-29.04) | 5.35E-11 |  | 3.47 (1.39-8.67) | 7.61E-03 |
|  |  |  |  |  |  |
| **Supplementary table 18c. Sensitivity analysis of logistic results for lung small cell carcinoma.** | | | | | |
| **Protein** | **Univariate logistic regression** | |  | **Multivariable logistic regression** | |
|  | **OR (95%CI)** | ***P*** |  | **OR (95%CI)** | ***P*** |
| ACADSB | 0.61 (0.42-0.89) | 1.05E-02 |  | 0.63 (0.43-0.92) | 1.65E-02 |
| CEACAM6 | 3.14 (2.21-4.45) | 1.60E-10 |  | 2.54 (1.66-3.88) | 1.77E-05 |
| COX6B1 | 0.25 (0.09-0.70) | 8.84E-03 |  | 0.23 (0.08-0.67) | 6.60E-03 |
| CPXM2 | 3.47 (1.90-6.35) | 5.30E-05 |  | 2.87 (1.29-6.35) | 9.45E-03 |
| IL12RB2 | 0.19 (0.07-0.48) | 4.30E-04 |  | 0.26 (0.10-0.70) | 7.50E-03 |

| **Supplementary table 19. Reverse Two-sample Mendelian randomization results for lung cancer subtypes.** | | | | | | | |
| --- | --- | --- | --- | --- | --- | --- | --- |
| **Protein** | **Exposure** | **Method** | **Nsnp** | **Beta** | **SE** | ***P*** | **OR (95%CI)** |
| AGRN | Lung adenocarcinoma | Inverse variance weighted | 11 | -0.03 | 0.02 | 4.84E-02 | 0.97 (0.93-0.998) |
| CD5L | Lung adenocarcinoma | Inverse variance weighted | 11 | 0.00 | 0.02 | 8.50E-01 | 1.00 (0.96-1.05) |
| GNPDA1 | Lung adenocarcinoma | Inverse variance weighted | 11 | -0.02 | 0.02 | 3.48E-01 | 0.98 (0.95-1.02) |
| ITGB2 | Lung adenocarcinoma | Inverse variance weighted | 11 | 0.01 | 0.02 | 6.20E-01 | 1.01 (0.97-1.04) |
| SEPTIN3 | Lung adenocarcinoma | Inverse variance weighted | 11 | -0.01 | 0.02 | 5.33E-01 | 0.99 (0.95-1.02) |
| ACAN | Squamous cell carcinoma | Inverse variance weighted | 5 | -0.03 | 0.02 | 8.00E-02 | 0.97 (0.94-1.003) |
| C7 | Squamous cell carcinoma | Inverse variance weighted | 5 | 0.01 | 0.02 | 5.21E-01 | 1.01 (0.98-1.05) |
| DMP1 | Squamous cell carcinoma | Inverse variance weighted | 5 | -0.04 | 0.03 | 1.68E-01 | 0.96 (0.91-1.02) |
| DPP10 | Squamous cell carcinoma | Inverse variance weighted | 5 | -0.01 | 0.02 | 5.50E-01 | 0.99 (0.95-1.03) |
| GP1BA | Squamous cell carcinoma | Inverse variance weighted | 5 | -0.01 | 0.04 | 7.82E-01 | 0.99 (0.92-1.06) |
| HEPH | Squamous cell carcinoma | Inverse variance weighted | 5 | 0.02 | 0.04 | 5.47E-01 | 1.02 (0.95-1.09) |
| CEACAM6 | Small cell carcinoma | Wald ratio | 1 | 0.00 | 0.03 | 9.45E-01 | 1.00 (0.95-1.06) |
| IL12RB2 | Small cell carcinoma | Wald ratio | 1 | 0.06 | 0.03 | 2.39E-02 | 1.06 (1.01-1.12) |
| CPXM2 | Small cell carcinoma | Wald ratio | 1 | -0.02 | 0.03 | 3.85E-01 | 0.98 (0.92-1.03) |
| ACADSB | Small cell carcinoma | Wald ratio | 1 | 0.02 | 0.02 | 3.18E-01 | 1.03 (0.98-1.08) |
| COX6B1 | Small cell carcinoma | Wald ratio | 1 | -0.04 | 0.03 | 1.51E-01 | 0.96 (0.91-1.01) |
